# Metabolomics analysis reveals metabolite changes during freeze-drying and oven-drying of *Angelica dahurica*

**DOI:** 10.1038/s41598-023-32402-0

**Published:** 2023-04-13

**Authors:** Qinghua Wu, Qi Yan, Lan Jiang, Cuiping Chen, Xulong Huang, Xinglong Zhu, Tao Zhou, Jiang Chen, Jie Yan, Feiyan Wen, Jin Pei

**Affiliations:** 1 State Key Laboratory of Characteristic Chinese Medicine Resources in Southwest China, Chengdu, 611137 China; 2 School of Pharmacy, Chengdu University of Traditional Chinese Medicine, Chengdu, 611137 China

**Keywords:** Biochemistry, Biological techniques, Plant sciences

## Abstract

*Angelica dahurica* (*Angelica dahurica* Fisch. ex Hoffm.) is widely used as a traditional Chinese medicine and the secondary metabolites have significant pharmacological activities. Drying has been shown to be a key factor affecting the coumarin content of *Angelica dahurica*. However, the underlying mechanism of metabolism is unclear. This study sought to determine the key differential metabolites and metabolic pathways related to this phenomenon**.** Liquid chromatography with tandem mass spectrometry (LC–MS/MS) based targeted metabolomics analysis was performed on *Angelica dahurica* that were freeze-drying (− 80 °C/9 h) and oven-drying (60 °C/10 h). Furthermore, the common metabolic pathways of paired comparison groups were performed based on KEEG enrichment analysis. The results showed that 193 metabolites were identified as key differential metabolites, most of which were upregulated under oven drying. It also displayed that many significant contents of PAL pathways were changed. This study revealed the large-scale recombination events of metabolites in *Angelica dahurica*. First, we identified additional active secondary metabolites apart from coumarins, and volatile oil were significantly accumulated in *Angelica dahurica*. We further explored the specific metabolite changes and mechanism of the phenomenon of coumarin upregulation caused by temperature rise. These results provide a theoretical reference for future research on the composition and processing method of *Angelica dahurica*.

## Introduction

Commonly used in traditional Chinese medicine, *Angelica dahurica* has a long history with several varieties documented. The functions and flavors of *Angelica dahurica* are widely acknowledged to be mainly determined by ingredients such as coumarin, volatile oil, and polysaccharide. Importantly, coumarin is associated with antibacterial, anti-inflammatory, and anticancer effects^1^. Volatile oil and polysaccharide account for the anti-inflammatory, analgesic and antioxidant properties^2^. In addition to its medical purposes, *Angelica dahurica* is used as a raw material in cosmetics and flavoring agents^3^.

After collecting fresh Chinese medicinal materials, it is necessary to remove the non-medicinal parts and dry the leftovers in time. The optimal drying method and conditions can potentially promote the chemical transformation and the biotransformation of the chemical substances to extract the medicinal components and pave the way for good clinical efficacy^4^. It is widely believed that drying can directly affect Chinese medicine quality. The two drying approaches used in this study were oven-drying and freeze-drying. Interestingly, vacuum freeze-drying technology mainly applies the principles of water sublimation^5^. It is a drying method that turns ice directly into water vapor to remove unbound water^6^. In contrast, oven drying is a method of artificial heating by placing materials in an oven, drying room, or dryer, etc.^7^. Accordingly, the drying temperature and time can be controlled according to the characteristics of various medicinal materials.

Previous studies on *Angelica dahurica* emphasized specific drying methods, procedures and equipment. Experience and many repetitive experiments were required to compare different drying processes to evaluate the best drying method and key parameters. However, research at the molecular level in *Angelica dahurica* processing remains largely unexplored. Pei L. et al. explored the influence and change in chemical components of coumarin and volatile oil in *Angelica dahurica*; Interestingly, peeling and drying with hot air at 60 °C yielded the best results followed by peeling and drying with hot air at 40 °C and freeze drying^8^. Similarly, Jie J. et al. reported that the coumarin content in *Angelica dahurica* was greater drying at 60 °C than at 40°C^9^. A study that measured the constituents of coumarin in imperatorin (Compound CID: 10212) and isoimperatorin (Compound CID: 68081) (Supplementary table S4) reported the best results with drying at 60 °C followed by drying at 80 °C and 25 °C^10^. Current evidence suggests that the content of the coumarin of *Angelica dahurica* is increased at the beginning and then decreased with processing temperature, with a peak at 60 °C. Accordingly, we chose the drying temperature of 60 °C for research. It has been established that owing to thermal instability, the content of the medicinal components drops at high temperatures (> 70 °C). However, the mechanism underlying the upregulation of the medicinal components of *Angelica dahurica* with temperature has not yet been elucidated.

In recent years, metabolomic analysis has been applied to biological research, from physiological metabolism in plants to the development of personal metabolomics, and its application contributes to a better understanding of the complex molecular interactions within biological systems^11^. It has been documented that widely-targeted metabolomics integrates the advantages of non-target and targeted metabolite detection technologies, exhibiting high sensitivity and wide coverage^12^. Therefore, by characterizing the metabolic profiles of *Angelica dahurica* dried in lyophilizers and dryers, it is possible to provide a mechanistic link between changes in *Angelica dahurica* metabolism and the phenomenon of coumarin upregulation caused by temperature increases.

In this study, widely-targeted metabolomics was used to compare the metabolic profiles between oven-drying and freeze-drying of *Angelica dahurica* for the first time to understand the phenomenon of coumarin upregulation caused by increased temperature. This is the first study to reveal the large-scale recombination events of metabolites in *Angelica dahurica*, providing a comprehensive overview of the mechanism underlying coumarin upregulation caused by temperature increases.

## Results

### Phenotypic differences between freeze-drying and oven-drying pieces

**Figure 1:**
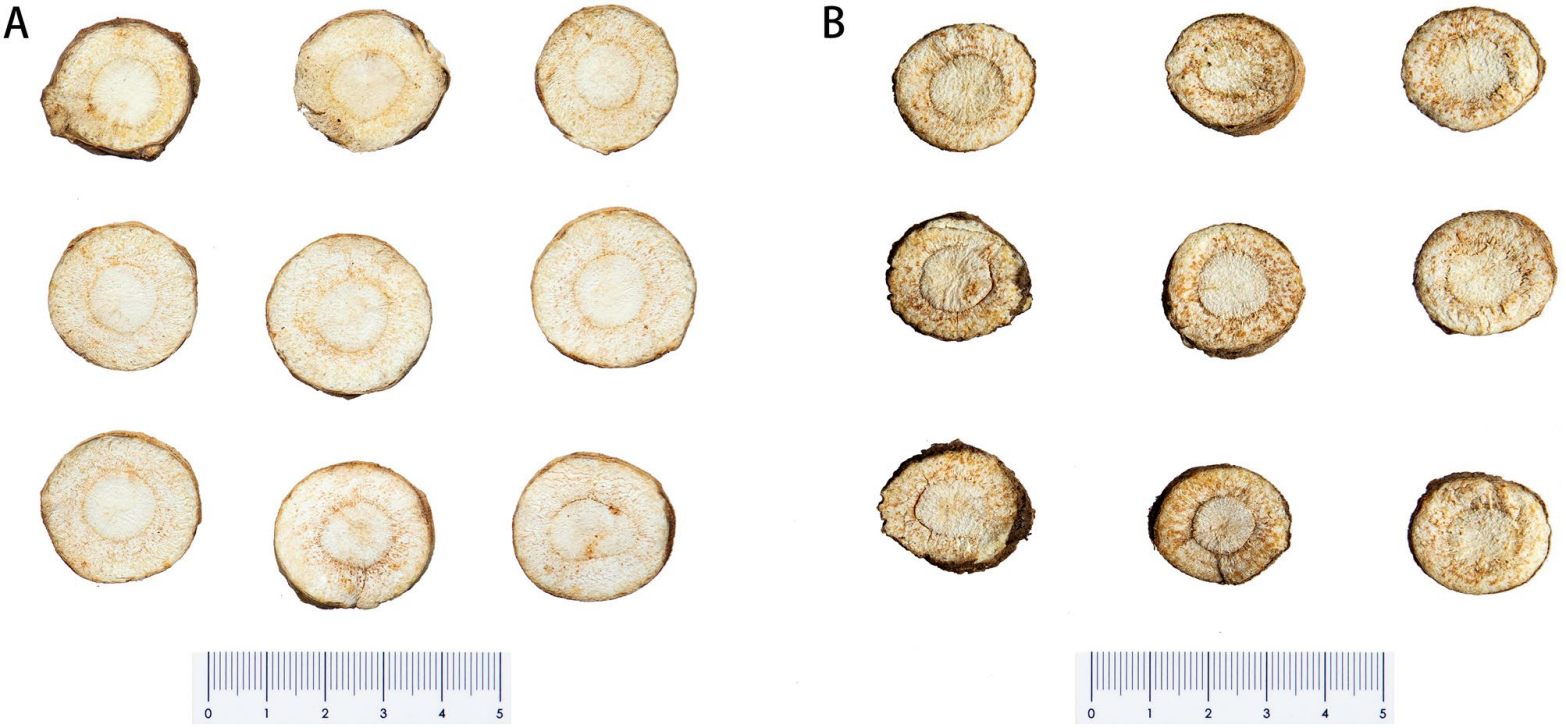
Oven-drying and freeze-dried pieces of *Angelica dahurica*. (**A**) Freeze-drying pieces; (**B**) Oven-drying pieces.

From the characteristics of the freeze-drying (Fig. 1A) and oven-drying (Fig. 1B) pieces, it can be seen that the oven-drying pieces were looser in texture, darker in color, and with a greater number of oil glands. The oil chamber represents a group of cells with secreting ability. Large quantities of plant metabolites were stored in the oil chamber, indicating that more metabolites accumulated after oven drying than freezedrying, and it was speculated that the coumarin content was also enhanced with increased temperature.

### The identification of metabolites of *Angelica dahurica*

The mass spectral data were processed using the software Analyst 1.6.3. The metabolites of the samples were qualitatively and quantitatively analyzed by mass spectrometry based on the local metabolic database. As shown in the figure (Supplementary fig. S2), the MRM metabolite detection peak map (multi-substance extracted ion chromatogram, XIC). It shows the substances that can be detected in the sample, and each mass spectrum peak of different colors represents a detected metabolite. The characteristic ions of each substance are screened out by triple quadrupole, the signal intensity (CPS) of the characteristic ions is obtained in the detector, the mass spectrum file of the sample is opened with MultiaQuant 3.0.3 software, and the integration and correction of chromatographic peaks are carried out. The peak area (Area) of the peak represents the relative content of the corresponding substance. Finally, all chromatographic peak area integration data are exported and saved.

### Overview of the metabolites of *Angelica dahurica*

**Figure 2:**
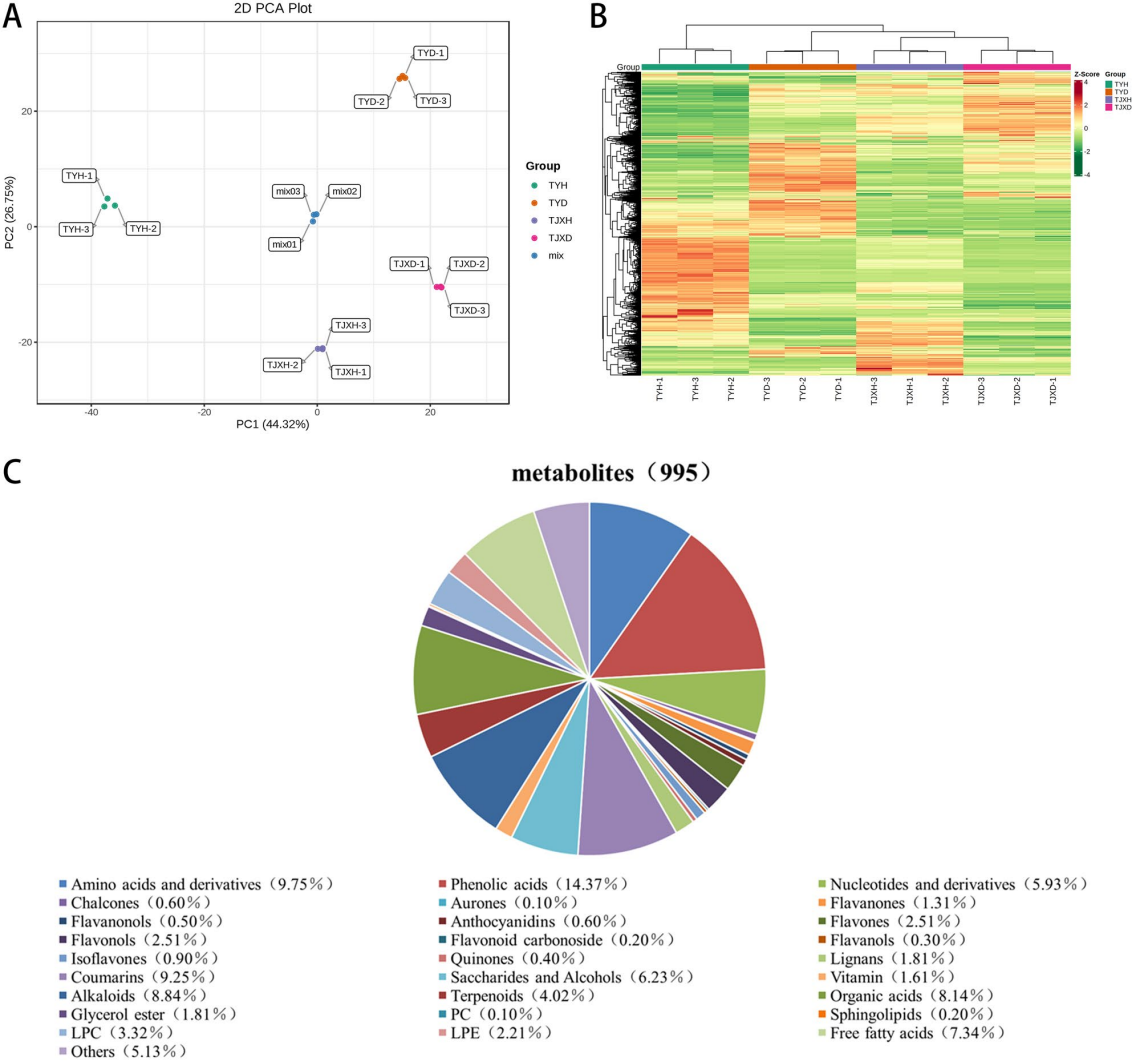
(**A**) PCA score plot of the metabolites in TYH, TYD, TJXH, and TJXD; (**B**) Heatmap of the metabolites in TYH, TYD, TJXH, and TJXD; (**C**) Classification of the 995 metabolites of *Angelica dahurica* samples.

To better understand the metabolite changes in *Angelica dahurica* after lyophilization and drying, the primary and secondary metabolites in the samples were identified by the UPLC-MS platform. A total of 995 metabolites were detected in the experiment divided into 28 classes, including amino acids and derivatives (n = 97), phenolic acids (n = 143), nucleotides and derivatives (n = 59), chalcones (n = 6), aurones (n = 1), flavanones (n = 13), flavanonols (n = 5), anthocyanidins (n = 6), flavones(n = 25), flavonols(n = 25), flavonoid carbonoside (n = 2), flavanols (n = 3), isoflavones (n = 9), quinones (n = 4), lignans (n = 18), coumarins (n = 92), saccharides and alcohols (n = 62), vitamin (n = 16), alkaloids (n = 88), terpenoids (n = 40), organic acids (n = 81), glycerol ester (n = 18), PC (n = 1), sphingolipids (n = 2), LPC (n = 33), LPE (n = 22), free fatty acids (n = 73), and others (n = 51). Among these differential metabolites, amino acids and derivatives (9.75%), phenolic acids (14.37%), coumarins (9.25%), alkaloids (8.84%), organic acids (8.14%), free fatty acids (7.34%) were the most abundant (Fig. 2C).

PCA of all treatments in the four groups samples (Fig. 2A) demonstrated that the *Angelica dahurica* under different drying approaches were separated, which indicated that the metabolic differences were prominent. Principal component 1 (PC1) and principal component 2 (PC2) accounted for 44.32% and 26.75% of the variance in the data, respectively. These results displayed that oven-drying and freeze-drying treatments accounted for differences in metabolites of PC1 and the groups from different plantation sites were mainly separated by PC2.

The cluster heatmap of metabolites in all samples is displayed in Fig. 2B. Some metabolites of *Angelica dahurica* were upregulated when treated in a drying oven but downregulated in a lyophilizer, suggesting significant differences in metabolites between oven-drying and freeze-drying treatment. The up regulation of ‘content’ is not an absolute increase or decrease, but a relative increase or decrease. During the screening of differential metabolites, if the grouping information is A vs B, it means that A is the control group and B is the experimental group for data analysis. If the final screening of differential metabolites is up-regulated, it means that the content of metabolite is relatively low in A, and relatively high in B. Four main clusters were obtained. Clusters 1 and 2 accumulated at high levels in TYD and TJXD. Clusters 3 and 4 accumulated at high levels in TYH and TJXH. Moreover, the three biological replications of each group were clustered together, indicating a good homogeneity between duplicates and the high reliability of the data.

### Widely targeted metabolite analysis based on OPLS-DA

**Figure 3:**
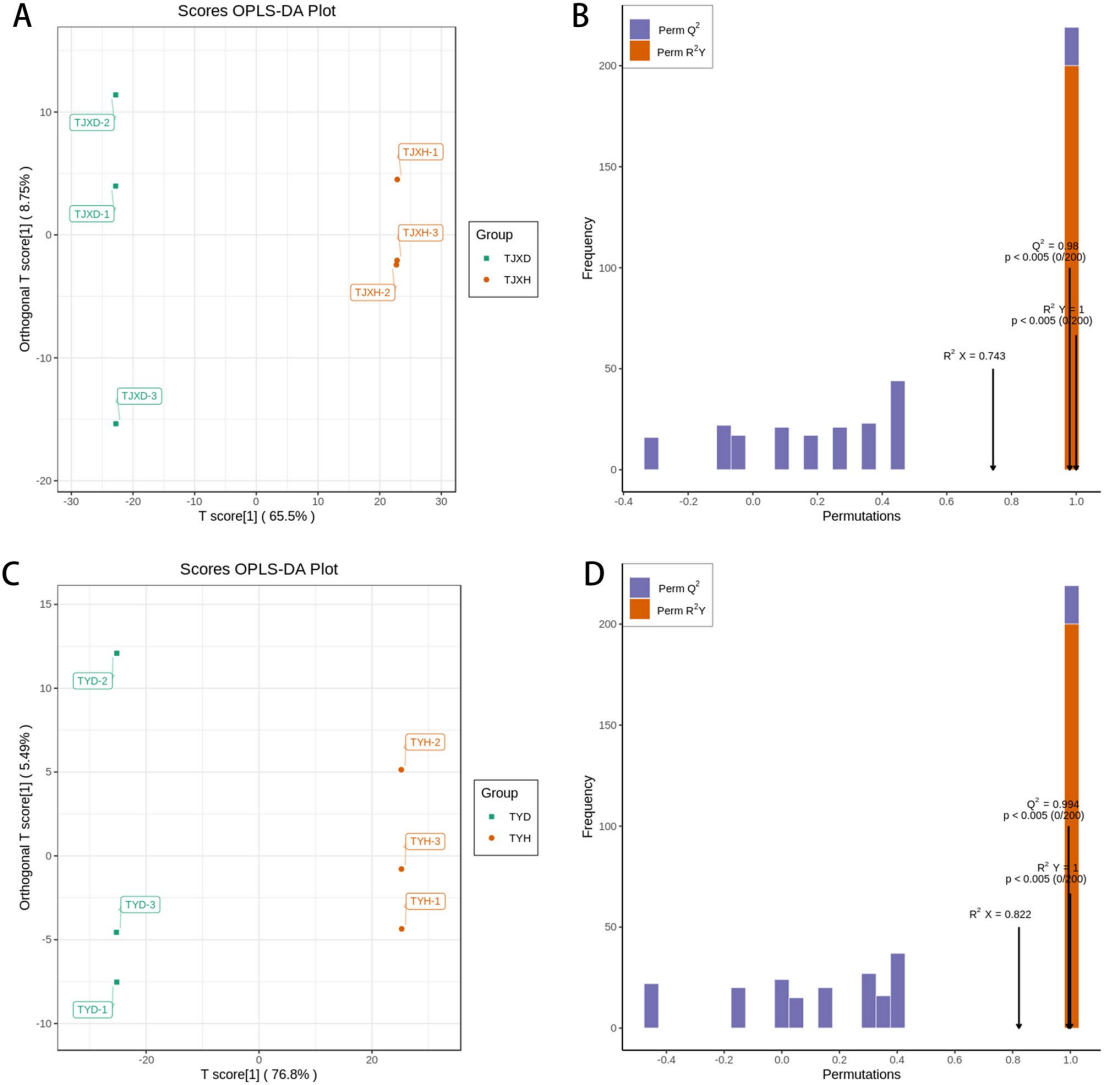
The score plots of OPLS-DA pairwise comparisons of differential metabolites: (**A**) TJXD vs TJXH, (**C**) TYD vs TYH; Model verification diagram: (**B**) TJXD vs TJXH, (**D**) TYD vs TYH.

OPLS-DA analysis is a multivariate statistical analysis with supervised pattern recognition, which can effectively eliminate irrelevant effects to screen differential metabolites. In the OPLS-DA score plot (Fig. 3C), the TYD samples were distributed on the left side of the confidence interval, while the TYH samples were distributed on the right. The core value of the principal component in the OSC process (T score [1]) was 76.8%. The orthogonal T score [1] in the OSC process was 5.49%. The prediction parameters of the evaluation model are R^2^X, R^2^Y and Q^2^. R^2^X and R^2^Y represent the interpretation rate of the X and Y matrices of the built model respectively, and Q^2^ represents the prediction ability of the model. The closer these three indicators are to 1, the more stable the model is. During model verification (n = 200, that is, 200 permutation experiments) of OPLS-DA (Fig. 3D), Q^2^ = 0.994 > 0.9, R^2^Y = 1, R^2^X = 0.822, P < 0.005. Similarly, in another OPLS-DA score plot (Fig. 3A), the TJXD and TJXH samples were clearly separated and during model verification (Fig. 3B), Q^2^ = 0.98 > 0.9, R^2^Y = 1, R^2^X = 0.743, P < 0.005 further indicated that two models were both reliable.

### Analysis of metabolites variations for *Angelica dahurica* derivative from two plantation bases under two drying methods

**Figure 4:**
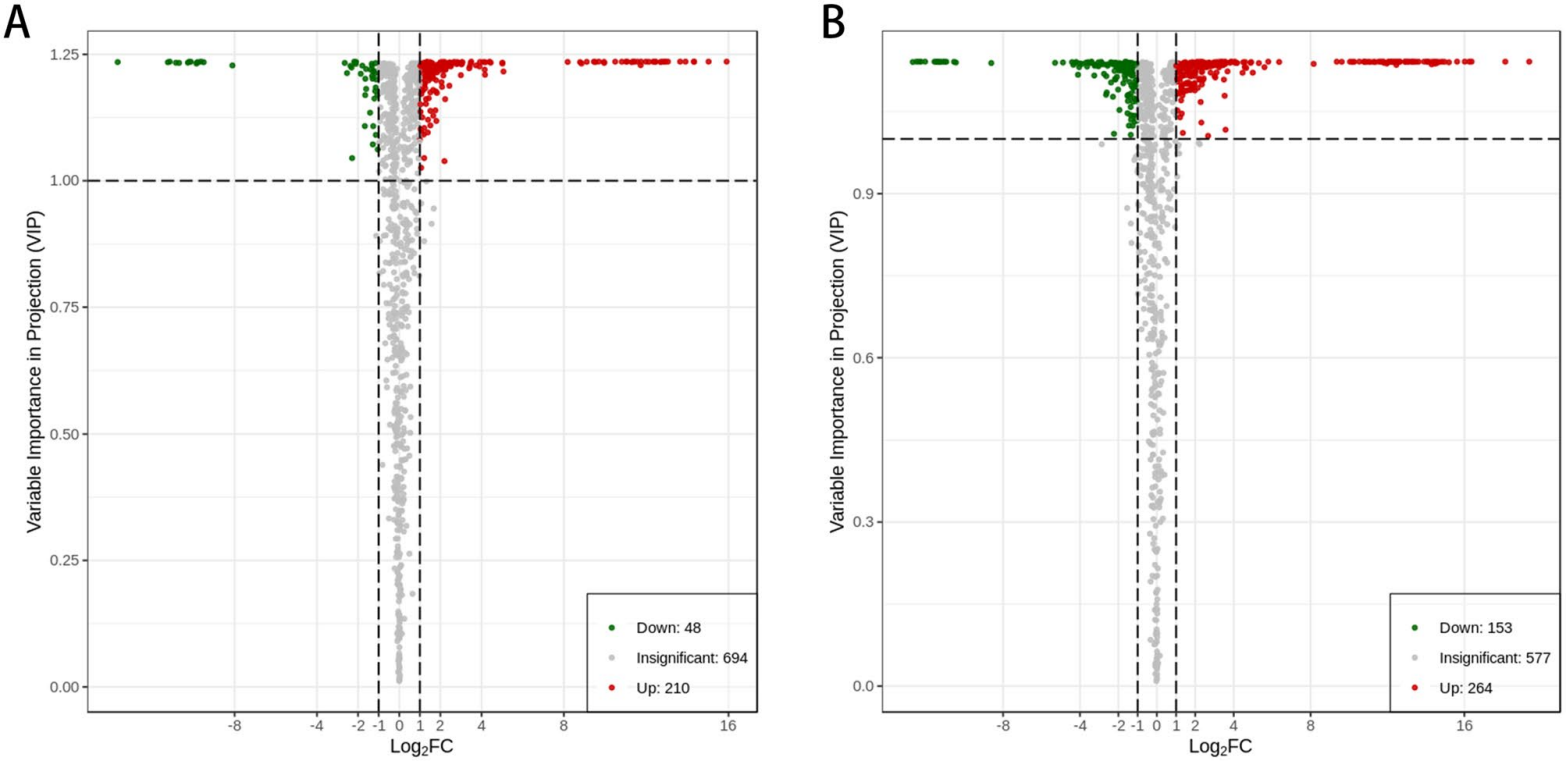
Volcano diagram of metabolites: (**A**) TJXD vs TJXH, (**B**) TYD vs TYH.

The differential metabolites of TYD vs. TYH and TJXD vs. TJXH were initially screened using OPLS-DA. The fold change value and VIP value were used comprehensively to screen the differential metabolites. Metabolites with a fold change ≥ 2, a fold change ≤ 0.5, and a VIP ≥ 1 were selected as significant differential metabolites. The volcano plot in Fig. 4A,B suggest a significant difference in differential metabolites, which further validated the reliability of the results. For TJXD vs. TJXH, 258 (210 upregulated and 48 downregulated) differential metabolites were obtained. For TYD vs. TYH, 417 (264 upregulated and 153 downregulated) were identified as differential metabolites. As shown in Supplementary Fig. S1, for both groups, more metabolites were upregulated under oven drying than freeze-drying, indicating that physiological and biochemical reactions were promoted by oven drying. As shown in Supplementary Table S1, the metabolites for TJXD vs. TJXH selected metabolites were classified into 10 major classes and 28 subclasses, including amino acids and derivatives (n = 27), phenolic acids (n = 45), nucleotides and derivatives (n = 32), flavonoids (n = 13), lignans and coumarins (n = 12), alkaloids (n = 24), Terpenoids (n = 4), organic acids (n = 24), lipids (n = 55) and others (n = 22). The metabolites for TYD vs. TYH selected were classified into 10 major categories and 36 subcategories, including amino acids and derivatives (n = 32), phenolic acids (n = 63), nucleotides and derivatives (n = 49), flavonoids (n = 51), quinones (n = 1), lignans and coumarins (n = 28), alkaloids (n = 44), terpenoids (n = 8), organic acids (n = 32), lipids (n = 55) and others (n = 34). Our results suggest more types of differential metabolites in TY than in TJX, especially alkaloids and flavonoids, which may be attributed to the different plantation bases.

### Identification of common differential metabolites of two plantation bases

The successful identification of common differential metabolites could assist in figuring out the physiological and biochemical reactions of *Angelica dahurica* during drying. We combined and filtered these data based on the two sets of differential metabolites mentioned previously. 193 common differential metabolites were obtained between both groups (Supplementary Table S2). Among these differential metabolites, 158 were upregulated, and 35 were downregulated, implying that some key physiological metabolites and metabolic activities might be activated during oven drying.

**Figure 5:**
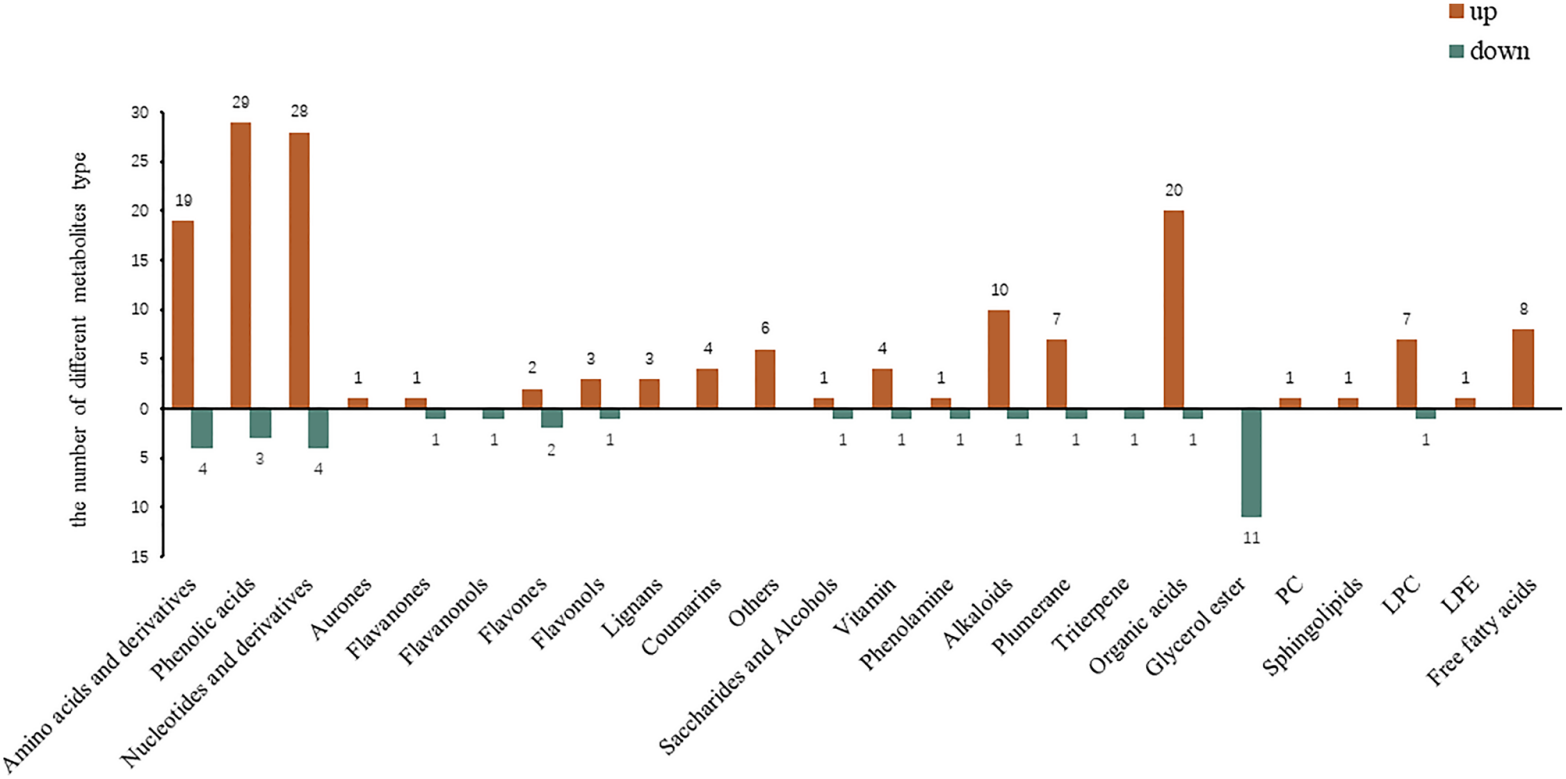
Classification of common differential metabolites between TYD VS TYH and TJXD VS TJXH groups.

These metabolites were classified into 10 major classes and 24 subclasses, including amino acids and derivatives (n = 25), nucleotides and derivatives (n = 32), phenolic acids (n = 32), flavonoids (n = 12), lignans and coumarins (n = 7), alkaloids (n = 21), organic acids (n = 21), lipids (n = 25), terpenoids (n = 1) and others (n = 13). Among these metabolites, amino acids and derivatives, nucleotides and derivatives, lipids and organic acids were the primary plant metabolites, while phenolic acids, flavonoids, lignans and coumarins, alkaloids and terpenoids were the secondary metabolites. As shown in Fig. 5, these differentially expressed metabolites were concentrated in amino and derivatives, phenolic acids, nucleotides and derivatives, and organic acids. Pairwise comparisons showed that the number of upregulated metabolites was significantly higher than downregulated metabolites. Moreover, the number of secondary metabolites was higher during oven drying than freezedrying, which indicated that the physiological and biochemical reactions of *Angelica dahurica* under heat were similar to *Angelica dahurica* under biological or abiotic stress. In addition, seven lignans and coumarins were all upregulated during oven drying, consistent with the literature.

### KEGG annotation and enrichment analysis of differential metabolites

**Figure 6:**
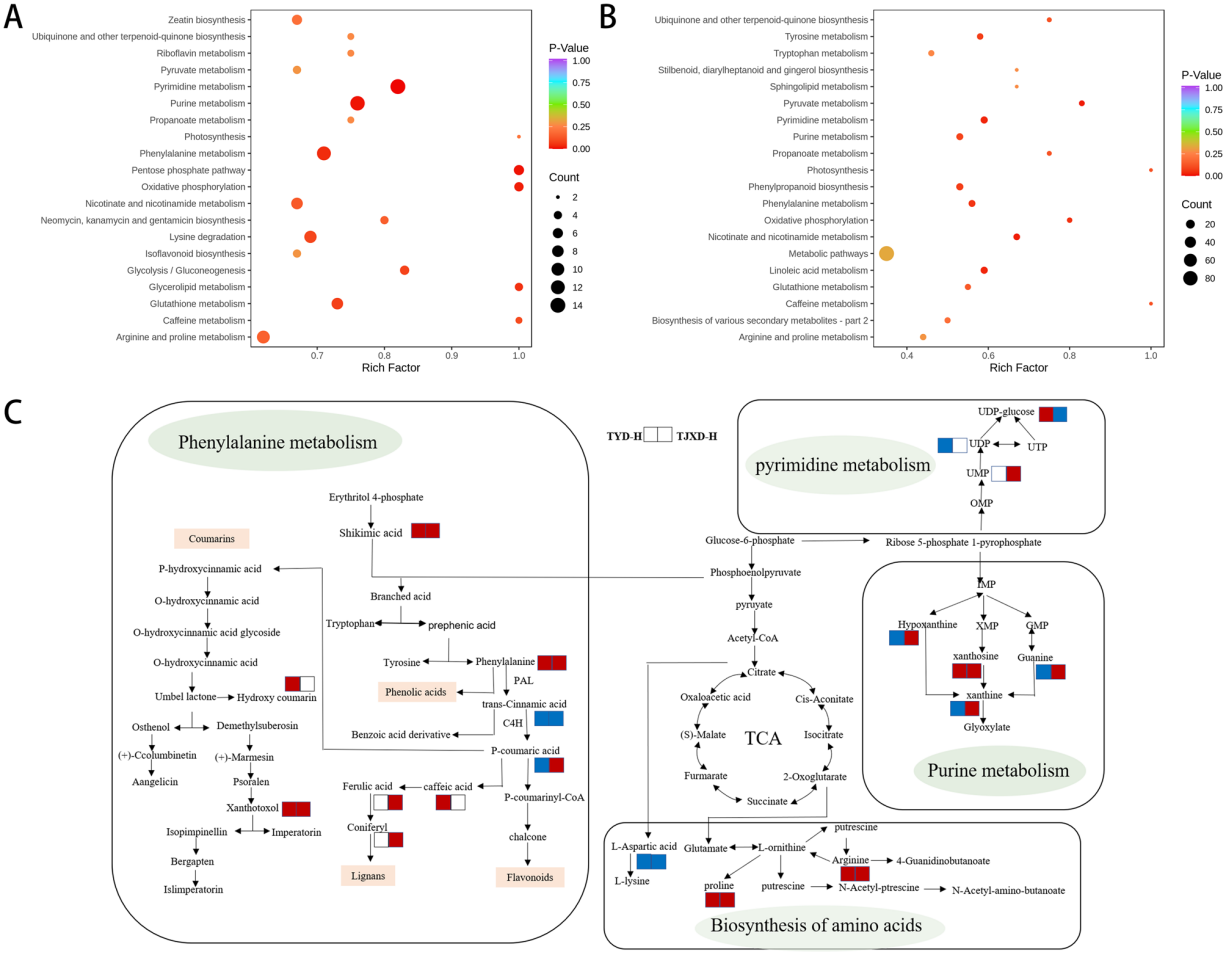
KEGG annotations and enrichment of differentially expressed metabolites: (**A**) TJXD vs TJXH, (**B**) TYD vs TYH; (**C**) Changes in key metabolites mapped to metabolic pathways in *Angelica dahurica* samples. Note: The red color small rectangle indicates that metabolite content is significantly upregulated; the blue small rectangle indicates that metabolite content is significantly downregulated; the white small rectangle indicates no significant difference in that metabolite content.

All differential metabolites in pairwise comparison groups were matched to the KEGG database (www.kegg.jp/kegg/kegg1.html.) to obtain the metabolic pathway information. KEGG annotation and enrichment analysis were conducted, and the enriched pathways are shown in Fig. 6A,B. For TJXD vs. TJXH, the enriched metabolic pathways of these differential metabolites consisted of tyrosine metabolism, tryptophan metabolism, pyrimidine metabolism, purine metabolism, phenylpropanoid biosynthesis, phenylalanine metabolism, and linoleic acid metabolism. For TYD vs. TYH, the enriched pathways of differential metabolites contained pyrimidine metabolism, purine metabolism, phenylalanine metabolism, lysine degradation and glutathione metabolism. The intersection yielded pyrimidine, purine, and phenylalanine metabolism as significantly enriched pathways for these differential metabolites. Purine and pyrimidine metabolism are involved in the synthesis of precursor materials that are essential for downstream synthesis. Specifically, purine and pyrimidine bases are produced during purine and pyrimidine metabolism and are basic materials for the synthesis of nucleotides. Nucleotides are essential cellular components that play an important role in plant growth, development, metabolism, and synthesis of other substances. Moreover, purine and pyrimidine metabolism is required for primary and secondary plant metabolism^13,14^. For instance, uridine diphosphate (UDP) (Compound CID: 6031) was produced during the pyrimidine metabolism (Fig. 6C). Under the catalysis of sucrose synthase, sucrose and UDP reacted reversibly to produce fructose and UDP glucose. UDP-glucose acted as a glucosyl donor in the derivatization of secondary metabolites and hormones in a wide range of reactions catalyzed by the enormous protein family of UDP-glucose glycosyltransferases. Xanthosine (Compound CID: 64959) was produced during the purine metabolism, participating in the synthesis of alkaloids such as theobromine, caffeine and so on (Supplementary table S4).

Phenylalanine metabolism is one of the most crucial pathways for secondary metabolite synthesis in plants. We found that phenolic acids, flavonoids, lignans and coumarins and some alkaloids were synthesized by this pathway (Fig. 6C). Phenylalanine (Compound CID: 6140) was synthesized by shikimic acid (Compound CID: 8742). Under the action of phenylalanine ammonia-lyase (PAL), phenylalanine is converted to trans-cinnamic acid (Compound CID: 139054223). Then trans-cinnamic acid was transferred to *P*-coumaric acid (Compound CID: 637542) under the action of cinnamate 4-hydroxylase (C4H) (Supplementary Table S4). Trans cinnamic-acid serves as the most important precursor substance of secondary metabolites. PAL and C4H are also considered two core enzymes.

## Discussion

At present, research on the composition of *Angelica dahurica* has mainly focused on coumarin and volatile oil. In this study, we used widely-targeted metabolomics^15^ which is well-established for its high sensitivity, accurate quantitative and qualitative properties, and wide coverage to identify metabolites of *Angelica dahurica* in two plantation bases following two distinct drying methods. Compared with other researches about the effects of different drying methods on the quality of *Angelica dahurica*, we used freeze drying as the control group and oven drying as the experimental group to reveal the differential metabolites produced under the two drying methods, aiming to explore the coumarin changes in *Angelica dahurica *with temperature from the perspective of metabolism. We screened 995 metabolites, of which 27 were in the top 50 in each of the four groups (Supplementary Table S3). Most secondary metabolites belonged to the coumarin class except for the primary metabolites, which maintain the organism's normal activities. Other classes of secondary metabolites were identified. For example, pterolactam (Compound CID: 181561), isolated from *Chrysanthemum coronarium* L. and rhizome of *Coniogramme japonica,* has been classified in pyrrole alkaloids and recent studies have shown that it has antimicrobial activity. Anca-Elena Dascalu et al. evaluated Pterolactam's antifungal activities on a panel of nine fungal strains and three non-albicans candida yeast species^16–18^. L-Pipecolic acid (Compound CID: 439,227) is an intermediate of L-Lysine catabolism, and its central injection is reported to exert a hypnotic effect on the brain^19^. Moreover, it plays an important role in medical issues, rhizosphere ecology, decontamination of polluted soils, nutrient acquisition and plant resistance^20–23^. At the same time, besides coumarin and volatile oil in *Angelica dahurica*, there were still some other secondary metabolites with high content, pharmacological effects and application value. This study provides a theoretical reference for future research on other substances in *Angelica dahurica* (Supplementary table S4).

It has been established that widely-targeted metabolome analysis enables the quantitative detection of approximately a thousand metabolites at a time, conducive to the comprehensive and effective comparison of metabolite differences and analysis of metabolic pathways^24^. Differential metabolite analysis yielded 193 differential metabolites classified into 10 major classes. Among these differential metabolites, 158 were upregulated, and 35 were downregulated. In lipids, fatty acids were upregulated, but glycerol ester consisting of linoleate and linolenate was downregulated. The increase in the saturation level of fatty acids has positive effects on maintaining membrane stability and heat tolerance, given that a greater proportion of saturated fatty acids might result in a higher lipid melting temperature and prevent a heat-induced increase in membrane fluidity^25^. Linoleate and linolenate are the major fatty acids in plant membranes^26^. Therefore, we speculate that with an increase in processing temperature, linoleate and linolenate content would be decreased after the cell membrane of *Angelica dahurica* was damaged, while an increase in fatty acid content could prevent increased membrane fluidity caused by heat. In addition, coumarin was upregulated, consistent with the literature. Based on the KEGG annotation and enrichment results, three metabolic pathways were mapped to these overlapping differential metabolites, namely pyrimidine metabolism, purine metabolism and phenylalanine metabolism. Given that purine and pyrimidine metabolism participate in the synthesis of upstream substances, we discussed the phenylalanine metabolism pathway next.

The biosynthesis pathway of phenylpropanoid, one of the major secondary metabolites in plants under abiotic or biotic stress, is reported to generate numerous antioxidants, including flavonoids, lignans, and phenols, to protect plants from being attacked^27,28^. UV-C irradiation has been found to increase phenylpropanoid pathway gene expression in sweet cherries (*Prunus avium* L.)^29^. In this study, phenylpropanoid biosynthesis was significantly enhanced with increased temperature. In phenolic acids, the first and second metabolites and their derivatives of phenylalanine were increased, such as cinnamic acid, p-coumaric acid, p-coumaraldehyde, p-coumaryl alcohol, hydrocinnamic acid, 2-hydroxycinnamic acid, and 4-methoxycinnamic acid which substantiated the activation of phenylalanine pathway and provided precursor substances for the increase of flavonoids, coumarin, lignin. It is possibly associated with the activation of PAL and C4H^30,31^. Besides, other accumulated phenolic acids, such as gallic acid (Compound CID: 370), reportedly have anticancer, anti-inflammatory, and hepatoprotective potential^32^. Flavonoids act as free radical scavengers, reducing agents, hydrogen donors and singlet oxygen quenchers, exhibiting high antioxidant properties^33^. Kaempferol-3-O-glucoside (Compound CID: 5282102) and catechin-5-O-glucoside (Compound CID: 44257081) are two kinds of flavonoids well-documented to have strong antioxidant activity^34^, were significantly increased and contributed to resisting oxidation during heating in the present study (Supplementary Table S4).

In our study, coumarin and lignin were all upregulated, and they are generated by phenylalanine metabolism. The phenylalanine metabolism pathway is an enzymatic reaction, and PAL is the central enzyme of phenylalanine metabolism. Under normal circumstances, PAL expression is low in plants and usually increases in response to biotic or abiotic stress like high temperature, mechanical damage, etc. Hence, it is reasonable to deduce that PAL is activated during the heat drying of *Angelica dahurica*, promoting phenylalanine metabolism and increasing the contents of coumarins and lignans^35^. Coumarin is well-recognized as the main pharmacological substance of *Angelica dahurica*. Significantly increased coumarins among the differential metabolites have certain pharmacological effects, such as scoparone (Compound CID: 8417) (Supplementary Table S4), previously established to reduce the proliferative responses of human peripheral mononuclear cells, relax smooth muscle, reduce total cholesterol and triglycerides and retard the characteristic pathomorphological changes in hypercholesterolaemic diabetic rabbits^36^. The psoralen derivative methoxsalen has a good curative effect on psoriasis and other dermatoses^37^. The observed upregulation of coumarins is consistent with the literature, and the analysis of upregulated coumarins enables the identification of the optimal drying approach for *Angelica dahurica*. Lignin occurs via the oxidative coupling of monolignols, synthesized by the phenylpropanoid pathway. It is an essential component of the secondary cell wall of plants, strengthening the cell structure. Accordingly, we infer that lignin accumulation is related to resisting high temperatures^38,39^.

In addition to the significant differences in the secondary metabolites generated by the phenylalanine pathway, 21 alkaloid components were significantly altered, of which 19 were upregulated. Alkaloids are formed from amino acids through a wide range of biochemical reactions. The changed alkaloids are mainly derived from tryptophan, phenylalanine, and ornithine metabolism, consistent with the differential metabolites of amino acid compounds. It can be seen that amino acid metabolism is a pathway for protein synthesis and acts as an intermediate for some metabolites, and participates in the regulation of various metabolic pathways, thereby affecting many physiological processes in plants. Furthermore, upregulated alkaloids such as betaine and norgalanthamine exhibit numerous pharmacological activities^40^.

This study still has some limitations. It is widely acknowledged that the plant metabolome is composed of over 200,000 metabolites that control plant development, and even *Arabidopsis* contains 5000 metabolites^41^. Accordingly, *Angelica dahurica* consists of more than 995 metabolites identified in the present study. This discrepancy may be attributed to the lack of large public herbal medicine metabolite databases. Moreover, another main active ingredient of *Angelica* dahurica is volatile oil; however, UPLC-MS/MS analysis has limited value for detecting volatile oil. In future research, the metabolome of *Angelica dahurica* should be analyzed with an emphasis on volatile oil. Further studies are warranted to verify whether PAL, 4-coumarate-CoA ligase (4CL), and C4H enzymes are key in the phenylpropanoid pathway activation. Overall, this study provides a theoretical reference for the research of other substances in *Angelica dahurica* and corroborates that coumarin is enhanced with increased temperature within a certain range during the processing, providing novel insights on the quality of *Angelica dahurica* for clinical application.

## Materials and methods

### Plant materials and treatment

The plant materials were harvested from Suining, Sichuan, China, from different planting fields in the region Tai Yi (TY) and Tang Jia Xiang (TJX) bases, identified as *Angelica dahurica* by Jin Pei, Prof. of Chengdu University of Traditional Chinese Medicine and kept in the national germplasm resource bank of traditional Chinese medicine. Then the experimental materials from the two fields were freeze-drying and oven drying at 60 °C, respectively. All collected samples were divided into four groups, which were from TY and oven drying (TYH), TXJ and oven drying (TJXH), TY and freeze-drying (TYD), and TJX and freeze-drying (TJXD). Samples were equally distributed to the four groups after harvesting and three repulications were performed for each experiment.

### Statement about the plant collected

Collection of *Angelica dahurica* in this study conforms to and complies with the IUCN Policy Statement on Research Involving Species at Risk of Extinction and the Convention on the Trade in Endangered Species of Wild Fauna and Flora. In addition, according to the List of National Key Protected Wild Plants issued by the State Forestry and Grassland Bureau of China, *Angelica dahurica*, the experimental material of this study, is neither an endangered plant species nor a national key protected wild plant. Besides, we have obtained permission for plant collection from Sichuan Suining Quantaitang Pharmaceutical Co., Ltd.

### Sample preparation and extraction

Related experiments such as sample preparation and extraction of metabolites were conducted by Wuhan Maitville Biotechnology Co., Ltd. Biological samples were freeze-dried by a vacuum freeze-dryer (Scientz-100F) and heat dried by an oven (DHG-9140A). The samples were crushed using a mixer mill (MM 400, Retsch) with a zirconia bead for 1.5 min at 30 Hz. 100 mg of powder was dissolved with 1.2 mL 70% methanol solution, vortexed 30 s every 30 min for 6 times in total. The samples were placed in a refrigerator at 4 °C overnight. Following centrifugation at 12,000 rpm for 10 min, the extracts were filtrated (SCAA-104, 0.22 μm pore size; ANPEL, Shanghai, China, (http://www.anpel.com.cn/) before UPLC-MS/MS analysis.

### Liquid chromatography-mass spectrometry

The sample extracts were analyzed using a UPLC-ESI–MS/MS system (UPLC, SHIMADZU Nexera X2,(https://www.shimadzu.com.cn/); MS, Applied Biosystems 4500 Q TRAP, (https://www.thermofisher.cn/cn/zh/home/brands/applied-biosystems.html). The analytical conditions were as follows, UPLC: column, Agilent SB-C18 (1.8 µm, 2.1 mm * 100 mm). The mobile phase consisted of solvent A, pure water with 0.1% formic acid, and solvent B, acetonitrile with 0.1% formic acid. The gradient program was as follows: 95:5 V/V at 0 min, 5:95 V/V at 11.0 min, 5:95 V/V at 12.0 min, 95:5 V/V at 12.1 min. Subsequently, the composition was adjusted to 95% A and 5.0% B within 1.1 min and kept for 2.9 min. The flow velocity was set as 0.35 mL per minute. The column oven was 40 °C, and the injection volume was 4 μL. The effluent was alternatively connected to an ESI-triple quadrupole-linear ion trap (QTRAP)-MS.

The ESI source operation parameters were as follows: an ion source, turbo spray; source temperature 550 °C; ion spray voltage (IS) 5500 V (positive ion mode)/-4500 V (negative ion mode); ion source gas I (GSI), gas II(GSII), curtain gas (CUR) was set at 50, 60, and 25.0 psi, respectively; the collision-activated dissociation (CAD) was high. QQQ scans were acquired as MRM experiments with collision gas (nitrogen) set to medium. DP and CE for individual MRM transitions were performed with further DP and CE optimization. A specific set of MRM transitions were monitored for each period according to the metabolites eluted within this period.

### Qualitative and quantitative determination of metabolites

Metabolite structure analysis was based on self-established databases HWDB. The primary and secondary spectra detected by mass spectrometry were analyzed qualitatively, and isotopic signals were removed during the analysis of some substances, including repeated signals of K + ions, Na + ions, NH 4 + ions, and fragment ions that were themselves other larger molecular weight substances repeating signal.

Metabolite quantification was carried out via the MRM mode of the QQQ mass spectrometer.

### Data analysis

A variety of statistical analysis methods were used to process the metabolic data, including principal component analysis (PCA), hierarchical cluster analysis (HCA) and orthogonal partial least squares-discriminant analysis (OPLS-DA). PCA was performed by the R function "prcomp" (www.r-project.org). The HCA results of samples and metabolites were presented as heatmaps with dendrograms and were carried out by R package Complex Heatmap. For HCA, normalized signal intensities of metabolites (unit variance scaling) were visualized as a color spectrum. VIP values were extracted from the OPLS-DA result, consisting of score plots and permutation plots, generated using soft R. Identified metabolites were annotated using the KEGG Compound database (http://www.kegg.jp/kegg/compound/). Annotated metabolites were then mapped to the KEGG database (http://www.kegg.jp/kegg/pathway.html).

## Conclusion

A total of 995 metabolites were detected in TYD, TYH, TJXD, and TJXH. Among them, besides coumarin and volatile oil in *Angelica dahurica*, there were still other secondary metabolites with high content, pharmacological effects and application value, which can be used for future research. Furthermore, 193 differential metabolites were identified as key differential metabolites, most of which were upregulated under oven drying. The KEEG annotation and enrichment analysis showed that moderate heating could promote the phenylalanine pathway, resulting in increased coumarin content and the established potential metabolite network revealed this phenomenon. At the same time, the activation of the purine and pyrimidine pathways upregulates most primary and secondary metabolites, which are of significant value.*. *Nevertheless, further studies are warranted to verify whether PAL, 4-coumarate-CoA ligase (4CL), and C4H enzymes are key in the phenylpropanoid pathway activation.

## Supplementary Information

Supplementary Information.

## Data Availability

The datasets used and/or analysed during the current study are available from the corresponding author on reasonable request.

## Abbreviations

4CL: 4-Coumarate-CoA ligase
C4H: Cinnamate 4-hydroxylase
CAD: Collision-activated dissociation
CUR: Curtain gas
GSI: Gas I
GSII: Gas II
HCA: Hierarchical cluster analysis
LC–MS/MS: Liquid chromatographytandem mass spectrometry
OPLS-DA: Orthogonal partial least squares-discriminant analysis
PAL: Phenylalanine ammonia lyase
PC1: Principal component 1
PC2: Principal component 2
PCA: Principal component analysis
QTRAP: Quadrupole-linear ion trap
UDP: Uridine diphosphate
