# Supplementary material for: Metabolomics analysis reveals metabolite changes during freeze-drying and oven-drying of Angelica dahurica
Source: Sci Rep. 2023 Apr 13;13:6022. doi: 10.1038/s41598-023-32402-0 (PMC10102171; doi:10.1038/s41598-023-32402-0)
Supplement: Supplementary file 1 — Supplementary Information. [file 41598_2023_32402_MOESM1_ESM.docx]

**Supplementary table S1**. Subgroup classification of differential metabolites between TYD VS TYH and TJXD VS TJXH

| Class II | TYD VS TYH up | TYD VS TYH down | TJXD VS TJXH up | TJXD VS TJXH down |
| --- | --- | --- | --- | --- |
| Amino acids and derivatives | 46 | 6 | 22 | 5 |
| Sesquiterpenoids | 2 | 2 | 1 | 0 |
| Chalcones | 0 | 3 | 1 | 0 |
| Aurones | 1 | 0 | 1 | 0 |
| Flavanones | 3 | 5 | 1 | 1 |
| Flavanonols | 0 | 3 | 0 | 1 |
| Phenolamine | 2 | 5 | 1 | 1 |
| Phenolic acids | 34 | 29 | 40 | 5 |
| Glycerol ester | 0 | 12 | 1 | 12 |
| Nucleotides and derivatives | 38 | 11 | 28 | 4 |
| Flavones | 11 | 4 | 2 | 2 |
| Flavonols | 11 | 4 | 3 | 1 |
| PC | 0 | 1 | 1 | 0 |
| Lignans | 4 | 5 | 3 | 0 |
| Others | 10 | 1 | 11 | 0 |
| Sphingolipids | 0 | 1 | 1 | 0 |
| LPC | 10 | 3 | 7 | 1 |
| LPE | 1 | 4 | 6 | 0 |
| Triterpene | 0 | 4 | 1 | 2 |
| Alkaloids | 15 | 5 | 11 | 2 |
| Saccharides and Alcohols | 9 | 9 | 2 | 3 |
| Vitamin | 4 | 1 | 5 | 1 |
| Coumarins | 9 | 10 | 8 | 1 |
| Plumerane | 10 | 2 | 7 | 1 |
| Free fatty acids | 10 | 13 | 24 | 2 |
| Organic acids | 27 | 5 | 21 | 3 |
| Pyridine alkaloids | 1 | 0 | 0 | 0 |
| Pyrrole alkaloids | 1 | 0 | 0 | 0 |
| Anthraquinone | 1 | 0 | 0 | 0 |
| Anthocyanidins | 1 | 1 | 0 | 0 |
| Quinorisidine alkaloids | 1 | 0 | 0 | 0 |
| Quinoline alkaloids | 0 | 1 | 0 | 0 |
| Tropan alkaloids | 1 | 0 | 0 | 0 |
| Triterpene Saponin | 0 | 2 | 0 | 0 |
| Isoflavones | 2 | 2 | 0 | 0 |
| Ditepenoids | 0 | 0 | 1 | 0 |

**Supplementary table S2.**193 common differential metabolites

| Class I | Compounds | VIP | Fold change | Type |
| --- | --- | --- | --- | --- |
| Amino acids and derivatives | 5-Oxo-L-Proline | 1.18788036 | 5.407003416 | up |
|  | N-Acetyl-L-threonine | 1.19439793 | 2.268436977 | up |
|  | Cyclo(L-Ala-L-Pro) | 1.2354882 | 4040.111111 | up |
|  | N-Acetyl-L-leucine | 1.21602841 | 33.44572871 | up |
|  | N-Acetyl-L-Glutamine | 1.23556805 | 12837.77778 | up |
|  | N-Phenylacetylglycine | 1.23523419 | 4480.333333 | up |
|  | Cyclo(Pro-Pro) | 1.21882873 | 3.816325148 | up |
|  | N-Acetyl-L-phenylalanine | 1.2355625 | 8511.851852 | up |
|  | Cyclo(Pro-Leu) | 1.22856992 | 3393.481481 | up |
|  | N-Acetyl-L-Arginine | 1.23434844 | 18.18682813 | up |
|  | N-Acetyl-L-tyrosine | 1.23030876 | 8.122470981 | up |
|  | Cyclo(Pro-Glu) | 1.23464326 | 734.8814815 | up |
|  | L-Lysine-Butanoic Acid | 1.10216857 | 2.048966874 | up |
|  | L-Alanyl-L-Phenylalanine | 1.19539073 | 2.107924707 | up |
|  | γ-L-Glutamyl-S-(trans-1-propenyl)-L-cysteine | 1.12062224 | 2.636820871 | up |
|  | γ-Glutamylphenylalanine | 1.22648409 | 2.182195378 | up |
|  | L-Glutamine-O-glycoside | 1.23250609 | 11.0680168 | up |
|  | L-Glutamic acid-O-glycoside | 1.20967284 | 17.96906278 | up |
|  | γ-Glutamyltyrosine | 1.22718245 | 2.015064098 | up |
|  | S-(Methyl)glutathione | 1.10427903 | 2.321033089 | up |
|  | L-Aspartic acid-O-diglucoside | 1.17176496 | 2.11218912 | up |
|  | L-Aspartic acid | 1.22098845 | 0.327905871 | down |
|  | L-Homomethionine | 1.23297515 | 0.159431462 | down |
|  | Glutathione reduced form | 1.23338145 | 0.000540595 | down |
|  | Oxiglutatione | 1.20166715 | 0.3190042 | down |
| Phenolic acids | 4-Hydroxybenzaldehyde | 1.23250452 | 4.094253395 | up |
|  | 4'-Hydroxyacetophenone | 1.12540079 | 2.175014556 | up |
|  | Phenyl acetate | 1.22372275 | 2.397755281 | up |
|  | 2,5-Dihydroxybenzaldehyde | 1.220121 | 2.605615792 | up |
|  | 4-Hydroxybenzoic acid | 1.22926066 | 2.69586815 | up |
|  | Protocatechualdehyde | 1.23441714 | 2.497033999 | up |
|  | p-Coumaraldehyde | 1.23556713 | 2653.592593 | up |
|  | Cinnamic acid | 1.23490136 | 15.16968039 | up |
|  | p-Coumaryl alcohol | 1.22592751 | 10.75241619 | up |
|  | Hydrocinnamic acid | 1.23450747 | 2518.814815 | up |
|  | p-Hydroxyphenyl acetic acid | 1.23554144 | 20461.85185 | up |
|  | Mandelic acid | 1.22730046 | 5.220315955 | up |
|  | 3-Aminosalicylic acid | 1.23515895 | 5543.444444 | up |
|  | 2,5-Dihydroxybenzoic acid; Gentisic Acid* | 1.23179293 | 32.26332722 | up |
|  | 3,4-Dihydroxybenzoic acid (Protocatechuic acid)* | 1.23348834 | 32.28202883 | up |
|  | p-Coumaric acid | 1.17381056 | 3.547965755 | up |
|  | 2-Hydroxycinnamic acid | 1.23073941 | 6.95116159 | up |
|  | 2-(Formylamino)benzoic acid | 1.23285959 | 5.883206029 | up |
|  | 3-(4-Hydroxyphenyl)-propionic acid | 1.20330717 | 2.16932147 | up |
|  | Gallic acid | 1.23556508 | 33464.81481 | up |
|  | 4-Methoxycinnamic acid | 1.22868479 | 3.619800816 | up |
|  | Methyl 2,4-dihydroxyphenylacetate | 1.23560005 | 7746.555556 | up |
|  | (S)-2-Hydroxy-3-(4-Hydroxyphenyl) Propanoic Acid | 1.23542752 | 5203.814815 | up |
|  | Syringaldehyde | 1.23532682 | 1801.259259 | up |
|  | Ethyl ferulate | 1.23154938 | 4.959362449 | up |
|  | 1-Feruloyl-sn-glycerol* | 1.12861097 | 3.147853581 | up |
|  | 2-Caffeoyl-L-tartaric acid (Caftaric acid) | 1.18280218 | 2.398822049 | up |
|  | Androsin | 1.23542452 | 6964.62963 | up |
|  | Vanillic Acid-4-O-Glucuronide | 1.22899378 | 2.381131048 | up |
|  | 2,3-Dihydroxybenzoic Acid* | 1.23467101 | 7.61842E-05 | down |
|  | Methyl Cinnamate | 1.18438683 | 0.452333135 | down |
|  | 1-O-Vanilloyl-D-Glucose | 1.23543654 | 0.000439704 | down |
| Nucleotides and derivatives | 1-Methylguanidine | 1.20884006 | 7.961155255 | up |
|  | 5-Methylcytosine | 1.23461288 | 441.6296296 | up |
|  | Thymine | 1.23558246 | 3435.222222 | up |
|  | 2-Aminopurine | 1.22055953 | 2.134697074 | up |
|  | Hypoxanthine | 1.22245709 | 10.56953703 | up |
|  | Guanine | 1.23458483 | 7.006760411 | up |
|  | Xanthine | 1.23559026 | 15392.96296 | up |
|  | 8-Azaguanine | 1.23328246 | 7.200517688 | up |
|  | 2-Deoxyribose-1-phosphate | 1.21080655 | 2.914371777 | up |
|  | 2-Deoxyribose-5'-phosphate | 1.19845669 | 2.692550843 | up |
|  | Thymidine | 1.22040397 | 3.091709166 | up |
|  | Cytarabine | 1.21041571 | 2.186735989 | up |
|  | Cytidine | 1.22459207 | 2.741308144 | up |
|  | β-Pseudouridine | 1.17800855 | 2.205159401 | up |
|  | 2'-Deoxyadenosine | 1.2342551 | 2.854976114 | up |
|  | Cordycepin (3'-Deoxyadenosine) | 1.23556567 | 2080.518519 | up |
|  | 5-Methyluridine | 1.13620439 | 2.048347311 | up |
|  | 2'-Deoxyguanosine | 1.23193259 | 3.012544003 | up |
|  | 9-(Arabinosyl)hypoxanthine | 1.23460134 | 2.559932888 | up |
|  | Guanosine | 1.23238218 | 2.579469609 | up |
|  | Xanthosine | 1.23426867 | 8.391516207 | up |
|  | 5-Aminoimidazole ribonucleotide | 1.22323704 | 2.385655902 | up |
|  | 2-(Dimethylamino)guanosine | 1.22398367 | 2.273144629 | up |
|  | N6-(2-Hydroxyethyl) adenosine | 1.22504663 | 2.389924132 | up |
|  | Cytidine 5'-monophosphate (Cytidylic acid) | 1.22379118 | 4.806504415 | up |
|  | Uridine 5'-monophosphate | 1.23419778 | 6.32161196 | up |
|  | Guanosine 3',5'-cyclic monophosphate | 1.23482958 | 17.06949583 | up |
|  | Succinyladenosine | 1.22901211 | 2.819863959 | up |
|  | Adenosine 5'-diphosphate | 1.2192783 | 0.385074478 | down |
|  | Uridine 5'-diphospho-D-glucose | 1.23546806 | 0.219369552 | down |
|  | Nicotinic acid adenine dinucleotide | 1.21156303 | 0.273519119 | down |
|  | NADP (Nicotinamide adenine dinucleotide phosphate) | 1.21286064 | 0.172228977 | down |
| Flavonoids | Aureusidin-4-O-glucoside | 1.17954428 | 3.127810075 | up |
|  | Naringenin-7-O-(6''-malonyl)glucoside | 1.15198515 | 2.46182225 | up |
|  | Diosmetin-7-O-Neohesperidoside (Neodiosmin) | 1.06198921 | 0.484595674 | down |
|  | Dihydrokaempferol-3-O-glucoside | 1.13453208 | 0.374360039 | down |
|  | 3-Hydroxy-3'-methoxyflavone | 1.23557078 | 7606.222222 | up |
|  | Luteolin-3'-O-glucoside | 1.2208658 | 2.892614309 | up |
|  | Chrysin | 1.20725338 | 0.40017284 | down |
|  | Acacetin | 1.22801342 | 0.003628642 | down |
|  | Kaempferol-3-O-glucoside (Astragalin) | 1.10953693 | 2.851955487 | up |
|  | Azalein (Azaleatin-3-O-rhamnoside) | 1.11830265 | 3.482435358 | up |
|  | Syringetin-3-O-glucoside | 1.09065669 | 0.45402541 | down |
|  | Catechin-5-O-glucoside | 1.23174586 | 4.510145043 | up |
| Lignans and Coumarins | 3,4-Methylenedioxy cinnamyl alcohol | 1.22936497 | 3.658224081 | up |
|  | Epipinoresinol* | 1.21854012 | 4.371515939 | up |
|  | Pinoresinol* | 1.21959468 | 4.7522723 | up |
|  | 3,4-Dihydrocoumarin | 1.23513834 | 987.9296296 | up |
|  | Xanthotoxol | 1.23052628 | 2.170886739 | up |
|  | Scoparone | 1.22602872 | 5.663158806 | up |
|  | Ethyl 3-coumarincarboxylate | 1.22452213 | 3.501640523 | up |
| Others | 2-Benzoxazolinone | 1.2355052 | 5537.148148 | up |
|  | 2-(Dodecylamino)-3-phenyl-1-propanol | 1.23548745 | 2719.407407 | up |
|  | N-benzoyl-2-aminoethyl-β-D-glucopyranoside | 1.23461052 | 8.227453838 | up |
|  | 30-O-I-Butyrylhamaudol | 1.2349851 | 290.737037 | up |
|  | Z-Ligustilide dimer E-232 | 1.09550295 | 2.612023493 | up |
|  | Z,Z9-6.89,7.39-Diligustilide | 1.23559644 | 827.1518519 | up |
|  | Nystose | 1.13917725 | 3.375068433 | up |
|  | Dihydroxyacetone phosphate | 1.04482833 | 0.204380971 | down |
|  | Isonicotinic acid | 1.23376422 | 3.987655984 | up |
|  | Nicotinic acid (Vitamin B3) | 1.23448395 | 4.082904669 | up |
|  | Pyridoxine | 1.23326492 | 5.436355964 | up |
|  | Riboflavin (Vitamin B2) | 1.23390973 | 2.847011183 | up |
|  | Dehydroascorbic acid | 1.23305552 | 0.000601355 | down |
| Alkaloids | Vanillylamine | 1.23545561 | 1442.703704 | up |
|  | N-Feruloylputrescine | 1.23431414 | 0.000413034 | down |
|  | Cadaverine | 1.23402506 | 7.102975107 | up |
|  | Betaine | 1.23556064 | 11.28415957 | up |
|  | N-Acetylputrescine | 1.16324838 | 2.699538785 | up |
|  | Agmatine | 1.2142392 | 3.562857651 | up |
|  | O-Phosphorylethanolamine | 1.19385672 | 2.025410727 | up |
|  | N-Acetylcadaverine | 1.22620456 | 4.342737722 | up |
|  | DL-2-Aminoadipic acid | 1.18982936 | 2.349981934 | up |
|  | Norgalanthamine | 1.23340919 | 21.73376781 | up |
|  | N-Isopentenyl-6-hydroxydendroxinium | 1.2329881 | 995.6925926 | up |
|  | (E)-N5-(1-((Carboxymethyl)amino)-3-((3-(4-hydroxy-3-methoxyphenyl)allylthio)-1-oxopropan-2-yl)glutamine | 1.22626189 | 4.643953951 | up |
|  | Dendrocrepine | 1.23502663 | 0.233578791 | down |
|  | Tryptamine | 1.2045392 | 2.639218644 | up |
|  | Indole-3-carboxylic acid* | 1.21395584 | 2.835439817 | up |
|  | Indole-5-carboxylic acid* | 1.20813345 | 3.100769103 | up |
|  | Tryptophol | 1.23559869 | 2154.555556 | up |
|  | Indole 3-acetic acid (IAA) | 1.23146173 | 8.234692986 | up |
|  | N-Acetylisatin | 1.02567554 | 2.092747283 | up |
|  | 1-Acetyl-β-carboline | 1.23108915 | 2.36779189 | up |
|  | N-Feruloylserotonin | 1.23527118 | 0.000895671 | down |
| Terpenoids | α-Amyrenone | 1.2352471 | 0.000842928 | down |
| Organic acids | Pyrrole-2-carboxylic acid | 1.23304292 | 7.793966363 | up |
|  | Fumaric acid | 1.22680035 | 3.63902439 | up |
|  | 5-Aminovaleric acid | 1.19884505 | 3.45272174 | up |
|  | Succinic acid | 1.2196647 | 2.340848134 | up |
|  | Methylmalonic acid | 1.2329644 | 2.432870934 | up |
|  | Acetoxyacetic acid | 1.23557481 | 61420.74074 | up |
|  | Aminomalonic acid | 1.23374824 | 2.349879291 | up |
|  | 2-Picolinic acid | 1.23413056 | 5.462255542 | up |
|  | 3-Guanidinopropionic acid | 1.23214418 | 3.057060181 | up |
|  | Glutaric acid | 1.16138803 | 4.694072877 | up |
|  | 1-Methylpiperidine-2-carboxylic acid | 1.13830021 | 2.854130234 | up |
|  | 4-Acetamidobutyric acid | 1.23546824 | 20.97091454 | up |
|  | 4-Guanidinobutyric acid | 1.23482167 | 6.389536963 | up |
|  | β-Ureidoisobutyric acid | 1.23542946 | 3035.740741 | up |
|  | Mevalonic acid | 1.23532399 | 20511.11111 | up |
|  | Benzoylformic acid | 1.23473752 | 6596.62963 | up |
|  | 2-Propylmalic Acid* | 1.23474371 | 4.323318237 | up |
|  | 2-Isopropylmalic Acid | 1.23251426 | 4.134175927 | up |
|  | 3-Isopropylmalic Acid* | 1.23373156 | 4.196837955 | up |
|  | 2-Hydroxyhexadecanoic acid | 1.23221029 | 4.494383213 | up |
|  | Iminodiacetic acid | 1.22755705 | 0.28847808 | down |
| Lipids | 1-Oleoyl-Sn-Glycerol | 1.23282266 | 0.451508532 | down |
|  | 2-α-Linolenoyl-glycerol-1-O-glucoside* | 1.23440788 | 0.001142775 | down |
|  | 1-α-Linolenoyl-glycerol-3-O-glucoside* | 1.23557894 | 0.001258853 | down |
|  | 2-Linoleoylglycerol-1-O-glucoside* | 1.23111269 | 0.230908191 | down |
|  | 1-Linoleoylglycerol-3-O-glucoside* | 1.22385639 | 0.200593593 | down |
|  | Gingerglycolipid A | 1.22731618 | 0.4 | down |
|  | 2-α-Linolenoyl-glycerol-1,3-di-O-glucoside* | 1.16939895 | 0.317805114 | down |
|  | 1-α-Linolenoyl-glycerol-2,3-di-O-glucoside* | 1.18159387 | 0.328056391 | down |
|  | Gingerglycolipid B | 1.21221699 | 0.441562432 | down |
|  | 2-Linoleoylglycerol-1,3-di-O-glucoside* | 1.10808406 | 0.313696886 | down |
|  | Gingerglycolipid C | 1.2003473 | 0.459826679 | down |
|  | Choline Alfoscerate | 1.2314851 | 2.267043327 | up |
|  | 3-Dehydrosphinganine | 1.20813976 | 4.415610568 | up |
|  | LysoPC 10:0 | 1.23187342 | 462.8740741 | up |
|  | LysoPC 12:0 | 1.22976159 | 5.104736022 | up |
|  | LysoPC 15:0(2n isomer) | 1.20615254 | 2.514055663 | up |
|  | LysoPC 16:0(2n isomer) | 1.23002092 | 2.864821791 | up |
|  | LysoPC 17:0(2n isomer) | 1.22068499 | 2.903391437 | up |
|  | LysoPC 18:0(2n isomer) | 1.20253176 | 2.835307026 | up |
|  | LysoPC 20:4 | 1.2344382 | 0.001378803 | down |
|  | LysoPE 15:0(2n isomer) | 1.22063031 | 4.473774405 | up |
|  | 16-Hydroxyhexadecanoic acid | 1.23553659 | 703.3851852 | up |
|  | DL-2-hydroxystearic acid | 1.22819311 | 3.075711778 | up |
|  | 2R-Hydroxyoctadecanoic Acid | 1.21268443 | 2.99508427 | up |
|  | 7S,8S-DiHODE | 1.22875925 | 5.742177853 | up |
|  | 9-Hydroxy-12-oxo-15(Z)-octadecenoic acid | 1.23156071 | 4.169871599 | up |
|  | 12,13-DHOME; (9Z)-12,13-Dihydroxyoctadec-9-enoic acid | 1.23494857 | 4.613116018 | up |
|  | Hydroxy ricinoleic acid | 1.19642489 | 2.024350738 | up |
|  | 15-Hydroperoxyicosatetraenoic acid | 1.23512247 | 552.7259259 | up |

**Supplementary table S3.** 27 Common metabolites among the top 50 metabolites in each group

| Formula | Compounds | Class I | Class II |
| --- | --- | --- | --- |
| C_16_H_22_O_4_ | Butyl isobutyl phthalate | Phenolic acids | Phenolic acids |
| C_17_H_16_O_6_ | Byakangelicol | Others | Others |
| C_5_H_11_N_3_O | 4-Guanidinobutanal | Others | Others |
| C_12_H_22_O_11_ | Galactinol | Others | Saccharides and Alcohols |
| C_6_H_11_NO_2_ | L-Pipecolic Acid | Organic acids | Organic acids |
| C_6_H_8_O_7_ | Citric Acid | Organic acids | Organic acids |
| C_4_H_6_O_5_ | L-Malic acid | Organic acids | Organic acids |
| C_10_H_13_N_5_O_5_ | Guanosine | Nucleotides and derivatives | Nucleotides and derivatives |
| C_6_H_6_N_4_S | 6-Methylmercaptopurine | Nucleotides and derivatives | Nucleotides and derivatives |
| C_26_H_50_NO_7_P | LysoPC 18:2 | Lipids | LPC |
| C_24_H_50_NO_7_P | LysoPC 16:0 | Lipids | LPC |
| C_18_H_36_O_2_ | Stearic Acid | Lipids | Free fatty acids |
| C_18_H_32_O_2_ | Linoleic acid | Lipids | Free fatty acids |
| C_18_H_34_O_2_ | Petroselinic acid | Lipids | Free fatty acids |
| C_21_H_22_O_4_ | 8-Geranyloxypsoralen | Lignans and Coumarins | Coumarins |
| C_13_H_10_O_5_ | Isopimpinellin | Lignans and Coumarins | Coumarins |
| C_20_H_24_O_10_ | Rutarin | Lignans and Coumarins | Coumarins |
| C_16_H_14_O_5_ | Oxypeucedanin | Lignans and Coumarins | Coumarins |
| C_16_H_14_O_5_ | Isooxypeucedanine | Lignans and Coumarins | Coumarins |
| C_16_H_14_O_4_ | Imperatorin | Lignans and Coumarins | Coumarins |
| C_16_H_16_O_10_ | Scopoletin-7-O-glucuronide | Lignans and Coumarins | Coumarins |
| C_11_H_12_N_2_O_2_ | L-Tryptophan | Amino acids and derivatives | Amino acids and derivatives |
| C_7_H_14_N_2_O_3_ | N-α-Acetyl-L-ornithine | Amino acids and derivatives | Amino acids and derivatives |
| C_6_H_14_N_2_O_2_ | L-Lysine | Amino acids and derivatives | Amino acids and derivatives |
| C_5_H_9_NO_2_ | Pterolactam | Alkaloids | Pyrrole alkaloids |
| C_11_H_9_O_2_N | 3-amino-2-naphthoic acid | Alkaloids | Alkaloids |
| C_11_H_9_NO_2_ | 3-Indoleacrylic acid | Alkaloids | Plumerane |


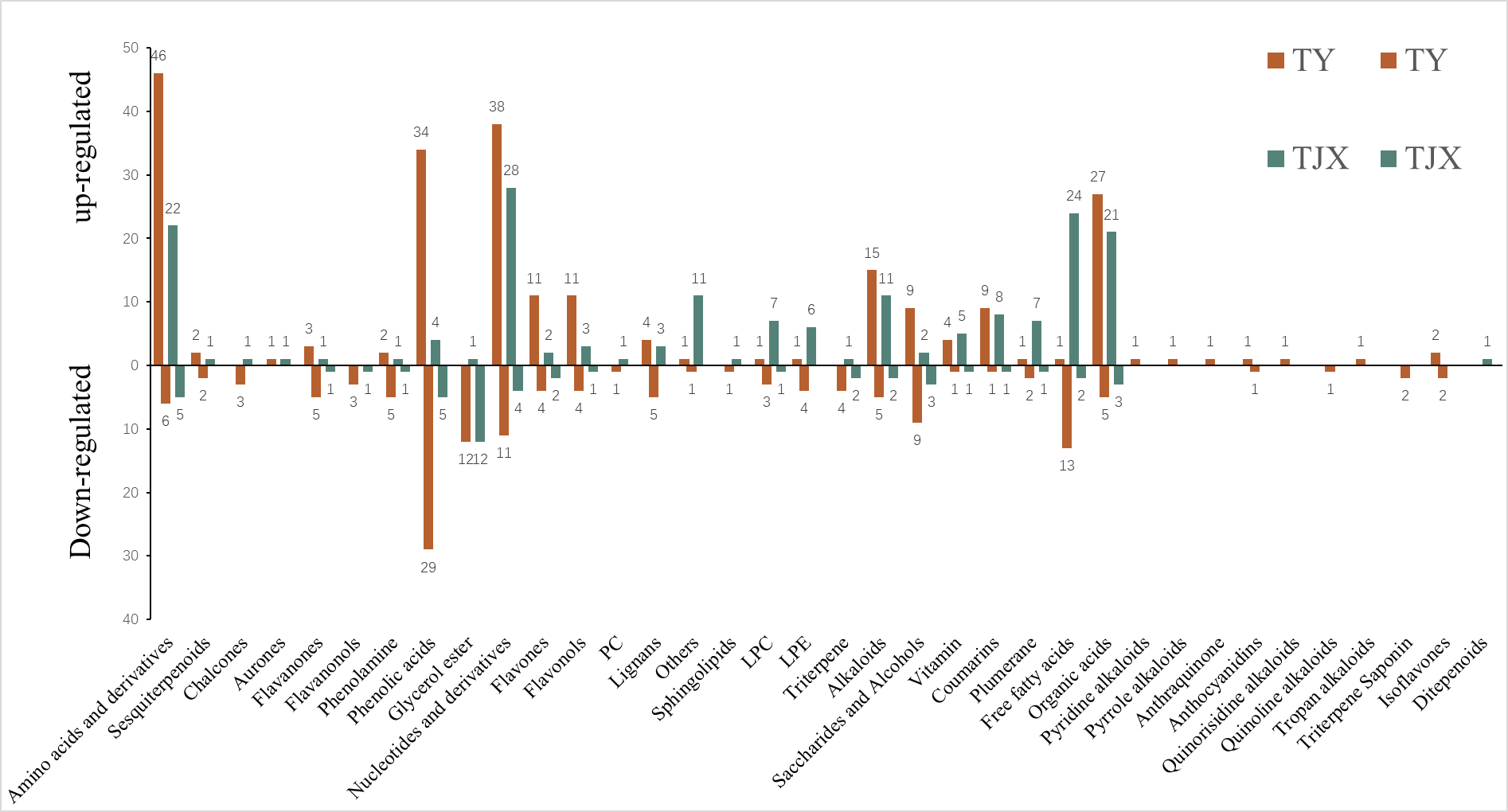


**Supplementary fig. S1** Subgroup classification of differential metabolites between TYD VS TYH and TJXD VS TJXH

**Supplementary table S4** The list of structural formula of compounds

| Number | Structural formula | Compound | Compound CID |
| --- | --- | --- | --- |
| 1 |  | Imperatorin | 10212 |
| 2 |  | Isoimperatorin | 68081 |
| 3 |  | Uridine diphosphate | 6031 |
| 4 |  | Xanthosine | 64959 |
| 5 |  | Phenylalanine | 6140 |
| 6 |  | Shikimic acid | 8742 |
| 7 |  | Trans cinnamic-acid | 139054223 |
| 8 |  | P-coumaric acid | 637542 |
| 9 |  | Pterolactam | 181561 |
| 10 |  | L-Pipecolic acid | 439227 |
| 11 |  | Gallic acid | 370 |
| 12 |  | Kaempferol-3-O-glucoside | 5282102 |
| 13 |  | Catechin-5-O-glucoside | 44257081 |
| 14 |  | Scoparone | 8417 |

**
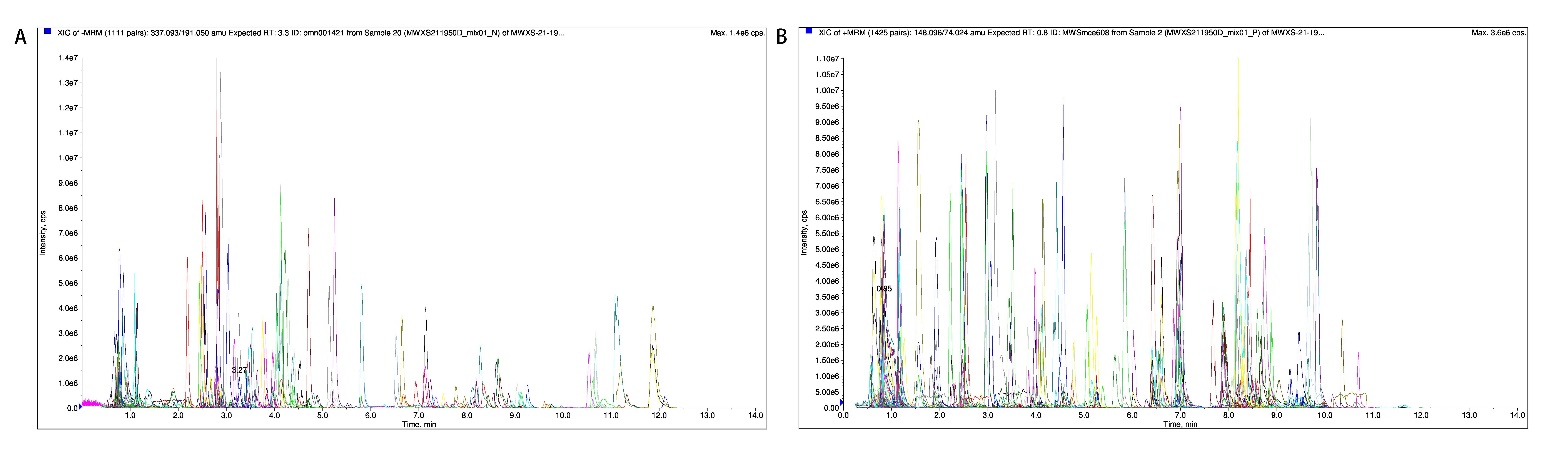
**

**Supplementary fig. S2** MRM detection of multimodal maps.

(A) Negative ion mode; (B) Positive ion mode.


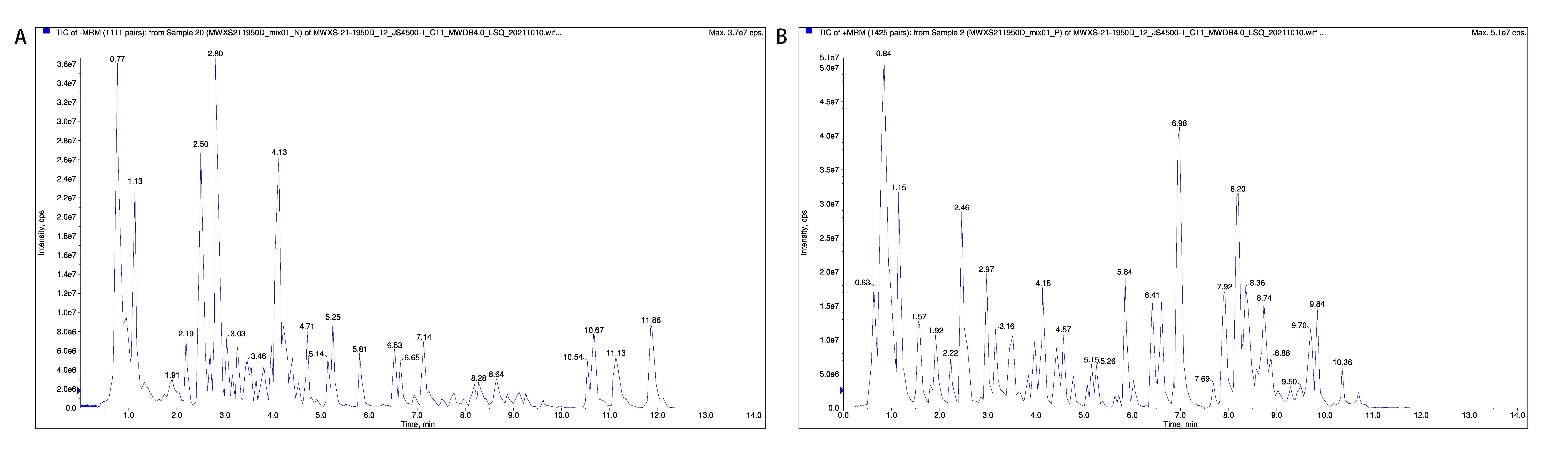


**Supplementary fig. S3** QC sample mass spectrometry detection TIC overlay.

1. Negative ion mode; (B) Positive ion mode.

**Supplementary table S5** Metabolites detected in all samples

| Index | Q1 (Da) | Q3 (Da) | Molecular Weight (Da) | Fomula | Ionization model | Compounds | Class I | Class II | CAS | Level | TYH-1 | TYH-2 | TYH-3 | TYD-1 | TYD-2 | TYD-3 | TJXH-1 | TJXH-2 | TJXH-3 | TJXD-1 | TJXD-2 | TJXD-3 | mix01 | mix02 | mix03 | cpd_ID | kegg_map |
| --- | --- | --- | --- | --- | --- | --- | --- | --- | --- | --- | --- | --- | --- | --- | --- | --- | --- | --- | --- | --- | --- | --- | --- | --- | --- | --- | --- |
| pme3200 | 7.40E+01 | 5.70E+01 | 7.31E+01 | C_2_H_7_N_3_ | [M+H]+ | 1-Methylguanidine | Nucleotides and derivatives | Nucleotides and derivatives | 471-29-4 | 3 | 3.23E+04 | 2.63E+04 | 2.04E+04 | 3.04E+03 | 2.35E+03 | 4.47E+03 | 3.65E+04 | 3.36E+04 | 3.26E+04 | 3.80E+03 | 2.89E+03 | 6.19E+03 | 9.29E+03 | 1.87E+04 | 1.74E+04 | C02294 | -- |
| Zmgn000765 | 7.30E+01 | 5.50E+01 | 7.40E+01 | C_3_H_6_O_2_ | [M-H]- | Propionic Acid | Organic acids | Organic acids | 79-09-4 | 3 | 1.73E+03 | 1.92E+03 | 2.27E+03 | 2.74E+03 | 1.28E+03 | 2.26E+03 | 3.10E+03 | 2.26E+03 | 1.56E+03 | 2.86E+03 | 2.25E+03 | 1.82E+03 | 1.72E+03 | 1.99E+03 | 3.55E+03 | C00163 | ko00640,ko00760,ko01100 |
| pmb0782 | 8.61E+01 | 6.92E+01 | 8.51E+01 | C_5_H_11_N | [M+H]+ | Piperidine | Alkaloids | Piperidine alkaloids | 110-89-4 | 3 | 2.52E+06 | 2.42E+06 | 2.63E+06 | 2.09E+06 | 2.14E+06 | 2.13E+06 | 2.38E+06 | 2.06E+06 | 2.47E+06 | 2.81E+06 | 2.75E+06 | 2.71E+06 | 2.49E+06 | 2.35E+06 | 2.85E+06 | C01746 | ko00960 |
| pme2292 | 8.91E+01 | 7.21E+01 | 8.81E+01 | C_4_H_12_N_2_ | [M+H]+ | Putrescine | Alkaloids | Alkaloids | 110-60-1 | 3 | 1.79E+05 | 2.08E+05 | 1.99E+05 | 4.85E+05 | 5.18E+05 | 4.68E+05 | 2.96E+05 | 3.22E+05 | 3.09E+05 | 1.82E+05 | 2.15E+05 | 1.95E+05 | 2.47E+05 | 3.15E+05 | 3.09E+05 | C00134 | ko00330,ko00472,ko00480,ko00960,ko01100,ko01110,ko02010 |
| ML10198895 | 8.80E+01 | 8.80E+01 | 8.90E+01 | C_3_H_7_NO_2_ | [M-H]- | N-Methylglycine | Amino acids and derivatives | Amino acids and derivatives | 107-97-1 | 3 | 1.02E+04 | 1.65E+04 | 1.03E+04 | 7.85E+03 | 1.08E+04 | 1.05E+04 | 1.35E+04 | 1.18E+04 | 1.16E+04 | 1.53E+04 | 1.68E+04 | 1.90E+04 | 5.93E+03 | 1.06E+04 | 8.68E+03 | C00213 | ko00260,ko00330,ko01100 |
| Lmgn002679 | 9.30E+01 | 7.50E+01 | 9.40E+01 | C_6_H_6_O | [M-H]- | Phenol | Phenolic acids | Phenolic acids | 108-95-2 | 3 | 2.99E+04 | 4.47E+04 | 4.27E+04 | 5.40E+04 | 3.59E+04 | 4.46E+04 | 9.00E+00 | 9.00E+00 | 9.00E+00 | 9.00E+00 | 9.00E+00 | 9.00E+00 | 1.95E+04 | 2.04E+04 | 3.08E+04 | C00146 | ko00350,ko01100 |
| Lmqp000873 | 1.01E+02 | 5.50E+01 | 1.00E+02 | C_4_H_4_O_3_ | [M+H]+ | Succinic anhydride | Organic acids | Organic acids | 108-30-5 | 3 | 3.42E+05 | 3.81E+05 | 3.61E+05 | 1.95E+05 | 1.86E+05 | 1.97E+05 | 3.38E+05 | 3.55E+05 | 3.86E+05 | 1.86E+05 | 1.81E+05 | 2.12E+05 | 2.96E+05 | 2.08E+05 | 3.48E+05 | C19524 | -- |
| mws4052 | 1.02E+02 | 5.61E+01 | 1.01E+02 | C_4_H_7_NO_2_ | [M+H]+ | 1-Aminocyclopropane-1-carboxylic acid | Organic acids | Organic acids | 22059-21-8 | 2 | 1.00E+05 | 9.89E+04 | 1.24E+05 | 9.39E+04 | 1.24E+05 | 9.94E+04 | 8.38E+04 | 9.22E+04 | 8.28E+04 | 1.10E+05 | 1.25E+05 | 9.49E+04 | 1.14E+05 | 1.13E+05 | 9.88E+04 | C01234 | ko00270,ko01100,ko01110 |
| MWSmce461 | 1.02E+02 | 5.61E+01 | 1.01E+02 | C_4_H_7_NO_2_ | [M+H]+ | L-Azetidine-2-carboxylic acid | Amino acids and derivatives | Amino acids and derivatives | 2133-34-8 | 2 | 1.05E+05 | 1.18E+05 | 1.05E+05 | 1.12E+05 | 1.22E+05 | 1.16E+05 | 1.32E+05 | 1.21E+05 | 1.15E+05 | 1.63E+05 | 1.32E+05 | 1.22E+05 | 1.40E+05 | 1.10E+05 | 1.14E+05 | -- | -- |
| MWS1840 | 1.02E+02 | 5.81E+01 | 1.01E+02 | C_6_H_15_N | [M+H]+ | Triethylamine | Alkaloids | Alkaloids | 121-44-8 | 2 | 1.20E+06 | 1.14E+06 | 1.19E+06 | 1.83E+06 | 1.85E+06 | 1.84E+06 | 1.58E+06 | 1.70E+06 | 1.72E+06 | 2.02E+06 | 1.91E+06 | 1.97E+06 | 1.69E+06 | 1.54E+06 | 1.73E+06 | C14691 | -- |
| pme1841 | 1.03E+02 | 8.61E+01 | 1.02E+02 | C_5_H_14_N_2_ | [M+H]+ | Cadaverine | Alkaloids | Alkaloids | 462-94-2 | 3 | 8.67E+05 | 7.72E+05 | 8.51E+05 | 7.43E+04 | 8.27E+04 | 7.48E+04 | 7.17E+05 | 6.80E+05 | 7.03E+05 | 9.03E+04 | 1.07E+05 | 9.88E+04 | 3.88E+05 | 4.03E+05 | 4.17E+05 | C01672 | ko00310,ko00480,ko00960,ko01100,ko01110 |
| MWSmce585 | 1.04E+02 | 5.90E+01 | 1.03E+02 | C_4_H_9_NO_2_ | [M+H]+ | Methyl 3-aminopropanoate | Amino acids and derivatives | Amino acids and derivatives | 4138-35-6 | 2 | 1.86E+06 | 1.92E+06 | 1.92E+06 | 2.13E+06 | 2.20E+06 | 2.08E+06 | 1.89E+06 | 2.01E+06 | 2.05E+06 | 2.29E+06 | 2.15E+06 | 2.26E+06 | 1.97E+06 | 2.12E+06 | 2.17E+06 | -- | -- |
| pme3011 | 1.04E+02 | 6.88E+01 | 1.03E+02 | C_4_H_9_NO_2_ | [M+H]+ | γ-Aminobutyric acid | Organic acids | Organic acids | 56-12-2 | 2 | 1.16E+07 | 1.17E+07 | 1.15E+07 | 6.92E+06 | 7.24E+06 | 6.98E+06 | 1.62E+07 | 1.61E+07 | 1.53E+07 | 1.15E+07 | 1.19E+07 | 1.19E+07 | 1.06E+07 | 1.12E+07 | 1.21E+07 | C00334 | ko00250,ko00330,ko00410,ko00650,ko00760,ko01100 |
| pme3033 | 1.04E+02 | 8.60E+01 | 1.03E+02 | C_4_H_9_NO_2_ | [M+H]+ | N,N-Dimethylglycine | Amino acids and derivatives | Amino acids and derivatives | 1118-68-9 | 3 | 5.56E+06 | 6.00E+06 | 6.32E+06 | 3.23E+06 | 3.61E+06 | 3.25E+06 | 7.79E+06 | 8.64E+06 | 7.39E+06 | 5.44E+06 | 5.91E+06 | 5.65E+06 | 4.92E+06 | 5.72E+06 | 5.56E+06 | C01026 | ko00260,ko01100 |
| pme3017 | 1.04E+02 | 5.80E+01 | 1.03E+02 | C_4_H_9_NO_2_ | [M+H]+ | 2-Aminoisobutyric acid | Organic acids | Organic acids | 62-57-7 | 2 | 2.61E+06 | 2.54E+06 | 2.76E+06 | 2.85E+06 | 2.67E+06 | 2.75E+06 | 3.22E+06 | 3.23E+06 | 3.12E+06 | 3.62E+06 | 3.68E+06 | 3.61E+06 | 2.89E+06 | 2.97E+06 | 3.13E+06 | C03665 | -- |
| Lmbn000193 | 1.03E+02 | 5.90E+01 | 1.04E+02 | C_3_H_4_O_4_ | [M-H]- | Tartronate semialdehyde | Organic acids | Organic acids | 2480-77-5 | 3 | 5.30E+05 | 6.29E+05 | 5.69E+05 | 4.07E+05 | 4.11E+05 | 4.32E+05 | 5.11E+05 | 4.42E+05 | 5.55E+05 | 3.02E+05 | 3.97E+05 | 3.83E+05 | 3.34E+05 | 4.71E+05 | 4.21E+05 | C01146 | ko00053,ko00630,ko01100 |
| ML10172161 | 1.03E+02 | 5.90E+01 | 1.04E+02 | C_3_H_4_O_4_ | [M-H]- | Hydroxypyruvic acid | Organic acids | Organic acids | 1113-60-6 | 3 | 5.33E+05 | 6.26E+05 | 5.84E+05 | 3.86E+05 | 3.72E+05 | 4.72E+05 | 5.52E+05 | 3.67E+05 | 5.17E+05 | 3.62E+05 | 3.31E+05 | 3.79E+05 | 3.16E+05 | 4.39E+05 | 3.83E+05 | C00168 | ko00260,ko00630,ko01100,ko01110,ko01200 |
| mws0576 | 1.03E+02 | 5.91E+01 | 1.04E+02 | C_4_H_8_O_3_ | [M-H]- | 3-Hydroxybutyric acid | Organic acids | Organic acids | 300-85-6 | 3 | 9.49E+06 | 1.13E+07 | 1.03E+07 | 8.90E+06 | 8.17E+06 | 9.57E+06 | 1.01E+07 | 7.50E+06 | 9.72E+06 | 7.00E+06 | 7.69E+06 | 7.38E+06 | 6.79E+06 | 7.62E+06 | 7.88E+06 | C01089 | ko00072,ko00650,ko01100 |
| pme1975 | 1.03E+02 | 5.90E+01 | 1.04E+02 | C_3_H_4_O_4_ | [M-H]- | Malonic acid | Organic acids | Organic acids | 141-82-2 | 3 | 7.70E+06 | 7.98E+06 | 7.91E+06 | 6.11E+06 | 6.25E+06 | 6.95E+06 | 7.23E+06 | 5.00E+06 | 7.38E+06 | 4.92E+06 | 5.64E+06 | 5.26E+06 | 5.01E+06 | 5.46E+06 | 5.39E+06 | C00383 | ko00061,ko00240,ko00410,ko01100,ko01212 |
| pmb0484 | 1.04E+02 | 6.01E+01 | 1.04E+02 | C_5_H_14_NO+ | [M]+ | Choline | Alkaloids | Alkaloids | 62-49-7 | 3 | 4.37E+06 | 4.36E+06 | 4.57E+06 | 4.91E+06 | 4.86E+06 | 4.52E+06 | 4.55E+06 | 4.67E+06 | 4.73E+06 | 5.35E+06 | 5.48E+06 | 5.45E+06 | 4.44E+06 | 5.01E+06 | 5.15E+06 | C00114 | ko00260,ko00564,ko01100,ko02010 |
| pme0010 | 1.06E+02 | 6.00E+01 | 1.05E+02 | C_3_H_7_NO_3_ | [M+H]+ | L-Serine | Amino acids and derivatives | Amino acids and derivatives | 56-45-1 | 3 | 1.90E+05 | 2.28E+05 | 2.34E+05 | 2.02E+05 | 1.64E+05 | 2.16E+05 | 3.21E+05 | 3.12E+05 | 2.39E+05 | 4.29E+05 | 5.10E+05 | 4.31E+05 | 2.65E+05 | 2.99E+05 | 2.97E+05 | C00065 | ko00260,ko00261,ko00270,ko00460,ko00564,ko00600,ko00630,ko00920,ko00970,ko01100,ko01110,ko01200,ko01230,ko01240,ko02010 |
| pme2433 | 1.06E+02 | 8.80E+01 | 1.05E+02 | C_4_H_11_NO_2_ | [M+H]+ | Diethanolamine | Alkaloids | Alkaloids | 111-42-2 | 3 | 6.23E+04 | 5.21E+04 | 5.39E+04 | 3.08E+04 | 3.42E+04 | 3.75E+04 | 5.42E+04 | 6.98E+04 | 4.77E+04 | 5.56E+04 | 6.38E+04 | 5.04E+04 | 3.90E+04 | 5.59E+04 | 5.66E+04 | C06772 | ko00564 |
| mws0601 | 1.10E+02 | 6.60E+01 | 1.11E+02 | C_5_H_5_NO_2_ | [M-H]- | Pyrrole-2-carboxylic acid | Organic acids | Organic acids | 634-97-9 | 3 | 4.26E+05 | 4.78E+05 | 4.57E+05 | 9.00E+00 | 9.00E+00 | 9.00E+00 | 1.09E+05 | 1.05E+05 | 1.11E+05 | 1.37E+04 | 1.56E+04 | 1.25E+04 | 1.65E+05 | 1.55E+05 | 1.65E+05 | C05942 | ko00330 |
| MWS5173 | 1.12E+02 | 7.00E+01 | 1.11E+02 | C_4_H_5_N_3_O | [M+H]+ | Isocytosine | Nucleotides and derivatives | Nucleotides and derivatives | 108-53-2 | 2 | 2.33E+06 | 2.31E+06 | 2.27E+06 | 1.19E+06 | 1.31E+06 | 1.20E+06 | 1.63E+06 | 1.60E+06 | 1.58E+06 | 1.18E+06 | 1.23E+06 | 1.07E+06 | 1.52E+06 | 1.53E+06 | 1.71E+06 | -- | -- |
| mws0255 | 1.12E+02 | 9.50E+01 | 1.11E+02 | C_4_H_5_N_3_O | [M+H]+ | Cytosine | Nucleotides and derivatives | Nucleotides and derivatives | 71-30-7 | 2 | 8.61E+05 | 8.66E+05 | 9.71E+05 | 9.22E+04 | 1.22E+05 | 1.32E+05 | 1.66E+05 | 3.38E+05 | 1.94E+05 | 8.06E+04 | 1.60E+05 | 9.06E+04 | 2.03E+05 | 1.80E+05 | 3.62E+05 | C00380 | ko00240,ko01100 |
| pme2122 | 1.12E+02 | 9.50E+01 | 1.11E+02 | C_5_H_9_N_3_ | [M+H]+ | Histamine | Alkaloids | Alkaloids | 51-45-6 | 3 | 1.08E+04 | 1.76E+04 | 7.58E+03 | 2.67E+04 | 2.54E+04 | 1.67E+04 | 2.63E+04 | 2.85E+04 | 2.48E+04 | 3.30E+04 | 3.96E+04 | 4.48E+04 | 2.70E+04 | 2.35E+04 | 2.40E+04 | C00388 | ko00340,ko01100,ko01110 |
| MWS1798 | 1.13E+02 | 6.71E+01 | 1.12E+02 | C_6_H_8_O_2_ | [M+H]+ | 2,4-Hexadienoic acid | Organic acids | Organic acids | 110-44-1 | 1 | 1.88E+05 | 1.91E+05 | 2.01E+05 | 2.82E+05 | 2.96E+05 | 2.88E+05 | 2.54E+05 | 2.57E+05 | 2.50E+05 | 3.52E+05 | 3.28E+05 | 3.03E+05 | 2.68E+05 | 2.65E+05 | 2.76E+05 | -- | -- |
| MWStz040 | 1.16E+02 | 7.01E+01 | 1.15E+02 | C_5_H_9_NO_2_ | [M+H]+ | Pterolactam | Alkaloids | Pyrrole alkaloids | 38072-88-7 | 1 | 6.46E+07 | 6.38E+07 | 6.10E+07 | 5.82E+06 | 6.21E+06 | 6.20E+06 | 2.70E+07 | 2.53E+07 | 2.45E+07 | 1.77E+07 | 1.75E+07 | 1.70E+07 | 2.72E+07 | 2.84E+07 | 2.92E+07 | -- | -- |
| pme0006 | 1.16E+02 | 7.01E+01 | 1.15E+02 | C_5_H_9_NO_2_ | [M+H]+ | L-Proline | Amino acids and derivatives | Amino acids and derivatives | 147-85-3 | 1 | 2.85E+06 | 2.84E+06 | 2.89E+06 | 1.79E+05 | 1.98E+05 | 2.02E+05 | 9.26E+05 | 9.25E+05 | 8.95E+05 | 6.02E+05 | 6.27E+05 | 6.02E+05 | 1.08E+06 | 1.10E+06 | 1.14E+06 | C00148 | ko00330,ko00332,ko00970,ko01100,ko01110,ko01230,ko02010 |
| mws0376 | 1.15E+02 | 7.10E+01 | 1.16E+02 | C_4_H_4_O_4_ | [M-H]- | Fumaric acid | Organic acids | Organic acids | 110-17-8 | 1 | 2.21E+06 | 2.21E+06 | 2.38E+06 | 6.55E+05 | 5.02E+05 | 4.98E+05 | 1.42E+06 | 1.48E+06 | 1.24E+06 | 3.53E+05 | 3.63E+05 | 4.22E+05 | 9.32E+05 | 1.12E+06 | 1.05E+06 | C00122 | ko00020,ko00190,ko00220,ko00250,ko00350,ko00360,ko00620,ko00650,ko00760,ko01100,ko01110,ko01200 |
| pmb1096 | 1.18E+02 | 9.10E+01 | 1.17E+02 | C_8_H_7_N | [M+H]+ | Indole | Alkaloids | Plumerane | 120-72-9 | 1 | 1.29E+04 | 1.51E+04 | 1.56E+04 | 2.02E+04 | 1.46E+04 | 1.62E+04 | 4.30E+04 | 4.20E+04 | 4.02E+04 | 5.21E+04 | 5.95E+04 | 6.83E+04 | 3.40E+04 | 3.53E+04 | 2.99E+04 | C00463 | ko00380,ko00400,ko00402,ko01100,ko01110 |
| pme0120 | 1.18E+02 | 1.01E+02 | 1.17E+02 | C_5_H_11_NO_2_ | [M+H]+ | 5-Aminovaleric acid | Organic acids | Organic acids | 660-88-8 | 3 | 7.44E+04 | 7.77E+04 | 7.17E+04 | 3.36E+04 | 2.70E+04 | 2.53E+04 | 1.27E+05 | 1.62E+05 | 1.70E+05 | 3.40E+04 | 4.83E+04 | 5.07E+04 | 6.72E+04 | 8.80E+04 | 8.31E+04 | C00431 | ko00310,ko00330,ko01100 |
| MWSmce548 | 1.18E+02 | 5.81E+01 | 1.17E+02 | C_5_H_11_NO_2_ | [M+H]+ | Betaine | Alkaloids | Alkaloids | 107-43-7 | 2 | 7.87E+06 | 7.35E+06 | 7.56E+06 | 4.71E+05 | 4.44E+05 | 3.44E+05 | 4.03E+06 | 4.07E+06 | 4.11E+06 | 3.56E+05 | 3.62E+05 | 3.65E+05 | 2.74E+06 | 2.63E+06 | 2.95E+06 | C00719 | ko00260,ko01100,ko02010 |
| mws0256 | 1.18E+02 | 7.21E+01 | 1.17E+02 | C_5_H_11_NO_2_ | [M+H]+ | L-Valine | Amino acids and derivatives | Amino acids and derivatives | 72-18-4 | 1 | 7.70E+06 | 8.01E+06 | 7.89E+06 | 4.49E+06 | 4.47E+06 | 4.42E+06 | 9.00E+06 | 9.07E+06 | 9.11E+06 | 9.80E+06 | 9.86E+06 | 9.70E+06 | 7.72E+06 | 7.31E+06 | 8.25E+06 | C00183 | ko00280,ko00290,ko00311,ko00460,ko00770,ko00966,ko00970,ko01100,ko01110,ko01210,ko01230,ko01240,ko02010 |
| mws0192 | 1.17E+02 | 7.30E+01 | 1.18E+02 | C_4_H_6_O_4_ | [M-H]- | Succinic acid | Organic acids | Organic acids | 110-15-6 | 1 | 2.20E+06 | 2.30E+06 | 2.23E+06 | 7.80E+05 | 8.28E+05 | 7.99E+05 | 1.74E+06 | 1.60E+06 | 1.74E+06 | 7.63E+05 | 6.34E+05 | 7.74E+05 | 1.41E+06 | 1.32E+06 | 1.55E+06 | C00042 | ko00020,ko00190,ko00250,ko00310,ko00350,ko00360,ko00620,ko00630,ko00640,ko00650,ko00760,ko00920,ko01100,ko01110,ko01200 |
| mws0470 | 1.17E+02 | 7.30E+01 | 1.18E+02 | C_4_H_6_O_4_ | [M-H]- | Methylmalonic acid | Organic acids | Organic acids | 516-05-2 | 1 | 2.11E+06 | 2.20E+06 | 2.15E+06 | 7.31E+05 | 8.75E+05 | 7.81E+05 | 1.64E+06 | 1.73E+06 | 1.73E+06 | 6.69E+05 | 7.09E+05 | 7.18E+05 | 1.42E+06 | 1.57E+06 | 1.63E+06 | C02170 | ko00240,ko00280,ko00640,ko01100 |
| pme2923 | 1.17E+02 | 7.50E+01 | 1.18E+02 | C_4_H_6_O_4_ | [M-H]- | Acetoxyacetic acid | Organic acids | Organic acids | 13831-30-6 | 3 | 6.34E+05 | 6.05E+05 | 5.61E+05 | 9.00E+00 | 9.00E+00 | 9.00E+00 | 5.75E+05 | 5.65E+05 | 5.18E+05 | 9.00E+00 | 9.00E+00 | 9.00E+00 | 3.03E+05 | 3.32E+05 | 3.37E+05 | -- | -- |
| mws0147 | 1.17E+02 | 5.90E+01 | 1.18E+02 | C_5_H_10_O_3_ | [M-H]- | β-Hydroxyisovaleric acid | Organic acids | Organic acids | 625-08-1 | 1 | 8.53E+05 | 7.99E+05 | 8.02E+05 | 6.08E+05 | 5.59E+05 | 5.84E+05 | 7.52E+05 | 7.98E+05 | 8.15E+05 | 8.00E+05 | 7.37E+05 | 7.39E+05 | 6.82E+05 | 7.71E+05 | 7.22E+05 | C20827 | ko00280,ko01100 |
| pme3096 | 1.18E+02 | 7.40E+01 | 1.19E+02 | C_3_H_5_NO_4_ | [M-H]- | Aminomalonic acid | Organic acids | Organic acids | 1068-84-4 | 3 | 3.17E+06 | 3.46E+06 | 3.44E+06 | 1.27E+06 | 1.26E+06 | 1.26E+06 | 2.70E+06 | 2.59E+06 | 2.69E+06 | 1.12E+06 | 1.10E+06 | 1.18E+06 | 2.14E+06 | 2.23E+06 | 2.46E+06 | C00872 | -- |
| mws0671 | 1.20E+02 | 7.41E+01 | 1.19E+02 | C_4_H_9_NO_3_ | [M+H]+ | L-Homoserine | Amino acids and derivatives | Amino acids and derivatives | 672-15-1 | 3 | 3.83E+05 | 3.83E+05 | 3.94E+05 | 2.96E+05 | 3.19E+05 | 3.65E+05 | 4.45E+05 | 5.52E+05 | 4.45E+05 | 5.81E+05 | 5.47E+05 | 5.55E+05 | 2.88E+05 | 4.37E+05 | 3.44E+05 | C00263 | ko00260,ko00270,ko00300,ko00920,ko01100,ko01110,ko01230 |
| mws0230 | 1.20E+02 | 7.40E+01 | 1.19E+02 | C_4_H_9_NO_3_ | [M+H]+ | L-Threonine | Amino acids and derivatives | Amino acids and derivatives | 72-19-5 | 3 | 1.17E+06 | 1.13E+06 | 1.10E+06 | 8.33E+05 | 9.84E+05 | 7.84E+05 | 1.61E+06 | 1.34E+06 | 1.57E+06 | 2.08E+06 | 2.05E+06 | 2.00E+06 | 1.42E+06 | 1.30E+06 | 1.51E+06 | C00188 | ko00260,ko00261,ko00290,ko00860,ko00970,ko01100,ko01110,ko01230,ko02010 |
| pmp001287 | 1.20E+02 | 1.03E+02 | 1.19E+02 | C_8_H_9_N | [M+H]+ | N-Benzylmethylene isomethylamine | Alkaloids | Alkaloids | - | 1 | 6.99E+06 | 6.49E+06 | 6.93E+06 | 8.42E+06 | 8.45E+06 | 8.31E+06 | 9.95E+06 | 1.06E+07 | 1.06E+07 | 1.31E+07 | 1.44E+07 | 1.35E+07 | 1.00E+07 | 9.50E+06 | 1.09E+07 | -- | -- |
| pme0195 | 1.20E+02 | 6.20E+01 | 1.21E+02 | C_3_H_7_NO_2_S | [M-H]- | L-Cysteine | Amino acids and derivatives | Amino acids and derivatives | 52-90-4 | 3 | 9.34E+03 | 9.77E+03 | 7.07E+03 | 9.06E+03 | 9.41E+03 | 9.41E+03 | 1.24E+04 | 1.21E+04 | 1.09E+04 | 1.05E+04 | 1.04E+04 | 1.41E+04 | 7.04E+03 | 1.29E+04 | 1.21E+04 | C00097 | ko00260,ko00270,ko00311,ko00332,ko00430,ko00460,ko00480,ko00730,ko00770,ko00920,ko00970,ko01100,ko01110,ko01200,ko01230,ko01240,ko04122 |
| pmb0069 | 1.22E+02 | 1.05E+02 | 1.21E+02 | C_7_H_7_NO | [M+H]+ | Benzamide | Phenolic acids | Phenolic acids | 55-21-0 | 3 | 2.96E+04 | 2.84E+04 | 3.71E+04 | 9.00E+00 | 9.00E+00 | 9.00E+00 | 2.08E+04 | 3.09E+04 | 2.49E+04 | 1.38E+04 | 2.80E+04 | 1.84E+04 | 1.62E+04 | 2.21E+04 | 2.02E+04 | C09815 | -- |
| mws0491 | 1.22E+02 | 1.05E+02 | 1.21E+02 | C_8_H_11_N | [M+H]+ | Phenethylamine | Alkaloids | Alkaloids | 64-04-0 | 2 | 2.94E+05 | 2.66E+05 | 4.44E+05 | 5.88E+04 | 8.10E+04 | 7.31E+04 | 2.26E+05 | 2.35E+05 | 2.56E+05 | 1.68E+05 | 1.78E+05 | 1.65E+05 | 2.11E+05 | 1.68E+05 | 2.11E+05 | C05332 | ko00360,ko01100 |
| mws0628 | 1.21E+02 | 9.20E+01 | 1.22E+02 | C_7_H_6_O_2_ | [M-H]- | 4-Hydroxybenzaldehyde | Phenolic acids | Phenolic acids | 123-08-0 | 1 | 1.34E+06 | 1.27E+06 | 1.12E+06 | 2.05E+05 | 1.71E+05 | 1.72E+05 | 1.18E+06 | 1.28E+06 | 1.32E+06 | 2.93E+05 | 3.31E+05 | 3.02E+05 | 6.85E+05 | 7.04E+05 | 7.23E+05 | C00633 | ko01100 |
| mws0133 | 1.23E+02 | 8.01E+01 | 1.22E+02 | C_6_H_6_N_2_O | [M+H]+ | Nicotinamide | Others | Vitamin | 98-92-0 | 1 | 5.63E+06 | 5.76E+06 | 6.18E+06 | 4.38E+06 | 4.25E+06 | 4.26E+06 | 9.42E+06 | 8.74E+06 | 9.59E+06 | 3.50E+06 | 3.48E+06 | 3.48E+06 | 5.19E+06 | 5.51E+06 | 6.15E+06 | C00153 | ko00760,ko01100,ko01240 |
| MWSmce465 | 1.23E+02 | 8.21E+01 | 1.22E+02 | C_7_H_10_N_2_ | [M+H]+ | 2,3,5-Trimethylpyrazine | Others | Others | 14667-55-1 | 1 | 1.35E+05 | 1.76E+05 | 2.20E+05 | 9.00E+00 | 9.00E+00 | 9.00E+00 | 9.00E+00 | 9.00E+00 | 9.00E+00 | 9.00E+00 | 9.00E+00 | 9.00E+00 | 9.36E+04 | 6.21E+04 | 7.91E+04 | -- | -- |
| Lmbp000668 | 1.24E+02 | 7.80E+01 | 1.23E+02 | C_6_H_5_NO_2_ | [M+H]+ | Isonicotinic acid | Others | Vitamin | 55-22-1 | 2 | 5.50E+06 | 5.03E+06 | 4.74E+06 | 8.80E+05 | 1.00E+06 | 8.93E+05 | 2.70E+06 | 2.64E+06 | 2.95E+06 | 6.89E+05 | 7.14E+05 | 6.76E+05 | 2.59E+06 | 2.33E+06 | 2.88E+06 | C07446 | -- |
| pme1216 | 1.22E+02 | 7.80E+01 | 1.23E+02 | C_6_H_5_NO_2_ | [M-H]- | 2-Picolinic acid | Organic acids | Organic acids | 98-98-6 | 3 | 3.91E+06 | 3.81E+06 | 3.74E+06 | 5.94E+05 | 6.15E+05 | 5.24E+05 | 2.11E+06 | 2.02E+06 | 1.98E+06 | 3.55E+05 | 4.00E+05 | 3.63E+05 | 1.60E+06 | 1.85E+06 | 1.92E+06 | C10164 | ko00380,ko01100 |
| pme0490 | 1.24E+02 | 7.80E+01 | 1.23E+02 | C_6_H_5_NO_2_ | [M+H]+ | Nicotinic acid (Vitamin B3) | Others | Vitamin | 59-67-6 | 1 | 5.25E+06 | 5.24E+06 | 4.91E+06 | 9.31E+05 | 1.01E+06 | 8.96E+05 | 2.70E+06 | 2.80E+06 | 2.78E+06 | 6.88E+05 | 7.01E+05 | 6.41E+05 | 2.54E+06 | 2.33E+06 | 2.77E+06 | C00253 | ko00760,ko00960,ko01100,ko01110,ko01240 |
| mws0236 | 1.24E+02 | 8.00E+01 | 1.25E+02 | C_2_H_7_NO_3_S | [M-H]- | 2-Aminoethanesulfonic acid | Organic acids | Organic acids | 107-35-7 | 3 | 2.97E+03 | 3.35E+03 | 2.30E+03 | 5.31E+03 | 7.32E+03 | 2.96E+03 | 5.97E+03 | 6.03E+03 | 4.93E+03 | 6.02E+03 | 3.31E+03 | 5.27E+03 | 5.33E+03 | 5.65E+03 | 5.91E+03 | C00245 | ko00430,ko00920,ko01100,ko02010 |
| mws0572 | 1.26E+02 | 1.09E+02 | 1.25E+02 | C_5_H_7_N_3_O | [M+H]+ | 5-Methylcytosine | Nucleotides and derivatives | Nucleotides and derivatives | 554-01-8 | 3 | 2.30E+04 | 3.85E+04 | 3.43E+04 | 9.00E+00 | 9.00E+00 | 9.00E+00 | 3.10E+03 | 4.40E+03 | 4.42E+03 | 9.00E+00 | 9.00E+00 | 9.00E+00 | 1.06E+04 | 1.10E+04 | 1.22E+04 | C02376 | ko00240,ko01100 |
| pmp001086 | 1.27E+02 | 9.70E+01 | 1.26E+02 | C_6_H_6_O_3_ | [M+H]+ | 5-Hydroxymethylfurfural | Others | Others | 67-47-0 | 3 | 1.27E+05 | 1.75E+05 | 1.31E+05 | 1.59E+05 | 2.17E+05 | 1.35E+05 | 1.48E+05 | 1.25E+05 | 1.14E+05 | 1.17E+05 | 1.13E+05 | 1.05E+05 | 1.05E+05 | 1.43E+05 | 1.35E+05 | C11101 | -- |
| mws0251 | 1.27E+02 | 1.10E+02 | 1.26E+02 | C_5_H_6_N_2_O_2_ | [M+H]+ | Thymine | Nucleotides and derivatives | Nucleotides and derivatives | 65-71-4 | 3 | 6.68E+05 | 6.79E+05 | 7.60E+05 | 9.00E+00 | 9.00E+00 | 9.00E+00 | 3.15E+04 | 2.92E+04 | 3.21E+04 | 9.00E+00 | 9.00E+00 | 9.00E+00 | 2.32E+05 | 2.28E+05 | 2.74E+05 | C00178 | ko00240,ko01100 |
| pmb0812 | 1.27E+02 | 8.12E+01 | 1.26E+02 | C_5_H_6_N_2_O_2_ | [M+H]+ | Imidazole-4-Acetic Acid | Alkaloids | Alkaloids | 645-65-8 | 3 | 6.45E+05 | 7.22E+05 | 9.87E+05 | 8.71E+05 | 1.04E+06 | 8.43E+05 | 7.54E+05 | 6.81E+05 | 8.06E+05 | 7.12E+05 | 8.43E+05 | 9.23E+05 | 8.66E+05 | 8.38E+05 | 6.80E+05 | C02835 | ko00340,ko01100 |
| mws0263 | 1.28E+02 | 8.20E+01 | 1.29E+02 | C_5_H_7_NO_3_ | [M-H]- | 5-Oxo-L-Proline | Amino acids and derivatives | Amino acids and derivatives | 98-79-3 | 3 | 1.55E+05 | 1.58E+05 | 8.09E+04 | 4.79E+03 | 4.60E+03 | 8.47E+03 | 1.04E+05 | 1.39E+05 | 8.44E+04 | 1.34E+04 | 2.13E+04 | 2.59E+04 | 7.08E+04 | 7.42E+04 | 6.59E+04 | C01879 | ko00480,ko01100 |
| MWSmce709 | 1.30E+02 | 1.03E+02 | 1.29E+02 | C_9_H_7_N | [M+H]+ | Isoquinoline | Alkaloids | Isoquinoline alkaloids | 119-65-3 | 2 | 7.52E+04 | 7.35E+04 | 5.47E+04 | 1.04E+05 | 8.99E+04 | 9.87E+04 | 2.07E+05 | 1.67E+05 | 1.82E+05 | 2.37E+05 | 2.58E+05 | 2.78E+05 | 1.80E+05 | 1.51E+05 | 1.72E+05 | C06323 | -- |
| ML10181668 | 1.30E+02 | 8.41E+01 | 1.29E+02 | C_6_H_11_NO_2_ | [M+H]+ | Cycloleucine | Amino acids and derivatives | Amino acids and derivatives | 52-52-8 | 1 | 5.69E+05 | 5.21E+05 | 6.12E+05 | 9.89E+05 | 9.78E+05 | 9.64E+05 | 1.04E+06 | 1.01E+06 | 1.06E+06 | 1.61E+06 | 1.73E+06 | 1.76E+06 | 1.11E+06 | 1.05E+06 | 1.18E+06 | C03969 | -- |
| MWS0811 | 1.30E+02 | 8.41E+01 | 1.29E+02 | C_6_H_11_NO_2_ | [M+H]+ | L-Pipecolic Acid | Organic acids | Organic acids | 3105-95-1 | 1 | 5.81E+07 | 5.59E+07 | 5.75E+07 | 3.37E+07 | 3.22E+07 | 3.28E+07 | 5.69E+07 | 5.95E+07 | 6.20E+07 | 4.49E+07 | 4.67E+07 | 4.60E+07 | 4.84E+07 | 4.70E+07 | 5.44E+07 | C00408 | ko00310,ko00960,ko01100,ko01110 |
| Zmdp000376 | 1.30E+02 | 8.41E+01 | 1.29E+02 | C_5_H_11_N_3_O | [M+H]+ | 4-Guanidinobutanal | Others | Others | - | 1 | 2.38E+07 | 2.99E+07 | 3.49E+07 | 1.93E+07 | 2.05E+07 | 1.92E+07 | 3.63E+07 | 3.57E+07 | 2.49E+07 | 2.55E+07 | 2.75E+07 | 2.28E+07 | 2.63E+07 | 2.83E+07 | 3.14E+07 | -- | -- |
| Zmgn000217 | 1.29E+02 | 8.50E+01 | 1.30E+02 | C_5_H_6_O_4_ | [M-H]- | Methylenesuccinic acid | Organic acids | Organic acids | 97-65-4 | 1 | 2.77E+05 | 2.22E+05 | 1.81E+05 | 5.74E+04 | 1.48E+05 | 1.46E+05 | 4.18E+05 | 3.68E+05 | 6.18E+05 | 2.53E+05 | 2.90E+05 | 2.57E+05 | 3.68E+05 | 3.94E+05 | 3.79E+05 | C00490 | ko00660,ko01100 |
| pme2693 | 1.31E+02 | 1.14E+02 | 1.30E+02 | C_6_H_14_N_2_O | [M+H]+ | N-Acetylputrescine | Alkaloids | Alkaloids | 18233-70-0 | 3 | 2.65E+05 | 2.84E+05 | 3.08E+05 | 3.73E+04 | 3.52E+04 | 3.64E+04 | 5.44E+04 | 6.66E+04 | 5.99E+04 | 2.53E+04 | 1.54E+04 | 2.63E+04 | 1.02E+05 | 1.20E+05 | 1.16E+05 | C02714 | ko00330,ko01100 |
| pmb0501 | 1.31E+02 | 1.14E+02 | 1.30E+02 | C_5_H_14_N_4_ | [M+H]+ | Agmatine | Alkaloids | Alkaloids | 306-60-5 | 3 | 2.80E+05 | 2.77E+05 | 3.10E+05 | 3.39E+04 | 3.17E+04 | 3.12E+04 | 6.52E+04 | 6.14E+04 | 7.39E+04 | 1.60E+04 | 1.74E+04 | 2.29E+04 | 9.64E+04 | 9.41E+04 | 1.21E+05 | C00179 | ko00330,ko01100 |
| Zmpn000638 | 1.30E+02 | 8.80E+01 | 1.31E+02 | C_4_H_9_N_3_O_2_ | [M-H]- | 3-Guanidinopropionic acid | Organic acids | Organic acids | 353-09-3 | 2 | 5.77E+05 | 5.86E+05 | 7.16E+05 | 5.47E+04 | 5.94E+04 | 5.14E+04 | 1.71E+05 | 1.90E+05 | 1.91E+05 | 5.84E+04 | 6.27E+04 | 5.97E+04 | 2.36E+05 | 2.77E+05 | 2.74E+05 | C03065 | -- |
| ML10197929 | 1.30E+02 | 8.80E+01 | 1.31E+02 | C_4_H_9_N_3_O_2_ | [M-H]- | Creatine | Organic acids | Organic acids | 57-00-1 | 2 | 2.28E+04 | 2.33E+04 | 2.33E+04 | 1.50E+04 | 1.83E+04 | 8.21E+03 | 1.93E+04 | 2.04E+04 | 2.79E+04 | 2.90E+04 | 3.31E+04 | 2.11E+04 | 1.06E+04 | 2.77E+04 | 2.16E+04 | C00300 | ko00260,ko00330,ko01100 |
| pmp001198 | 1.32E+02 | 5.71E+01 | 1.31E+02 | C_6_H_13_NO_2_ | [M+H]+ | 6-Deoxyfagomine | Alkaloids | Piperidine alkaloids | 197449-09-5 | 1 | 4.46E+06 | 4.23E+06 | 3.92E+06 | 3.63E+06 | 3.60E+06 | 3.60E+06 | 3.86E+06 | 3.86E+06 | 3.82E+06 | 4.64E+06 | 4.70E+06 | 4.94E+06 | 4.18E+06 | 3.79E+06 | 4.70E+06 | -- | -- |
| mws1587 | 1.32E+02 | 8.60E+01 | 1.31E+02 | C_6_H_13_NO_2_ | [M+H]+ | L-Norleucine* | Amino acids and derivatives | Amino acids and derivatives | 327-57-1 | 1 | 1.83E+06 | 1.77E+06 | 1.70E+06 | 1.15E+06 | 1.07E+06 | 1.09E+06 | 1.56E+06 | 1.52E+06 | 1.59E+06 | 1.44E+06 | 1.51E+06 | 1.48E+06 | 1.64E+06 | 1.45E+06 | 1.60E+06 | C01933 | -- |
| mws0258 | 1.32E+02 | 8.60E+01 | 1.31E+02 | C_6_H_13_NO_2_ | [M+H]+ | L-Isoleucine* | Amino acids and derivatives | Amino acids and derivatives | 73-32-5 | 1 | 1.82E+06 | 1.82E+06 | 1.76E+06 | 1.16E+06 | 1.12E+06 | 1.07E+06 | 1.63E+06 | 1.50E+06 | 1.56E+06 | 1.49E+06 | 1.48E+06 | 1.50E+06 | 1.58E+06 | 1.43E+06 | 1.71E+06 | C00407 | ko00280,ko00290,ko00460,ko00960,ko00966,ko00970,ko01100,ko01110,ko01210,ko01230,ko02010 |
| pme0274 | 1.32E+02 | 6.90E+01 | 1.31E+02 | C_6_H_13_NO_2_ | [M+H]+ | 6-Aminocaproic acid | Organic acids | Organic acids | 60-32-2 | 2 | 2.52E+06 | 2.47E+06 | 2.69E+06 | 2.27E+06 | 2.01E+06 | 1.92E+06 | 2.71E+06 | 2.37E+06 | 2.74E+06 | 2.97E+06 | 2.97E+06 | 3.25E+06 | 2.41E+06 | 2.79E+06 | 2.86E+06 | C02378 | ko01100 |
| mws0227 | 1.32E+02 | 8.62E+01 | 1.31E+02 | C_6_H_13_NO_2_ | [M+H]+ | L-Leucine* | Amino acids and derivatives | Amino acids and derivatives | 61-90-5 | 1 | 1.71E+06 | 1.78E+06 | 1.53E+06 | 1.07E+06 | 1.04E+06 | 1.10E+06 | 1.58E+06 | 1.55E+06 | 1.55E+06 | 1.51E+06 | 1.43E+06 | 1.43E+06 | 1.46E+06 | 1.46E+06 | 1.65E+06 | C00123 | ko00280,ko00290,ko00966,ko00970,ko01100,ko01110,ko01210,ko01230,ko02010 |
| pme0243 | 1.31E+02 | 8.71E+01 | 1.32E+02 | C_5_H_8_O_4_ | [M-H]- | Glutaric acid | Organic acids | Organic acids | 110-94-1 | 3 | 4.41E+05 | 3.85E+05 | 4.57E+05 | 8.05E+04 | 1.31E+05 | 1.12E+05 | 2.25E+05 | 2.56E+05 | 3.37E+05 | 6.43E+04 | 3.25E+04 | 7.77E+04 | 1.93E+05 | 2.05E+05 | 2.36E+05 | C00489 | ko00071,ko00310,ko01100 |
| Lmbn001364 | 1.31E+02 | 8.71E+01 | 1.32E+02 | C_5_H_8_O_4_ | [M-H]- | 4-Hydroxy-2-Oxopentanoic Acid | Organic acids | Organic acids | 3318-73-8 | 3 | 1.93E+05 | 1.80E+05 | 1.74E+05 | 2.06E+05 | 2.08E+05 | 2.33E+05 | 2.06E+05 | 1.92E+05 | 2.13E+05 | 2.15E+05 | 2.26E+05 | 2.00E+05 | 1.99E+05 | 2.15E+05 | 2.38E+05 | C03589 | ko00360,ko01100 |
| Lmbn001288 | 1.31E+02 | 8.70E+01 | 1.32E+02 | C_5_H_8_O_4_ | [M-H]- | 2-Hydroxy-2-methyl-3-oxobutanoic acid | Organic acids | Organic acids | 71698-08-3 | 3 | 1.72E+05 | 1.66E+05 | 1.79E+05 | 2.11E+05 | 1.90E+05 | 2.20E+05 | 2.18E+05 | 2.03E+05 | 2.19E+05 | 2.18E+05 | 2.13E+05 | 2.16E+05 | 1.93E+05 | 2.14E+05 | 2.47E+05 | C06010 | ko00290,ko00650,ko00660,ko00770,ko01100,ko01110,ko01210,ko01230 |
| mws0473 | 1.31E+02 | 8.71E+01 | 1.32E+02 | C_5_H_8_O_4_ | [M-H]- | 2-Methylsuccinic acid | Organic acids | Organic acids | 498-21-5 | 3 | 1.71E+06 | 1.88E+06 | 1.80E+06 | 2.12E+06 | 2.27E+06 | 2.17E+06 | 2.14E+06 | 2.03E+06 | 2.20E+06 | 2.29E+06 | 2.43E+06 | 2.38E+06 | 2.06E+06 | 2.00E+06 | 2.28E+06 | -- | -- |
| Lmgn000160 | 1.31E+02 | 7.00E+01 | 1.32E+02 | C_4_H_8_N_2_O_3_ | [M-H]- | 3-Ureidopropionic Acid | Organic acids | Organic acids | 462-88-4 | 2 | 5.49E+04 | 5.44E+04 | 5.58E+04 | 6.46E+04 | 7.22E+04 | 7.16E+04 | 1.38E+05 | 1.46E+05 | 1.56E+05 | 1.79E+05 | 1.77E+05 | 1.70E+05 | 1.00E+05 | 1.12E+05 | 1.11E+05 | C02642 | ko00240,ko00410,ko00770,ko01100 |
| mws0001 | 1.33E+02 | 7.40E+01 | 1.32E+02 | C_4_H_8_N_2_O_3_ | [M+H]+ | L-Asparagine | Amino acids and derivatives | Amino acids and derivatives | 70-47-3 | 3 | 1.37E+05 | 1.97E+05 | 1.76E+05 | 1.61E+05 | 1.67E+05 | 1.79E+05 | 2.92E+05 | 3.11E+05 | 2.62E+05 | 3.50E+05 | 2.88E+05 | 3.23E+05 | 1.50E+05 | 2.01E+05 | 2.06E+05 | C00152 | ko00250,ko00460,ko00970,ko01100,ko01110,ko01230 |
| mws0341 | 1.31E+02 | 8.50E+01 | 1.32E+02 | C_6_H_12_O_3_ | [M-H]- | 2-Hydroxyisocaproic acid | Organic acids | Organic acids | 20312-37-2 | 3 | 1.09E+06 | 1.04E+06 | 1.22E+06 | 1.20E+05 | 1.63E+05 | 1.39E+05 | 2.84E+05 | 1.91E+05 | 2.43E+05 | 1.30E+05 | 1.47E+05 | 1.41E+05 | 4.45E+05 | 4.22E+05 | 5.16E+05 | C03264 | -- |
| mws0972 | 1.31E+02 | 8.50E+01 | 1.32E+02 | C_6_H_12_O_3_ | [M-H]- | 6-Hydroxyhexanoic acid | Organic acids | Organic acids | 1191-25-9 | 3 | 6.84E+04 | 5.25E+04 | 7.66E+04 | 2.40E+04 | 1.55E+04 | 1.87E+04 | 4.33E+04 | 1.51E+04 | 2.80E+04 | 7.27E+03 | 2.00E+04 | 3.42E+04 | 3.94E+04 | 3.37E+04 | 4.99E+04 | C06103 | -- |
| pme2527 | 1.33E+02 | 1.16E+02 | 1.32E+02 | C_5_H_12_N_2_O_2_ | [M+H]+ | L-Ornithine | Amino acids and derivatives | Amino acids and derivatives | 70-26-8 | 3 | 2.34E+06 | 2.35E+06 | 3.06E+06 | 4.86E+05 | 4.81E+05 | 5.00E+05 | 6.69E+05 | 6.63E+05 | 7.69E+05 | 7.23E+05 | 7.93E+05 | 7.38E+05 | 9.66E+05 | 1.01E+06 | 1.16E+06 | C00077 | ko00220,ko00330,ko00472,ko00480,ko01100,ko01110,ko01210,ko01230,ko02010 |
| MWS1882 | 1.32E+02 | 8.80E+01 | 1.33E+02 | C_4_H_7_NO_4_ | [M-H]- | Iminodiacetic acid | Organic acids | Organic acids | 142-73-4 | 2 | 5.03E+04 | 5.77E+04 | 5.80E+04 | 6.47E+05 | 6.40E+05 | 6.85E+05 | 1.31E+05 | 1.36E+05 | 1.55E+05 | 4.94E+05 | 5.28E+05 | 4.43E+05 | 3.14E+05 | 3.31E+05 | 3.50E+05 | C19911 | -- |
| MWSmce609 | 1.34E+02 | 7.40E+01 | 1.33E+02 | C_4_H_7_NO_4_ | [M+H]+ | L-Aspartic acid | Amino acids and derivatives | Amino acids and derivatives | 56-84-8 | 2 | 2.20E+05 | 2.33E+05 | 2.57E+05 | 9.98E+05 | 1.04E+06 | 9.95E+05 | 1.96E+05 | 2.15E+05 | 2.16E+05 | 6.92E+05 | 6.82E+05 | 5.39E+05 | 5.01E+05 | 4.99E+05 | 5.29E+05 | C00049 | ko00220,ko00250,ko00260,ko00261,ko00270,ko00300,ko00340,ko00410,ko00460,ko00710,ko00760,ko00770,ko00970,ko01100,ko01110,ko01200,ko01210,ko01230,ko01240,ko02010 |
| Lmbn000198 | 1.33E+02 | 7.10E+01 | 1.34E+02 | C_4_H_6_O_5_ | [M-H]- | 3-Dehydro-L-Threonic Acid | Others | Saccharides and Alcohols | - | 1 | 9.37E+05 | 9.63E+05 | 9.63E+05 | 1.46E+06 | 1.55E+06 | 1.58E+06 | 1.00E+06 | 9.20E+05 | 1.11E+06 | 1.07E+06 | 9.39E+05 | 1.23E+06 | 1.02E+06 | 1.30E+06 | 1.25E+06 | C03064 | ko00053,ko01100 |
| MWS1709 | 1.33E+02 | 7.10E+01 | 1.34E+02 | C_4_H_6_O_5_ | [M-H]- | D-Malic acid | Organic acids | Organic acids | 636-61-3 | 1 | 1.06E+06 | 9.37E+05 | 8.33E+05 | 1.43E+06 | 1.43E+06 | 1.28E+06 | 7.56E+05 | 9.91E+05 | 1.10E+06 | 9.19E+05 | 1.09E+06 | 1.26E+06 | 9.54E+05 | 1.29E+06 | 9.46E+05 | C00497 | ko00650,ko01100 |
| mws0275 | 1.33E+02 | 7.10E+01 | 1.34E+02 | C_4_H_6_O_5_ | [M-H]- | L-Malic acid | Organic acids | Organic acids | 97-67-6 | 1 | 1.22E+07 | 1.05E+07 | 1.19E+07 | 1.85E+07 | 1.77E+07 | 1.79E+07 | 1.31E+07 | 1.25E+07 | 1.33E+07 | 1.44E+07 | 1.26E+07 | 1.39E+07 | 1.36E+07 | 1.59E+07 | 1.49E+07 | C00149 | ko00020,ko00620,ko00630,ko00710,ko01100,ko01110,ko01200 |
| mws5028 | 1.36E+02 | 8.01E+01 | 1.35E+02 | C_7_H_5_NO_2_ | [M+H]+ | 2-Benzoxazolinone | Others | Others | 59-49-4 | 2 | 7.52E+04 | 6.68E+04 | 7.07E+04 | 9.00E+00 | 9.00E+00 | 9.00E+00 | 5.48E+04 | 4.44E+04 | 5.03E+04 | 9.00E+00 | 9.00E+00 | 9.00E+00 | 3.82E+04 | 4.59E+04 | 2.52E+04 | -- | -- |
| Zmdp000972 | 1.36E+02 | 9.11E+01 | 1.35E+02 | C_4_H_9_NO_2_S | [M+H]+ | S-Methyl-L-cysteine | Amino acids and derivatives | Amino acids and derivatives | 1187-84-4 | 2 | 1.45E+06 | 1.35E+06 | 1.46E+06 | 1.69E+06 | 1.75E+06 | 1.66E+06 | 3.30E+06 | 3.33E+06 | 3.50E+06 | 4.86E+06 | 4.55E+06 | 4.52E+06 | 2.89E+06 | 2.55E+06 | 2.96E+06 | C22040 | ko02010 |
| pme0040 | 1.36E+02 | 1.19E+02 | 1.35E+02 | C_5_H_5_N_5_ | [M+H]+ | Adenine | Nucleotides and derivatives | Nucleotides and derivatives | 73-24-5 | 1 | 1.05E+07 | 1.02E+07 | 9.30E+06 | 3.02E+06 | 3.10E+06 | 2.86E+06 | 4.03E+06 | 3.98E+06 | 4.17E+06 | 2.72E+06 | 3.17E+06 | 2.83E+06 | 5.31E+06 | 5.09E+06 | 5.72E+06 | C00147 | ko00230,ko00908,ko01100 |
| pmb0374 | 1.36E+02 | 1.19E+02 | 1.35E+02 | C_5_H_5_N_5_ | [M+H]+ | 2-Aminopurine | Nucleotides and derivatives | Nucleotides and derivatives | 452-06-2 | 1 | 7.81E+05 | 8.48E+05 | 7.55E+05 | 1.78E+05 | 1.93E+05 | 1.93E+05 | 3.62E+05 | 3.82E+05 | 3.47E+05 | 1.76E+05 | 1.82E+05 | 1.53E+05 | 4.32E+05 | 3.69E+05 | 4.49E+05 | -- | -- |
| Lmxp000939 | 1.36E+02 | 1.19E+02 | 1.35E+02 | C_5_H_5_N_5_ | [M+H]+ | Zarzissine | Alkaloids | Alkaloids | 160568-14-9 | 1 | 1.10E+07 | 1.07E+07 | 9.64E+06 | 3.02E+06 | 3.23E+06 | 3.00E+06 | 3.77E+06 | 3.66E+06 | 3.97E+06 | 2.56E+06 | 2.91E+06 | 2.49E+06 | 5.36E+06 | 5.21E+06 | 5.57E+06 | -- | -- |
| HJKP000649 | 1.36E+02 | 9.11E+01 | 1.35E+02 | C_8_H_9_NO | [M+H]+ | N-benzylformamide | Alkaloids | Alkaloids | 6343-54-0 | 1 | 2.19E+06 | 2.12E+06 | 2.19E+06 | 2.76E+06 | 2.51E+06 | 2.46E+06 | 5.06E+06 | 5.03E+06 | 5.40E+06 | 7.39E+06 | 6.98E+06 | 7.03E+06 | 4.35E+06 | 3.89E+06 | 4.45E+06 | C15561 | -- |
| mws0889 | 1.35E+02 | 7.50E+01 | 1.36E+02 | C_4_H_8_O_5_ | [M-H]- | D-Threonic Acid | Others | Saccharides and Alcohols | 3909-12-4 | 3 | 1.42E+06 | 1.30E+06 | 1.15E+06 | 6.01E+05 | 6.28E+05 | 5.37E+05 | 4.68E+05 | 3.99E+05 | 5.33E+05 | 3.03E+05 | 3.43E+05 | 3.26E+05 | 6.29E+05 | 7.22E+05 | 6.32E+05 | C21649 | -- |
| pme0033 | 1.37E+02 | 1.19E+02 | 1.36E+02 | C_5_H_4_N_4_O | [M+H]+ | Hypoxanthine | Nucleotides and derivatives | Nucleotides and derivatives | 68-94-0 | 3 | 9.40E+05 | 1.08E+06 | 9.00E+05 | 2.83E+04 | 3.89E+04 | 3.18E+04 | 1.49E+05 | 1.44E+05 | 1.65E+05 | 1.20E+04 | 1.97E+04 | 1.16E+04 | 2.83E+05 | 2.51E+05 | 3.66E+05 | C00262 | ko00230,ko01100 |
| MWSmce466 | 1.35E+02 | 9.20E+01 | 1.36E+02 | C_8_H_8_O_2_ | [M-H]- | 4'-Hydroxyacetophenone | Phenolic acids | Phenolic acids | 99-93-4 | 1 | 1.12E+05 | 1.32E+05 | 1.28E+05 | 5.99E+04 | 7.08E+04 | 5.46E+04 | 8.62E+04 | 1.06E+05 | 7.68E+04 | 2.98E+04 | 4.64E+04 | 4.74E+04 | 6.76E+04 | 9.11E+04 | 6.73E+04 | C10700 | ko01100 |
| MWS1848 | 1.35E+02 | 9.20E+01 | 1.36E+02 | C_8_H_8_O_2_ | [M-H]- | Phenyl acetate | Phenolic acids | Phenolic acids | 122-79-2 | 1 | 1.42E+05 | 1.38E+05 | 1.51E+05 | 7.17E+04 | 5.78E+04 | 5.98E+04 | 1.19E+05 | 1.05E+05 | 1.21E+05 | 5.00E+04 | 4.96E+04 | 4.40E+04 | 1.02E+05 | 8.52E+04 | 9.19E+04 | C00548 | -- |
| pme1002 | 1.38E+02 | 1.03E+02 | 1.37E+02 | C_8_H_11_NO | [M+H]+ | L-Tyramine | Alkaloids | Alkaloids | 51-67-2 | 3 | 1.98E+04 | 1.98E+04 | 2.75E+04 | 9.00E+00 | 9.00E+00 | 9.00E+00 | 2.27E+04 | 2.40E+04 | 2.07E+04 | 1.54E+04 | 1.49E+04 | 1.10E+04 | 1.53E+04 | 1.41E+04 | 2.09E+04 | C00483 | ko00350,ko00950,ko01100,ko01110,ko01240 |
| Lmgn001670 | 1.37E+02 | 1.08E+02 | 1.38E+02 | C_7_H_6_O_3_ | [M-H]- | Salicylic acid | Phenolic acids | Phenolic acids | 69-72-7 | 1 | 6.43E+04 | 6.11E+04 | 6.20E+04 | 8.40E+04 | 8.67E+04 | 9.39E+04 | 8.31E+04 | 6.21E+04 | 7.88E+04 | 4.74E+04 | 3.58E+04 | 6.31E+04 | 4.63E+04 | 7.29E+04 | 7.28E+04 | C00805 | ko00360,ko01100,ko01110,ko04075 |
| Lmbn001981 | 1.37E+02 | 9.30E+01 | 1.38E+02 | C_7_H_6_O_3_ | [M-H]- | 2,5-Dihydroxybenzaldehyde | Phenolic acids | Phenolic acids | 1194-98-5 | 2 | 2.24E+06 | 1.69E+06 | 2.04E+06 | 3.96E+05 | 3.62E+05 | 4.13E+05 | 6.70E+05 | 6.73E+05 | 6.13E+05 | 2.52E+05 | 2.80E+05 | 2.19E+05 | 8.08E+05 | 8.70E+05 | 9.35E+05 | C05585 | ko00350,ko01100 |
| mws0749 | 1.37E+02 | 9.30E+01 | 1.38E+02 | C_7_H_6_O_3_ | [M-H]- | 4-Hydroxybenzoic acid | Phenolic acids | Phenolic acids | 99-96-7 | 2 | 2.58E+06 | 2.44E+06 | 2.80E+06 | 6.17E+05 | 5.59E+05 | 5.26E+05 | 9.54E+05 | 8.94E+05 | 9.52E+05 | 3.23E+05 | 3.77E+05 | 3.38E+05 | 1.05E+06 | 1.18E+06 | 1.21E+06 | C00156 | ko00130,ko00790,ko01100,ko01110,ko01240 |
| Hmgn001653 | 1.37E+02 | 9.30E+01 | 1.38E+02 | C_7_H_6_O_3_ | [M-H]- | Protocatechualdehyde | Phenolic acids | Phenolic acids | 139-85-5 | 2 | 1.51E+06 | 1.36E+06 | 1.45E+06 | 2.75E+05 | 3.04E+05 | 2.83E+05 | 5.02E+05 | 4.88E+05 | 4.83E+05 | 1.99E+05 | 2.00E+05 | 1.90E+05 | 6.23E+05 | 6.29E+05 | 7.03E+05 | C16700 | ko00950 |
| mws2368 | 1.37E+02 | 1.19E+02 | 1.38E+02 | C_8_H_10_O_2_ | [M-H]- | Tyrosol | Phenolic acids | Phenolic acids | 501-94-0 | 2 | 1.47E+04 | 1.49E+04 | 1.67E+04 | 1.63E+04 | 1.13E+04 | 1.13E+04 | 1.94E+04 | 1.50E+04 | 1.99E+04 | 5.42E+03 | 5.47E+03 | 6.02E+03 | 1.01E+04 | 1.20E+04 | 1.52E+04 | C06044 | ko00350,ko01100 |
| pme2828 | 1.40E+02 | 9.90E+01 | 1.39E+02 | C_6_H_5_NO_3_ | [M+H]+ | 4-Nitrophenol | Phenolic acids | Phenolic acids | 100-02-7 | 3 | 3.62E+06 | 4.26E+06 | 3.71E+06 | 5.37E+06 | 5.38E+06 | 6.36E+06 | 4.33E+06 | 4.11E+06 | 3.89E+06 | 4.86E+06 | 4.89E+06 | 5.02E+06 | 4.61E+06 | 4.56E+06 | 5.26E+06 | C00870 | -- |
| mws0704 | 1.40E+02 | 7.90E+01 | 1.41E+02 | C_2_H_8_NO_4_P | [M-H]- | O-Phosphorylethanolamine | Alkaloids | Alkaloids | 1071-23-4 | 3 | 9.00E+00 | 9.00E+00 | 9.00E+00 | 2.42E+04 | 2.75E+04 | 3.06E+04 | 4.06E+04 | 4.16E+04 | 4.46E+04 | 1.73E+04 | 2.32E+04 | 2.20E+04 | 2.00E+04 | 2.58E+04 | 2.51E+04 | C00346 | ko00563,ko00564,ko00600,ko01100 |
| pma2987 | 1.42E+02 | 1.24E+02 | 1.41E+02 | C_6_H_11_N_3_O | [M+H]+ | Histidinol | Alkaloids | Alkaloids | 501-28-0 | 3 | 6.73E+06 | 6.35E+06 | 7.67E+06 | 7.94E+06 | 8.67E+06 | 8.98E+06 | 1.01E+07 | 9.61E+06 | 8.56E+06 | 8.74E+06 | 8.27E+06 | 9.02E+06 | 7.91E+06 | 9.27E+06 | 8.26E+06 | C00860 | ko00340,ko01100,ko01110,ko01230 |
| pme3207 | 1.41E+02 | 5.90E+01 | 1.42E+02 | C_6_H_6_O_4_ | [M-H]- | Muconic acid | Organic acids | Organic acids | 1119-72-8 | 3 | 1.41E+06 | 1.50E+06 | 1.46E+06 | 1.82E+06 | 1.64E+06 | 1.73E+06 | 1.25E+06 | 1.21E+06 | 1.19E+06 | 7.68E+05 | 7.23E+05 | 7.58E+05 | 1.22E+06 | 1.30E+06 | 1.33E+06 | C02480 | ko01100 |
| Lmgn003849 | 1.41E+02 | 5.90E+01 | 1.42E+02 | C_8_H_14_O_2_ | [M-H]- | 2-n-Propyl-4-pentenoic acid | Organic acids | Organic acids | 1575-72-0 | 2 | 4.41E+05 | 6.38E+05 | 4.42E+05 | 4.05E+05 | 6.51E+05 | 6.58E+05 | 3.20E+05 | 3.92E+05 | 3.85E+05 | 5.57E+05 | 5.61E+05 | 5.24E+05 | 6.29E+05 | 4.61E+05 | 6.94E+05 | C16648 | -- |
| Lmgn003978 | 1.41E+02 | 5.90E+01 | 1.42E+02 | C_8_H_14_O_2_ | [M-H]- | 2-n-Propyl-2-pentenoic acid | Organic acids | Organic acids | 60218-41-9 | 2 | 5.61E+05 | 6.21E+05 | 6.06E+05 | 6.72E+05 | 6.20E+05 | 6.41E+05 | 4.57E+05 | 4.52E+05 | 4.54E+05 | 4.15E+05 | 4.56E+05 | 5.19E+05 | 5.93E+05 | 5.64E+05 | 6.02E+05 | C16653 | -- |
| pmb0764 | 1.44E+02 | 1.13E+02 | 1.43E+02 | C_6_H_9_NOS | [M+H]+ | 4-Methyl-5-thiazoleethanol | Others | Others | 137-00-8 | 3 | 1.37E+05 | 1.43E+05 | 1.27E+05 | 6.63E+04 | 5.72E+04 | 5.61E+04 | 4.98E+04 | 4.82E+04 | 5.46E+04 | 3.25E+04 | 3.85E+04 | 3.53E+04 | 7.32E+04 | 7.49E+04 | 7.81E+04 | C04294 | ko00730,ko01100,ko01240 |
| Rfmb320 | 1.44E+02 | 8.41E+01 | 1.43E+02 | C_7_H_13_NO_2_ | [M+H]+ | 1-Methylpiperidine-2-carboxylic acid | Organic acids | Organic acids | 7730-87-2 | 3 | 1.36E+05 | 1.31E+05 | 1.52E+05 | 2.43E+04 | 4.68E+04 | 3.99E+04 | 9.86E+04 | 1.04E+05 | 1.29E+05 | 4.81E+04 | 2.43E+04 | 4.37E+04 | 7.90E+04 | 7.40E+04 | 1.02E+05 | -- | -- |
| pmp000966 | 1.44E+02 | 8.41E+01 | 1.43E+02 | C_7_H_13_NO_2_ | [M+H]+ | Stachydrine | Alkaloids | Pyrrole alkaloids | 471-87-4 | 3 | 1.32E+06 | 1.60E+06 | 1.40E+06 | 1.83E+06 | 1.64E+06 | 2.10E+06 | 1.84E+06 | 1.71E+06 | 1.78E+06 | 1.98E+06 | 2.13E+06 | 2.21E+06 | 1.75E+06 | 1.60E+06 | 1.85E+06 | C10172 | -- |
| mws0851 | 1.43E+02 | 1.43E+02 | 1.44E+02 | C_8_H_16_O_2_ | [M-H]- | Valproic Acid | Organic acids | Organic acids | 99-66-1 | 2 | 4.94E+06 | 4.80E+06 | 4.87E+06 | 4.59E+06 | 4.36E+06 | 4.41E+06 | 2.93E+06 | 2.98E+06 | 2.94E+06 | 2.90E+06 | 2.83E+06 | 2.83E+06 | 3.78E+06 | 4.37E+06 | 4.50E+06 | C07185 | -- |
| Lmyn008503 | 1.43E+02 | 1.43E+02 | 1.44E+02 | C_8_H_16_O_2_ | [M-H]- | Octanoic acid | Lipids | Free fatty acids | 124-07-2 | 2 | 5.26E+03 | 7.07E+03 | 7.36E+03 | 7.85E+03 | 6.60E+03 | 7.24E+03 | 4.15E+03 | 4.34E+03 | 3.20E+03 | 4.44E+03 | 5.31E+03 | 2.73E+03 | 7.44E+03 | 6.32E+03 | 6.61E+03 | C06423 | ko00061,ko00785,ko01100,ko01240 |
| MWS3020 | 1.45E+02 | 8.61E+01 | 1.44E+02 | C_7_H_16_N_2_O | [M+H]+ | N-Acetylcadaverine | Alkaloids | Alkaloids | 32343-73-0 | 2 | 3.75E+05 | 3.22E+05 | 2.60E+05 | 3.68E+04 | 4.80E+04 | 2.61E+04 | 1.36E+05 | 1.35E+05 | 1.45E+05 | 2.69E+04 | 3.24E+04 | 3.64E+04 | 1.47E+05 | 1.40E+05 | 1.60E+05 | -- | -- |
| mws0103 | 1.46E+02 | 9.10E+01 | 1.45E+02 | C_9_H_7_NO | [M+H]+ | Indole-3-carboxaldehyde | Alkaloids | Plumerane | 487-89-8 | 1 | 2.76E+05 | 3.01E+05 | 2.48E+05 | 9.14E+04 | 1.01E+05 | 1.00E+05 | 4.33E+05 | 4.01E+05 | 4.37E+05 | 2.27E+05 | 2.19E+05 | 2.37E+05 | 2.40E+05 | 2.45E+05 | 2.76E+05 | C08493 | -- |
| pme0295 | 1.46E+02 | 8.60E+01 | 1.45E+02 | C_6_H_11_NO_3_ | [M+H]+ | 4-Acetamidobutyric acid | Organic acids | Organic acids | 3025-96-5 | 3 | 4.66E+06 | 4.63E+06 | 4.72E+06 | 4.90E+05 | 4.60E+05 | 4.72E+05 | 6.20E+06 | 6.24E+06 | 6.04E+06 | 2.83E+05 | 2.98E+05 | 3.01E+05 | 2.99E+06 | 3.54E+06 | 3.43E+06 | C02946 | ko00330,ko01100 |
| mws0567 | 1.46E+02 | 8.70E+01 | 1.45E+02 | C_5_H_11_N_3_O_2_ | [M+H]+ | 4-Guanidinobutyric acid | Organic acids | Organic acids | 463-00-3 | 1 | 2.53E+07 | 2.52E+07 | 2.49E+07 | 2.19E+06 | 2.13E+06 | 2.20E+06 | 1.25E+07 | 1.24E+07 | 1.26E+07 | 1.87E+06 | 2.08E+06 | 1.92E+06 | 1.06E+07 | 1.08E+07 | 1.17E+07 | C01035 | ko00330,ko01100 |
| Lmgn000224 | 1.45E+02 | 5.70E+01 | 1.46E+02 | C_5_H_6_O_5_ | [M-H]- | 2-Methyl-3-oxosuccinic acid | Organic acids | Organic acids | - | 3 | 1.87E+04 | 2.01E+04 | 1.64E+04 | 1.32E+04 | 1.02E+04 | 1.77E+04 | 1.77E+04 | 3.94E+04 | 1.94E+04 | 1.36E+04 | 1.30E+04 | 3.90E+04 | 3.02E+04 | 1.58E+04 | 1.43E+04 | C06030 | ko00660 |
| pme2380 | 1.45E+02 | 1.01E+02 | 1.46E+02 | C_5_H_6_O_5_ | [M-H]- | α-Ketoglutaric acid | Organic acids | Organic acids | 328-50-7 | 3 | 3.16E+05 | 3.24E+05 | 3.35E+05 | 4.27E+05 | 4.34E+05 | 4.08E+05 | 5.27E+05 | 6.45E+05 | 4.99E+05 | 4.07E+05 | 4.00E+05 | 3.71E+05 | 3.91E+05 | 4.19E+05 | 4.22E+05 | C00026 | ko00020,ko00040,ko00053,ko00220,ko00250,ko00300,ko00310,ko00340,ko00430,ko00630,ko00650,ko00660,ko01100,ko01110,ko01200,ko01210,ko01230,ko01240 |
| mws1012 | 1.47E+02 | 1.03E+02 | 1.46E+02 | C_9_H_6_O_2_ | [M+H]+ | Coumarin | Lignans and Coumarins | Coumarins | 91-64-5 | 3 | 9.74E+03 | 1.13E+04 | 1.71E+04 | 1.02E+04 | 1.25E+04 | 1.54E+04 | 9.72E+03 | 1.27E+04 | 9.44E+03 | 1.80E+04 | 1.84E+04 | 2.36E+04 | 1.57E+04 | 1.13E+04 | 1.49E+04 | C05851 | ko00940,ko01110 |
| mws0924 | 1.45E+02 | 1.01E+02 | 1.46E+02 | C_6_H_10_O_4_ | [M-H]- | 2-Methylglutaric acid | Organic acids | Organic acids | 617-62-9 | 3 | 5.57E+04 | 8.14E+04 | 7.23E+04 | 5.99E+04 | 6.41E+04 | 4.84E+04 | 1.74E+05 | 1.54E+05 | 1.22E+05 | 1.20E+05 | 1.11E+05 | 1.12E+05 | 7.52E+04 | 1.02E+05 | 1.24E+05 | -- | -- |
| Wmzn000227 | 1.45E+02 | 1.09E+02 | 1.46E+02 | C_6_H_10_O_4_ | [M-H]- | 2,2-Dimethylsuccinic acid | Organic acids | Organic acids | 597-43-3 | 1 | 1.14E+05 | 1.03E+05 | 8.78E+04 | 5.63E+04 | 5.42E+04 | 5.35E+04 | 1.71E+05 | 1.58E+05 | 1.30E+05 | 8.16E+04 | 9.89E+04 | 8.34E+04 | 9.41E+04 | 9.52E+04 | 1.00E+05 | -- | -- |
| mws0208 | 1.45E+02 | 1.01E+02 | 1.46E+02 | C_6_H_10_O_4_ | [M-H]- | Adipic Acid | Organic acids | Organic acids | 124-04-9 | 3 | 4.34E+04 | 4.51E+04 | 7.63E+04 | 6.37E+04 | 4.87E+04 | 9.31E+04 | 1.92E+05 | 1.63E+05 | 1.64E+05 | 1.08E+05 | 1.29E+05 | 1.60E+05 | 1.16E+05 | 1.15E+05 | 1.04E+05 | C06104 | ko01100 |
| pme3146 | 1.45E+02 | 1.02E+02 | 1.46E+02 | C_5_H_10_N_2_O_3_ | [M-H]- | β-Ureidoisobutyric acid | Organic acids | Organic acids | 2905-86-4 | 3 | 1.84E+05 | 1.91E+05 | 3.29E+05 | 9.00E+00 | 9.00E+00 | 9.00E+00 | 3.13E+04 | 2.45E+04 | 2.63E+04 | 9.00E+00 | 9.00E+00 | 9.00E+00 | 5.82E+04 | 7.02E+04 | 8.68E+04 | C05100 | -- |
| pme0193 | 1.47E+02 | 8.40E+01 | 1.46E+02 | C_5_H_10_N_2_O_3_ | [M+H]+ | L-Glutamine | Amino acids and derivatives | Amino acids and derivatives | 56-85-9 | 2 | 1.08E+07 | 1.04E+07 | 1.07E+07 | 1.81E+07 | 1.79E+07 | 1.85E+07 | 1.98E+07 | 1.95E+07 | 2.15E+07 | 3.01E+07 | 3.11E+07 | 3.10E+07 | 1.98E+07 | 2.02E+07 | 2.09E+07 | C00064 | ko00220,ko00230,ko00240,ko00250,ko00630,ko00750,ko00910,ko00970,ko01100,ko01230,ko01240,ko02010 |
| pme0026 | 1.47E+02 | 8.40E+01 | 1.46E+02 | C_6_H_14_N_2_O_2_ | [M+H]+ | L-Lysine | Amino acids and derivatives | Amino acids and derivatives | 56-87-1 | 2 | 1.39E+07 | 1.30E+07 | 1.42E+07 | 2.26E+07 | 2.30E+07 | 2.22E+07 | 2.47E+07 | 2.49E+07 | 2.57E+07 | 3.61E+07 | 3.84E+07 | 3.76E+07 | 2.52E+07 | 2.42E+07 | 2.65E+07 | C00047 | ko00300,ko00310,ko00780,ko00960,ko00970,ko01100,ko01110,ko01210,ko01230,ko02010 |
| Zmzn000113 | 1.46E+02 | 1.02E+02 | 1.47E+02 | C_5_H_9_NO_4_ | [M-H]- | L-threo-3-Methylaspartate | Amino acids and derivatives | Amino acids and derivatives | 6061-13-8 | 3 | 2.94E+05 | 2.29E+05 | 3.56E+05 | 4.94E+05 | 4.62E+05 | 5.39E+05 | 1.06E+05 | 1.18E+05 | 8.78E+04 | 4.91E+05 | 5.70E+05 | 5.51E+05 | 3.30E+05 | 3.20E+05 | 3.73E+05 | C03618 | ko00630,ko00660,ko01100,ko01200 |
| mws1050 | 1.48E+02 | 8.80E+01 | 1.47E+02 | C_5_H_9_NO_4_ | [M+H]+ | O-Acetylserine | Amino acids and derivatives | Amino acids and derivatives | 5147-00-2 | 3 | 9.27E+05 | 1.16E+06 | 1.34E+06 | 1.24E+06 | 1.43E+06 | 1.41E+06 | 1.42E+06 | 1.45E+06 | 1.47E+06 | 1.39E+06 | 1.55E+06 | 1.49E+06 | 1.40E+06 | 1.43E+06 | 1.64E+06 | C00979 | ko00270,ko00908,ko00920,ko01100,ko01110,ko01200,ko01230,ko04122 |
| pme0014 | 1.48E+02 | 8.40E+01 | 1.47E+02 | C_5_H_9_NO_4_ | [M+H]+ | L-Glutamic acid | Amino acids and derivatives | Amino acids and derivatives | 56-86-0 | 2 | 2.40E+06 | 2.53E+06 | 2.33E+06 | 3.77E+06 | 3.69E+06 | 3.64E+06 | 3.20E+06 | 3.28E+06 | 3.28E+06 | 4.00E+06 | 3.98E+06 | 4.07E+06 | 3.57E+06 | 3.24E+06 | 3.54E+06 | C00025 | ko00220,ko00250,ko00330,ko00332,ko00340,ko00430,ko00480,ko00524,ko00630,ko00650,ko00660,ko00860,ko00910,ko00970,ko01100,ko01110,ko01200,ko01210,ko01230,ko01240,ko02010 |
| MWSmce608 | 1.48E+02 | 7.40E+01 | 1.47E+02 | C_6_H_13_NO_3_ | [M+H]+ | 4-Hydoxy-L-Isoleucine* | Amino acids and derivatives | Amino acids and derivatives | 781658-23-9 | 1 | 1.03E+07 | 1.03E+07 | 1.04E+07 | 1.72E+07 | 1.65E+07 | 1.69E+07 | 1.51E+07 | 1.54E+07 | 1.60E+07 | 1.83E+07 | 1.82E+07 | 1.87E+07 | 1.43E+07 | 1.40E+07 | 1.59E+07 | -- | -- |
| MWSmce190 | 1.48E+02 | 7.40E+01 | 1.47E+02 | C_6_H_13_NO_3_ | [M+H]+ | (2S,3R,4S)-4-Hydroxyisoleucine* | Amino acids and derivatives | Amino acids and derivatives | 55399-93-4 | 1 | 1.11E+07 | 1.13E+07 | 1.12E+07 | 1.64E+07 | 1.64E+07 | 1.69E+07 | 1.56E+07 | 1.56E+07 | 1.60E+07 | 1.84E+07 | 1.89E+07 | 1.70E+07 | 1.52E+07 | 1.51E+07 | 1.62E+07 | -- | -- |
| pmp001285 | 1.49E+02 | 6.50E+01 | 1.48E+02 | C_8_H_4_O_3_ | [M+H]+ | Phthalic anhydride | Phenolic acids | Phenolic acids | 85-44-9 | 1 | 4.71E+05 | 3.65E+05 | 3.28E+05 | 3.98E+05 | 4.50E+05 | 4.25E+05 | 3.64E+05 | 4.08E+05 | 3.92E+05 | 4.40E+05 | 4.31E+05 | 4.80E+05 | 3.58E+05 | 4.06E+05 | 4.08E+05 | -- | -- |
| Zmyn000230 | 1.47E+02 | 5.70E+01 | 1.48E+02 | C_5_H_8_O_5_ | [M-H]- | 2-Dehydro-3-deoxy-L-arabinonate | Others | Saccharides and Alcohols | - | 1 | 8.29E+05 | 7.76E+05 | 9.54E+05 | 2.75E+05 | 2.65E+05 | 2.96E+05 | 4.10E+05 | 4.58E+05 | 4.11E+05 | 3.30E+05 | 3.61E+05 | 3.75E+05 | 4.17E+05 | 4.93E+05 | 4.64E+05 | C00684 | ko00053,ko01100 |
| MWS3036 | 1.47E+02 | 8.50E+01 | 1.48E+02 | C_5_H_8_O_5_ | [M-H]- | 3-Hydroxyglutaric acid | Organic acids | Organic acids | 638-18-6 | 2 | 2.58E+05 | 2.96E+05 | 3.22E+05 | 1.00E+05 | 9.28E+04 | 1.05E+05 | 1.20E+05 | 1.07E+05 | 1.13E+05 | 1.09E+05 | 1.10E+05 | 1.05E+05 | 1.35E+05 | 1.37E+05 | 1.30E+05 | -- | -- |
| Zmyn000247 | 1.47E+02 | 5.70E+01 | 1.48E+02 | C_5_H_8_O_5_ | [M-H]- | 2-Hydroxyglutaric Acid | Organic acids | Organic acids | 13095-48-2 | 1 | 8.37E+05 | 9.02E+05 | 9.22E+05 | 1.72E+05 | 2.39E+05 | 2.51E+05 | 4.18E+05 | 3.74E+05 | 3.91E+05 | 2.79E+05 | 2.91E+05 | 3.64E+05 | 3.55E+05 | 4.46E+05 | 3.97E+05 | C03196 | ko00310,ko01100 |
| ML10171848 | 1.47E+02 | 8.50E+01 | 1.48E+02 | C_5_H_8_O_5_ | [M-H]- | D-Arabinono-1,4-lactone | Others | Saccharides and Alcohols | 2782-09-4 | 1 | 2.78E+05 | 2.89E+05 | 3.06E+05 | 1.03E+05 | 1.07E+05 | 1.03E+05 | 1.29E+05 | 1.35E+05 | 1.24E+05 | 1.35E+05 | 1.24E+05 | 1.15E+05 | 1.34E+05 | 1.45E+05 | 1.76E+05 | C00652 | ko00053 |
| pmb2826 | 1.47E+02 | 8.71E+01 | 1.48E+02 | C_5_H_8_O_5_ | [M-H]- | L-Citramalic acid | Organic acids | Organic acids | 6236-09-5 | 1 | 5.07E+04 | 5.65E+04 | 3.82E+04 | 5.25E+04 | 6.77E+04 | 6.94E+04 | 7.12E+04 | 1.04E+05 | 6.44E+04 | 5.44E+04 | 2.54E+04 | 6.36E+04 | 5.58E+04 | 6.81E+04 | 6.55E+04 | C02614 | ko00660,ko01100 |
| Lmbn000216 | 1.47E+02 | 5.70E+01 | 1.48E+02 | C_5_H_8_O_5_ | [M-H]- | 3-Methylmalic acid | Organic acids | Organic acids | 152204-30-3 | 1 | 8.18E+05 | 8.21E+05 | 9.55E+05 | 2.80E+05 | 2.52E+05 | 2.52E+05 | 4.16E+05 | 4.03E+05 | 3.91E+05 | 3.17E+05 | 3.51E+05 | 3.98E+05 | 4.33E+05 | 4.94E+05 | 4.54E+05 | C06032 | ko00290,ko00660,ko01100,ko01210,ko01230 |
| mws1024 | 1.47E+02 | 1.29E+02 | 1.48E+02 | C_9_H_8_O_2_ | [M-H]- | p-Coumaraldehyde | Phenolic acids | Phenolic acids | 2538-87-6 | 3 | 6.20E+03 | 6.23E+03 | 5.35E+03 | 9.00E+00 | 9.00E+00 | 9.00E+00 | 2.50E+04 | 2.40E+04 | 2.26E+04 | 9.00E+00 | 9.00E+00 | 9.00E+00 | 8.21E+03 | 4.30E+03 | 9.03E+03 | C05608 | ko00940,ko01100,ko01110 |
| MWS20194 | 1.49E+02 | 1.03E+02 | 1.48E+02 | C_9_H_8_O_2_ | [M+H]+ | Cinnamic acid | Phenolic acids | Phenolic acids | 140-10-3 | 1 | 6.52E+06 | 6.89E+06 | 5.53E+06 | 2.78E+05 | 2.71E+05 | 2.90E+05 | 1.93E+06 | 1.94E+06 | 2.11E+06 | 1.24E+05 | 1.41E+05 | 1.30E+05 | 2.54E+06 | 2.35E+06 | 3.04E+06 | C00423 | ko00130,ko00360,ko00940,ko00998,ko01100,ko01110 |
| pme2987 | 1.47E+02 | 1.19E+02 | 1.48E+02 | C_9_H_8_O_2_ | [M-H]- | 3,4-Dihydrocoumarin | Lignans and Coumarins | Coumarins | 119-84-6 | 2 | 2.84E+04 | 3.07E+04 | 3.18E+04 | 9.00E+00 | 9.00E+00 | 9.00E+00 | 8.99E+03 | 7.34E+03 | 1.03E+04 | 9.00E+00 | 9.00E+00 | 9.00E+00 | 1.17E+04 | 1.49E+04 | 1.23E+04 | C02274 | -- |
| pme3154 | 1.47E+02 | 5.90E+01 | 1.48E+02 | C_6_H_12_O_4_ | [M-H]- | Mevalonic acid | Organic acids | Organic acids | 150-97-0 | 2 | 2.44E+05 | 2.32E+05 | 3.07E+05 | 9.00E+00 | 9.00E+00 | 9.00E+00 | 1.96E+05 | 1.46E+05 | 2.12E+05 | 9.00E+00 | 9.00E+00 | 9.00E+00 | 1.02E+05 | 1.18E+05 | 1.04E+05 | C00418 | ko00900,ko01100,ko01110 |
| Lmgp000796 | 1.50E+02 | 6.10E+01 | 1.49E+02 | C_8_H_7_NO_2_ | [M+H]+ | 4-Hydroxymandelonitrile | Alkaloids | Phenolamine | 13093-65-7 | 2 | 2.66E+06 | 2.67E+06 | 2.84E+06 | 2.12E+06 | 2.10E+06 | 2.05E+06 | 3.81E+06 | 4.13E+06 | 4.04E+06 | 6.66E+06 | 6.89E+06 | 6.87E+06 | 3.73E+06 | 3.69E+06 | 4.14E+06 | C00650 | -- |
| pme1210 | 1.50E+02 | 6.10E+01 | 1.49E+02 | C_5_H_11_NO_2_S | [M+H]+ | L-Methionine | Amino acids and derivatives | Amino acids and derivatives | 63-68-3 | 2 | 2.83E+06 | 2.86E+06 | 3.09E+06 | 2.08E+06 | 2.21E+06 | 2.22E+06 | 4.32E+06 | 4.38E+06 | 4.68E+06 | 7.16E+06 | 7.27E+06 | 6.97E+06 | 3.72E+06 | 3.59E+06 | 4.68E+06 | C00073 | ko00270,ko00966,ko00970,ko01100,ko01110,ko01210,ko01230,ko01240 |
| mws0847 | 1.50E+02 | 1.33E+02 | 1.49E+02 | C_6_H_7_N_5_ | [M+H]+ | 1-Methyladenine | Nucleotides and derivatives | Nucleotides and derivatives | 5142-22-3 | 3 | 4.83E+04 | 7.28E+04 | 4.93E+04 | 3.59E+04 | 4.47E+04 | 4.55E+04 | 8.33E+04 | 7.42E+04 | 9.89E+04 | 1.39E+05 | 1.21E+05 | 1.47E+05 | 6.56E+04 | 5.16E+04 | 9.28E+04 | C02216 | -- |
| mws0489 | 1.49E+02 | 7.70E+01 | 1.50E+02 | C_8_H_6_O_3_ | [M-H]- | Benzoylformic acid | Organic acids | Organic acids | 611-73-4 | 3 | 1.28E+05 | 1.34E+05 | 9.49E+04 | 5.13E+04 | 4.20E+04 | 5.57E+04 | 4.23E+04 | 6.86E+04 | 6.73E+04 | 9.00E+00 | 9.00E+00 | 9.00E+00 | 6.19E+04 | 7.56E+04 | 6.73E+04 | C02137 | ko00360,ko01100 |
| mws1499 | 1.49E+02 | 5.90E+01 | 1.50E+02 | C_5_H_10_O_5_ | [M-H]- | D-Arabinose | Others | Saccharides and Alcohols | 10323-20-3 | 3 | 2.15E+05 | 2.24E+05 | 2.33E+05 | 3.70E+05 | 3.70E+05 | 3.81E+05 | 2.65E+05 | 2.58E+05 | 2.14E+05 | 2.62E+05 | 2.18E+05 | 2.00E+05 | 2.40E+05 | 2.34E+05 | 3.00E+05 | C00216 | ko00040,ko00053 |
| mws0921 | 1.49E+02 | 1.31E+02 | 1.50E+02 | C_9_H_10_O_2_ | [M-H]- | p-Coumaryl alcohol | Phenolic acids | Phenolic acids | 3690-05-9 | 3 | 2.09E+04 | 1.30E+04 | 2.08E+04 | 5.85E+03 | 3.56E+03 | 1.59E+03 | 4.82E+04 | 4.87E+04 | 6.93E+04 | 5.85E+03 | 4.31E+03 | 5.29E+03 | 1.86E+04 | 1.47E+04 | 1.70E+04 | C02646 | ko00940,ko01100,ko01110 |
| mws0008 | 1.49E+02 | 1.05E+02 | 1.50E+02 | C_9_H_10_O_2_ | [M-H]- | Hydrocinnamic acid | Phenolic acids | Phenolic acids | 501-52-0 | 3 | 6.48E+04 | 8.50E+04 | 6.53E+04 | 9.00E+00 | 9.00E+00 | 9.00E+00 | 2.67E+04 | 1.57E+04 | 2.57E+04 | 9.00E+00 | 9.00E+00 | 9.00E+00 | 2.29E+04 | 2.83E+04 | 2.92E+04 | C05629 | ko00360,ko01100 |
| MWSmce557 | 1.51E+02 | 1.09E+02 | 1.50E+02 | C_8_H_10_N_2_O | [M+H]+ | 2-Acetyl-3-ethylpyrazine | Alkaloids | Alkaloids | 32974-92-8 | 2 | 3.01E+04 | 2.64E+04 | 3.06E+04 | 9.00E+00 | 9.00E+00 | 9.00E+00 | 9.00E+00 | 9.00E+00 | 9.00E+00 | 9.00E+00 | 9.00E+00 | 9.00E+00 | 8.52E+03 | 1.01E+04 | 1.35E+04 | -- | -- |
| Lmxn006423 | 1.50E+02 | 1.22E+02 | 1.51E+02 | C_7_H_5_NOS | [M-H]- | 2(3H)-Benzothiazolone | Alkaloids | Alkaloids | 934-34-9 | 1 | 1.00E+04 | 1.07E+04 | 1.38E+04 | 1.61E+04 | 1.66E+04 | 1.15E+04 | 2.08E+04 | 2.15E+04 | 1.70E+04 | 1.28E+04 | 1.46E+04 | 1.86E+04 | 1.52E+04 | 1.76E+04 | 1.46E+04 | -- | -- |
| pme0183 | 1.52E+02 | 1.35E+02 | 1.51E+02 | C_5_H_5_N_5_O | [M+H]+ | Isoguanine | Nucleotides and derivatives | Nucleotides and derivatives | 3373-53-3 | 3 | 4.21E+05 | 5.12E+05 | 3.31E+05 | 4.25E+04 | 3.43E+04 | 2.21E+04 | 1.21E+05 | 1.18E+05 | 1.04E+05 | 5.73E+04 | 8.55E+04 | 6.66E+04 | 1.48E+05 | 9.84E+04 | 1.28E+05 | -- | -- |
| pme1109 | 1.52E+02 | 1.35E+02 | 1.51E+02 | C_5_H_5_N_5_O | [M+H]+ | Guanine | Nucleotides and derivatives | Nucleotides and derivatives | 73-40-5 | 2 | 5.57E+06 | 5.80E+06 | 4.96E+06 | 2.16E+05 | 2.41E+05 | 2.19E+05 | 9.24E+05 | 8.29E+05 | 8.38E+05 | 1.19E+05 | 1.28E+05 | 1.22E+05 | 1.92E+06 | 1.67E+06 | 2.15E+06 | C00242 | ko00230,ko01100 |
| pme0256 | 1.51E+02 | 1.51E+02 | 1.52E+02 | C_5_H_4_N_4_O_2_ | [M-H]- | Xanthine | Nucleotides and derivatives | Nucleotides and derivatives | 69-89-6 | 1 | 2.43E+06 | 2.38E+06 | 2.95E+06 | 9.00E+00 | 9.00E+00 | 9.00E+00 | 1.43E+05 | 1.31E+05 | 1.42E+05 | 9.00E+00 | 9.00E+00 | 9.00E+00 | 5.56E+05 | 7.57E+05 | 8.44E+05 | C00385 | ko00230,ko00232,ko01100,ko01110 |
| MWS2984 | 1.53E+02 | 1.36E+02 | 1.52E+02 | C_4_H_4_N_6_O | [M+H]+ | 8-Azaguanine | Nucleotides and derivatives | Nucleotides and derivatives | 134-58-7 | 2 | 1.80E+06 | 1.67E+06 | 1.69E+06 | 3.18E+04 | 3.03E+04 | 3.05E+04 | 1.42E+05 | 1.29E+05 | 1.46E+05 | 1.84E+04 | 2.13E+04 | 1.82E+04 | 5.69E+05 | 4.80E+05 | 6.39E+05 | -- | -- |
| mws0182 | 1.51E+02 | 1.07E+02 | 1.52E+02 | C_8_H_8_O_3_ | [M-H]- | p-Hydroxyphenyl acetic acid | Phenolic acids | Phenolic acids | 156-38-7 | 3 | 1.54E+05 | 1.48E+05 | 1.66E+05 | 9.00E+00 | 9.00E+00 | 9.00E+00 | 2.04E+05 | 1.77E+05 | 1.72E+05 | 9.00E+00 | 9.00E+00 | 9.00E+00 | 9.52E+04 | 1.09E+05 | 9.90E+04 | C00642 | ko00350,ko00360,ko01100 |
| mws0458 | 1.51E+02 | 1.36E+02 | 1.52E+02 | C_8_H_8_O_3_ | [M-H]- | Vanillin | Phenolic acids | Phenolic acids | 121-33-5 | 3 | 1.86E+06 | 1.82E+06 | 2.03E+06 | 1.07E+06 | 1.04E+06 | 1.05E+06 | 1.51E+06 | 1.52E+06 | 1.58E+06 | 9.06E+05 | 8.48E+05 | 8.36E+05 | 1.36E+06 | 1.39E+06 | 1.53E+06 | C00755 | ko00998,ko01100,ko01110 |
| pme2362 | 1.51E+02 | 1.07E+02 | 1.52E+02 | C_8_H_8_O_3_ | [M-H]- | Mandelic acid | Phenolic acids | Phenolic acids | 90-64-2 | 3 | 1.59E+05 | 1.45E+05 | 1.52E+05 | 2.01E+04 | 1.35E+04 | 7.00E+03 | 1.54E+05 | 1.27E+05 | 1.11E+05 | 2.42E+04 | 2.58E+04 | 2.51E+04 | 7.16E+04 | 8.02E+04 | 7.23E+04 | C01984 | -- |
| mws0437 | 1.51E+02 | 7.10E+01 | 1.52E+02 | C_5_H_12_O_5_ | [M-H]- | D-Arabitol | Others | Saccharides and Alcohols | 488-82-4 | 3 | 3.32E+05 | 3.10E+05 | 3.13E+05 | 4.16E+05 | 4.21E+05 | 4.58E+05 | 2.33E+05 | 2.05E+05 | 2.17E+05 | 2.39E+05 | 2.73E+05 | 2.23E+05 | 2.79E+05 | 3.06E+05 | 2.99E+05 | C01904 | ko00040,ko01100 |
| mws0213 | 1.51E+02 | 7.10E+01 | 1.52E+02 | C_5_H_12_O_5_ | [M-H]- | Ribitol | Others | Saccharides and Alcohols | 488-81-3 | 3 | 5.07E+05 | 5.06E+05 | 4.83E+05 | 5.90E+05 | 6.43E+05 | 6.58E+05 | 3.38E+05 | 3.24E+05 | 3.27E+05 | 3.83E+05 | 3.67E+05 | 3.55E+05 | 4.40E+05 | 4.45E+05 | 4.67E+05 | C00474 | ko00040,ko00740,ko01100 |
| pme0513 | 1.51E+02 | 5.90E+01 | 1.52E+02 | C_5_H_12_O_5_ | [M-H]- | Xylitol | Others | Saccharides and Alcohols | 87-99-0 | 3 | 1.88E+05 | 2.13E+05 | 2.14E+05 | 2.77E+05 | 2.80E+05 | 2.88E+05 | 1.49E+05 | 1.86E+05 | 1.81E+05 | 1.58E+05 | 1.70E+05 | 1.61E+05 | 2.01E+05 | 1.99E+05 | 2.23E+05 | C00379 | ko00040,ko01100,ko02010 |
| mws0438 | 1.51E+02 | 5.90E+01 | 1.52E+02 | C_5_H_12_O_5_ | [M-H]- | L-Arabitol | Others | Saccharides and Alcohols | 7643-75-6 | 3 | 3.01E+05 | 2.86E+05 | 3.21E+05 | 3.55E+05 | 3.88E+05 | 3.81E+05 | 2.08E+05 | 2.11E+05 | 2.20E+05 | 2.18E+05 | 2.30E+05 | 2.23E+05 | 2.79E+05 | 2.79E+05 | 2.67E+05 | C00532 | ko00040,ko01100 |
| mws0444 | 1.52E+02 | 1.08E+02 | 1.53E+02 | C_7_H_7_NO_3_ | [M-H]- | 3-Aminosalicylic acid | Phenolic acids | Phenolic acids | 570-23-0 | 3 | 2.16E+05 | 1.91E+05 | 2.39E+05 | 9.00E+00 | 9.00E+00 | 9.00E+00 | 6.01E+04 | 4.94E+04 | 4.02E+04 | 9.00E+00 | 9.00E+00 | 9.00E+00 | 6.42E+04 | 8.11E+04 | 7.09E+04 | -- | -- |
| mws0596 | 1.54E+02 | 1.36E+02 | 1.53E+02 | C_7_H_7_NO_3_ | [M+H]+ | 3-Hydroxyanthranilic acid | Alkaloids | Phenolamine | 548-93-6 | 3 | 1.47E+05 | 1.59E+05 | 1.61E+05 | 9.00E+00 | 9.00E+00 | 9.00E+00 | 9.00E+00 | 9.00E+00 | 9.00E+00 | 9.00E+00 | 9.00E+00 | 9.00E+00 | 4.95E+04 | 5.14E+04 | 7.53E+04 | C00632 | ko00380,ko01100,ko01240 |
| Hmlp000935 | 1.54E+02 | 1.37E+02 | 1.53E+02 | C_8_H_11_NO_2_ | [M+H]+ | Vanillylamine | Alkaloids | Phenolamine | 1196-92-5 | 2 | 1.40E+05 | 1.46E+05 | 1.30E+05 | 9.00E+00 | 9.00E+00 | 9.00E+00 | 1.18E+04 | 1.31E+04 | 1.41E+04 | 9.00E+00 | 9.00E+00 | 9.00E+00 | 4.92E+04 | 3.48E+04 | 5.27E+04 | C16666 | ko00998,ko01100,ko01110 |
| mws0180 | 1.53E+02 | 1.09E+02 | 1.54E+02 | C_7_H_6_O_4_ | [M-H]- | 2,5-Dihydroxybenzoic acid; Gentisic Acid* | Phenolic acids | Phenolic acids | 490-79-9 | 2 | 8.41E+05 | 9.41E+05 | 9.74E+05 | 3.49E+05 | 3.19E+05 | 4.32E+05 | 2.21E+06 | 1.94E+06 | 2.20E+06 | 4.95E+04 | 7.41E+04 | 7.31E+04 | 8.51E+05 | 8.56E+05 | 8.50E+05 | C00628 | ko00350,ko01100 |
| mws0183 | 1.53E+02 | 1.09E+02 | 1.54E+02 | C_7_H_6_O_4_ | [M-H]- | 3,4-Dihydroxybenzoic acid (Protocatechuic acid)* | Phenolic acids | Phenolic acids | 99-50-3 | 2 | 1.35E+06 | 1.48E+06 | 1.56E+06 | 5.54E+05 | 4.87E+05 | 7.51E+05 | 3.48E+06 | 3.27E+06 | 3.97E+06 | 9.37E+04 | 1.26E+05 | 1.13E+05 | 1.41E+06 | 1.52E+06 | 1.50E+06 | C00230 | ko01100,ko01110 |
| mws0639 | 1.53E+02 | 1.09E+02 | 1.54E+02 | C_7_H_6_O_4_ | [M-H]- | 2,3-Dihydroxybenzoic Acid* | Phenolic acids | Phenolic acids | 303-38-8 | 2 | 1.47E+06 | 1.58E+06 | 2.44E+06 | 3.20E+05 | 4.26E+05 | 4.32E+05 | 9.00E+00 | 9.00E+00 | 9.00E+00 | 1.35E+05 | 1.41E+05 | 7.91E+04 | 5.88E+05 | 6.68E+05 | 6.50E+05 | C00196 | ko01110 |
| mws0254 | 1.56E+02 | 1.10E+02 | 1.55E+02 | C_6_H_9_N_3_O_2_ | [M+H]+ | L-Histidine | Amino acids and derivatives | Amino acids and derivatives | 71-00-1 | 1 | 5.67E+06 | 5.30E+06 | 5.61E+06 | 9.50E+06 | 9.57E+06 | 9.48E+06 | 1.33E+07 | 1.36E+07 | 1.40E+07 | 1.73E+07 | 1.80E+07 | 1.74E+07 | 1.16E+07 | 1.17E+07 | 1.17E+07 | C00135 | ko00340,ko00410,ko00970,ko01100,ko01110,ko01230,ko02010 |
| MWSslk051 | 1.56E+02 | 8.41E+01 | 1.55E+02 | C_8_H_13_NO_2_ | [M+H]+ | Scopine | Alkaloids | Tropan alkaloids | 498-45-3 | 3 | 3.58E+04 | 4.09E+04 | 3.39E+04 | 9.66E+03 | 8.63E+03 | 5.27E+03 | 2.05E+04 | 1.93E+04 | 1.95E+04 | 1.52E+04 | 7.54E+03 | 8.35E+03 | 1.48E+04 | 2.27E+04 | 2.07E+04 | -- | -- |
| pmb0819 | 1.57E+02 | 1.30E+02 | 1.56E+02 | C_10_H_8_N_2_ | [M+H]+ | 3-Indoleacetonitrile | Alkaloids | Plumerane | 771-51-7 | 3 | 4.72E+04 | 4.77E+04 | 3.21E+04 | 1.00E+05 | 1.10E+05 | 1.07E+05 | 1.71E+05 | 1.75E+05 | 1.69E+05 | 2.00E+05 | 1.86E+05 | 1.91E+05 | 1.40E+05 | 1.06E+05 | 1.46E+05 | C02938 | ko00380,ko01100 |
| pme2867 | 1.58E+02 | 8.30E+01 | 1.57E+02 | C_7_H_11_NO_3_ | [M+H]+ | 3-Methylcrotonyl-L-glycine | Amino acids and derivatives | Amino acids and derivatives | 33008-07-0 | 3 | 7.27E+04 | 6.44E+04 | 9.22E+04 | 9.00E+00 | 9.00E+00 | 9.00E+00 | 9.00E+00 | 9.00E+00 | 9.00E+00 | 9.00E+00 | 9.00E+00 | 9.00E+00 | 2.34E+04 | 3.33E+04 | 3.05E+04 | C20828 | -- |
| MWS1789 | 1.59E+02 | 1.03E+02 | 1.58E+02 | C_9_H_18_O_2_ | [M+H]+ | Butyl 3-methylbutanoate | Organic acids | Organic acids | 109-19-3 | 2 | 1.46E+04 | 1.38E+04 | 1.59E+04 | 4.11E+04 | 2.34E+04 | 3.84E+04 | 2.08E+04 | 3.05E+04 | 1.99E+04 | 2.64E+04 | 3.05E+04 | 3.31E+04 | 2.56E+04 | 2.95E+04 | 2.92E+04 | -- | -- |
| Lmbn001467 | 1.58E+02 | 1.16E+02 | 1.59E+02 | C_7_H_13_NO_3_ | [M-H]- | 5-Acetamidopentanoic Acid | Organic acids | Organic acids | 1072-10-2 | 1 | 8.84E+04 | 8.88E+04 | 8.11E+04 | 1.05E+05 | 1.19E+05 | 1.20E+05 | 1.94E+05 | 1.73E+05 | 2.04E+05 | 2.31E+05 | 2.58E+05 | 2.37E+05 | 1.71E+05 | 1.59E+05 | 1.64E+05 | C03087 | ko00310,ko01100 |
| mws0987 | 1.61E+02 | 1.15E+02 | 1.60E+02 | C_10_H_8_O_2_ | [M+H]+ | 6-MethylCoumarin | Lignans and Coumarins | Coumarins | 92-48-8 | 3 | 5.21E+03 | 4.57E+03 | 7.93E+03 | 4.05E+03 | 6.12E+03 | 5.43E+03 | 5.38E+03 | 3.68E+03 | 4.74E+03 | 5.31E+03 | 6.41E+03 | 6.44E+03 | 3.98E+03 | 2.67E+03 | 6.96E+03 | -- | -- |
| Zmjn001813 | 1.59E+02 | 9.71E+01 | 1.60E+02 | C_7_H_12_O_4_ | [M-H]- | Pimelic acid | Organic acids | Organic acids | 111-16-0 | 3 | 6.43E+04 | 7.11E+04 | 6.73E+04 | 2.48E+04 | 3.55E+04 | 4.18E+04 | 2.80E+04 | 2.34E+04 | 2.19E+04 | 1.56E+04 | 1.23E+04 | 1.02E+04 | 3.22E+04 | 3.39E+04 | 3.17E+04 | C02656 | ko00780,ko01100,ko01240 |
| Lmbn002072 | 1.59E+02 | 9.71E+01 | 1.60E+02 | C_7_H_12_O_4_ | [M-H]- | 2-Propylsuccinic acid | Organic acids | Organic acids | 618-57-5 | 3 | 4.45E+04 | 5.24E+04 | 4.46E+04 | 2.69E+04 | 2.44E+04 | 2.91E+04 | 1.68E+04 | 2.30E+04 | 2.00E+04 | 1.37E+04 | 8.17E+03 | 1.72E+04 | 2.22E+04 | 2.35E+04 | 2.46E+04 | C16657 | -- |
| pme0128 | 1.59E+02 | 8.80E+01 | 1.60E+02 | C_6_H_12_N_2_O_3_ | [M-H]- | L-Alanyl-L-Alanine | Amino acids and derivatives | Amino acids and derivatives | 1948-31-8 | 3 | 3.90E+04 | 3.55E+04 | 4.90E+04 | 9.00E+00 | 9.00E+00 | 9.00E+00 | 9.00E+00 | 9.00E+00 | 9.00E+00 | 9.00E+00 | 9.00E+00 | 9.00E+00 | 9.67E+03 | 1.19E+04 | 9.51E+03 | -- | -- |
| mws0005 | 1.61E+02 | 1.44E+02 | 1.60E+02 | C_10_H_12_N_2_ | [M+H]+ | Tryptamine | Alkaloids | Plumerane | 61-54-1 | 2 | 7.76E+06 | 5.82E+06 | 1.29E+07 | 2.86E+04 | 3.44E+04 | 2.97E+04 | 2.11E+05 | 1.78E+05 | 2.27E+05 | 7.83E+04 | 8.89E+04 | 6.61E+04 | 2.56E+06 | 2.52E+06 | 2.76E+06 | C00398 | ko00380,ko00901,ko01100,ko01110 |
| mws1417 | 1.60E+02 | 1.16E+02 | 1.61E+02 | C_9_H_7_NO_2_ | [M-H]- | Indole-3-carboxylic acid* | Alkaloids | Plumerane | 771-50-6 | 2 | 7.13E+05 | 7.98E+05 | 7.74E+05 | 8.56E+04 | 9.40E+04 | 8.09E+04 | 3.18E+05 | 3.27E+05 | 3.36E+05 | 9.40E+04 | 1.29E+05 | 1.23E+05 | 3.13E+05 | 3.41E+05 | 3.58E+05 | C19837 | -- |
| mws0102 | 1.60E+02 | 1.16E+02 | 1.61E+02 | C_9_H_7_NO_2_ | [M-H]- | Indole-5-carboxylic acid* | Alkaloids | Plumerane | 1670-81-1 | 2 | 6.80E+05 | 6.26E+05 | 6.48E+05 | 7.87E+04 | 1.06E+05 | 7.91E+04 | 3.94E+05 | 2.98E+05 | 2.95E+05 | 9.46E+04 | 1.22E+05 | 1.02E+05 | 3.02E+05 | 3.27E+05 | 3.35E+05 | -- | -- |
| mws1346 | 1.62E+02 | 9.80E+01 | 1.61E+02 | C_6_H_11_NO_4_ | [M+H]+ | DL-2-Aminoadipic acid | Alkaloids | Alkaloids | 542-32-5 | 3 | 6.73E+05 | 7.57E+05 | 6.71E+05 | 1.80E+05 | 1.72E+05 | 1.89E+05 | 3.33E+05 | 3.89E+05 | 3.84E+05 | 1.79E+05 | 1.25E+05 | 1.67E+05 | 2.92E+05 | 3.45E+05 | 3.29E+05 | C00956 | ko00300,ko00310,ko00311,ko01100,ko01110,ko01210,ko01230 |
| pme3382 | 1.60E+02 | 9.80E+01 | 1.61E+02 | C_6_H_11_NO_4_ | [M-H]- | N-Acetyl-L-threonine | Amino acids and derivatives | Amino acids and derivatives | 17093-74-2 | 3 | 2.78E+05 | 2.64E+05 | 3.10E+05 | 4.48E+04 | 5.02E+04 | 5.52E+04 | 8.64E+04 | 8.33E+04 | 8.22E+04 | 3.75E+04 | 4.36E+04 | 3.00E+04 | 1.11E+05 | 1.06E+05 | 1.16E+05 | -- | -- |
| mws1320 | 1.60E+02 | 1.30E+02 | 1.61E+02 | C_10_H_11_NO | [M-H]- | Tryptophol | Alkaloids | Plumerane | 526-55-6 | 1 | 8.73E+03 | 9.93E+03 | 1.26E+04 | 9.00E+00 | 9.00E+00 | 9.00E+00 | 1.95E+04 | 1.93E+04 | 1.93E+04 | 9.00E+00 | 9.00E+00 | 9.00E+00 | 6.15E+03 | 6.23E+03 | 8.97E+03 | C00955 | ko00380 |
| Hmqn002118 | 1.61E+02 | 1.33E+02 | 1.62E+02 | C_9_H_6_O_3_ | [M-H]- | 7-Hydroxycoumarin | Lignans and Coumarins | Lignans | 93-35-6 | 1 | 2.73E+06 | 2.80E+06 | 3.57E+06 | 1.30E+07 | 1.36E+07 | 1.20E+07 | 1.08E+07 | 1.11E+07 | 1.11E+07 | 8.80E+06 | 9.08E+06 | 9.43E+06 | 8.24E+06 | 9.00E+06 | 9.26E+06 | C09315 | ko00940,ko01110 |
| MWS0559 | 1.61E+02 | 7.10E+01 | 1.62E+02 | C_6_H_10_O_5_ | [M-H]- | 1,6-anhydro-β-D-glucose | Others | Saccharides and Alcohols | 498-07-7 | 2 | 2.71E+05 | 2.19E+05 | 2.68E+05 | 3.22E+05 | 3.89E+05 | 3.92E+05 | 1.92E+05 | 1.29E+05 | 1.74E+05 | 1.56E+05 | 1.46E+05 | 1.43E+05 | 2.52E+05 | 2.25E+05 | 2.72E+05 | -- | -- |
| pme2914 | 1.61E+02 | 9.90E+01 | 1.62E+02 | C_6_H_10_O_5_ | [M-H]- | 3-Hydroxy-3-methylpentane-1,5-dioic acid | Amino acids and derivatives | Amino acids and derivatives | 503-49-1 | 1 | 9.76E+06 | 9.50E+06 | 1.06E+07 | 5.70E+06 | 5.61E+06 | 5.69E+06 | 4.73E+06 | 4.47E+06 | 5.20E+06 | 4.57E+06 | 4.76E+06 | 4.90E+06 | 5.98E+06 | 6.48E+06 | 6.92E+06 | C03761 | -- |
| Lmqp008175 | 1.63E+02 | 1.03E+02 | 1.62E+02 | C_10_H_10_O_2_ | [M+H]+ | Methyl Cinnamate | Phenolic acids | Phenolic acids | 103-26-4 | 2 | 1.99E+05 | 2.15E+05 | 3.57E+05 | 1.81E+06 | 1.34E+06 | 1.63E+06 | 4.93E+05 | 4.51E+05 | 6.51E+05 | 1.26E+06 | 1.19E+06 | 1.08E+06 | 9.66E+05 | 9.06E+05 | 9.06E+05 | C06358 | -- |
| pmf0440 | 1.63E+02 | 9.11E+01 | 1.62E+02 | C_10_H_10_O_2_ | [M+H]+ | 4-MethoxycinnaMaldehyde | Phenolic acids | Phenolic acids | 1963-36-6 | 3 | 8.78E+04 | 9.30E+04 | 7.36E+04 | 1.08E+05 | 8.51E+04 | 1.06E+05 | 9.50E+04 | 7.82E+04 | 8.51E+04 | 1.04E+05 | 1.18E+05 | 1.17E+05 | 8.10E+04 | 8.38E+04 | 1.05E+05 | C10475 | -- |
| pme2758 | 1.62E+02 | 1.44E+02 | 1.63E+02 | C_5_H_9_NO_5_ | [M-H]- | 4-Hydroxy-L-glutamic acid | Amino acids and derivatives | Amino acids and derivatives | 3913-68-6 | 3 | 2.14E+04 | 1.89E+04 | 1.44E+04 | 1.41E+04 | 2.44E+04 | 1.62E+04 | 2.15E+04 | 1.37E+04 | 2.14E+04 | 2.79E+04 | 2.19E+04 | 2.18E+04 | 1.47E+04 | 2.31E+04 | 2.10E+04 | C03079 | -- |
| Lmbp000123 | 1.64E+02 | 5.61E+01 | 1.63E+02 | C_6_H_13_NO_2_S | [M+H]+ | L-Homomethionine | Amino acids and derivatives | Amino acids and derivatives | 25148-30-5 | 3 | 4.85E+04 | 3.91E+04 | 3.02E+04 | 2.09E+05 | 1.79E+05 | 1.38E+05 | 7.75E+04 | 7.65E+04 | 8.78E+04 | 5.12E+05 | 4.68E+05 | 5.36E+05 | 1.98E+05 | 1.92E+05 | 1.74E+05 | C17213 | ko00966,ko01110,ko01210 |
| pme1439 | 1.65E+02 | 1.19E+02 | 1.64E+02 | C_9_H_8_O_3_ | [M+H]+ | p-Coumaric acid | Phenolic acids | Phenolic acids | 501-98-4 | 3 | 2.81E+05 | 2.97E+05 | 2.43E+05 | 8.58E+04 | 1.04E+05 | 7.83E+04 | 4.03E+05 | 3.50E+05 | 4.18E+05 | 9.96E+04 | 1.55E+05 | 7.57E+04 | 2.26E+05 | 2.14E+05 | 2.55E+05 | C00811 | ko00130,ko00350,ko00940,ko00950,ko00998,ko01100,ko01110 |
| mws0159 | 1.63E+02 | 9.10E+01 | 1.64E+02 | C_9_H_8_O_3_ | [M-H]- | Phenylpyruvic acid | Organic acids | Organic acids | 156-06-9 | 2 | 6.79E+04 | 7.80E+04 | 9.49E+04 | 2.14E+05 | 2.02E+05 | 2.08E+05 | 2.96E+05 | 2.79E+05 | 2.62E+05 | 1.44E+05 | 1.73E+05 | 1.70E+05 | 1.87E+05 | 1.85E+05 | 1.46E+05 | C00166 | ko00360,ko00400,ko00960,ko01100,ko01110,ko01210,ko01230 |
| Lmmn001643 | 1.63E+02 | 1.19E+02 | 1.64E+02 | C_9_H_8_O_3_ | [M-H]- | 2-Hydroxycinnamic acid | Phenolic acids | Phenolic acids | 583-17-5 | 1 | 4.24E+06 | 4.12E+06 | 4.16E+06 | 1.24E+06 | 1.14E+06 | 1.11E+06 | 5.71E+06 | 7.42E+06 | 7.07E+06 | 9.25E+05 | 1.01E+06 | 9.71E+05 | 3.02E+06 | 3.46E+06 | 3.38E+06 | C01772 | ko00360,ko00940,ko01100,ko01110 |
| Lmmn004032 | 1.63E+02 | 1.48E+02 | 1.64E+02 | C_11_H_16_O | [M-H]- | Cis-Jasmone | Others | Others | 488-10-8 | 2 | 1.13E+04 | 1.19E+04 | 8.36E+03 | 2.20E+04 | 2.27E+04 | 1.40E+04 | 1.60E+04 | 1.36E+04 | 2.01E+04 | 5.25E+03 | 5.82E+03 | 3.41E+03 | 1.11E+04 | 1.03E+04 | 1.54E+04 | C08490 | -- |
| pme3083 | 1.64E+02 | 1.20E+02 | 1.65E+02 | C_8_H_7_NO_3_ | [M-H]- | 2-(Formylamino)benzoic acid | Phenolic acids | Phenolic acids | 3342-77-6 | 3 | 6.63E+05 | 6.58E+05 | 6.25E+05 | 1.79E+05 | 2.04E+05 | 1.93E+05 | 9.48E+05 | 9.55E+05 | 9.85E+05 | 1.46E+05 | 1.68E+05 | 1.77E+05 | 4.87E+05 | 5.11E+05 | 5.26E+05 | C05653 | ko00380,ko01100 |
| pme2617 | 1.66E+02 | 1.02E+02 | 1.65E+02 | C_5_H_11_NO_3_S | [M+H]+ | L-Methionine Sulfoxide | Amino acids and derivatives | Amino acids and derivatives | 3226-65-1 | 3 | 2.29E+06 | 2.27E+06 | 2.35E+06 | 2.95E+06 | 2.69E+06 | 2.58E+06 | 2.95E+06 | 2.90E+06 | 2.86E+06 | 3.38E+06 | 3.22E+06 | 3.42E+06 | 2.81E+06 | 2.67E+06 | 2.93E+06 | C02989 | ko00270 |
| MWS4525 | 1.66E+02 | 1.24E+02 | 1.65E+02 | C_6_H_7_N_5_O | [M+H]+ | 6-O-methylguanine | Nucleotides and derivatives | Nucleotides and derivatives | 20535-83-5 | 1 | 7.41E+04 | 6.82E+04 | 8.51E+04 | 1.01E+05 | 1.03E+05 | 1.14E+05 | 1.84E+05 | 1.95E+05 | 1.91E+05 | 2.80E+05 | 2.80E+05 | 2.79E+05 | 1.61E+05 | 1.38E+05 | 1.79E+05 | -- | -- |
| pme0021 | 1.66E+02 | 1.20E+02 | 1.65E+02 | C_9_H_11_NO_2_ | [M+H]+ | L-Phenylalanine | Amino acids and derivatives | Amino acids and derivatives | 63-91-2 | 1 | 3.91E+06 | 3.83E+06 | 4.10E+06 | 4.65E+06 | 4.65E+06 | 4.65E+06 | 6.00E+06 | 6.10E+06 | 6.42E+06 | 7.61E+06 | 8.02E+06 | 8.55E+06 | 5.53E+06 | 5.55E+06 | 6.32E+06 | C00079 | ko00360,ko00400,ko00460,ko00940,ko00960,ko00966,ko00970,ko00998,ko01100,ko01110,ko01210,ko01230,ko02010 |
| pme0281 | 1.65E+02 | 1.21E+02 | 1.66E+02 | C_8_H_6_O_4_ | [M-H]- | Terephthalic acid | Phenolic acids | Phenolic acids | 100-21-0 | 3 | 3.90E+05 | 5.83E+05 | 5.65E+05 | 4.79E+05 | 5.14E+05 | 3.86E+05 | 5.70E+05 | 4.70E+05 | 5.05E+05 | 4.33E+05 | 4.81E+05 | 5.27E+05 | 4.24E+05 | 4.70E+05 | 3.98E+05 | C06337 | ko01100 |
| pmc0274 | 1.67E+02 | 1.21E+02 | 1.66E+02 | C_6_H_6_N_4_S | [M+H]+ | 6-Methylmercaptopurine | Nucleotides and derivatives | Nucleotides and derivatives | 50-66-8 | 3 | 2.08E+07 | 2.01E+07 | 2.07E+07 | 2.32E+07 | 2.30E+07 | 2.45E+07 | 2.93E+07 | 2.98E+07 | 3.12E+07 | 3.82E+07 | 4.02E+07 | 3.94E+07 | 2.74E+07 | 2.81E+07 | 2.95E+07 | C16614 | -- |
| mws0344 | 1.65E+02 | 7.50E+01 | 1.66E+02 | C_5_H_10_O_6_ | [M-H]- | D-Xylonic acid | Others | Saccharides and Alcohols | 526-91-0 | 1 | 6.14E+06 | 6.43E+06 | 6.72E+06 | 2.13E+06 | 2.66E+06 | 2.48E+06 | 2.63E+06 | 2.85E+06 | 2.87E+06 | 2.36E+06 | 2.27E+06 | 2.18E+06 | 2.82E+06 | 3.19E+06 | 3.38E+06 | C00502 | ko00040,ko01100 |
| pme1266 | 1.67E+02 | 1.24E+02 | 1.66E+02 | C_6_H_6_N_4_O_2_ | [M+H]+ | 3-Methylxanthine | Nucleotides and derivatives | Nucleotides and derivatives | 1076-22-8 | 3 | 8.03E+04 | 8.18E+04 | 1.08E+05 | 9.00E+00 | 9.00E+00 | 9.00E+00 | 9.00E+00 | 9.00E+00 | 9.00E+00 | 9.00E+00 | 9.00E+00 | 9.00E+00 | 2.32E+04 | 2.61E+04 | 3.62E+04 | C16357 | ko00232,ko01100,ko01110 |
| Lmrn003000 | 1.65E+02 | 1.03E+02 | 1.66E+02 | C_9_H_10_O_3_ | [M-H]- | 2-Hydroxy-3-phenylpropanoic acid | Phenolic acids | Phenolic acids | 7326-19-4 | 1 | 1.25E+04 | 1.41E+04 | 9.51E+03 | 1.53E+04 | 1.95E+04 | 9.09E+03 | 4.88E+04 | 4.38E+04 | 3.66E+04 | 6.86E+04 | 5.06E+04 | 5.17E+04 | 2.63E+04 | 2.43E+04 | 2.91E+04 | C05607 | ko00360,ko00960,ko01100 |
| MWS0274 | 1.65E+02 | 1.19E+02 | 1.66E+02 | C_9_H_10_O_3_ | [M-H]- | DL-3-Phenyllactic acid | Organic acids | Organic acids | 828-01-3 | 1 | 8.61E+04 | 1.11E+05 | 1.41E+05 | 4.21E+04 | 5.84E+04 | 3.73E+04 | 8.07E+04 | 3.62E+04 | 5.62E+04 | 3.77E+04 | 3.78E+04 | 3.17E+04 | 7.40E+04 | 6.40E+04 | 9.31E+04 | -- | -- |
| Lmbn005172 | 1.65E+02 | 1.19E+02 | 1.66E+02 | C_9_H_10_O_3_ | [M-H]- | 2,6-Dimethoxybenzaldehyde | Phenolic acids | Phenolic acids | 3392-97-0 | 1 | 3.24E+04 | 5.44E+04 | 3.64E+04 | 2.08E+04 | 1.79E+04 | 1.47E+04 | 1.59E+04 | 2.75E+04 | 2.64E+04 | 1.75E+04 | 1.29E+04 | 8.05E+03 | 3.35E+04 | 2.66E+04 | 2.72E+04 | -- | -- |
| mws0467 | 1.65E+02 | 1.19E+02 | 1.66E+02 | C_9_H_10_O_3_ | [M-H]- | 3-(4-Hydroxyphenyl)-propionic acid | Phenolic acids | Phenolic acids | 501-97-3 | 2 | 1.56E+05 | 1.48E+05 | 1.68E+05 | 7.31E+04 | 5.32E+04 | 5.99E+04 | 1.05E+05 | 1.09E+05 | 1.07E+05 | 4.17E+04 | 5.71E+04 | 4.91E+04 | 9.80E+04 | 9.50E+04 | 1.07E+05 | C01744 | -- |
| MWSmce165 | 1.65E+02 | 5.90E+01 | 1.66E+02 | C_6_H_14_O_5_ | [M-H]- | L-Fucitol | Others | Saccharides and Alcohols | 13074-06-1 | 2 | 8.10E+05 | 7.76E+05 | 7.18E+05 | 2.80E+05 | 2.09E+05 | 2.63E+05 | 3.53E+05 | 3.62E+05 | 3.84E+05 | 2.79E+05 | 2.55E+05 | 2.39E+05 | 3.23E+05 | 3.78E+05 | 3.88E+05 | -- | -- |
| Zmjp000624 | 1.68E+02 | 1.50E+02 | 1.67E+02 | C_8_H_9_NO_3_ | [M+H]+ | Pyridoxal | Others | Vitamin | 66-72-8 | 2 | 1.50E+05 | 1.56E+05 | 1.52E+05 | 1.66E+05 | 1.80E+05 | 1.65E+05 | 1.09E+05 | 9.40E+04 | 1.03E+05 | 9.06E+04 | 8.48E+04 | 8.12E+04 | 1.22E+05 | 1.28E+05 | 1.38E+05 | C00250 | ko00750,ko01100,ko01240 |
| NK10253223 | 1.68E+02 | 9.41E+01 | 1.67E+02 | C_8_H_9_NO_3_ | [M+H]+ | 2-Amino-3-methoxybenzoic acid | Phenolic acids | Phenolic acids | 3177-80-8 | 3 | 1.13E+05 | 1.15E+05 | 1.06E+05 | 1.59E+05 | 1.52E+05 | 1.60E+05 | 7.09E+04 | 7.22E+04 | 8.92E+04 | 8.05E+04 | 7.73E+04 | 7.00E+04 | 1.02E+05 | 9.48E+04 | 1.14E+05 | C05831 | ko00380 |
| Hmtp000776 | 1.68E+02 | 1.05E+02 | 1.67E+02 | C_8_H_9_NO_3_ | [M+H]+ | 4,5,6-Trihydroxy-2-cyclohexen-1-ylideneacetonitrile | Alkaloids | Alkaloids | - | 2 | 2.97E+05 | 2.94E+05 | 3.19E+05 | 4.09E+05 | 4.03E+05 | 4.08E+05 | 5.08E+05 | 5.33E+05 | 5.48E+05 | 6.44E+05 | 6.54E+05 | 6.91E+05 | 4.73E+05 | 4.75E+05 | 5.35E+05 | -- | -- |
| mws2125 | 1.67E+02 | 7.87E+01 | 1.68E+02 | C_3_H_5_O_6_P | [M-H]- | Phosphoenolpyruvate | Organic acids | Organic acids | 138-08-9 | 3 | 1.34E+05 | 1.25E+05 | 1.32E+05 | 1.61E+05 | 1.33E+05 | 1.67E+05 | 7.19E+04 | 8.87E+04 | 7.99E+04 | 1.89E+05 | 1.81E+05 | 2.11E+05 | 1.06E+05 | 1.51E+05 | 1.20E+05 | C00074 | ko00010,ko00020,ko00400,ko00440,ko00620,ko00710,ko00998,ko01100,ko01110,ko01200,ko01230,ko01240 |
| Hmbp001276 | 1.69E+02 | 6.50E+01 | 1.68E+02 | C_8_H_8_O_4_ | [M+H]+ | Gallacetophenone | Phenolic acids | Phenolic acids | 528-21-2 | 2 | 1.87E+05 | 1.95E+05 | 1.91E+05 | 4.43E+05 | 5.84E+05 | 5.46E+05 | 2.08E+05 | 2.28E+05 | 2.53E+05 | 2.69E+05 | 2.66E+05 | 2.76E+05 | 3.10E+05 | 2.85E+05 | 2.97E+05 | -- | -- |
| mws0028 | 1.67E+02 | 1.08E+02 | 1.68E+02 | C_8_H_8_O_4_ | [M-H]- | Vanillic acid | Phenolic acids | Phenolic acids | 121-34-6 | 1 | 1.29E+06 | 1.14E+06 | 1.26E+06 | 4.79E+05 | 4.80E+05 | 4.99E+05 | 9.95E+05 | 9.41E+05 | 9.94E+05 | 5.30E+05 | 4.83E+05 | 4.66E+05 | 7.40E+05 | 8.27E+05 | 9.00E+05 | C06672 | -- |
| MWSslk066 | 1.69E+02 | 6.50E+01 | 1.68E+02 | C_8_H_8_O_4_ | [M+H]+ | 3-Hydroxy-4-methoxybenzoic acid | Phenolic acids | Phenolic acids | 645-08-9 | 1 | 6.29E+05 | 6.27E+05 | 5.59E+05 | 4.81E+05 | 4.80E+05 | 4.92E+05 | 5.86E+05 | 5.41E+05 | 5.83E+05 | 7.27E+05 | 6.14E+05 | 6.30E+05 | 5.97E+05 | 4.73E+05 | 6.39E+05 | -- | -- |
| MWStz091 | 1.69E+02 | 7.01E+01 | 1.68E+02 | C_8_H_12_N_2_O_2_ | [M+H]+ | Cyclo(L-Ala-L-Pro) | Amino acids and derivatives | Amino acids and derivatives | 36357-32-1 | 2 | 9.19E+04 | 8.25E+04 | 9.73E+04 | 9.00E+00 | 9.00E+00 | 9.00E+00 | 3.58E+04 | 3.28E+04 | 4.05E+04 | 9.00E+00 | 9.00E+00 | 9.00E+00 | 3.58E+04 | 4.38E+04 | 3.58E+04 | -- | -- |
| pme1383 | 1.70E+02 | 1.34E+02 | 1.69E+02 | C_8_H_11_NO_3_ | [M+H]+ | Pyridoxine | Others | Vitamin | 65-23-6 | 3 | 5.75E+05 | 6.77E+05 | 6.80E+05 | 1.16E+05 | 1.60E+05 | 1.30E+05 | 3.19E+05 | 3.58E+05 | 3.41E+05 | 6.60E+04 | 5.80E+04 | 6.32E+04 | 3.57E+05 | 3.84E+05 | 3.89E+05 | C00314 | ko00750,ko01100,ko01240 |
| pme0181 | 1.70E+02 | 1.24E+02 | 1.69E+02 | C_7_H_11_N_3_O_2_ | [M+H]+ | 3-Methyl-L-Histidine | Amino acids and derivatives | Amino acids and derivatives | 368-16-1 | 3 | 3.36E+05 | 3.14E+05 | 3.23E+05 | 4.09E+05 | 4.33E+05 | 3.78E+05 | 3.05E+05 | 2.72E+05 | 2.79E+05 | 2.94E+05 | 2.99E+05 | 3.14E+05 | 3.40E+05 | 3.27E+05 | 3.60E+05 | C01152 | ko00340,ko01100 |
| Zmzn000078 | 1.69E+02 | 7.90E+01 | 1.70E+02 | C_3_H_7_O_6_P | [M-H]- | Dihydroxyacetone phosphate | Others | Saccharides and Alcohols | 57-04-5 | 3 | 7.06E+03 | 1.19E+04 | 1.07E+04 | 3.34E+04 | 3.59E+04 | 3.03E+04 | 1.46E+04 | 1.01E+04 | 2.18E+03 | 4.88E+04 | 3.95E+04 | 4.32E+04 | 2.68E+04 | 3.25E+04 | 2.77E+04 | C00111 | ko00010,ko00040,ko00051,ko00052,ko00561,ko00562,ko00564,ko00640,ko00710,ko00760,ko01100,ko01110,ko01200,ko01230 |
| pme3186 | 1.69E+02 | 7.90E+01 | 1.70E+02 | C_3_H_7_O_6_P | [M-H]- | DL-Glyceraldehyde-3-phosphate | Organic acids | Organic acids | 591-59-3 | 3 | 2.98E+05 | 3.35E+05 | 4.12E+05 | 5.89E+04 | 4.57E+04 | 4.73E+04 | 5.62E+04 | 6.28E+04 | 6.09E+04 | 1.05E+05 | 1.04E+05 | 1.25E+05 | 1.15E+05 | 1.51E+05 | 1.23E+05 | C00661 | -- |
| mws0024 | 1.69E+02 | 1.25E+02 | 1.70E+02 | C_7_H_6_O_5_ | [M-H]- | Gallic acid | Phenolic acids | Phenolic acids | 149-91-7 | 3 | 1.51E+05 | 1.80E+05 | 2.10E+05 | 5.18E+04 | 3.89E+04 | 6.94E+04 | 3.29E+05 | 2.79E+05 | 2.95E+05 | 9.00E+00 | 9.00E+00 | 9.00E+00 | 1.22E+05 | 1.48E+05 | 1.45E+05 | C01424 | -- |
| ML10176345 | 1.71E+02 | 1.09E+02 | 1.72E+02 | C_7_H_8_O_5_ | [M-H]- | 3-Dehydroshikimic acid | Organic acids | Organic acids | 2922-42-1 | 3 | 5.33E+03 | 2.32E+04 | 9.45E+03 | 3.46E+04 | 1.71E+04 | 4.31E+04 | 9.00E+00 | 9.00E+00 | 9.00E+00 | 8.71E+03 | 5.80E+03 | 9.91E+03 | 1.55E+04 | 3.24E+04 | 1.84E+04 | C02637 | ko00400,ko01100,ko01110,ko01230,ko01240 |
| MWSmce625 | 1.37E+02 | 5.51E+01 | 1.72E+02 | C_8_H_12_N_2_ | [M+H]+ | 2,3,5,6-Tetramethylpyrazine; Ligustrazine | Alkaloids | Alkaloids | 1124-11-4 | 1 | 5.66E+06 | 5.45E+06 | 7.29E+06 | 9.00E+00 | 9.00E+00 | 9.00E+00 | 9.00E+00 | 9.00E+00 | 9.00E+00 | 9.00E+00 | 9.00E+00 | 9.00E+00 | 1.67E+06 | 1.58E+06 | 1.88E+06 | -- | -- |
| Lmbn003524 | 1.71E+02 | 9.91E+01 | 1.72E+02 | C_9_H_16_O_3_ | [M-H]- | 9-Oxononanoic acid | Organic acids | Organic acids | 2553-17-5 | 3 | 6.07E+04 | 5.17E+04 | 5.96E+04 | 3.49E+04 | 3.34E+04 | 3.66E+04 | 9.66E+04 | 9.45E+04 | 9.68E+04 | 2.77E+04 | 2.02E+04 | 2.37E+04 | 5.17E+04 | 5.40E+04 | 6.07E+04 | C16322 | ko00592,ko01110 |
| MWS5169 | 1.72E+02 | 1.28E+02 | 1.73E+02 | C_10_H_7_NO_2_ | [M-H]- | Quinoline-4-carboxylic acid | Alkaloids | Quinoline alkaloids | 486-74-8 | 2 | 3.99E+04 | 3.50E+04 | 3.51E+04 | 1.88E+05 | 1.79E+05 | 1.87E+05 | 1.28E+05 | 1.27E+05 | 1.43E+05 | 2.51E+05 | 2.49E+05 | 2.56E+05 | 1.26E+05 | 1.39E+05 | 1.56E+05 | C06414 | -- |
| pme0253 | 1.74E+02 | 8.60E+01 | 1.73E+02 | C_8_H_15_NO_3_ | [M+H]+ | N-Acetyl-L-leucine | Amino acids and derivatives | Amino acids and derivatives | 1188-21-2 | 3 | 7.44E+05 | 9.25E+05 | 1.02E+06 | 1.07E+04 | 2.20E+04 | 2.28E+04 | 4.81E+05 | 3.53E+05 | 4.46E+05 | 1.71E+04 | 6.32E+03 | 1.49E+04 | 3.49E+05 | 3.24E+05 | 4.24E+05 | C02710 | -- |
| MA10039492 | 1.73E+02 | 7.10E+01 | 1.74E+02 | C_6_H_6_O_6_ | [M-H]- | Dehydroascorbic acid | Others | Vitamin | 490-83-5 | 3 | 9.00E+00 | 9.00E+00 | 9.00E+00 | 2.87E+04 | 2.00E+04 | 2.65E+04 | 9.00E+00 | 9.00E+00 | 9.00E+00 | 2.07E+04 | 9.18E+03 | 1.50E+04 | 1.38E+04 | 1.62E+04 | 7.16E+03 | C05422 | ko00053,ko00480,ko01100 |
| pme3009 | 1.75E+02 | 9.90E+01 | 1.74E+02 | C_6_H_6_O_6_ | [M+H]+ | Trans-Citridic acid | Organic acids | Organic acids | 4023-65-8 | 2 | 1.43E+05 | 1.54E+05 | 1.43E+05 | 2.43E+05 | 2.45E+05 | 2.52E+05 | 2.18E+05 | 1.97E+05 | 1.92E+05 | 2.28E+05 | 2.64E+05 | 2.35E+05 | 2.01E+05 | 1.95E+05 | 1.90E+05 | C02341 | ko00660,ko01100 |
| MWS4471 | 1.73E+02 | 5.80E+01 | 1.74E+02 | C_6_H_10_N_2_O_4_ | [M-H]- | N-Alpha-Acetyl-L-Asparagine | Amino acids and derivatives | Amino acids and derivatives | 4033-40-3 | 2 | 2.01E+05 | 1.86E+05 | 1.77E+05 | 3.16E+05 | 3.32E+05 | 3.16E+05 | 2.67E+05 | 2.81E+05 | 2.90E+05 | 2.99E+05 | 3.19E+05 | 3.32E+05 | 2.66E+05 | 2.84E+05 | 2.49E+05 | -- | -- |
| Lmgn002555 | 1.73E+02 | 5.90E+01 | 1.74E+02 | C_8_H_14_O_4_ | [M-H]- | 2-Propylglutaric acid | Organic acids | Organic acids | 32806-62-5 | 3 | 1.09E+05 | 8.38E+04 | 9.50E+04 | 1.23E+05 | 9.28E+04 | 1.25E+05 | 3.13E+05 | 2.85E+05 | 3.60E+05 | 3.35E+05 | 3.85E+05 | 3.44E+05 | 1.74E+05 | 2.50E+05 | 2.47E+05 | C16658 | -- |
| mws0242 | 1.73E+02 | 1.11E+02 | 1.74E+02 | C_8_H_14_O_4_ | [M-H]- | Suberic Acid | Organic acids | Organic acids | 505-48-6 | 3 | 6.82E+04 | 6.87E+04 | 6.36E+04 | 8.38E+04 | 7.32E+04 | 6.61E+04 | 2.43E+04 | 2.04E+04 | 3.09E+04 | 2.45E+04 | 2.40E+04 | 2.07E+04 | 3.01E+04 | 4.68E+04 | 7.84E+04 | C08278 | -- |
| Zmyn000155 | 1.73E+02 | 1.31E+02 | 1.74E+02 | C_7_H_14_N_2_O_3_ | [M-H]- | N-α-Acetyl-L-ornithine | Amino acids and derivatives | Amino acids and derivatives | 6205-08-9 | 1 | 1.44E+07 | 1.28E+07 | 1.39E+07 | 2.44E+07 | 2.58E+07 | 2.49E+07 | 2.02E+07 | 2.13E+07 | 2.21E+07 | 2.43E+07 | 2.42E+07 | 2.43E+07 | 1.91E+07 | 2.09E+07 | 2.22E+07 | C00437 | ko00220,ko01100,ko01110,ko01210,ko01230 |
| mws0260 | 1.75E+02 | 1.16E+02 | 1.74E+02 | C_6_H_14_N_4_O_2_ | [M+H]+ | L-Arginine | Amino acids and derivatives | Amino acids and derivatives | 74-79-3 | 1 | 9.26E+06 | 8.91E+06 | 9.13E+06 | 1.55E+07 | 1.55E+07 | 1.60E+07 | 1.24E+07 | 1.26E+07 | 1.33E+07 | 1.63E+07 | 1.69E+07 | 1.66E+07 | 1.36E+07 | 1.38E+07 | 1.40E+07 | C00062 | ko00220,ko00261,ko00330,ko00472,ko00970,ko01100,ko01110,ko01230,ko02010 |
| pme1651 | 1.76E+02 | 1.30E+02 | 1.75E+02 | C_10_H_9_NO_2_ | [M+H]+ | Indole 3-acetic acid (IAA) | Alkaloids | Plumerane | 87-51-4 | 3 | 2.11E+05 | 2.28E+05 | 2.11E+05 | 2.07E+04 | 1.96E+04 | 1.54E+04 | 4.07E+05 | 4.24E+05 | 3.93E+05 | 4.19E+04 | 5.56E+04 | 5.12E+04 | 1.71E+05 | 1.65E+05 | 1.85E+05 | C00954 | ko00380,ko01100,ko04075 |
| pme0008 | 1.76E+02 | 1.13E+02 | 1.75E+02 | C_6_H_13_N_3_O_3_ | [M+H]+ | L-Citrulline | Amino acids and derivatives | Amino acids and derivatives | 372-75-8 | 3 | 7.72E+06 | 7.45E+06 | 1.03E+07 | 5.45E+06 | 5.78E+06 | 5.64E+06 | 4.88E+06 | 4.97E+06 | 5.19E+06 | 6.05E+06 | 6.19E+06 | 5.94E+06 | 5.87E+06 | 6.27E+06 | 6.36E+06 | C00327 | ko00220,ko01100,ko01110,ko01230 |
| mws1075 | 1.77E+02 | 1.21E+02 | 1.76E+02 | C_10_H_8_O_3_ | [M+H]+ | 7-Methoxycoumarin | Lignans and Coumarins | Coumarins | 531-59-9 | 3 | 3.13E+04 | 3.66E+04 | 3.18E+04 | 2.70E+04 | 2.47E+04 | 2.42E+04 | 5.07E+04 | 3.09E+04 | 6.03E+04 | 1.21E+04 | 1.15E+04 | 1.00E+04 | 3.61E+04 | 3.08E+04 | 2.83E+04 | C09268 | -- |
| Zmgn001448 | 1.75E+02 | 1.15E+02 | 1.76E+02 | C_7_H_12_O_5_ | [M-H]- | 2-Propylmalic Acid* | Organic acids | Organic acids | - | 1 | 1.08E+07 | 1.12E+07 | 1.12E+07 | 2.57E+06 | 2.58E+06 | 2.52E+06 | 2.66E+06 | 2.47E+06 | 2.69E+06 | 6.00E+05 | 5.95E+05 | 6.16E+05 | 4.27E+06 | 4.66E+06 | 4.84E+06 | C05994 | ko00620 |
| pmb3101 | 1.75E+02 | 1.15E+02 | 1.76E+02 | C_7_H_12_O_5_ | [M-H]- | 2-Isopropylmalic Acid | Organic acids | Organic acids | 49601-06-1 | 1 | 1.43E+07 | 1.42E+07 | 1.53E+07 | 3.78E+06 | 3.66E+06 | 3.44E+06 | 3.41E+06 | 3.58E+06 | 3.58E+06 | 7.83E+05 | 9.23E+05 | 8.50E+05 | 5.83E+06 | 6.21E+06 | 6.76E+06 | C02504 | ko00290,ko00620,ko01100,ko01110,ko01210,ko01230 |
| Lmbn001754 | 1.75E+02 | 1.15E+02 | 1.76E+02 | C_7_H_12_O_5_ | [M-H]- | 3-Isopropylmalic Acid* | Organic acids | Organic acids | 921-28-8 | 1 | 1.25E+07 | 1.31E+07 | 1.37E+07 | 3.27E+06 | 3.53E+06 | 3.10E+06 | 3.42E+06 | 3.16E+06 | 3.22E+06 | 7.30E+05 | 7.93E+05 | 8.12E+05 | 5.54E+06 | 5.70E+06 | 6.33E+06 | C04411 | ko00290,ko01100,ko01110,ko01210,ko01230 |
| mws1013 | 1.77E+02 | 1.33E+02 | 1.78E+02 | C_9_H_6_O_4_ | [M-H]- | Esculetin | Lignans and Coumarins | Coumarins | 305-01-1 | 2 | 6.89E+04 | 5.69E+04 | 6.93E+04 | 1.46E+05 | 1.41E+05 | 1.47E+05 | 1.13E+05 | 1.02E+05 | 1.46E+05 | 6.26E+04 | 6.46E+04 | 7.44E+04 | 8.47E+04 | 8.18E+04 | 9.84E+04 | C09263 | -- |
| mws1195 | 1.79E+02 | 1.47E+02 | 1.78E+02 | C_10_H_10_O_3_ | [M+H]+ | p-Coumaric acid methyl ester | Phenolic acids | Phenolic acids | 3943-97-3 | 2 | 2.74E+04 | 1.96E+04 | 2.16E+04 | 1.21E+04 | 1.05E+04 | 1.28E+04 | 1.76E+05 | 1.99E+05 | 2.10E+05 | 4.98E+04 | 6.78E+04 | 5.37E+04 | 6.80E+04 | 5.73E+04 | 6.97E+04 | -- | -- |
| mws0009 | 1.79E+02 | 9.10E+01 | 1.78E+02 | C_10_H_10_O_3_ | [M+H]+ | Coniferaldehyde | Phenolic acids | Phenolic acids | 20649-42-7 | 1 | 2.25E+04 | 1.22E+04 | 9.91E+03 | 2.00E+04 | 2.36E+04 | 2.56E+04 | 8.62E+04 | 7.35E+04 | 9.60E+04 | 2.51E+04 | 2.32E+04 | 3.34E+04 | 3.16E+04 | 3.37E+04 | 4.88E+04 | C02666 | ko00940,ko01100,ko01110 |
| Jmzn006005 | 1.77E+02 | 1.17E+02 | 1.78E+02 | C_10_H_10_O_3_ | [M-H]- | 3,4-Methylenedioxy cinnamyl alcohol | Lignans and Coumarins | Lignans | 58095-76-4 | 3 | 7.42E+04 | 4.61E+04 | 5.85E+04 | 1.89E+04 | 2.08E+04 | 2.18E+04 | 3.68E+05 | 4.00E+05 | 4.00E+05 | 1.01E+05 | 1.19E+05 | 9.87E+04 | 1.28E+05 | 1.52E+05 | 1.51E+05 | -- | -- |
| pmb2795 | 1.77E+02 | 1.45E+02 | 1.78E+02 | C_10_H_10_O_3_ | [M-H]- | 4-Methoxycinnamic acid | Phenolic acids | Phenolic acids | 830-09-1 | 3 | 3.55E+04 | 2.26E+04 | 2.48E+04 | 1.19E+04 | 1.11E+04 | 1.04E+04 | 1.71E+05 | 1.96E+05 | 2.00E+05 | 4.99E+04 | 5.73E+04 | 4.93E+04 | 6.08E+04 | 6.63E+04 | 6.93E+04 | -- | -- |
| mws2212 | 1.79E+02 | 1.35E+02 | 1.80E+02 | C_9_H_8_O_4_ | [M-H]- | Caffeic acid | Phenolic acids | Phenolic acids | 331-39-5 | 1 | 1.34E+06 | 1.18E+06 | 1.16E+06 | 3.99E+06 | 3.83E+06 | 3.85E+06 | 3.47E+06 | 3.11E+06 | 3.54E+06 | 1.82E+06 | 1.76E+06 | 1.88E+06 | 2.51E+06 | 3.01E+06 | 2.80E+06 | C01197 | ko00940,ko01100,ko01110 |
| mws4170 | 1.79E+02 | 5.90E+01 | 1.80E+02 | C_6_H_12_O_6_ | [M-H]- | D-Glucose | Others | Saccharides and Alcohols | 50-99-7 | 1 | 4.96E+06 | 6.21E+06 | 6.15E+06 | 1.22E+07 | 1.24E+07 | 1.13E+07 | 5.25E+06 | 4.91E+06 | 6.73E+06 | 4.28E+06 | 4.49E+06 | 4.82E+06 | 6.55E+06 | 8.09E+06 | 8.19E+06 | C00031 | ko00010,ko00030,ko00052,ko00500,ko00520,ko00524,ko00901,ko01100,ko01110,ko02010 |
| pmf0139 | 1.79E+02 | 5.90E+01 | 1.80E+02 | C_6_H_12_O_6_ | [M-H]- | D-Galactose | Others | Saccharides and Alcohols | 59-23-4 | 3 | 5.18E+06 | 4.52E+06 | 4.73E+06 | 1.04E+07 | 1.04E+07 | 1.09E+07 | 3.98E+06 | 4.20E+06 | 3.93E+06 | 3.49E+06 | 3.84E+06 | 3.50E+06 | 5.46E+06 | 6.11E+06 | 6.00E+06 | C00124 | ko00052,ko01100 |
| Hmln000297 | 1.79E+02 | 5.90E+01 | 1.80E+02 | C_6_H_12_O_6_ | [M-H]- | Inositol | Others | Saccharides and Alcohols | 87-89-8 | 1 | 6.55E+06 | 7.10E+06 | 6.59E+06 | 1.22E+07 | 1.20E+07 | 1.25E+07 | 4.43E+06 | 4.85E+06 | 4.87E+06 | 4.56E+06 | 4.62E+06 | 3.83E+06 | 6.62E+06 | 6.09E+06 | 7.11E+06 | C00137 | ko00052,ko00053,ko00562,ko01100,ko01110,ko02010,ko04070 |
| mws1164 | 1.79E+02 | 5.90E+01 | 1.80E+02 | C_6_H_12_O_6_ | [M-H]- | D-Fructose | Others | Saccharides and Alcohols | 57-48-7 | 1 | 7.74E+06 | 7.36E+06 | 6.90E+06 | 1.12E+07 | 1.32E+07 | 1.28E+07 | 4.88E+06 | 5.20E+06 | 5.71E+06 | 4.92E+06 | 4.63E+06 | 4.53E+06 | 7.80E+06 | 8.34E+06 | 7.62E+06 | C05003 | -- |
| MWSmce576 | 1.79E+02 | 5.90E+01 | 1.80E+02 | C_6_H_12_O_6_ | [M-H]- | L-Glucose | Others | Saccharides and Alcohols | 921-60-8 | 1 | 7.61E+06 | 6.58E+06 | 6.83E+06 | 1.23E+07 | 1.20E+07 | 1.36E+07 | 5.43E+06 | 5.17E+06 | 5.41E+06 | 4.79E+06 | 4.45E+06 | 4.68E+06 | 7.43E+06 | 6.98E+06 | 7.48E+06 | -- | -- |
| mws0093 | 1.79E+02 | 1.46E+02 | 1.80E+02 | C_10_H_12_O_3_ | [M-H]- | Coniferyl alcohol | Phenolic acids | Phenolic acids | 458-35-5 | 3 | 4.64E+04 | 5.43E+04 | 4.00E+04 | 3.17E+04 | 3.04E+04 | 3.75E+04 | 1.73E+05 | 1.91E+05 | 1.95E+05 | 3.51E+04 | 3.68E+04 | 6.31E+04 | 7.85E+04 | 6.31E+04 | 7.93E+04 | C00590 | ko00940,ko00998,ko01100,ko01110 |
| mws0250 | 1.82E+02 | 1.36E+02 | 1.81E+02 | C_9_H_11_NO_3_ | [M+H]+ | L-Tyrosine | Amino acids and derivatives | Amino acids and derivatives | 60-18-4 | 1 | 4.37E+06 | 4.42E+06 | 4.52E+06 | 5.33E+06 | 5.26E+06 | 5.51E+06 | 9.30E+06 | 1.01E+07 | 1.02E+07 | 1.49E+07 | 1.44E+07 | 1.37E+07 | 8.55E+06 | 7.68E+06 | 9.56E+06 | C00082 | ko00130,ko00261,ko00350,ko00360,ko00400,ko00460,ko00730,ko00940,ko00950,ko00965,ko00966,ko00970,ko00998,ko01100,ko01110,ko01210,ko01230,ko01240 |
| Zmsp000878 | 1.82E+02 | 1.36E+02 | 1.81E+02 | C_9_H_11_NO_3_ | [M+H]+ | 4-Hydroxy-5-(2-oxo-1-pyrrolidinyl)benzoic acid | Alkaloids | Pyrrole alkaloids | - | 1 | 1.60E+07 | 1.55E+07 | 1.63E+07 | 1.92E+07 | 1.91E+07 | 1.95E+07 | 3.24E+07 | 3.35E+07 | 3.49E+07 | 4.46E+07 | 4.49E+07 | 4.37E+07 | 3.03E+07 | 2.63E+07 | 3.14E+07 | -- | -- |
| MWStz070 | 1.82E+02 | 9.11E+01 | 1.81E+02 | C_9_H_11_NO_3_ | [M+H]+ | N-(2-Hydroxy-4-methoxyphenyl)acetamide | Alkaloids | Phenolamine | 58469-06-0 | 1 | 8.48E+06 | 8.14E+06 | 8.63E+06 | 1.01E+07 | 9.70E+06 | 9.66E+06 | 1.85E+07 | 1.84E+07 | 1.96E+07 | 2.60E+07 | 2.53E+07 | 2.45E+07 | 1.63E+07 | 1.45E+07 | 1.67E+07 | -- | -- |
| Hmtn001288 | 1.81E+02 | 1.35E+02 | 1.82E+02 | C_9_H_10_O_4_ | [M-H]- | Methyl 2,4-dihydroxyphenylacetate | Phenolic acids | Phenolic acids | 67828-42-6 | 3 | 8.15E+04 | 7.29E+04 | 8.32E+04 | 9.00E+00 | 9.00E+00 | 9.00E+00 | 7.08E+04 | 6.81E+04 | 7.03E+04 | 9.00E+00 | 9.00E+00 | 9.00E+00 | 3.35E+04 | 5.81E+04 | 4.10E+04 | -- | -- |
| Hmcn006267 | 1.81E+02 | 1.07E+02 | 1.82E+02 | C_9_H_10_O_4_ | [M-H]- | 2,6-Dimethoxybenzoic acid | Phenolic acids | Phenolic acids | 1466-76-8 | 3 | 1.04E+04 | 7.56E+03 | 8.91E+03 | 1.19E+04 | 1.52E+04 | 1.49E+04 | 1.25E+04 | 1.03E+04 | 1.32E+04 | 1.98E+04 | 1.68E+04 | 1.57E+04 | 1.17E+04 | 1.20E+04 | 1.56E+04 | -- | -- |
| Lmrn001951 | 1.81E+02 | 1.35E+02 | 1.82E+02 | C_9_H_10_O_4_ | [M-H]- | (S)-2-Hydroxy-3-(4-Hydroxyphenyl)Propanoic Acid | Phenolic acids | Phenolic acids | 23508-35-2 | 3 | 5.63E+04 | 5.63E+04 | 5.79E+04 | 9.00E+00 | 9.00E+00 | 9.00E+00 | 5.43E+04 | 4.22E+04 | 4.40E+04 | 9.00E+00 | 9.00E+00 | 9.00E+00 | 2.24E+04 | 3.24E+04 | 2.75E+04 | -- | -- |
| mws1350 | 1.81E+02 | 1.51E+02 | 1.82E+02 | C_9_H_10_O_4_ | [M-H]- | Syringaldehyde; 4-Hydroxy-3,5-Dimethoxybenzaldehyde | Phenolic acids | Phenolic acids | 134-96-3 | 3 | 4.29E+04 | 4.06E+04 | 4.07E+04 | 9.96E+03 | 2.46E+04 | 1.41E+04 | 1.40E+04 | 1.79E+04 | 1.67E+04 | 9.00E+00 | 9.00E+00 | 9.00E+00 | 1.80E+04 | 8.67E+03 | 2.23E+04 | -- | -- |
| Lmlp001436 | 1.83E+02 | 1.65E+02 | 1.82E+02 | C_9_H_10_O_4_ | [M+H]+ | Dihydrocaffeic acid | Phenolic acids | Phenolic acids | 1078-61-1 | 3 | 7.43E+05 | 7.50E+05 | 8.08E+05 | 9.00E+00 | 9.00E+00 | 9.00E+00 | 9.00E+00 | 9.00E+00 | 9.00E+00 | 9.00E+00 | 9.00E+00 | 9.00E+00 | 2.48E+05 | 2.32E+05 | 2.56E+05 | C10447 | ko00350 |
| mws1155 | 1.81E+02 | 1.01E+02 | 1.82E+02 | C_6_H_14_O_6_ | [M-H]- | D-Mannitol | Others | Saccharides and Alcohols | 69-65-8 | 3 | 8.50E+04 | 8.67E+04 | 9.89E+04 | 9.38E+04 | 1.01E+05 | 1.24E+05 | 1.01E+05 | 1.02E+05 | 1.09E+05 | 1.45E+05 | 1.28E+05 | 1.18E+05 | 1.08E+05 | 1.03E+05 | 1.32E+05 | C00392 | ko00051,ko01100,ko02010 |
| mws0214 | 1.81E+02 | 7.10E+01 | 1.82E+02 | C_6_H_14_O_6_ | [M-H]- | D-Sorbitol | Others | Saccharides and Alcohols | 50-70-4 | 3 | 1.70E+05 | 1.77E+05 | 1.26E+05 | 2.40E+05 | 2.55E+05 | 2.56E+05 | 2.47E+05 | 2.07E+05 | 2.10E+05 | 2.49E+05 | 2.07E+05 | 1.97E+05 | 1.71E+05 | 2.30E+05 | 2.07E+05 | C00794 | ko00051,ko00052,ko01100,ko02010 |
| pme2237 | 1.81E+02 | 1.01E+02 | 1.82E+02 | C_6_H_14_O_6_ | [M-H]- | Dulcitol | Others | Saccharides and Alcohols | 608-66-2 | 3 | 2.81E+05 | 2.31E+05 | 2.39E+05 | 3.31E+05 | 3.40E+05 | 3.42E+05 | 3.24E+05 | 2.87E+05 | 2.74E+05 | 3.75E+05 | 3.73E+05 | 3.61E+05 | 2.56E+05 | 3.25E+05 | 3.24E+05 | C01697 | ko00052,ko01100 |
| pme2596 | 1.84E+02 | 1.48E+02 | 1.83E+02 | C_8_H_9_NO_4_ | [M+H]+ | 4-Pyridoxic acid | Others | Vitamin | 82-82-6 | 3 | 3.04E+04 | 3.07E+04 | 3.19E+04 | 2.57E+04 | 2.21E+04 | 2.08E+04 | 1.80E+04 | 1.95E+04 | 2.08E+04 | 2.42E+04 | 2.79E+04 | 2.34E+04 | 2.01E+04 | 2.15E+04 | 2.64E+04 | C00847 | ko00750,ko01100 |
| pmb1754 | 1.84E+02 | 1.25E+02 | 1.84E+02 | C_5_H_15_NO_4_P+ | [M]+ | O-Phosphocholine | Alkaloids | Alkaloids | 107-73-3 | 3 | 2.90E+04 | 2.78E+04 | 4.94E+04 | 5.41E+04 | 5.60E+04 | 6.07E+04 | 5.55E+04 | 4.92E+04 | 5.53E+04 | 5.82E+04 | 4.50E+04 | 5.30E+04 | 5.27E+04 | 4.64E+04 | 5.11E+04 | C00588 | ko00564,ko01100 |
| pme2602 | 1.84E+02 | 9.70E+01 | 1.85E+02 | C_3_H_8_NO_6_P | [M-H]- | O-Phospho-L-serine | Amino acids and derivatives | Amino acids and derivatives | 407-41-0 | 3 | 8.48E+03 | 7.52E+03 | 5.03E+03 | 2.18E+04 | 3.63E+04 | 2.70E+04 | 1.50E+04 | 2.03E+04 | 2.57E+04 | 3.48E+04 | 3.73E+04 | 3.40E+04 | 2.01E+04 | 2.52E+04 | 2.67E+04 | C01005 | ko00260,ko00270,ko00970,ko01100,ko01110,ko01200,ko01230 |
| Zmgn000447 | 1.85E+02 | 7.90E+01 | 1.86E+02 | C_3_H_7_O_7_P | [M-H]- | 3-Phospho-D-glyceric acid | Others | Saccharides and Alcohols | 820-11-1 | 3 | 1.66E+05 | 1.36E+05 | 1.40E+05 | 3.48E+05 | 3.88E+05 | 3.95E+05 | 3.90E+05 | 3.72E+05 | 4.63E+05 | 3.75E+05 | 4.26E+05 | 4.23E+05 | 3.13E+05 | 3.41E+05 | 3.50E+05 | C00197 | ko00010,ko00030,ko00260,ko00270,ko00561,ko00630,ko00710,ko01100,ko01110,ko01200,ko01230,ko01240 |
| MWSmce324 | 1.87E+02 | 1.31E+02 | 1.86E+02 | C_11_H_6_O_3_ | [M+H]+ | Psoralen* | Lignans and Coumarins | Coumarins | 66-97-7 | 1 | 4.95E+07 | 5.75E+07 | 4.65E+07 | 4.88E+07 | 4.61E+07 | 4.77E+07 | 7.06E+06 | 7.05E+06 | 7.73E+06 | 3.77E+06 | 3.72E+06 | 3.96E+06 | 3.14E+07 | 3.19E+07 | 3.39E+07 | C09305 | ko01110 |
| mws1668 | 1.87E+02 | 1.31E+02 | 1.86E+02 | C_11_H_6_O_3_ | [M+H]+ | Angelicin* | Lignans and Coumarins | Coumarins | 523-50-2 | 1 | 4.40E+07 | 5.27E+07 | 4.83E+07 | 4.62E+07 | 4.49E+07 | 4.50E+07 | 6.87E+06 | 7.11E+06 | 7.22E+06 | 3.55E+06 | 3.64E+06 | 3.76E+06 | 2.89E+07 | 2.85E+07 | 3.13E+07 | C09060 | ko01110 |
| mws0752 | 1.85E+02 | 1.85E+02 | 1.86E+02 | C_11_H_22_O_2_ | [M-H]- | Undecylic Acid | Lipids | Free fatty acids | 112-37-8 | 2 | 4.30E+05 | 3.98E+05 | 4.11E+05 | 4.40E+05 | 3.91E+05 | 4.13E+05 | 3.99E+05 | 4.06E+05 | 3.68E+05 | 4.26E+05 | 4.06E+05 | 4.44E+05 | 4.24E+05 | 4.69E+05 | 4.86E+05 | C17715 | -- |
| Hmmp001310 | 1.88E+02 | 1.18E+02 | 1.87E+02 | C_11_H_9_NO_2_ | [M+H]+ | 3-Indoleacrylic acid | Alkaloids | Plumerane | 1204-06-4 | 1 | 1.53E+07 | 1.66E+07 | 1.44E+07 | 2.21E+07 | 2.26E+07 | 2.36E+07 | 4.44E+07 | 4.43E+07 | 4.69E+07 | 5.23E+07 | 5.53E+07 | 5.79E+07 | 3.59E+07 | 3.36E+07 | 4.04E+07 | C21283 | -- |
| Hmgp002327 | 1.88E+02 | 1.18E+02 | 1.87E+02 | C_11_H_9_O_2_N | [M+H]+ | 3-amino-2-naphthoic acid | Alkaloids | Alkaloids | - | 1 | 1.67E+07 | 1.70E+07 | 1.74E+07 | 2.30E+07 | 2.25E+07 | 2.09E+07 | 4.64E+07 | 4.57E+07 | 4.79E+07 | 5.42E+07 | 5.79E+07 | 5.54E+07 | 3.72E+07 | 3.53E+07 | 4.12E+07 | -- | -- |
| Smcp001137 | 1.88E+02 | 1.18E+02 | 1.87E+02 | C_11_H_9_NO_2_ | [M+H]+ | naphthisoxazol A | Others | Others | - | 2 | 1.69E+06 | 1.75E+06 | 1.80E+06 | 2.50E+06 | 2.51E+06 | 2.51E+06 | 5.18E+06 | 5.13E+06 | 5.32E+06 | 6.49E+06 | 7.08E+06 | 6.25E+06 | 4.53E+06 | 4.12E+06 | 4.43E+06 | -- | -- |
| pmn001492 | 1.87E+02 | 1.23E+02 | 1.88E+02 | C_11_H_8_O_3_ | [M-H]- | Ayapin | Lignans and Coumarins | Coumarins | 494-56-4 | 2 | 1.42E+05 | 1.25E+05 | 1.42E+05 | 1.75E+05 | 1.71E+05 | 1.86E+05 | 5.08E+04 | 6.05E+04 | 4.57E+04 | 3.14E+04 | 4.16E+04 | 3.68E+04 | 9.33E+04 | 1.13E+05 | 1.07E+05 | C18078 | -- |
| pme0137 | 1.87E+02 | 1.25E+02 | 1.88E+02 | C_7_H_12_N_2_O_4_ | [M-H]- | N-Acetyl-L-Glutamine | Amino acids and derivatives | Amino acids and derivatives | 2490-97-3 | 3 | 8.50E+04 | 1.04E+05 | 1.20E+05 | 9.00E+00 | 9.00E+00 | 9.00E+00 | 1.16E+05 | 1.07E+05 | 1.23E+05 | 9.00E+00 | 9.00E+00 | 9.00E+00 | 5.73E+04 | 5.23E+04 | 5.06E+04 | -- | -- |
| pmn001380 | 1.87E+02 | 1.69E+02 | 1.88E+02 | C_9_H_16_O_4_ | [M-H]- | Eucommiol | Others | Others | 55930-44-4 | 1 | 1.83E+05 | 1.69E+05 | 1.81E+05 | 2.78E+05 | 2.55E+05 | 2.84E+05 | 9.37E+04 | 7.08E+04 | 7.55E+04 | 3.56E+04 | 3.92E+04 | 4.57E+04 | 1.36E+05 | 1.62E+05 | 1.58E+05 | C17878 | -- |
| mws0237 | 1.87E+02 | 1.25E+02 | 1.88E+02 | C_9_H_16_O_4_ | [M-H]- | Azelaic acid | Organic acids | Organic acids | 123-99-9 | 1 | 3.09E+06 | 2.92E+06 | 2.99E+06 | 4.62E+06 | 4.46E+06 | 4.53E+06 | 1.30E+06 | 1.32E+06 | 1.22E+06 | 8.90E+05 | 8.47E+05 | 8.16E+05 | 2.38E+06 | 2.54E+06 | 2.83E+06 | C08261 | -- |
| mws0736 | 1.89E+02 | 8.60E+01 | 1.88E+02 | C_8_H_16_N_2_O_3_ | [M+H]+ | N-Glycyl-L-leucine* | Amino acids and derivatives | Amino acids and derivatives | 869-19-2 | 2 | 8.59E+05 | 7.95E+05 | 8.34E+05 | 1.60E+05 | 1.95E+05 | 1.80E+05 | 1.65E+05 | 1.77E+05 | 1.74E+05 | 1.78E+05 | 1.72E+05 | 1.49E+05 | 3.36E+05 | 3.52E+05 | 4.56E+05 | C02155 | -- |
| mws5041 | 1.89E+02 | 8.61E+01 | 1.88E+02 | C_8_H_16_N_2_O_3_ | [M+H]+ | L-Glycyl-L-isoleucine* | Amino acids and derivatives | Amino acids and derivatives | 19461-38-2 | 2 | 5.89E+05 | 5.24E+05 | 6.67E+05 | 1.31E+05 | 1.58E+05 | 1.34E+05 | 1.11E+05 | 1.19E+05 | 1.48E+05 | 1.07E+05 | 9.88E+04 | 1.10E+05 | 2.44E+05 | 2.44E+05 | 3.09E+05 | -- | -- |
| pme0122 | 1.89E+02 | 1.26E+02 | 1.88E+02 | C_8_H_16_N_2_O_3_ | [M+H]+ | N6-Acetyl-L-lysine | Amino acids and derivatives | Amino acids and derivatives | 692-04-6 | 1 | 4.66E+06 | 4.60E+06 | 6.36E+06 | 1.93E+06 | 1.89E+06 | 1.82E+06 | 4.36E+06 | 3.72E+06 | 3.80E+06 | 3.80E+06 | 4.94E+06 | 3.86E+06 | 4.13E+06 | 3.79E+06 | 3.40E+06 | C02727 | ko00310,ko01100 |
| Zmjp000182 | 1.89E+02 | 7.01E+01 | 1.88E+02 | C_7_H_16_N_4_O_2_ | [M+H]+ | N-Monomethyl-L-arginine | Amino acids and derivatives | Amino acids and derivatives | 17035-90-4 | 1 | 9.75E+06 | 9.06E+06 | 9.89E+06 | 1.34E+07 | 1.26E+07 | 1.20E+07 | 1.09E+07 | 1.18E+07 | 1.08E+07 | 1.30E+07 | 1.36E+07 | 1.36E+07 | 1.16E+07 | 1.08E+07 | 1.17E+07 | C03884 | -- |
| pme3388 | 1.89E+02 | 1.44E+02 | 1.88E+02 | C_7_H_16_N_4_O_2_ | [M+H]+ | Homoarginine | Amino acids and derivatives | Amino acids and derivatives | 156-86-5 | 1 | 1.68E+06 | 1.55E+06 | 1.64E+06 | 1.87E+06 | 2.09E+06 | 1.86E+06 | 1.76E+06 | 1.68E+06 | 1.79E+06 | 2.14E+06 | 1.95E+06 | 2.17E+06 | 1.75E+06 | 1.89E+06 | 2.11E+06 | C01924 | -- |
| Zmdp000292 | 1.89E+02 | 7.01E+01 | 1.88E+02 | C_7_H_16_N_4_O_2_ | [M+H]+ | Arginine methyl ester | Amino acids and derivatives | Amino acids and derivatives | - | 1 | 9.72E+06 | 9.26E+06 | 9.74E+06 | 1.24E+07 | 1.25E+07 | 1.25E+07 | 1.08E+07 | 1.15E+07 | 1.12E+07 | 1.28E+07 | 1.30E+07 | 1.36E+07 | 1.12E+07 | 1.19E+07 | 1.26E+07 | -- | -- |
| Zmzp000145 | 1.89E+02 | 8.41E+01 | 1.88E+02 | C_9_H_20_N_2_O_2_ | [M+H]+ | Trimethyllysine | Amino acids and derivatives | Amino acids and derivatives | 23284-33-5 | 2 | 1.01E+07 | 1.06E+07 | 1.13E+07 | 3.37E+06 | 3.22E+06 | 3.79E+06 | 6.77E+06 | 7.78E+06 | 7.90E+06 | 7.35E+06 | 8.03E+06 | 8.57E+06 | 7.63E+06 | 7.31E+06 | 8.05E+06 | C03793 | ko00310,ko01100 |
| Zmtn001624 | 1.88E+02 | 1.44E+02 | 1.89E+02 | C_10_H_7_NO_3_ | [M-H]- | N-Acetylisatin | Alkaloids | Plumerane | 574-17-4 | 3 | 1.05E+05 | 8.36E+04 | 7.71E+04 | 2.01E+04 | 1.10E+04 | 2.13E+04 | 5.27E+04 | 3.74E+04 | 6.30E+04 | 2.94E+04 | 2.81E+04 | 1.57E+04 | 3.35E+04 | 3.07E+04 | 4.36E+04 | C02172 | ko00380 |
| pme0075 | 1.88E+02 | 1.28E+02 | 1.89E+02 | C_7_H_11_NO_5_ | [M-H]- | N-Acetyl-L-glutamic acid | Amino acids and derivatives | Amino acids and derivatives | 1188-37-0 | 3 | 2.61E+05 | 2.25E+05 | 2.41E+05 | 1.65E+05 | 1.52E+05 | 1.29E+05 | 1.56E+05 | 1.18E+05 | 9.72E+04 | 7.50E+04 | 7.32E+04 | 7.21E+04 | 1.03E+05 | 1.52E+05 | 1.74E+05 | C00624 | ko00220,ko01100,ko01110,ko01210,ko01230 |
| pme2244 | 1.90E+02 | 1.18E+02 | 1.89E+02 | C_11_H_11_NO_2_ | [M+H]+ | 3-Indolepropionic acid | Alkaloids | Plumerane | 830-96-6 | 2 | 5.32E+03 | 6.39E+03 | 6.12E+03 | 1.06E+04 | 5.57E+03 | 5.32E+03 | 2.47E+04 | 2.84E+04 | 3.37E+04 | 2.55E+04 | 1.38E+04 | 2.59E+04 | 2.12E+04 | 1.94E+04 | 3.21E+04 | -- | -- |
| mws0193 | 1.90E+02 | 1.27E+02 | 1.89E+02 | C_7_H_15_N_3_O_3_ | [M+H]+ | L-Homocitrulline | Amino acids and derivatives | Amino acids and derivatives | 1190-49-4 | 3 | 4.16E+05 | 4.28E+05 | 5.30E+05 | 1.72E+05 | 1.92E+05 | 1.17E+05 | 2.71E+05 | 2.97E+05 | 3.32E+05 | 3.13E+05 | 3.34E+05 | 3.93E+05 | 2.87E+05 | 3.43E+05 | 3.19E+05 | C02427 | -- |
| MWSslk069 | 1.91E+02 | 1.35E+02 | 1.90E+02 | C_11_H_10_O_3_ | [M+H]+ | 7-Methoxy-4-Methylcoumarin | Lignans and Coumarins | Coumarins | 2555-28-4 | 3 | 4.07E+03 | 1.99E+03 | 1.21E+03 | 5.11E+03 | 5.70E+03 | 3.40E+03 | 2.20E+03 | 2.36E+03 | 1.20E+03 | 2.26E+03 | 5.99E+03 | 4.27E+03 | 4.10E+03 | 2.77E+03 | 5.34E+03 | -- | -- |
| pme0278 | 1.91E+02 | 1.28E+02 | 1.90E+02 | C_7_H_14_N_2_O_4_ | [M+H]+ | 2,6-Diaminooimelic acid | Organic acids | Organic acids | 583-93-7 | 3 | 5.90E+04 | 8.80E+04 | 8.15E+04 | 4.58E+04 | 3.51E+04 | 2.31E+04 | 4.40E+04 | 5.61E+04 | 5.48E+04 | 3.97E+04 | 5.74E+04 | 4.07E+04 | 2.93E+04 | 5.19E+04 | 7.21E+04 | C00666 | ko00300,ko01100,ko01110,ko01230 |
| mws0281 | 1.91E+02 | 1.11E+02 | 1.92E+02 | C_6_H_8_O_7_ | [M-H]- | Citric Acid | Organic acids | Organic acids | 77-92-9 | 1 | 1.69E+07 | 1.74E+07 | 1.87E+07 | 2.40E+07 | 2.17E+07 | 2.25E+07 | 2.53E+07 | 2.43E+07 | 2.24E+07 | 2.78E+07 | 2.80E+07 | 3.17E+07 | 2.21E+07 | 2.73E+07 | 2.48E+07 | C00158 | ko00020,ko00250,ko00630,ko01100,ko01110,ko01200,ko01210,ko01230,ko01240 |
| Zmyn000453 | 1.91E+02 | 1.11E+02 | 1.92E+02 | C_6_H_8_O_7_ | [M-H]- | Isocitric Acid | Organic acids | Organic acids | 320-77-4 | 1 | 6.75E+06 | 7.09E+06 | 6.31E+06 | 9.22E+06 | 8.81E+06 | 9.85E+06 | 9.98E+06 | 9.96E+06 | 9.62E+06 | 1.31E+07 | 1.33E+07 | 1.32E+07 | 1.04E+07 | 9.64E+06 | 9.97E+06 | C00311 | ko00020,ko00630,ko01100,ko01110,ko01200,ko01210,ko01230,ko01240 |
| MWSmce221 | 1.93E+02 | 9.11E+01 | 1.92E+02 | C_10_H_8_O_4_ | [M+H]+ | 5,7-Dihydroxy-4-methylcoumarin | Lignans and Coumarins | Coumarins | 2107-76-8 | 3 | 2.24E+05 | 2.61E+05 | 2.18E+05 | 2.35E+05 | 2.53E+05 | 1.91E+05 | 6.96E+04 | 7.29E+04 | 5.25E+04 | 5.35E+04 | 6.28E+04 | 7.39E+04 | 1.54E+05 | 1.87E+05 | 1.73E+05 | -- | -- |
| MWSCX014 | 1.93E+02 | 1.33E+02 | 1.92E+02 | C_10_H_8_O_4_ | [M+H]+ | Scopoletin (7-Hydroxy-5-methoxycoumarin) | Lignans and Coumarins | Coumarins | 92-61-5 | 1 | 1.69E+07 | 1.68E+07 | 1.72E+07 | 1.80E+07 | 1.87E+07 | 1.66E+07 | 4.10E+06 | 4.03E+06 | 4.48E+06 | 4.30E+06 | 4.41E+06 | 4.63E+06 | 1.18E+07 | 1.16E+07 | 1.32E+07 | C01752 | ko00940,ko01110 |
| MWSmce159 | 1.93E+02 | 1.47E+02 | 1.92E+02 | C_10_H_8_O_4_ | [M+H]+ | 6,7-Dihydroxy-4-methylcoumarin | Lignans and Coumarins | Coumarins | 529-84-0 | 3 | 4.04E+04 | 5.41E+04 | 4.02E+04 | 3.96E+04 | 4.94E+04 | 4.72E+04 | 1.20E+04 | 1.84E+04 | 1.98E+04 | 1.41E+04 | 2.08E+04 | 2.26E+04 | 2.16E+04 | 2.61E+04 | 2.61E+04 | -- | -- |
| Hmcp002123 | 1.93E+02 | 1.78E+02 | 1.92E+02 | C_10_H_8_O_4_ | [M+H]+ | Isoscopoletin (6-Hydroxy-7-Methoxycoumarin) | Lignans and Coumarins | Coumarins | 776-86-3 | 1 | 1.64E+07 | 1.82E+07 | 1.79E+07 | 1.84E+07 | 1.90E+07 | 1.89E+07 | 4.49E+06 | 4.88E+06 | 5.03E+06 | 4.37E+06 | 4.04E+06 | 3.98E+06 | 1.20E+07 | 1.22E+07 | 1.24E+07 | C18079 | -- |
| MWSslk135 | 1.93E+02 | 1.47E+02 | 1.92E+02 | C_10_H_8_O_4_ | [M+H]+ | 7,8-Dihydroxy-4-methylcoumarin | Lignans and Coumarins | Coumarins | 2107-77-9 | 3 | 7.16E+04 | 8.46E+04 | 7.23E+04 | 9.00E+00 | 9.00E+00 | 9.00E+00 | 9.00E+00 | 9.00E+00 | 9.00E+00 | 9.00E+00 | 9.00E+00 | 9.00E+00 | 2.52E+04 | 2.42E+04 | 2.71E+04 | -- | -- |
| mws0277 | 1.91E+02 | 8.50E+01 | 1.92E+02 | C_7_H_12_O_6_ | [M-H]- | Quinic Acid | Organic acids | Organic acids | 77-95-2 | 1 | 5.43E+05 | 5.55E+05 | 4.55E+05 | 6.57E+05 | 7.51E+05 | 6.63E+05 | 5.68E+05 | 7.40E+05 | 6.76E+05 | 9.55E+05 | 1.02E+06 | 8.95E+05 | 7.39E+05 | 6.96E+05 | 6.94E+05 | C00296 | ko00400,ko01100 |
| pmp001227 | 1.93E+02 | 1.05E+02 | 1.92E+02 | C_12_H_16_O_2_ | [M+H]+ | Senkyunolide A | Others | Others | 63038-10-8 | 3 | 9.41E+04 | 8.69E+04 | 7.37E+04 | 9.98E+04 | 1.04E+05 | 1.07E+05 | 3.06E+04 | 2.52E+04 | 2.42E+04 | 3.09E+04 | 3.17E+04 | 3.45E+04 | 7.56E+04 | 6.88E+04 | 5.64E+04 | C17853 | -- |
| pme2743 | 1.94E+02 | 9.10E+01 | 1.93E+02 | C_10_H_11_NO_3_ | [M+H]+ | N-Phenylacetylglycine | Amino acids and derivatives | Amino acids and derivatives | 500-98-1 | 3 | 3.79E+04 | 3.54E+04 | 4.19E+04 | 9.00E+00 | 9.00E+00 | 9.00E+00 | 3.31E+04 | 4.52E+04 | 4.26E+04 | 9.00E+00 | 9.00E+00 | 9.00E+00 | 3.02E+04 | 2.48E+04 | 2.13E+04 | C05598 | ko00360 |
| mws1189 | 1.93E+02 | 7.28E+01 | 1.94E+02 | C_6_H_10_O_7_ | [M-H]- | D-Galacturonic acid | Others | Saccharides and Alcohols | 685-73-4 | 3 | 1.82E+05 | 1.42E+05 | 1.41E+05 | 1.65E+05 | 1.75E+05 | 1.93E+05 | 1.22E+05 | 1.10E+05 | 8.18E+04 | 1.29E+05 | 1.08E+05 | 1.03E+05 | 1.42E+05 | 1.31E+05 | 1.16E+05 | C00333 | ko00040,ko00053,ko00520,ko01100,ko01240,ko02010 |
| pme3705 | 1.93E+02 | 7.30E+01 | 1.94E+02 | C_6_H_10_O_7_ | [M-H]- | D-Glucoronic acid | Others | Saccharides and Alcohols | 6556-12-3 | 3 | 1.83E+05 | 1.86E+05 | 1.73E+05 | 1.95E+05 | 1.96E+05 | 2.22E+05 | 1.04E+05 | 1.04E+05 | 1.16E+05 | 1.50E+05 | 1.71E+05 | 1.56E+05 | 1.78E+05 | 1.66E+05 | 1.96E+05 | C00191 | ko00040,ko00053,ko00520,ko00562,ko01100,ko01240 |
| Xmgn006542 | 1.93E+02 | 1.33E+02 | 1.94E+02 | C_10_H_10_O_4_ | [M-H]- | Dimethyl Phthalate | Phenolic acids | Phenolic acids | 131-11-3 | 3 | 2.63E+05 | 2.24E+05 | 2.18E+05 | 7.86E+05 | 6.82E+05 | 6.91E+05 | 1.94E+06 | 1.91E+06 | 2.09E+06 | 1.83E+06 | 2.03E+06 | 1.87E+06 | 1.08E+06 | 1.20E+06 | 1.20E+06 | C11233 | -- |
| Lmdn003756 | 1.93E+02 | 1.35E+02 | 1.94E+02 | C_10_H_10_O_4_ | [M-H]- | Methyl caffeate | Phenolic acids | Phenolic acids | 3843-74-1 | 1 | 1.69E+04 | 1.16E+04 | 1.43E+04 | 1.29E+04 | 1.25E+04 | 1.08E+04 | 9.00E+00 | 9.00E+00 | 9.00E+00 | 7.82E+03 | 1.23E+04 | 4.87E+03 | 9.10E+03 | 6.58E+03 | 7.87E+03 | C10477 | -- |
| mws0014 | 1.93E+02 | 1.34E+02 | 1.94E+02 | C_10_H_10_O_4_ | [M-H]- | Ferulic acid | Phenolic acids | Phenolic acids | 537-98-4 | 1 | 1.45E+06 | 1.24E+06 | 1.42E+06 | 1.47E+06 | 1.31E+06 | 1.57E+06 | 2.87E+06 | 2.79E+06 | 4.55E+06 | 1.39E+06 | 1.38E+06 | 1.40E+06 | 1.49E+06 | 2.04E+06 | 2.15E+06 | C01494 | ko00940,ko01100,ko01110 |
| Lmrj002244 | 1.95E+02 | 7.01E+01 | 1.94E+02 | C_10_H_14_N_2_O_2_ | [M+H]+ | Cyclo(Pro-Pro) | Amino acids and derivatives | Amino acids and derivatives | 6708-06-1 | 3 | 6.07E+05 | 6.11E+05 | 6.10E+05 | 4.13E+04 | 3.05E+04 | 3.60E+04 | 1.93E+05 | 1.65E+05 | 1.49E+05 | 4.17E+04 | 3.95E+04 | 5.17E+04 | 2.28E+05 | 2.43E+05 | 1.86E+05 | -- | -- |
| Lmyp003317 | 1.96E+02 | 1.50E+02 | 1.95E+02 | C_9_H_9_NO_4_ | [M+H]+ | 6,7-Dimethoxy-2-benzoxazolinone (DMBOA) | Alkaloids | Alkaloids | 29550-09-2 | 2 | 6.07E+04 | 6.05E+04 | 9.34E+04 | 3.34E+04 | 2.97E+04 | 2.65E+04 | 1.62E+05 | 1.22E+05 | 1.38E+05 | 9.06E+04 | 9.19E+04 | 8.55E+04 | 7.99E+04 | 7.43E+04 | 7.72E+04 | -- | -- |
| pme0534 | 1.95E+02 | 7.49E+01 | 1.96E+02 | C_6_H_12_O_7_ | [M-H]- | Gluconic acid | Others | Saccharides and Alcohols | 526-95-4 | 1 | 5.67E+06 | 6.00E+06 | 5.33E+06 | 2.91E+06 | 2.66E+06 | 2.80E+06 | 3.34E+06 | 3.15E+06 | 3.35E+06 | 2.35E+06 | 2.48E+06 | 2.25E+06 | 3.04E+06 | 3.44E+06 | 3.37E+06 | C00257 | ko00030,ko01100,ko01110,ko01200 |
| Lmrj002698 | 1.97E+02 | 7.21E+01 | 1.96E+02 | C_10_H_16_N_2_O_2_ | [M+H]+ | Cyclo(Pro-Val) | Amino acids and derivatives | Amino acids and derivatives | 5654-87-5 | 3 | 2.62E+04 | 2.62E+04 | 2.84E+04 | 9.00E+00 | 9.00E+00 | 9.00E+00 | 9.00E+00 | 9.00E+00 | 9.00E+00 | 9.00E+00 | 9.00E+00 | 9.00E+00 | 1.36E+04 | 1.50E+04 | 1.19E+04 | -- | -- |
| pme3827 | 1.98E+02 | 1.52E+02 | 1.97E+02 | C_9_H_11_NO_4_ | [M+H]+ | 3,4-Dihydroxy-L-phenylalanine (L-Dopa) | Amino acids and derivatives | Amino acids and derivatives | 59-92-7 | 3 | 5.08E+04 | 3.95E+04 | 7.14E+04 | 2.41E+04 | 3.30E+04 | 2.02E+04 | 6.35E+04 | 3.05E+04 | 2.97E+04 | 4.79E+04 | 4.54E+04 | 9.02E+04 | 3.30E+04 | 4.28E+04 | 2.93E+04 | C00355 | ko00350,ko00950,ko00965,ko01100,ko01110 |
| pmb2497 | 1.97E+02 | 1.53E+02 | 1.98E+02 | C_9_H_10_O_5_ | [M-H]- | 4-Hydroxy-3-methoxymandelate | Phenolic acids | Phenolic acids | 55-10-7 | 3 | 6.11E+04 | 7.16E+04 | 7.13E+04 | 9.32E+04 | 9.05E+04 | 7.60E+04 | 8.48E+04 | 8.68E+04 | 8.67E+04 | 1.13E+05 | 8.99E+04 | 1.01E+05 | 8.04E+04 | 8.60E+04 | 8.16E+04 | C05584 | ko00350,ko01100 |
| Zmzn000079 | 1.99E+02 | 7.90E+01 | 2.00E+02 | C_4_H_9_O_7_P | [M-H]- | D-Erythrose-4-phosphate | Others | Saccharides and Alcohols | 585-18-2 | 3 | 1.14E+04 | 8.46E+03 | 1.50E+04 | 4.96E+04 | 4.67E+04 | 6.07E+04 | 4.15E+04 | 4.12E+04 | 3.65E+04 | 5.12E+04 | 4.19E+04 | 5.26E+04 | 3.37E+04 | 3.63E+04 | 4.13E+04 | C00279 | ko00030,ko00400,ko00710,ko00750,ko00998,ko01100,ko01110,ko01200,ko01230,ko01240 |
| pmb2640 | 1.99E+02 | 1.99E+02 | 2.00E+02 | C_12_H_24_O_2_ | [M-H]- | Dodecanoic acid (Lauric acid) | Lipids | Free fatty acids | 143-07-7 | 3 | 1.73E+05 | 1.62E+05 | 1.67E+05 | 1.07E+05 | 9.11E+04 | 8.20E+04 | 7.59E+04 | 7.32E+04 | 7.45E+04 | 6.94E+04 | 7.39E+04 | 7.86E+04 | 1.00E+05 | 1.13E+05 | 1.11E+05 | C02679 | ko00061,ko01100,ko01110 |
| Zmnn006956 | 2.01E+02 | 1.17E+02 | 2.02E+02 | C_11_H_6_O_4_ | [M-H]- | Bergaptol | Lignans and Coumarins | Coumarins | 486-60-2 | 1 | 9.79E+06 | 9.69E+06 | 9.76E+06 | 5.40E+06 | 5.14E+06 | 5.22E+06 | 7.61E+06 | 7.21E+06 | 7.78E+06 | 4.45E+06 | 4.64E+06 | 4.78E+06 | 6.82E+06 | 7.40E+06 | 7.25E+06 | C00758 | -- |
| MWSmce388 | 2.03E+02 | 1.47E+02 | 2.02E+02 | C_11_H_6_O_4_ | [M+H]+ | Xanthotoxol | Lignans and Coumarins | Coumarins | 2009-24-7 | 2 | 1.31E+07 | 1.12E+07 | 1.17E+07 | 5.41E+06 | 5.12E+06 | 5.53E+06 | 9.71E+06 | 9.25E+06 | 1.00E+07 | 4.28E+06 | 4.68E+06 | 4.40E+06 | 8.28E+06 | 7.91E+06 | 8.61E+06 | C00841 | -- |
| pmb0130 | 2.03E+02 | 1.44E+02 | 2.02E+02 | C_12_H_14_N_2_O | [M+H]+ | Acetryptine | Alkaloids | Plumerane | 3551-18-6 | 3 | 2.73E+05 | 2.29E+05 | 5.13E+05 | 9.00E+00 | 9.00E+00 | 9.00E+00 | 9.00E+00 | 9.00E+00 | 9.00E+00 | 9.00E+00 | 9.00E+00 | 9.00E+00 | 1.05E+05 | 9.63E+04 | 1.10E+05 | -- | -- |
| pme0266 | 2.01E+02 | 1.39E+02 | 2.02E+02 | C_10_H_18_O_4_ | [M-H]- | Sebacate | Organic acids | Organic acids | 111-20-6 | 3 | 4.23E+03 | 4.35E+03 | 3.31E+03 | 3.05E+03 | 3.69E+03 | 1.64E+03 | 2.94E+03 | 1.18E+04 | 2.15E+03 | 3.82E+03 | 3.48E+03 | 3.48E+03 | 4.81E+03 | 3.05E+03 | 4.02E+03 | C08277 | -- |
| NK10251888 | 2.03E+02 | 7.01E+01 | 2.02E+02 | C_8_H_18_N_4_O_2_ | [M+H]+ | NG,NG-Dimethyl-L-arginine | Amino acids and derivatives | Amino acids and derivatives | 30315-93-6 | 1 | 6.65E+06 | 7.04E+06 | 8.68E+06 | 2.55E+06 | 2.38E+06 | 2.32E+06 | 3.68E+06 | 3.70E+06 | 3.21E+06 | 2.02E+06 | 2.26E+06 | 2.18E+06 | 3.80E+06 | 3.92E+06 | 4.02E+06 | C03626 | -- |
| mws0018 | 2.03E+02 | 8.30E+01 | 2.02E+02 | C_10_H_26_N_4_ | [M+H]+ | Spermine | Alkaloids | Alkaloids | 71-44-3 | 3 | 5.59E+06 | 6.11E+06 | 6.56E+06 | 7.79E+06 | 7.82E+06 | 7.85E+06 | 8.01E+06 | 9.30E+06 | 7.56E+06 | 8.67E+06 | 9.29E+06 | 8.54E+06 | 7.00E+06 | 8.28E+06 | 8.14E+06 | C00750 | ko00330,ko00410,ko00480,ko00770,ko01100,ko01240 |
| pme2060 | 2.04E+02 | 1.36E+02 | 2.03E+02 | C_10_H_13_N_5_ | [M+H]+ | N6-Isopentenyladenine | Nucleotides and derivatives | Nucleotides and derivatives | 2365-40-4 | 3 | 1.45E+05 | 1.56E+05 | 1.68E+05 | 9.00E+00 | 9.00E+00 | 9.00E+00 | 9.00E+00 | 9.00E+00 | 9.00E+00 | 9.00E+00 | 9.00E+00 | 9.00E+00 | 5.79E+04 | 4.84E+04 | 3.52E+04 | C04083 | ko00908,ko01110,ko04075 |
| Zmbp002538 | 2.05E+02 | 1.46E+02 | 2.04E+02 | C_11_H_12_N_2_O_2_ | [M+H]+ | 1-Methoxy-indole-3-acetamide | Alkaloids | Plumerane | - | 1 | 3.49E+06 | 3.61E+06 | 3.59E+06 | 5.21E+06 | 5.15E+06 | 5.09E+06 | 1.02E+07 | 1.04E+07 | 1.07E+07 | 1.18E+07 | 1.27E+07 | 1.24E+07 | 7.81E+06 | 7.48E+06 | 8.61E+06 | -- | -- |
| mws0282 | 2.03E+02 | 1.16E+02 | 2.04E+02 | C_11_H_12_N_2_O_2_ | [M-H]- | L-Tryptophan | Amino acids and derivatives | Amino acids and derivatives | 73-22-3 | 1 | 1.46E+07 | 1.48E+07 | 1.53E+07 | 2.04E+07 | 2.18E+07 | 2.29E+07 | 3.58E+07 | 3.62E+07 | 3.42E+07 | 4.37E+07 | 4.24E+07 | 4.09E+07 | 2.56E+07 | 2.92E+07 | 3.06E+07 | C00078 | ko00260,ko00380,ko00400,ko00901,ko00966,ko00970,ko00998,ko01100,ko01110,ko01210,ko01230,ko01240 |
| Zmtn001464 | 2.04E+02 | 1.60E+02 | 2.05E+02 | C_10_H_7_NO_4_ | [M-H]- | 4,8-Dihydroxyquinoline-2-carboxylic acid | Organic acids | Organic acids | 59-00-7 | 3 | 1.16E+05 | 9.97E+04 | 1.10E+05 | 1.66E+05 | 1.62E+05 | 1.71E+05 | 3.38E+05 | 3.32E+05 | 3.46E+05 | 3.89E+05 | 3.85E+05 | 3.97E+05 | 2.48E+05 | 2.85E+05 | 3.02E+05 | C02470 | ko00380 |
| pmb0818 | 2.06E+02 | 1.48E+02 | 2.05E+02 | C_11_H_11_NO_3_ | [M+H]+ | Methoxyindoleacetic acid | Alkaloids | Plumerane | 3471-31-6 | 3 | 7.57E+05 | 6.96E+05 | 6.92E+05 | 1.09E+06 | 1.05E+06 | 1.13E+06 | 2.22E+06 | 2.05E+06 | 2.18E+06 | 2.71E+06 | 2.98E+06 | 2.78E+06 | 1.74E+06 | 1.67E+06 | 1.73E+06 | C05660 | ko00380 |
| pme1261 | 2.06E+02 | 7.60E+01 | 2.05E+02 | C_9_H_19_NO_4_ | [M+H]+ | D-Panthenol | Others | Saccharides and Alcohols | 81-13-0 | 3 | 7.39E+03 | 8.99E+03 | 1.64E+04 | 1.60E+04 | 1.17E+04 | 1.52E+04 | 9.00E+00 | 9.00E+00 | 9.00E+00 | 9.00E+00 | 9.00E+00 | 9.00E+00 | 5.89E+03 | 6.02E+03 | 9.38E+03 | C05944 | ko00770 |
| MWSmce082 | 2.07E+02 | 1.51E+02 | 2.06E+02 | C_11_H_10_O_4_ | [M+H]+ | Scoparone | Lignans and Coumarins | Coumarins | 120-08-1 | 1 | 9.42E+05 | 1.26E+06 | 9.94E+05 | 2.24E+05 | 2.26E+05 | 2.16E+05 | 1.64E+05 | 1.75E+05 | 1.84E+05 | 2.85E+04 | 2.66E+04 | 3.74E+04 | 4.22E+05 | 4.24E+05 | 4.67E+05 | C09311 | -- |
| mws4173 | 2.07E+02 | 1.92E+02 | 2.06E+02 | C_11_H_10_O_4_ | [M+H]+ | 5,7-Dimethoxycoumarin (Limettin)(Citropten) | Lignans and Coumarins | Coumarins | 487-06-9 | 2 | 3.61E+04 | 3.14E+04 | 4.22E+04 | 3.85E+04 | 4.34E+04 | 4.31E+04 | 3.16E+04 | 3.01E+04 | 2.52E+04 | 4.55E+04 | 4.56E+04 | 5.01E+04 | 3.53E+04 | 3.73E+04 | 3.09E+04 | -- | -- |
| pmp001236 | 2.07E+02 | 1.21E+02 | 2.06E+02 | C_11_H_10_O_4_ | [M+H]+ | Clove chromone | Others | Others | - | 3 | 2.04E+04 | 2.21E+04 | 2.82E+04 | 2.71E+04 | 3.51E+04 | 2.43E+04 | 1.95E+04 | 1.72E+04 | 1.01E+04 | 3.03E+04 | 2.94E+04 | 3.38E+04 | 2.14E+04 | 2.16E+04 | 2.38E+04 | -- | -- |
| Lmjp002718 | 2.07E+02 | 9.51E+01 | 2.06E+02 | C_13_H_18_O_2_ | [M+H]+ | Arteannuin A | Terpenoids | Sesquiterpenoids | 82442-48-6 | 2 | 8.33E+05 | 4.96E+05 | 4.66E+05 | 9.59E+05 | 8.25E+05 | 8.09E+05 | 8.95E+05 | 7.01E+05 | 8.90E+05 | 1.01E+06 | 1.02E+06 | 1.05E+06 | 8.56E+05 | 1.17E+06 | 1.19E+06 | -- | -- |
| Lmbn013410 | 2.05E+02 | 1.89E+02 | 2.06E+02 | C_14_H_22_O | [M-H]- | 2,4-Di-Tert-Butylphenol* | Phenolic acids | Phenolic acids | 96-76-4 | 1 | 1.02E+06 | 1.09E+06 | 8.88E+05 | 1.19E+06 | 1.16E+06 | 1.22E+06 | 1.09E+06 | 9.65E+05 | 9.76E+05 | 1.17E+06 | 1.23E+06 | 1.24E+06 | 8.97E+05 | 9.26E+05 | 9.67E+05 | -- | -- |
| Lmln010063 | 2.05E+02 | 1.89E+02 | 2.06E+02 | C_14_H_22_O | [M-H]- | 2,6-Di-tert-butylphenol* | Phenolic acids | Phenolic acids | 128-39-2 | 1 | 1.11E+06 | 1.15E+06 | 9.81E+05 | 1.30E+06 | 1.23E+06 | 1.13E+06 | 1.15E+06 | 1.07E+06 | 1.04E+06 | 1.22E+06 | 1.25E+06 | 1.22E+06 | 8.62E+05 | 9.85E+05 | 1.04E+06 | -- | -- |
| MWS1877 | 2.05E+02 | 1.33E+02 | 2.06E+02 | C_14_H_22_O | [M-H]- | 4-tert-Octylphenol | Phenolic acids | Phenolic acids | 140-66-9 | 1 | 6.21E+03 | 5.01E+03 | 4.03E+03 | 5.01E+03 | 6.04E+03 | 6.26E+03 | 5.82E+03 | 2.86E+03 | 4.71E+03 | 6.54E+03 | 3.64E+03 | 5.54E+03 | 6.06E+03 | 5.93E+03 | 4.82E+03 | C14205 | -- |
| MWS1961 | 2.05E+02 | 1.89E+02 | 2.06E+02 | C_13_H_22_N_2_ | [M-H]- | N,N'-Dicyclohexylcarbodiimide | Others | Others | 538-75-0 | 1 | 1.13E+06 | 1.19E+06 | 1.08E+06 | 1.24E+06 | 1.27E+06 | 1.19E+06 | 1.20E+06 | 1.12E+06 | 1.08E+06 | 1.19E+06 | 1.26E+06 | 1.31E+06 | 9.04E+05 | 9.48E+05 | 9.62E+05 | -- | -- |
| Zmgn002106 | 2.06E+02 | 5.80E+01 | 2.07E+02 | C_11_H_13_NO_3_ | [M-H]- | N-Acetyl-L-phenylalanine | Amino acids and derivatives | Amino acids and derivatives | 2018-61-3 | 1 | 6.14E+04 | 4.68E+04 | 6.42E+04 | 9.00E+00 | 9.00E+00 | 9.00E+00 | 7.22E+04 | 7.73E+04 | 8.04E+04 | 9.00E+00 | 9.00E+00 | 9.00E+00 | 4.16E+04 | 3.70E+04 | 3.40E+04 | C03519 | ko00360,ko01100 |
| mws1014 | 2.09E+02 | 1.49E+02 | 2.08E+02 | C_10_H_8_O_5_ | [M+H]+ | Fraxetin (7,8-Dihydroxy-6-methoxycoumarin) | Lignans and Coumarins | Coumarins | 574-84-5 | 2 | 6.82E+04 | 4.36E+04 | 2.46E+04 | 6.33E+04 | 7.81E+04 | 6.96E+04 | 4.30E+04 | 4.19E+04 | 7.53E+04 | 1.21E+05 | 8.04E+04 | 6.87E+04 | 6.05E+04 | 9.21E+04 | 7.00E+04 | C09265 | -- |
| Lmbp002962 | 2.09E+02 | 1.94E+02 | 2.08E+02 | C_10_H_8_O_5_ | [M+H]+ | Isofraxetin | Lignans and Coumarins | Coumarins | 50656-75-2 | 1 | 9.09E+04 | 7.94E+04 | 5.40E+04 | 4.56E+05 | 4.31E+05 | 2.81E+05 | 8.73E+04 | 7.62E+04 | 1.22E+05 | 8.50E+04 | 1.17E+05 | 1.02E+05 | 1.81E+05 | 1.25E+05 | 1.59E+05 | -- | -- |
| pmb2620 | 2.07E+02 | 1.33E+02 | 2.08E+02 | C_11_H_12_O_4_ | [M-H]- | 3,4-Dimethoxycinnamic acid | Phenolic acids | Phenolic acids | 14737-89-4 | 3 | 2.63E+04 | 1.85E+04 | 2.75E+04 | 3.38E+04 | 3.73E+04 | 3.73E+04 | 6.18E+04 | 5.91E+04 | 6.32E+04 | 5.12E+04 | 5.61E+04 | 5.11E+04 | 4.15E+04 | 3.64E+04 | 4.46E+04 | -- | -- |
| mws1212 | 2.09E+02 | 1.77E+02 | 2.08E+02 | C_11_H_12_O_4_ | [M+H]+ | Ferulic acid methyl ester | Phenolic acids | Phenolic acids | 2309-07-1 | 3 | 5.23E+04 | 3.46E+04 | 3.32E+04 | 5.17E+04 | 5.36E+04 | 6.32E+04 | 1.04E+05 | 9.90E+04 | 1.16E+05 | 1.03E+05 | 1.09E+05 | 1.05E+05 | 6.71E+04 | 5.98E+04 | 8.01E+04 | -- | -- |
| pmp001229 | 2.09E+02 | 1.25E+02 | 2.08E+02 | C_12_H_16_O_3_ | [M+H]+ | Senkyunolide K | Others | Others | 114569-33-4 | 3 | 6.02E+03 | 7.69E+03 | 5.32E+03 | 2.14E+03 | 3.42E+03 | 4.66E+03 | 9.00E+00 | 9.00E+00 | 9.00E+00 | 9.00E+00 | 9.00E+00 | 9.00E+00 | 2.51E+03 | 2.66E+03 | 2.29E+03 | -- | -- |
| Zmpn000199 | 2.09E+02 | 8.50E+01 | 2.10E+02 | C_6_H_10_O_8_ | [M-H]- | D-Galactaric acid | Others | Saccharides and Alcohols | 526-99-8 | 2 | 3.65E+06 | 3.93E+06 | 3.72E+06 | 3.65E+06 | 3.36E+06 | 3.56E+06 | 3.82E+06 | 4.31E+06 | 4.03E+06 | 3.72E+06 | 4.04E+06 | 3.55E+06 | 3.25E+06 | 3.92E+06 | 3.78E+06 | C00879 | ko00053,ko01100 |
| Zmyn000108 | 2.09E+02 | 8.50E+01 | 2.10E+02 | C_6_H_10_O_8_ | [M-H]- | D-Saccharic acid | Others | Saccharides and Alcohols | 87-73-0 | 2 | 3.82E+06 | 4.39E+06 | 4.11E+06 | 3.76E+06 | 3.64E+06 | 4.10E+06 | 4.47E+06 | 3.99E+06 | 3.93E+06 | 3.71E+06 | 4.18E+06 | 4.25E+06 | 3.65E+06 | 3.94E+06 | 4.23E+06 | C00818 | ko00053,ko01100 |
| Hmcn000192 | 2.09E+02 | 5.90E+01 | 2.10E+02 | C_7_H_14_O_7_ | [M-H]- | Sedoheptulose | Others | Saccharides and Alcohols | 3019-74-7 | 2 | 1.58E+06 | 1.74E+06 | 1.64E+06 | 1.46E+06 | 1.40E+06 | 1.60E+06 | 1.76E+06 | 1.67E+06 | 1.78E+06 | 1.45E+06 | 1.65E+06 | 1.60E+06 | 1.50E+06 | 1.55E+06 | 1.75E+06 | C02076 | ko00710,ko01100 |
| Hmcp003783 | 2.11E+02 | 1.93E+02 | 2.10E+02 | C_13_H_10_N_2_O | [M+H]+ | 1-Acetyl-β-carboline | Alkaloids | Plumerane | - | 1 | 6.67E+04 | 6.39E+04 | 6.42E+04 | 2.22E+04 | 1.80E+04 | 1.70E+04 | 1.51E+05 | 1.55E+05 | 1.50E+05 | 6.10E+04 | 6.30E+04 | 6.88E+04 | 7.30E+04 | 6.32E+04 | 7.69E+04 | -- | -- |
| pme1654 | 2.09E+02 | 5.90E+01 | 2.10E+02 | C_12_H_18_O_3_ | [M-H]- | Jasmonic acid | Organic acids | Organic acids | 77026-92-7 | 3 | 2.08E+05 | 2.23E+05 | 1.83E+05 | 4.15E+05 | 4.52E+05 | 4.25E+05 | 2.35E+05 | 2.13E+05 | 2.09E+05 | 2.95E+05 | 2.83E+05 | 2.74E+05 | 2.72E+05 | 2.88E+05 | 3.08E+05 | C08491 | ko00592,ko01100,ko01110,ko04075 |
| Lmhp002764 | 2.11E+02 | 7.01E+01 | 2.10E+02 | C_11_H_18_N_2_O_2_ | [M+H]+ | Cyclo(Pro-Leu) | Amino acids and derivatives | Amino acids and derivatives | 5654-86-4 | 3 | 9.15E+04 | 1.09E+05 | 8.27E+04 | 9.00E+00 | 9.00E+00 | 9.00E+00 | 3.86E+04 | 4.18E+04 | 1.13E+04 | 9.00E+00 | 9.00E+00 | 9.00E+00 | 4.60E+04 | 4.79E+04 | 6.22E+04 | -- | -- |
| pmb2507 | 2.13E+02 | 9.70E+01 | 2.14E+02 | C_5_H_11_O_7_P | [M-H]- | 2-Deoxyribose-1-phosphate | Nucleotides and derivatives | Nucleotides and derivatives | 17210-42-3 | 3 | 1.20E+07 | 1.20E+07 | 1.27E+07 | 2.36E+06 | 2.43E+06 | 2.33E+06 | 1.15E+07 | 1.09E+07 | 1.09E+07 | 4.44E+06 | 3.91E+06 | 3.07E+06 | 6.23E+06 | 7.18E+06 | 7.48E+06 | C00672 | ko00030,ko00240,ko01100 |
| mws0863 | 2.13E+02 | 9.70E+01 | 2.14E+02 | C_5_H_11_O_7_P | [M-H]- | 2-Deoxyribose-5'-phosphate | Nucleotides and derivatives | Nucleotides and derivatives | 102916-66-5 | 3 | 2.76E+05 | 3.49E+05 | 2.51E+05 | 7.51E+04 | 1.24E+05 | 9.13E+04 | 1.47E+05 | 2.06E+05 | 2.01E+05 | 7.09E+04 | 7.35E+04 | 6.12E+04 | 1.85E+05 | 1.27E+05 | 1.60E+05 | C00673 | ko00030,ko01100 |
| mws1579 | 2.17E+02 | 2.02E+02 | 2.16E+02 | C_12_H_8_O_4_ | [M+H]+ | 8-Methoxypsoralen | Lignans and Coumarins | Coumarins | 298-81-7 | 2 | 3.27E+06 | 4.03E+06 | 3.68E+06 | 3.12E+06 | 2.80E+06 | 2.81E+06 | 5.95E+05 | 5.19E+05 | 5.74E+05 | 5.76E+05 | 5.45E+05 | 6.32E+05 | 2.05E+06 | 2.39E+06 | 2.21E+06 | C01864 | -- |
| MWSmce042 | 2.17E+02 | 2.02E+02 | 2.16E+02 | C_12_H_8_O_4_ | [M+H]+ | Bergapten | Lignans and Coumarins | Coumarins | 484-20-8 | 2 | 1.05E+07 | 9.94E+06 | 9.04E+06 | 1.02E+07 | 9.91E+06 | 9.95E+06 | 6.42E+06 | 6.16E+06 | 6.49E+06 | 6.80E+06 | 6.68E+06 | 6.73E+06 | 8.51E+06 | 8.13E+06 | 8.37E+06 | C01557 | ko01110 |
| pme0170 | 2.17E+02 | 1.58E+02 | 2.16E+02 | C_8_H_16_N_4_O_3_ | [M+H]+ | N-Acetyl-L-Arginine | Amino acids and derivatives | Amino acids and derivatives | 155-84-0 | 2 | 7.03E+06 | 6.74E+06 | 7.10E+06 | 3.94E+05 | 3.98E+05 | 4.58E+05 | 7.94E+06 | 7.77E+06 | 7.03E+06 | 4.04E+05 | 4.61E+05 | 3.86E+05 | 3.58E+06 | 3.96E+06 | 4.13E+06 | -- | -- |
| MWS1900 | 2.15E+02 | 1.53E+02 | 2.16E+02 | C_11_H_20_O_4_ | [M-H]- | Undecanedioic acid | Lipids | Free fatty acids | 1852-04-6 | 1 | 4.57E+04 | 5.41E+04 | 6.45E+04 | 8.69E+04 | 9.18E+04 | 9.40E+04 | 3.06E+04 | 2.96E+04 | 2.41E+04 | 2.85E+04 | 1.78E+04 | 2.78E+04 | 4.83E+04 | 6.17E+04 | 6.68E+04 | -- | -- |
| mws5045 | 2.15E+02 | 1.69E+02 | 2.16E+02 | C_12_H_24_O_3_ | [M-H]- | 12-Hydroxydodecanoic acid | Lipids | Free fatty acids | 505-95-3 | 2 | 5.97E+03 | 9.18E+03 | 8.97E+03 | 4.45E+03 | 6.27E+03 | 4.00E+03 | 4.81E+03 | 3.69E+03 | 4.59E+03 | 4.95E+03 | 3.83E+03 | 3.36E+03 | 6.19E+03 | 4.56E+03 | 7.45E+03 | C08317 | -- |
| MWSmce184 | 2.19E+02 | 1.73E+02 | 2.18E+02 | C_12_H_10_O_4_ | [M+H]+ | Ethyl 3-coumarincarboxylate | Lignans and Coumarins | Coumarins | 1846-76-0 | 3 | 1.43E+05 | 1.30E+05 | 1.25E+05 | 3.30E+04 | 3.23E+04 | 3.28E+04 | 7.26E+04 | 7.46E+04 | 8.65E+04 | 2.08E+04 | 2.07E+04 | 2.52E+04 | 5.31E+04 | 6.30E+04 | 7.44E+04 | -- | -- |
| pme2566 | 2.17E+02 | 1.99E+02 | 2.18E+02 | C_8_H_14_N_2_O_5_ | [M-H]- | 5-L-Glutamyl-L-amino acid | Amino acids and derivatives | Amino acids and derivatives | 5875-41-2 | 3 | 1.65E+04 | 2.38E+04 | 1.38E+04 | 1.24E+04 | 1.02E+04 | 1.48E+04 | 1.29E+04 | 2.07E+04 | 1.02E+04 | 1.34E+04 | 2.18E+04 | 1.73E+04 | 9.55E+03 | 1.21E+04 | 1.67E+04 | -- | -- |
| Smjp001258 | 2.19E+02 | 1.32E+02 | 2.18E+02 | C_12_H_14_N_2_O_2_ | [M+H]+ | Abrine | Alkaloids | Plumerane | 526-31-8 | 2 | 2.20E+03 | 1.76E+04 | 8.05E+03 | 2.16E+04 | 1.54E+04 | 1.81E+04 | 4.55E+04 | 6.34E+04 | 5.66E+04 | 5.14E+04 | 5.00E+04 | 3.62E+04 | 2.46E+04 | 1.96E+04 | 2.12E+04 | C02983 | -- |
| mws0677 | 2.19E+02 | 1.60E+02 | 2.18E+02 | C_12_H_14_N_2_O_2_ | [M+H]+ | N-Acetyl-5-hydroxytryptamine | Alkaloids | Plumerane | 1210-83-9 | 2 | 5.03E+04 | 3.98E+04 | 3.49E+04 | 3.68E+04 | 4.21E+04 | 6.50E+04 | 1.34E+05 | 1.50E+05 | 1.31E+05 | 9.54E+04 | 9.00E+04 | 8.99E+04 | 5.29E+04 | 9.35E+04 | 6.65E+04 | C00978 | ko00380,ko01100,ko01110 |
| MWSmce542 | 2.19E+02 | 1.32E+02 | 2.18E+02 | C_12_H_14_N_2_O_2_ | [M+H]+ | (S)-Indoximod | Alkaloids | Plumerane | 21339-55-9 | 2 | 6.90E+03 | 1.05E+04 | 1.34E+04 | 2.14E+04 | 1.82E+04 | 1.56E+04 | 3.95E+04 | 2.67E+04 | 2.79E+04 | 3.84E+04 | 3.46E+04 | 2.64E+04 | 2.35E+04 | 2.00E+04 | 2.41E+04 | -- | -- |
| mws1337 | 2.20E+02 | 2.02E+02 | 2.19E+02 | C_9_H_17_NO_5_ | [M+H]+ | D-Pantothenic Acid | Others | Vitamin | 79-83-4 | 1 | 1.77E+06 | 1.86E+06 | 1.82E+06 | 3.09E+06 | 2.74E+06 | 2.96E+06 | 2.06E+06 | 2.00E+06 | 2.11E+06 | 2.59E+06 | 2.56E+06 | 2.38E+06 | 2.05E+06 | 2.09E+06 | 2.38E+06 | C00864 | ko00410,ko00770,ko01100,ko01110,ko01240 |
| mws2608 | 2.22E+02 | 8.41E+01 | 2.21E+02 | C_8_H_15_NO_6_ | [M+H]+ | N-Acetyl-D-galactosamine | Others | Saccharides and Alcohols | 1811-31-0 | 3 | 8.62E+04 | 7.25E+04 | 1.00E+05 | 5.71E+04 | 6.43E+04 | 6.01E+04 | 6.03E+04 | 8.08E+04 | 4.27E+04 | 6.29E+04 | 7.60E+04 | 4.95E+04 | 5.32E+04 | 4.92E+04 | 8.53E+04 | C01132 | ko00052,ko01100 |
| pme2755 | 2.22E+02 | 1.44E+02 | 2.21E+02 | C_8_H_15_NO_6_ | [M+H]+ | N-Acetyl-D-glucosamine | Others | Saccharides and Alcohols | 7512-17-6 | 3 | 1.18E+05 | 1.05E+05 | 1.32E+05 | 1.33E+05 | 1.72E+05 | 2.01E+05 | 2.01E+05 | 1.90E+05 | 1.66E+05 | 1.67E+05 | 1.48E+05 | 1.85E+05 | 1.25E+05 | 1.78E+05 | 2.02E+05 | C00140 | ko00520,ko01100,ko02010 |
| mws4174 | 2.22E+02 | 1.38E+02 | 2.21E+02 | C_8_H_15_NO_6_ | [M+H]+ | N-Acetyl-D-mannosamine | Others | Saccharides and Alcohols | 7772-94-3 | 3 | 4.98E+04 | 4.17E+04 | 4.17E+04 | 1.08E+04 | 1.51E+04 | 8.11E+03 | 2.20E+04 | 2.88E+04 | 1.76E+04 | 2.82E+04 | 1.64E+04 | 1.45E+04 | 1.77E+04 | 2.49E+04 | 2.58E+04 | C00645 | ko00520,ko01100 |
| mws1639 | 2.23E+02 | 1.90E+02 | 2.22E+02 | C_11_H_10_O_5_ | [M+H]+ | Isofraxidin | Lignans and Coumarins | Coumarins | 486-21-5 | 1 | 1.25E+06 | 1.15E+06 | 1.17E+06 | 8.03E+05 | 8.15E+05 | 8.89E+05 | 3.89E+05 | 4.33E+05 | 4.73E+05 | 5.35E+05 | 4.70E+05 | 4.47E+05 | 7.78E+05 | 7.87E+05 | 8.50E+05 | C17480 | -- |
| pmp000284 | 2.23E+02 | 2.08E+02 | 2.22E+02 | C_11_H_10_O_5_ | [M+H]+ | Fraxidin (8-Hydroxy-6,7-dimethoxycoumarin) | Lignans and Coumarins | Coumarins | 525-21-3 | 1 | 4.63E+05 | 3.92E+05 | 4.05E+05 | 2.74E+05 | 2.79E+05 | 2.66E+05 | 1.60E+05 | 1.52E+05 | 1.43E+05 | 1.51E+05 | 1.88E+05 | 2.03E+05 | 2.74E+05 | 2.75E+05 | 2.73E+05 | C17479 | -- |
| Lmjp002764 | 2.23E+02 | 1.90E+02 | 2.22E+02 | C_11_H_10_O_5_ | [M+H]+ | Umckalin (7-hydroxy-5,6-dimethoxycoumarin) | Lignans and Coumarins | Coumarins | 43053-62-9 | 2 | 8.89E+05 | 2.37E+05 | 5.55E+05 | 1.16E+06 | 1.15E+06 | 8.79E+05 | 4.40E+05 | 3.37E+05 | 6.81E+05 | 2.28E+05 | 2.04E+05 | 2.07E+05 | 6.58E+05 | 6.06E+05 | 5.37E+05 | -- | -- |
| MWSmce177 | 2.21E+02 | 1.33E+02 | 2.22E+02 | C_12_H_14_O_4_ | [M-H]- | Ethyl ferulate | Phenolic acids | Phenolic acids | 4046-02-0 | 1 | 1.36E+04 | 1.49E+04 | 1.65E+04 | 3.43E+04 | 2.79E+04 | 3.74E+04 | 4.88E+04 | 5.02E+04 | 5.79E+04 | 1.00E+04 | 1.02E+04 | 1.13E+04 | 2.56E+04 | 2.63E+04 | 3.11E+04 | -- | -- |
| MWS4296 | 2.23E+02 | 1.20E+02 | 2.22E+02 | C_11_H_14_N_2_O_3_ | [M+H]+ | Glycylphenylalanine | Amino acids and derivatives | Amino acids and derivatives | 3321-03-7 | 1 | 3.07E+05 | 2.62E+05 | 3.68E+05 | 3.99E+04 | 6.06E+04 | 7.26E+04 | 6.87E+04 | 7.51E+04 | 7.92E+04 | 7.16E+04 | 7.45E+04 | 6.75E+04 | 1.44E+05 | 1.04E+05 | 1.08E+05 | -- | -- |
| mws0520 | 2.24E+02 | 1.36E+02 | 2.23E+02 | C_11_H_13_NO_4_ | [M+H]+ | N-Acetyl-L-tyrosine | Amino acids and derivatives | Amino acids and derivatives | 537-55-3 | 3 | 2.38E+05 | 2.76E+05 | 2.62E+05 | 2.21E+04 | 1.84E+04 | 2.53E+04 | 3.64E+05 | 3.86E+05 | 3.80E+05 | 5.41E+04 | 4.61E+04 | 3.89E+04 | 1.91E+05 | 2.16E+05 | 2.09E+05 | -- | -- |
| Zmln002252 | 2.23E+02 | 1.49E+02 | 2.24E+02 | C_10_H_8_O_6_ | [M-H]- | Sideretin (5,7,8-Trihydroxy-6-methoxycoumarin) | Lignans and Coumarins | Coumarins | - | 3 | 6.64E+04 | 4.40E+04 | 4.08E+04 | 1.70E+04 | 1.43E+04 | 1.58E+04 | 9.00E+00 | 9.00E+00 | 9.00E+00 | 9.00E+00 | 9.00E+00 | 9.00E+00 | 1.93E+04 | 2.22E+04 | 3.05E+04 | C22007 | -- |
| mws4085 | 2.23E+02 | 1.93E+02 | 2.24E+02 | C_11_H_12_O_5_ | [M-H]- | Sinapic acid | Phenolic acids | Phenolic acids | 530-59-6 | 3 | 7.85E+04 | 7.73E+04 | 8.35E+04 | 2.69E+04 | 2.57E+04 | 2.31E+04 | 9.00E+00 | 9.00E+00 | 9.00E+00 | 9.00E+00 | 9.00E+00 | 9.00E+00 | 3.39E+04 | 2.05E+04 | 3.51E+04 | C00482 | ko00940,ko01100,ko01110 |
| Lmmp003517 | 2.25E+02 | 9.51E+01 | 2.24E+02 | C_13_H_20_O_3_ | [M+H]+ | Solatuberenol A | Others | Others | - | 2 | 3.80E+05 | 2.52E+05 | 3.19E+05 | 3.87E+05 | 2.72E+05 | 2.88E+05 | 4.14E+05 | 3.85E+05 | 3.63E+05 | 3.51E+05 | 4.51E+05 | 4.81E+05 | 4.30E+05 | 3.27E+05 | 4.37E+05 | -- | -- |
| Lmyp003951 | 2.27E+02 | 1.81E+02 | 2.26E+02 | C_11_H_14_O_5_ | [M+H]+ | 3-Hydroxy-1-(4-hydroxy-3,5-dimethoxyphenyl)propan-1-one | Phenolic acids | Phenolic acids | - | 3 | 3.47E+05 | 3.71E+05 | 3.02E+05 | 4.66E+05 | 4.95E+05 | 5.39E+05 | 2.40E+05 | 2.37E+05 | 3.18E+05 | 2.90E+05 | 3.18E+05 | 3.13E+05 | 3.46E+05 | 4.38E+05 | 3.85E+05 | -- | -- |
| Lmhp001430 | 2.27E+02 | 1.16E+02 | 2.26E+02 | C_10_H_14_N_2_O_4_ | [M+H]+ | Cyclo(Pro-Glu) | Amino acids and derivatives | Amino acids and derivatives | - | 2 | 2.81E+05 | 2.25E+05 | 3.59E+05 | 9.00E+00 | 9.00E+00 | 9.00E+00 | 6.86E+03 | 5.03E+03 | 7.95E+03 | 9.00E+00 | 9.00E+00 | 9.00E+00 | 1.01E+05 | 7.92E+04 | 9.11E+04 | -- | -- |
| pme1194 | 2.28E+02 | 1.12E+02 | 2.27E+02 | C_9_H_13_N_3_O_4_ | [M+H]+ | 2'-Deoxycytidine | Nucleotides and derivatives | Nucleotides and derivatives | 951-77-9 | 3 | 2.86E+05 | 3.07E+05 | 3.99E+05 | 5.45E+04 | 6.13E+04 | 4.91E+04 | 7.89E+04 | 7.02E+04 | 7.86E+04 | 4.30E+04 | 4.73E+04 | 5.69E+04 | 1.37E+05 | 1.51E+05 | 1.67E+05 | C00881 | ko00240,ko01100,ko02010 |
| Lmhp001461 | 2.29E+02 | 7.01E+01 | 2.28E+02 | C_11_H_20_N_2_O_3_ | [M+H]+ | L-Prolyl-L-Leucine | Amino acids and derivatives | Amino acids and derivatives | 52899-07-7 | 2 | 1.97E+06 | 2.04E+06 | 1.90E+06 | 2.86E+05 | 3.30E+05 | 3.46E+05 | 6.41E+05 | 7.11E+05 | 5.72E+05 | 3.42E+05 | 3.68E+05 | 2.70E+05 | 9.29E+05 | 8.50E+05 | 9.28E+05 | -- | -- |
| mws0119 | 2.27E+02 | 2.27E+02 | 2.28E+02 | C_14_H_28_O_2_ | [M-H]- | Myristic Acid | Lipids | Free fatty acids | 544-63-8 | 2 | 7.16E+06 | 7.71E+06 | 7.36E+06 | 9.42E+06 | 8.77E+06 | 8.61E+06 | 8.85E+06 | 7.57E+06 | 8.79E+06 | 8.17E+06 | 8.00E+06 | 7.49E+06 | 1.01E+07 | 1.02E+07 | 1.02E+07 | C06424 | ko00061,ko01100 |
| MWSmce160 | 2.31E+02 | 1.75E+02 | 2.30E+02 | C_14_H_14_O_3_ | [M+H]+ | Demethylsuberosin | Lignans and Coumarins | Coumarins | 21422-04-8 | 1 | 1.52E+06 | 1.40E+06 | 1.59E+06 | 2.44E+06 | 2.33E+06 | 2.39E+06 | 7.38E+05 | 7.35E+05 | 7.75E+05 | 9.51E+05 | 9.42E+05 | 9.51E+05 | 1.52E+06 | 1.59E+06 | 1.57E+06 | C18083 | -- |
| Zmbp007538 | 2.31E+02 | 1.75E+02 | 2.30E+02 | C_14_H_14_O_3_ | [M+H]+ | osthenol | Lignans and Coumarins | Coumarins | 484-14-0 | 1 | 5.06E+06 | 4.76E+06 | 4.26E+06 | 6.21E+06 | 6.08E+06 | 5.81E+06 | 3.34E+06 | 3.37E+06 | 3.30E+06 | 3.70E+06 | 3.93E+06 | 4.09E+06 | 4.97E+06 | 4.89E+06 | 5.01E+06 | -- | -- |
| Lmhp001670 | 2.31E+02 | 7.21E+01 | 2.30E+02 | C_11_H_22_N_2_O_3_ | [M+H]+ | L-Valyl-L-Leucine | Amino acids and derivatives | Amino acids and derivatives | 3989-97-7 | 2 | 1.97E+06 | 1.70E+06 | 1.84E+06 | 2.50E+05 | 3.68E+05 | 3.15E+05 | 2.44E+05 | 2.40E+05 | 2.41E+05 | 2.28E+05 | 3.24E+05 | 2.42E+05 | 6.99E+05 | 6.96E+05 | 7.15E+05 | -- | -- |
| pmp000287 | 2.33E+02 | 2.18E+02 | 2.32E+02 | C_12_H_8_O_5_ | [M+H]+ | 5-Methoxy-8-hydroxypsoralen | Lignans and Coumarins | Coumarins | 28437-68-5 | 2 | 3.48E+06 | 3.27E+06 | 2.64E+06 | 3.13E+06 | 2.66E+06 | 2.69E+06 | 3.42E+06 | 2.86E+06 | 3.49E+06 | 1.85E+06 | 1.75E+06 | 1.01E+06 | 2.82E+06 | 2.58E+06 | 2.50E+06 | -- | -- |
| Hmgp006596 | 2.35E+02 | 1.62E+02 | 2.34E+02 | C_11_H_10_N_2_S_2_ | [M+H]+ | Cyclobrassinin | Alkaloids | Plumerane | 105748-58-1 | 2 | 9.99E+05 | 1.02E+06 | 1.09E+06 | 1.15E+06 | 1.15E+06 | 1.11E+06 | 8.66E+05 | 9.35E+05 | 8.49E+05 | 7.94E+05 | 9.26E+05 | 8.71E+05 | 9.82E+05 | 1.10E+06 | 9.36E+05 | -- | -- |
| pmb0962 | 2.35E+02 | 1.18E+02 | 2.34E+02 | C_10_H_22_N_2_O_4_ | [M+H]+ | L-Lysine-Butanoic Acid | Amino acids and derivatives | Amino acids and derivatives | 80407-71-2 | 3 | 1.48E+05 | 1.30E+05 | 1.17E+05 | 1.11E+04 | 1.77E+04 | 1.30E+04 | 1.01E+05 | 1.04E+05 | 9.28E+04 | 5.62E+04 | 5.68E+04 | 3.23E+04 | 4.56E+04 | 5.99E+04 | 6.43E+04 | -- | -- |
| Lmcn005938 | 2.33E+02 | 1.89E+02 | 2.34E+02 | C_15_H_22_O_2_ | [M-H]- | Valerenic acid | Terpenoids | Sesquiterpenoids | - | 2 | 4.76E+03 | 3.00E+03 | 3.78E+03 | 2.96E+03 | 5.66E+03 | 3.82E+03 | 1.73E+03 | 2.13E+03 | 3.97E+03 | 3.25E+03 | 1.56E+03 | 3.10E+03 | 2.01E+03 | 2.73E+03 | 3.24E+03 | C09743 | -- |
| ML10174588 | 2.33E+02 | 2.33E+02 | 2.34E+02 | C_15_H_22_O_2_ | [M-H]- | Confertifoline | Terpenoids | Sesquiterpenoids | 1811-23-0 | 2 | 4.36E+06 | 4.39E+06 | 3.77E+06 | 4.49E+06 | 4.47E+06 | 4.32E+06 | 4.28E+06 | 4.11E+06 | 4.32E+06 | 4.20E+06 | 4.09E+06 | 4.60E+06 | 3.70E+06 | 4.02E+06 | 4.19E+06 | C09376 | -- |
| Lmhn010314 | 2.33E+02 | 2.17E+02 | 2.34E+02 | C_15_H_22_O_2_ | [M-H]- | Aspergillusene A | Terpenoids | Sesquiterpenoids | - | 2 | 2.43E+04 | 3.03E+04 | 1.85E+04 | 2.73E+04 | 2.05E+04 | 3.02E+04 | 2.67E+04 | 2.17E+04 | 2.45E+04 | 2.43E+04 | 2.95E+04 | 2.48E+04 | 2.00E+04 | 2.38E+04 | 2.37E+04 | -- | -- |
| MWSmce061 | 2.37E+02 | 1.76E+02 | 2.36E+02 | C_12_H_12_O_5_ | [M+H]+ | Dimethylfraxetin | Lignans and Coumarins | Coumarins | 6035-49-0 | 3 | 5.38E+04 | 5.41E+04 | 6.07E+04 | 6.60E+04 | 7.13E+04 | 6.92E+04 | 1.30E+04 | 1.34E+04 | 1.83E+04 | 1.53E+04 | 1.54E+04 | 1.44E+04 | 3.87E+04 | 3.63E+04 | 3.81E+04 | -- | -- |
| Hmlp006964 | 2.37E+02 | 1.76E+02 | 2.36E+02 | C_12_H_12_O_5_ | [M+H]+ | 5,6,7-Trimethoxycoumarin | Lignans and Coumarins | Coumarins | 55085-47-7 | 3 | 5.31E+04 | 5.00E+04 | 6.17E+04 | 6.57E+04 | 6.12E+04 | 5.61E+04 | 1.37E+04 | 1.12E+04 | 1.56E+04 | 1.19E+04 | 1.36E+04 | 1.63E+04 | 3.73E+04 | 4.07E+04 | 3.76E+04 | -- | -- |
| pmp000254 | 2.37E+02 | 1.49E+02 | 2.36E+02 | C_13_H_16_O_4_ | [M+H]+ | Z-6-Hydroxy-7-methoxydihydroligustilide | Others | Others | - | 3 | 1.02E+05 | 1.13E+05 | 9.05E+04 | 9.74E+04 | 1.15E+05 | 1.06E+05 | 1.51E+05 | 1.27E+05 | 1.21E+05 | 1.54E+05 | 1.34E+05 | 1.20E+05 | 1.11E+05 | 1.31E+05 | 1.02E+05 | -- | -- |
| mws4176 | 2.37E+02 | 1.20E+02 | 2.36E+02 | C_12_H_16_N_2_O_3_ | [M+H]+ | L-Alanyl-L-Phenylalanine | Amino acids and derivatives | Amino acids and derivatives | 3061-90-3 | 2 | 1.93E+05 | 1.91E+05 | 1.72E+05 | 3.51E+04 | 2.92E+04 | 3.47E+04 | 6.86E+04 | 7.96E+04 | 9.22E+04 | 3.72E+04 | 4.14E+04 | 3.55E+04 | 8.09E+04 | 7.07E+04 | 1.18E+05 | -- | -- |
| Zmdn011161 | 2.39E+02 | 2.23E+02 | 2.40E+02 | C_15_H_12_O_3_ | [M-H]- | Chrysophanol-9-anthrone | Quinones | Anthraquinone | 491-58-7 | 2 | 4.96E+04 | 7.87E+04 | 8.00E+04 | 1.17E+05 | 1.09E+05 | 1.03E+05 | 8.16E+04 | 1.24E+05 | 1.08E+05 | 5.37E+04 | 5.28E+04 | 1.90E+04 | 1.02E+07 | 1.01E+07 | 1.51E+07 | C10314 | -- |
| MWS0552 | 2.39E+02 | 2.23E+02 | 2.40E+02 | C_15_H_28_O_2_ | [M-H]- | Cis-10-Pentadecenoic Acid(C15: 1) | Lipids | Free fatty acids | 84743-29-3 | 1 | 3.90E+04 | 4.60E+04 | 5.27E+04 | 6.13E+04 | 7.72E+04 | 8.27E+04 | 5.76E+04 | 9.02E+04 | 8.95E+04 | 7.28E+04 | 4.67E+04 | 8.08E+04 | 7.69E+06 | 8.07E+06 | 1.17E+07 | -- | -- |
| pme2827 | 2.39E+02 | 2.23E+02 | 2.40E+02 | C_16_H_32_O | [M-H]- | Palmitaldehyde | Lipids | Free fatty acids | 629-80-1 | 3 | 5.21E+06 | 4.83E+06 | 5.27E+06 | 4.69E+06 | 4.97E+06 | 5.07E+06 | 4.85E+06 | 6.12E+06 | 5.66E+06 | 5.97E+06 | 5.97E+06 | 5.69E+06 | 1.31E+07 | 1.43E+07 | 1.77E+07 | C00517 | ko00071 |
| mws1383 | 2.43E+02 | 1.45E+02 | 2.42E+02 | C_12_H_10_N_4_O_2_ | [M+H]+ | Lumichrome | Alkaloids | Alkaloids | 1086-80-2 | 1 | 3.90E+04 | 3.86E+04 | 4.63E+04 | 1.70E+04 | 1.06E+04 | 1.18E+04 | 3.01E+04 | 3.64E+04 | 3.77E+04 | 1.91E+04 | 1.48E+04 | 1.82E+04 | 2.31E+04 | 2.27E+04 | 2.34E+04 | C01727 | ko00740 |
| pme0264 | 2.43E+02 | 1.27E+02 | 2.42E+02 | C_10_H_14_N_2_O_5_ | [M+H]+ | Thymidine | Nucleotides and derivatives | Nucleotides and derivatives | 50-89-5 | 1 | 1.23E+06 | 1.23E+06 | 1.43E+06 | 1.13E+05 | 1.03E+05 | 1.05E+05 | 2.29E+05 | 2.39E+05 | 2.30E+05 | 6.82E+04 | 8.92E+04 | 6.85E+04 | 5.56E+05 | 4.61E+05 | 5.82E+05 | C00214 | ko00240,ko01100 |
| mws0359 | 2.41E+02 | 2.41E+02 | 2.42E+02 | C_15_H_30_O_2_ | [M-H]- | Pentadecanoic Acid | Lipids | Free fatty acids | 1002-84-2 | 1 | 3.83E+05 | 3.90E+05 | 4.24E+05 | 4.44E+05 | 4.78E+05 | 4.80E+05 | 4.43E+05 | 4.16E+05 | 4.37E+05 | 4.24E+05 | 4.26E+05 | 4.03E+05 | 4.53E+05 | 4.24E+05 | 4.33E+05 | C16537 | -- |
| MWS2430 | 2.41E+02 | 2.41E+02 | 2.42E+02 | C_15_H_30_O_2_ | [M-H]- | 13-methylmyristic acid | Lipids | Free fatty acids | 2485-71-4 | 1 | 4.82E+05 | 4.48E+05 | 5.39E+05 | 1.00E+05 | 9.97E+04 | 9.68E+04 | 1.03E+05 | 9.28E+04 | 1.19E+05 | 1.31E+05 | 1.08E+05 | 9.64E+04 | 2.04E+05 | 2.23E+05 | 2.24E+05 | -- | -- |
| ML10180524 | 2.44E+02 | 1.12E+02 | 2.43E+02 | C_9_H_13_N_3_O_5_ | [M+H]+ | Cytarabine | Nucleotides and derivatives | Nucleotides and derivatives | 147-94-4 | 3 | 4.72E+06 | 4.78E+06 | 4.99E+06 | 1.07E+06 | 1.37E+06 | 1.01E+06 | 2.01E+06 | 2.51E+06 | 2.55E+06 | 1.04E+06 | 1.12E+06 | 1.07E+06 | 1.86E+06 | 2.10E+06 | 3.01E+06 | C02961 | -- |
| pme3732 | 2.44E+02 | 1.12E+02 | 2.43E+02 | C_9_H_13_N_3_O_5_ | [M+H]+ | Cytidine | Nucleotides and derivatives | Nucleotides and derivatives | 65-46-3 | 3 | 2.95E+07 | 2.98E+07 | 3.00E+07 | 5.94E+06 | 6.30E+06 | 6.40E+06 | 1.37E+07 | 1.38E+07 | 1.40E+07 | 5.65E+06 | 4.47E+06 | 4.99E+06 | 1.32E+07 | 1.14E+07 | 1.64E+07 | C00475 | ko00240,ko01100,ko02010 |
| mws0248 | 2.43E+02 | 1.10E+02 | 2.44E+02 | C_9_H_12_N_2_O_6_ | [M-H]- | Uridine | Nucleotides and derivatives | Nucleotides and derivatives | 58-96-8 | 1 | 1.73E+06 | 1.97E+06 | 1.81E+06 | 9.07E+05 | 9.27E+05 | 7.33E+05 | 1.12E+06 | 1.27E+06 | 1.15E+06 | 6.87E+05 | 7.40E+05 | 6.06E+05 | 1.14E+06 | 1.32E+06 | 1.31E+06 | C00299 | ko00240,ko01100,ko02010 |
| MWSmce295 | 2.45E+02 | 1.13E+02 | 2.44E+02 | C_9_H_12_N_2_O_6_ | [M+H]+ | 1-beta-D-Arabinofuranosyluracil | Nucleotides and derivatives | Nucleotides and derivatives | 3083-77-0 | 2 | 3.04E+06 | 3.31E+06 | 3.13E+06 | 9.10E+05 | 1.12E+06 | 9.59E+05 | 1.32E+06 | 1.40E+06 | 1.47E+06 | 8.91E+05 | 8.43E+05 | 6.71E+05 | 1.36E+06 | 1.44E+06 | 1.80E+06 | C16908 | -- |
| mws0976 | 2.43E+02 | 1.53E+02 | 2.44E+02 | C_9_H_12_N_2_O_6_ | [M-H]- | β-Pseudouridine | Nucleotides and derivatives | Nucleotides and derivatives | 1445-07-4 | 2 | 2.20E+05 | 2.02E+05 | 2.66E+05 | 1.20E+05 | 1.11E+05 | 8.59E+04 | 1.83E+05 | 1.79E+05 | 1.27E+05 | 7.64E+04 | 6.97E+04 | 7.58E+04 | 1.40E+05 | 1.26E+05 | 2.05E+05 | C02067 | ko00240,ko01100 |
| pme2266 | 2.45E+02 | 2.27E+02 | 2.44E+02 | C_10_H_16_N_2_O_3_S | [M+H]+ | Biotin | Others | Vitamin | 58-85-5 | 2 | 8.51E+04 | 9.80E+04 | 8.76E+04 | 9.75E+04 | 1.01E+05 | 1.07E+05 | 8.58E+04 | 8.35E+04 | 8.69E+04 | 9.03E+04 | 6.70E+04 | 1.06E+05 | 1.04E+05 | 8.62E+04 | 7.98E+04 | C00120 | ko00780,ko01100,ko01240,ko02010 |
| MWSmce385 | 2.45E+02 | 6.91E+01 | 2.44E+02 | C_15_H_16_O_3_ | [M+H]+ | Suberosin | Lignans and Coumarins | Coumarins | 581-31-7 | 1 | 1.25E+07 | 1.25E+07 | 1.30E+07 | 1.92E+07 | 1.66E+07 | 1.86E+07 | 1.05E+07 | 1.12E+07 | 1.11E+07 | 9.39E+06 | 9.59E+06 | 1.02E+07 | 1.31E+07 | 1.34E+07 | 1.45E+07 | -- | -- |
| pmf0527 | 2.45E+02 | 1.59E+02 | 2.44E+02 | C_15_H_16_O_3_ | [M+H]+ | Osthole | Lignans and Coumarins | Coumarins | 484-12-8 | 1 | 6.46E+05 | 6.63E+05 | 6.18E+05 | 7.43E+05 | 7.22E+05 | 7.07E+05 | 5.24E+05 | 5.76E+05 | 5.42E+05 | 4.50E+05 | 4.78E+05 | 4.43E+05 | 5.96E+05 | 6.19E+05 | 6.23E+05 | C09280 | -- |
| Lmhp003064 | 2.45E+02 | 1.20E+02 | 2.44E+02 | C_14_H_16_N_2_O_2_ | [M+H]+ | Cyclo(Pro-Phe) | Amino acids and derivatives | Amino acids and derivatives | 3705-26-8 | 3 | 5.03E+04 | 6.05E+04 | 4.16E+04 | 9.00E+00 | 9.00E+00 | 9.00E+00 | 9.00E+00 | 9.00E+00 | 9.00E+00 | 9.00E+00 | 9.00E+00 | 9.00E+00 | 1.83E+04 | 1.64E+04 | 1.90E+04 | C11847 | -- |
| MWS5231 | 2.43E+02 | 1.81E+02 | 2.44E+02 | C_13_H_24_O_4_ | [M-H]- | Tridecanedioic acid | Lipids | Free fatty acids | 505-52-2 | 1 | 7.73E+04 | 7.58E+04 | 7.35E+04 | 8.53E+04 | 9.36E+04 | 9.31E+04 | 9.50E+04 | 8.18E+04 | 8.17E+04 | 7.79E+04 | 7.68E+04 | 8.75E+04 | 9.27E+04 | 9.10E+04 | 8.70E+04 | -- | -- |
| Lmhp002031 | 2.45E+02 | 8.61E+01 | 2.44E+02 | C_12_H_24_N_2_O_3_ | [M+H]+ | L-Leucyl-L-Leucine | Amino acids and derivatives | Amino acids and derivatives | 3303-31-9 | 2 | 3.30E+05 | 3.26E+05 | 4.26E+05 | 8.07E+04 | 7.32E+04 | 7.24E+04 | 3.88E+04 | 3.92E+04 | 4.23E+04 | 7.62E+04 | 8.65E+04 | 7.55E+04 | 1.45E+05 | 1.62E+05 | 1.61E+05 | -- | -- |
| MWSslk220 | 2.47E+02 | 2.31E+02 | 2.46E+02 | C_13_H_10_O_5_ | [M+H]+ | Pimpinellin | Lignans and Coumarins | Coumarins | 131-12-4 | 3 | 2.47E+04 | 2.64E+04 | 2.11E+04 | 2.67E+04 | 3.21E+04 | 3.35E+04 | 3.99E+04 | 3.97E+04 | 4.27E+04 | 4.33E+04 | 3.96E+04 | 4.90E+04 | 3.28E+04 | 3.55E+04 | 3.47E+04 | C09285 | -- |
| pmp000288 | 2.47E+02 | 1.61E+02 | 2.46E+02 | C_13_H_10_O_5_ | [M+H]+ | Isopimpinellin | Lignans and Coumarins | Coumarins | 482-27-9 | 1 | 2.91E+07 | 2.70E+07 | 3.17E+07 | 3.46E+07 | 3.33E+07 | 3.35E+07 | 2.70E+07 | 2.76E+07 | 2.79E+07 | 3.14E+07 | 3.33E+07 | 3.50E+07 | 3.14E+07 | 3.16E+07 | 3.22E+07 | C02162 | ko01110 |
| MWSmce265 | 2.47E+02 | 2.29E+02 | 2.46E+02 | C_14_H_14_O_4_ | [M+H]+ | Decursinol | Others | Saccharides and Alcohols | 23458-02-8 | 2 | 2.63E+06 | 2.74E+06 | 2.48E+06 | 2.83E+06 | 3.45E+06 | 3.05E+06 | 8.64E+05 | 8.26E+05 | 9.22E+05 | 9.59E+05 | 9.72E+05 | 9.52E+05 | 1.96E+06 | 1.86E+06 | 2.09E+06 | C09259 | -- |
| mws0124 | 2.47E+02 | 1.30E+02 | 2.46E+02 | C_13_H_14_N_2_O_3_ | [M+H]+ | N-(3-Indolylacetyl)-L-alanine | Amino acids and derivatives | Amino acids and derivatives | 57105-39-2 | 3 | 4.00E+04 | 1.52E+04 | 1.68E+04 | 3.36E+04 | 2.93E+04 | 3.66E+04 | 2.31E+04 | 2.48E+04 | 3.39E+04 | 2.57E+04 | 1.58E+04 | 2.07E+04 | 1.84E+04 | 2.85E+04 | 3.01E+04 | -- | -- |
| pmb2591 | 2.45E+02 | 2.03E+02 | 2.46E+02 | C_13_H_14_N_2_O_3_ | [M-H]- | N-Acetyl-L-Tryptophan | Amino acids and derivatives | Amino acids and derivatives | 1218-34-4 | 2 | 5.08E+05 | 4.15E+05 | 4.85E+05 | 6.67E+05 | 8.56E+05 | 6.84E+05 | 5.46E+05 | 5.70E+05 | 6.80E+05 | 6.06E+05 | 7.72E+05 | 6.45E+05 | 7.35E+05 | 5.88E+05 | 7.13E+05 | -- | -- |
| Lmrj002087 | 2.47E+02 | 8.61E+01 | 2.46E+02 | C_10_H_18_N_2_O_5_ | [M+H]+ | L-Isoleucyl-L-Aspartate | Amino acids and derivatives | Amino acids and derivatives | - | 2 | 7.18E+05 | 6.97E+05 | 7.65E+05 | 1.72E+05 | 2.38E+05 | 2.61E+05 | 3.25E+05 | 3.64E+05 | 3.22E+05 | 2.44E+05 | 2.57E+05 | 2.24E+05 | 4.14E+05 | 3.76E+05 | 4.51E+05 | -- | -- |
| Zmdp001647 | 2.47E+02 | 1.84E+02 | 2.46E+02 | C_10_H_18_N_2_O_5_ | [M+H]+ | γ-Glutamyl-L-valine | Amino acids and derivatives | Amino acids and derivatives | - | 1 | 1.16E+05 | 1.17E+05 | 1.29E+05 | 7.09E+04 | 6.94E+04 | 6.15E+04 | 1.17E+05 | 1.35E+05 | 1.37E+05 | 9.66E+04 | 8.68E+04 | 1.00E+05 | 1.02E+05 | 9.87E+04 | 1.10E+05 | -- | -- |
| Lmbp010056 | 2.51E+02 | 9.11E+01 | 2.50E+02 | C_17_H_14_O_2_ | [M+H]+ | 2-(2-Phenylethyl)chromone | Others | Others | 61828-53-3 | 1 | 7.30E+03 | 1.25E+04 | 1.54E+04 | 1.31E+04 | 1.50E+04 | 1.23E+04 | 8.38E+03 | 1.35E+04 | 1.36E+04 | 7.43E+03 | 9.34E+03 | 1.07E+04 | 1.13E+04 | 1.10E+04 | 1.39E+04 | C09007 | -- |
| Lmcp006876 | 2.51E+02 | 9.11E+01 | 2.50E+02 | C_17_H_14_O_2_ | [M+H]+ | 8-(Benzyloxy)-1-Naphthol | Others | Others | - | 2 | 6.94E+03 | 1.08E+04 | 1.23E+04 | 1.02E+04 | 1.18E+04 | 1.44E+04 | 8.93E+03 | 1.18E+04 | 1.13E+04 | 6.96E+03 | 5.90E+03 | 6.99E+03 | 1.06E+04 | 1.14E+04 | 1.47E+04 | -- | -- |
| Lmjn006711 | 2.49E+02 | 2.05E+02 | 2.50E+02 | C_15_H_22_O_3_ | [M-H]- | 4,5-Epoxyartemisinic Acid | Terpenoids | Sesquiterpenoids | 92466-31-4 | 2 | 8.85E+04 | 7.72E+04 | 7.90E+04 | 9.92E+04 | 8.69E+04 | 8.44E+04 | 8.32E+04 | 7.19E+04 | 7.54E+04 | 7.94E+04 | 7.47E+04 | 8.01E+04 | 7.75E+04 | 8.85E+04 | 8.86E+04 | -- | -- |
| Hmpn004989 | 2.49E+02 | 2.05E+02 | 2.50E+02 | C_15_H_22_O_3_ | [M-H]- | 5-Hydroxylbisabolon-9-one | Terpenoids | Sesquiterpenoids | - | 2 | 8.81E+03 | 8.06E+03 | 1.40E+04 | 7.60E+03 | 9.33E+03 | 9.83E+03 | 1.33E+04 | 1.04E+04 | 1.03E+04 | 1.21E+04 | 1.41E+04 | 1.11E+04 | 1.22E+04 | 1.13E+04 | 1.38E+04 | -- | -- |
| pme3961 | 2.52E+02 | 1.36E+02 | 2.51E+02 | C_10_H_13_N_5_O_3_ | [M+H]+ | 2'-Deoxyadenosine | Nucleotides and derivatives | Nucleotides and derivatives | 958-09-8 | 2 | 9.62E+06 | 9.01E+06 | 1.11E+07 | 3.28E+05 | 3.31E+05 | 3.30E+05 | 1.07E+06 | 1.04E+06 | 1.03E+06 | 3.74E+05 | 3.76E+05 | 3.51E+05 | 3.84E+06 | 3.66E+06 | 4.13E+06 | C00559 | ko00230,ko01100,ko02010 |
| mws1715 | 2.52E+02 | 6.90E+01 | 2.51E+02 | C_10_H_13_N_5_O_3_ | [M+H]+ | Cordycepin (3'-Deoxyadenosine) | Nucleotides and derivatives | Nucleotides and derivatives | 73-03-0 | 3 | 1.10E+05 | 9.71E+04 | 1.33E+05 | 9.00E+00 | 9.00E+00 | 9.00E+00 | 1.89E+04 | 1.94E+04 | 1.79E+04 | 9.00E+00 | 9.00E+00 | 9.00E+00 | 4.55E+04 | 5.48E+04 | 4.81E+04 | C08431 | -- |
| MWSslk183 | 2.55E+02 | 1.81E+02 | 2.54E+02 | C_15_H_10_O_4_ | [M+H]+ | 7,8-Dihydroxy-4-phenylcoumarin | Lignans and Coumarins | Coumarins | 842-01-3 | 3 | 4.16E+04 | 4.36E+04 | 4.84E+04 | 8.78E+03 | 1.16E+04 | 9.66E+03 | 5.28E+04 | 5.86E+04 | 6.17E+04 | 3.45E+04 | 4.13E+04 | 3.64E+04 | 4.23E+04 | 4.01E+04 | 4.10E+04 | -- | -- |
| mws0040 | 2.55E+02 | 1.53E+02 | 2.54E+02 | C_15_H_10_O_4_ | [M+H]+ | Chrysin | Flavonoids | Flavones | 480-40-0 | 3 | 6.46E+03 | 7.58E+03 | 1.08E+04 | 1.70E+05 | 1.60E+05 | 1.76E+05 | 4.52E+04 | 3.23E+04 | 4.01E+04 | 9.50E+04 | 9.93E+04 | 9.96E+04 | 8.57E+04 | 7.11E+04 | 8.91E+04 | C10028 | ko00941 |
| Lmcn009122 | 2.53E+02 | 2.35E+02 | 2.54E+02 | C_16_H_30_O_2_ | [M-H]- | (7Z)-Hexadecenoic acid | Lipids | Free fatty acids | 2416-19-5 | 1 | 1.88E+04 | 1.88E+04 | 1.64E+04 | 2.90E+04 | 2.71E+04 | 3.10E+04 | 1.39E+04 | 1.74E+04 | 2.28E+04 | 3.44E+04 | 2.35E+04 | 1.87E+04 | 2.11E+04 | 2.17E+04 | 2.62E+04 | -- | -- |
| mws0361 | 2.53E+02 | 2.35E+02 | 2.54E+02 | C_16_H_30_O_2_ | [M-H]- | Palmitoleic Acid | Lipids | Free fatty acids | 373-49-9 | 1 | 1.24E+04 | 1.98E+04 | 1.22E+04 | 1.67E+04 | 1.78E+04 | 1.70E+04 | 1.18E+04 | 8.01E+03 | 1.81E+04 | 2.96E+04 | 1.59E+04 | 1.18E+04 | 1.68E+04 | 1.95E+04 | 1.80E+04 | C08362 | ko00061 |
| pma3101 | 2.56E+02 | 1.24E+02 | 2.56E+02 | C_11_H_14_NO_6_+ | [M]+ | Nicotinate D-ribonucleoside | Others | Vitamin | 17720-18-2 | 3 | 1.51E+06 | 1.48E+06 | 1.46E+06 | 1.39E+06 | 1.29E+06 | 1.42E+06 | 6.53E+05 | 7.05E+05 | 7.47E+05 | 1.09E+06 | 1.11E+06 | 1.07E+06 | 1.14E+06 | 1.19E+06 | 1.24E+06 | C05841 | ko00760,ko01100 |
| mws1488 | 2.55E+02 | 2.37E+02 | 2.56E+02 | C_16_H_32_O_2_ | [M-H]- | Palmitic acid | Lipids | Free fatty acids | 57-10-3 | 2 | 2.91E+04 | 3.46E+04 | 3.88E+04 | 4.63E+04 | 3.97E+04 | 3.94E+04 | 4.35E+04 | 4.10E+04 | 4.37E+04 | 4.23E+04 | 4.16E+04 | 3.77E+04 | 4.06E+04 | 4.06E+04 | 3.36E+04 | C00249 | ko00061,ko00062,ko00071,ko00073,ko01040,ko01100,ko01212 |
| mws0120 | 2.58E+02 | 1.04E+02 | 2.57E+02 | C_8_H_20_NO_6_P | [M+H]+ | Choline Alfoscerate | Lipids | PC | 28319-77-9 | 3 | 8.61E+04 | 9.16E+04 | 6.11E+04 | 7.72E+05 | 7.85E+05 | 6.98E+05 | 1.09E+06 | 1.15E+06 | 1.09E+06 | 4.79E+05 | 4.74E+05 | 5.16E+05 | 6.28E+05 | 6.42E+05 | 6.00E+05 | C00670 | ko00564,ko00565 |
| pme1187 | 2.57E+02 | 1.24E+02 | 2.58E+02 | C_10_H_14_N_2_O_6_ | [M-H]- | 5-Methyluridine | Nucleotides and derivatives | Nucleotides and derivatives | 1463-10-1 | 3 | 1.34E+05 | 1.35E+05 | 1.25E+05 | 9.36E+03 | 1.20E+04 | 1.41E+04 | 2.72E+04 | 3.10E+04 | 2.07E+04 | 1.50E+04 | 1.27E+04 | 1.08E+04 | 4.51E+04 | 5.36E+04 | 5.34E+04 | -- | -- |
| Zmyn000110 | 2.58E+02 | 7.90E+01 | 2.59E+02 | C_6_H_14_NO_8_P | [M-H]- | D-Glucosamine 1-phosphate | Others | Saccharides and Alcohols | 2152-75-2 | 3 | 5.70E+04 | 5.61E+04 | 6.88E+04 | 1.10E+05 | 1.15E+05 | 1.29E+05 | 1.01E+05 | 1.06E+05 | 1.01E+05 | 1.28E+05 | 1.35E+05 | 1.36E+05 | 7.62E+04 | 9.92E+04 | 9.88E+04 | C06156 | ko00520,ko01100 |
| mws1090 | 2.59E+02 | 9.70E+01 | 2.60E+02 | C_6_H_13_O_9_P | [M-H]- | Glucose-1-phosphate | Others | Saccharides and Alcohols | 59-56-3 | 3 | 9.49E+05 | 9.36E+05 | 1.01E+06 | 4.42E+06 | 4.97E+06 | 5.35E+06 | 2.55E+06 | 2.48E+06 | 2.34E+06 | 4.82E+06 | 4.28E+06 | 4.38E+06 | 2.79E+06 | 3.10E+06 | 3.34E+06 | C00103 | ko00010,ko00040,ko00052,ko00500,ko00520,ko00523,ko00561,ko01100,ko01110,ko01240 |
| pme3313 | 2.59E+02 | 9.70E+01 | 2.60E+02 | C_6_H_13_O_9_P | [M-H]- | D-Fructose 6-phosphate | Others | Saccharides and Alcohols | 643-13-0 | 3 | 2.96E+05 | 3.06E+05 | 3.40E+05 | 2.40E+06 | 2.12E+06 | 2.55E+06 | 1.04E+06 | 1.07E+06 | 1.18E+06 | 2.21E+06 | 2.07E+06 | 1.82E+06 | 1.31E+06 | 1.27E+06 | 1.41E+06 | C00085 | ko00052,ko00500,ko00710,ko01100,ko01110,ko01200 |
| mws0866 | 2.59E+02 | 9.70E+01 | 2.60E+02 | C_6_H_13_O_9_P | [M-H]- | D-Glucose 6-phosphate | Others | Saccharides and Alcohols | 56-73-5 | 3 | 9.23E+05 | 9.49E+05 | 1.03E+06 | 4.91E+06 | 5.08E+06 | 5.26E+06 | 2.46E+06 | 2.45E+06 | 2.63E+06 | 4.87E+06 | 4.78E+06 | 4.88E+06 | 2.94E+06 | 3.34E+06 | 3.59E+06 | C00092 | ko00500,ko00524,ko00562,ko00998,ko01100,ko01110 |
| Cmpp007319 | 2.61E+02 | 1.89E+02 | 2.60E+02 | C_15_H_16_O_4_ | [M+H]+ | 7-Methoxy-5-Prenyloxycoumarin | Lignans and Coumarins | Coumarins | 35590-41-1 | 2 | 1.51E+06 | 1.34E+06 | 1.21E+06 | 1.42E+06 | 1.32E+06 | 1.28E+06 | 9.12E+05 | 9.27E+05 | 9.05E+05 | 5.75E+05 | 5.98E+05 | 6.17E+05 | 1.07E+06 | 1.05E+06 | 1.13E+06 | -- | -- |
| Zmnn010135 | 2.59E+02 | 2.44E+02 | 2.60E+02 | C_15_H_16_O_4_ | [M-H]- | Sibiricol | Lignans and Coumarins | Coumarins | 26481-12-9 | 2 | 3.00E+05 | 3.11E+05 | 2.91E+05 | 5.38E+05 | 4.33E+05 | 4.53E+05 | 2.48E+05 | 2.41E+05 | 2.60E+05 | 3.32E+05 | 3.23E+05 | 3.41E+05 | 3.30E+05 | 3.45E+05 | 4.03E+05 | -- | -- |
| Cmpp005475 | 2.61E+02 | 1.89E+02 | 2.60E+02 | C_15_H_16_O_4_ | [M+H]+ | Meranzin | Lignans and Coumarins | Coumarins | 23971-42-8 | 3 | 4.32E+04 | 4.57E+04 | 4.89E+04 | 3.94E+04 | 3.68E+04 | 2.99E+04 | 2.84E+04 | 2.86E+04 | 2.22E+04 | 2.41E+04 | 2.18E+04 | 2.31E+04 | 3.39E+04 | 4.48E+04 | 3.45E+04 | -- | -- |
| Zmdp002216 | 2.61E+02 | 8.61E+01 | 2.60E+02 | C_11_H_20_N_2_O_5_ | [M+H]+ | L-γ-Glutamyl-L-leucine | Amino acids and derivatives | Amino acids and derivatives | 2566-39-4 | 1 | 2.72E+05 | 2.28E+05 | 2.67E+05 | 1.21E+05 | 1.11E+05 | 1.07E+05 | 2.63E+05 | 2.46E+05 | 2.37E+05 | 1.08E+05 | 1.68E+05 | 1.71E+05 | 1.99E+05 | 1.69E+05 | 2.30E+05 | -- | -- |
| Zmpn000095 | 2.61E+02 | 7.90E+01 | 2.62E+02 | C_6_H_15_O_9_P | [M-H]- | Sorbitol-6-phosphate | Others | Saccharides and Alcohols | 20479-58-7 | 3 | 1.70E+04 | 2.83E+04 | 3.10E+04 | 4.03E+04 | 3.95E+04 | 4.30E+04 | 3.55E+04 | 3.31E+04 | 3.10E+04 | 3.90E+04 | 4.12E+04 | 4.50E+04 | 3.37E+04 | 3.64E+04 | 3.96E+04 | C01096 | ko00051,ko01100 |
| Hmcp005031 | 2.63E+02 | 1.91E+02 | 2.62E+02 | C_14_H_14_O_5_ | [M+H]+ | Rutaretin | Lignans and Coumarins | Coumarins | 13895-92-6 | 2 | 4.32E+06 | 5.01E+06 | 4.07E+06 | 1.25E+06 | 1.37E+06 | 1.34E+06 | 8.38E+05 | 7.91E+05 | 8.25E+05 | 6.81E+05 | 7.43E+05 | 6.97E+05 | 1.85E+06 | 2.03E+06 | 1.99E+06 | -- | -- |
| Lmhp001732 | 2.63E+02 | 7.01E+01 | 2.62E+02 | C_14_H_18_N_2_O_3_ | [M+H]+ | L-Prolyl-L-Phenylalanine | Amino acids and derivatives | Amino acids and derivatives | 13589-02-1 | 2 | 8.32E+04 | 6.80E+04 | 8.24E+04 | 3.40E+04 | 3.62E+04 | 4.24E+04 | 3.35E+04 | 4.52E+04 | 2.71E+04 | 3.70E+04 | 3.66E+04 | 3.77E+04 | 6.48E+04 | 6.86E+04 | 6.80E+04 | -- | -- |
| Hmsp001724 | 2.63E+02 | 2.45E+02 | 2.62E+02 | C_15_H_22_N_2_O_2_ | [M+H]+ | 5α-Hydroxysophocarpine | Alkaloids | Quinorisidine alkaloids | - | 2 | 1.69E+04 | 1.99E+04 | 1.27E+04 | 9.00E+00 | 9.00E+00 | 9.00E+00 | 9.00E+00 | 9.00E+00 | 9.00E+00 | 9.00E+00 | 9.00E+00 | 9.00E+00 | 9.54E+03 | 1.02E+04 | 5.65E+03 | -- | -- |
| mws0715 | 2.63E+02 | 1.45E+02 | 2.64E+02 | C_13_H_16_N_2_O_4_ | [M-H]- | Phenylacetyl-L-glutamine | Amino acids and derivatives | Amino acids and derivatives | 28047-15-6 | 3 | 1.67E+04 | 1.44E+04 | 1.60E+04 | 9.00E+00 | 9.00E+00 | 9.00E+00 | 9.00E+00 | 9.00E+00 | 9.00E+00 | 9.00E+00 | 9.00E+00 | 9.00E+00 | 5.22E+03 | 1.04E+04 | 6.68E+03 | C04148 | ko00360 |
| Lmtn004049 | 2.63E+02 | 2.04E+02 | 2.64E+02 | C_15_H_20_O_4_ | [M-H]- | Abscisic acid | Organic acids | Organic acids | 21293-29-8 | 1 | 6.27E+04 | 6.07E+04 | 6.16E+04 | 7.01E+04 | 9.13E+04 | 8.50E+04 | 7.34E+04 | 6.59E+04 | 7.97E+04 | 9.13E+04 | 1.05E+05 | 9.44E+04 | 7.01E+04 | 7.84E+04 | 8.56E+04 | C06082 | ko00906,ko01100,ko01110,ko04075 |
| Lmlp003161 | 2.65E+02 | 1.77E+02 | 2.64E+02 | C_14_H_20_N_2_O_3_ | [M+H]+ | N-Feruloylputrescine | Alkaloids | Phenolamine | 501-13-3 | 3 | 9.00E+00 | 9.00E+00 | 9.00E+00 | 2.06E+04 | 1.53E+04 | 1.77E+04 | 9.00E+00 | 9.00E+00 | 9.00E+00 | 1.80E+04 | 1.74E+04 | 3.00E+04 | 1.55E+04 | 1.34E+04 | 1.69E+04 | C10497 | ko00330,ko01100 |
| Lmhp002001 | 2.65E+02 | 7.21E+01 | 2.64E+02 | C_14_H_20_N_2_O_3_ | [M+H]+ | L-Valyl-L-Phenylalanine | Amino acids and derivatives | Amino acids and derivatives | 3918-92-1 | 2 | 6.92E+05 | 6.86E+05 | 6.89E+05 | 1.10E+05 | 1.46E+05 | 1.40E+05 | 1.25E+05 | 1.40E+05 | 1.31E+05 | 1.30E+05 | 1.16E+05 | 1.06E+05 | 3.08E+05 | 3.18E+05 | 3.07E+05 | -- | -- |
| Zmhn005256 | 2.65E+02 | 2.24E+02 | 2.66E+02 | C_18_H_18_O_2_ | [M-H]- | (E)-5-Allyl-3'-(prop-1-enyl)biphenyl-2,4'-diol | Lignans and Coumarins | Lignans | - | 1 | 2.95E+05 | 2.35E+05 | 2.72E+05 | 7.24E+03 | 1.55E+04 | 9.18E+03 | 1.37E+04 | 3.03E+04 | 2.57E+04 | 3.60E+04 | 2.63E+04 | 3.42E+04 | 8.53E+04 | 9.38E+04 | 9.49E+04 | -- | -- |
| MWSmce202 | 2.65E+02 | 2.24E+02 | 2.66E+02 | C_18_H_18_O_2_ | [M-H]- | Honokiol | Lignans and Coumarins | Lignans | 35354-74-6 | 2 | 2.92E+05 | 2.40E+05 | 2.87E+05 | 7.80E+03 | 1.59E+04 | 1.07E+04 | 1.29E+04 | 3.12E+04 | 2.60E+04 | 3.64E+04 | 2.72E+04 | 3.70E+04 | 8.75E+04 | 9.95E+04 | 1.06E+05 | C10630 | -- |
| pme1184 | 2.68E+02 | 1.52E+02 | 2.67E+02 | C_10_H_13_N_5_O_4_ | [M+H]+ | 2'-Deoxyguanosine | Nucleotides and derivatives | Nucleotides and derivatives | 961-07-9 | 2 | 3.01E+06 | 2.95E+06 | 3.32E+06 | 1.84E+05 | 1.85E+05 | 1.88E+05 | 4.08E+05 | 3.99E+05 | 4.08E+05 | 1.32E+05 | 1.45E+05 | 1.26E+05 | 1.07E+06 | 1.07E+06 | 1.20E+06 | C00330 | ko00230,ko01100,ko02010 |
| pme0230 | 2.68E+02 | 1.36E+02 | 2.67E+02 | C_10_H_13_N_5_O_4_ | [M+H]+ | Adenosine | Nucleotides and derivatives | Nucleotides and derivatives | 58-61-7 | 1 | 1.57E+07 | 1.53E+07 | 1.47E+07 | 4.12E+06 | 4.40E+06 | 4.16E+06 | 7.52E+06 | 7.00E+06 | 7.21E+06 | 3.69E+06 | 3.82E+06 | 3.42E+06 | 8.13E+06 | 7.52E+06 | 8.27E+06 | C00212 | ko00230,ko01100,ko02010 |
| Zmjp000966 | 2.68E+02 | 1.36E+02 | 2.67E+02 | C_10_H_13_N_5_O_4_ | [M+H]+ | Vidarabine | Others | Others | 5536-17-4 | 1 | 1.52E+07 | 1.44E+07 | 1.42E+07 | 4.05E+06 | 4.45E+06 | 3.99E+06 | 7.14E+06 | 6.71E+06 | 6.98E+06 | 3.59E+06 | 3.95E+06 | 3.33E+06 | 8.04E+06 | 7.47E+06 | 8.36E+06 | -- | -- |
| MWSmce139 | 2.69E+02 | 2.41E+02 | 2.68E+02 | C_15_H_8_O_5_ | [M+H]+ | Coumestrol | Lignans and Coumarins | Coumarins | 479-13-0 | 3 | 2.22E+04 | 1.92E+04 | 1.89E+04 | 2.06E+04 | 1.85E+04 | 2.27E+04 | 8.24E+03 | 1.23E+04 | 1.22E+04 | 5.54E+03 | 8.53E+03 | 8.60E+03 | 1.25E+04 | 1.54E+04 | 1.71E+04 | C10205 | ko00943,ko01110 |
| MWSHY0140 | 2.69E+02 | 2.26E+02 | 2.68E+02 | C_16_H_12_O_4_ | [M+H]+ | 3-Hydroxy-3'-methoxyflavone | Flavonoids | Flavones | 76666-32-5 | 2 | 1.97E+05 | 1.99E+05 | 1.94E+05 | 9.00E+00 | 9.00E+00 | 9.00E+00 | 6.50E+04 | 6.97E+04 | 7.06E+04 | 9.00E+00 | 9.00E+00 | 9.00E+00 | 7.32E+04 | 7.73E+04 | 6.97E+04 | -- | -- |
| mws1060 | 2.67E+02 | 1.35E+02 | 2.68E+02 | C_10_H_12_N_4_O_5_ | [M-H]- | 9-(Arabinosyl)hypoxanthine | Nucleotides and derivatives | Nucleotides and derivatives | 7013-16-3 | 1 | 8.86E+05 | 8.46E+05 | 9.02E+05 | 2.44E+05 | 2.49E+05 | 2.15E+05 | 2.71E+05 | 2.56E+05 | 2.67E+05 | 1.02E+05 | 1.03E+05 | 1.05E+05 | 3.70E+05 | 4.51E+05 | 4.74E+05 | -- | -- |
| pmp000086 | 2.69E+02 | 1.77E+02 | 2.68E+02 | C_13_H_16_O_6_ | [M+H]+ | 1-Feruloyl-sn-glycerol* | Phenolic acids | Phenolic acids | - | 3 | 8.70E+04 | 6.51E+04 | 8.71E+04 | 1.16E+04 | 1.26E+04 | 1.27E+04 | 4.05E+04 | 2.95E+04 | 2.70E+04 | 8.16E+03 | 1.54E+04 | 7.22E+03 | 3.84E+04 | 1.45E+04 | 2.92E+04 | -- | -- |
| pmp000087 | 2.69E+02 | 1.77E+02 | 2.68E+02 | C_13_H_16_O_6_ | [M+H]+ | 2-Feruloyl-sn-glycerol* | Phenolic acids | Phenolic acids | - | 3 | 1.05E+05 | 7.80E+04 | 9.19E+04 | 2.89E+04 | 6.57E+03 | 2.49E+04 | 4.22E+04 | 3.37E+04 | 2.93E+04 | 1.58E+04 | 1.02E+04 | 1.10E+04 | 3.41E+04 | 1.99E+04 | 3.17E+04 | -- | -- |
| mws0383 | 2.67E+02 | 2.67E+02 | 2.68E+02 | C_17_H_32_O_2_ | [M-H]- | 10-Heptadecenoic Acid | Lipids | Free fatty acids | 29743-97-3 | 2 | 1.77E+06 | 1.98E+06 | 1.72E+06 | 3.17E+06 | 2.73E+06 | 3.10E+06 | 3.10E+06 | 2.93E+06 | 3.06E+06 | 3.32E+06 | 3.07E+06 | 2.77E+06 | 2.92E+06 | 3.16E+06 | 2.91E+06 | -- | -- |
| Hmcp009386 | 2.71E+02 | 1.47E+02 | 2.70E+02 | C_16_H_14_O_4_ | [M+H]+ | Alloimperatorin | Lignans and Coumarins | Coumarins | 642-05-7 | 1 | 2.21E+06 | 2.10E+06 | 2.05E+06 | 2.24E+06 | 2.24E+06 | 2.22E+06 | 1.70E+06 | 1.86E+06 | 1.84E+06 | 1.66E+06 | 1.87E+06 | 1.86E+06 | 2.05E+06 | 1.98E+06 | 2.26E+06 | C09053 | -- |
| Hmmp008597 | 2.71E+02 | 1.47E+02 | 2.70E+02 | C_16_H_14_O_4_ | [M+H]+ | 4,4'-Dihydroxy-2'-methoxychalcone (3-Deoxysappanchalcone) | Flavonoids | Chalcones | 112408-67-0 | 1 | 2.31E+06 | 2.38E+06 | 2.21E+06 | 2.43E+06 | 2.35E+06 | 2.36E+06 | 1.69E+06 | 1.81E+06 | 1.97E+06 | 1.85E+06 | 1.85E+06 | 2.05E+06 | 2.18E+06 | 2.05E+06 | 2.32E+06 | C15531 | -- |
| pmf0526 | 2.71E+02 | 1.47E+02 | 2.70E+02 | C_16_H_14_O_4_ | [M+H]+ | Isoimperatorin | Lignans and Coumarins | Coumarins | 482-45-1 | 1 | 2.88E+06 | 2.77E+06 | 2.76E+06 | 3.00E+06 | 2.95E+06 | 2.85E+06 | 2.14E+06 | 2.24E+06 | 2.21E+06 | 2.09E+06 | 2.14E+06 | 2.22E+06 | 2.57E+06 | 2.60E+06 | 2.52E+06 | C16976 | -- |
| Zmbp009086 | 2.71E+02 | 2.03E+02 | 2.70E+02 | C_16_H_14_O_4_ | [M+H]+ | Imperatorin | Lignans and Coumarins | Coumarins | 482-44-0 | 1 | 2.29E+07 | 2.21E+07 | 2.18E+07 | 2.42E+07 | 2.39E+07 | 2.29E+07 | 1.92E+07 | 1.91E+07 | 1.95E+07 | 1.91E+07 | 1.99E+07 | 2.01E+07 | 2.09E+07 | 2.08E+07 | 2.36E+07 | C09269 | -- |
| MWS20159 | 2.71E+02 | 9.11E+01 | 2.70E+02 | C_13_H_18_O_6_ | [M+H]+ | Phenyl-β-D-glucopyranoside | Others | Others | - | 1 | 4.43E+04 | 3.92E+04 | 4.15E+04 | 3.07E+04 | 2.88E+04 | 2.72E+04 | 1.62E+04 | 1.69E+04 | 1.73E+04 | 1.54E+04 | 1.32E+04 | 1.66E+04 | 1.86E+04 | 3.18E+04 | 3.27E+04 | -- | -- |
| pme0376 | 2.71E+02 | 1.51E+02 | 2.72E+02 | C_15_H_12_O_5_ | [M-H]- | Naringenin (5,7,4'-Trihydroxyflavanone) | Flavonoids | Flavanones | 480-41-1 | 3 | 5.06E+03 | 4.99E+03 | 3.94E+03 | 1.02E+04 | 1.26E+04 | 1.77E+04 | 2.53E+04 | 2.38E+04 | 1.97E+04 | 1.49E+04 | 2.05E+04 | 1.79E+04 | 1.53E+04 | 1.42E+04 | 1.62E+04 | C00509 | ko00941,ko00943,ko01100,ko01110 |
| pme2960 | 2.73E+02 | 1.53E+02 | 2.72E+02 | C_15_H_12_O_5_ | [M+H]+ | Naringenin chalcone | Flavonoids | Chalcones | 73692-50-9 | 3 | 3.68E+03 | 6.74E+03 | 6.47E+03 | 1.20E+04 | 1.24E+04 | 1.30E+04 | 1.96E+04 | 1.84E+04 | 1.75E+04 | 9.05E+03 | 1.36E+04 | 1.62E+04 | 1.26E+04 | 1.29E+04 | 1.30E+04 | C06561 | ko00941,ko01100,ko01110 |
| pme3475 | 2.73E+02 | 1.53E+02 | 2.72E+02 | C_15_H_12_O_5_ | [M+H]+ | Butin | Flavonoids | Flavanones | 492-14-8 | 3 | 6.66E+03 | 6.83E+03 | 7.43E+03 | 1.34E+04 | 1.39E+04 | 1.56E+04 | 1.94E+04 | 1.54E+04 | 1.49E+04 | 1.54E+04 | 1.66E+04 | 1.43E+04 | 1.75E+04 | 1.46E+04 | 1.48E+04 | C09614 | ko00941 |
| mws0914 | 2.71E+02 | 1.51E+02 | 2.72E+02 | C_15_H_12_O_5_ | [M-H]- | Pinobanksin | Flavonoids | Flavanonols | 548-82-3 | 3 | 9.00E+00 | 9.00E+00 | 9.00E+00 | 1.11E+04 | 1.48E+04 | 1.47E+04 | 2.29E+04 | 1.88E+04 | 1.96E+04 | 1.42E+04 | 1.57E+04 | 1.33E+04 | 1.34E+04 | 1.19E+04 | 1.69E+04 | C09826 | ko00941 |
| pme3472 | 2.71E+02 | 1.08E+02 | 2.72E+02 | C_12_H_16_O_7_ | [M-H]- | Arbutin | Phenolic acids | Phenolic acids | 497-76-7 | 3 | 1.68E+04 | 1.70E+04 | 1.47E+04 | 4.23E+04 | 3.81E+04 | 4.02E+04 | 3.90E+04 | 3.95E+04 | 4.56E+04 | 5.41E+04 | 4.43E+04 | 5.72E+04 | 2.86E+04 | 3.48E+04 | 3.69E+04 | C06186 | ko00010 |
| mws1355 | 2.71E+02 | 2.25E+02 | 2.72E+02 | C_16_H_32_O_3_ | [M-H]- | 16-Hydroxyhexadecanoic acid | Lipids | Free fatty acids | 506-13-8 | 3 | 1.30E+04 | 1.29E+04 | 1.41E+04 | 9.00E+00 | 9.00E+00 | 9.00E+00 | 6.13E+03 | 6.08E+03 | 6.78E+03 | 9.00E+00 | 9.00E+00 | 9.00E+00 | 4.86E+03 | 7.34E+03 | 7.69E+03 | C18218 | ko00073,ko01100 |
| Lmmn003323 | 2.71E+02 | 2.25E+02 | 2.72E+02 | C_16_H_32_O_3_ | [M-H]- | 2-Hydroxyhexadecanoic acid | Organic acids | Organic acids | 764-67-0 | 1 | 8.91E+06 | 8.39E+06 | 8.88E+06 | 2.83E+06 | 2.51E+06 | 2.45E+06 | 9.73E+06 | 1.02E+07 | 9.76E+06 | 2.19E+06 | 2.41E+06 | 2.00E+06 | 5.46E+06 | 6.04E+06 | 6.36E+06 | -- | -- |
| Zmsp001272 | 2.74E+02 | 2.56E+02 | 2.73E+02 | C_16_H_19_NO_3_ | [M+H]+ | Norgalanthamine | Alkaloids | Alkaloids | - | 2 | 2.52E+05 | 2.36E+05 | 2.21E+05 | 1.79E+04 | 1.55E+04 | 1.58E+04 | 2.49E+05 | 2.68E+05 | 2.89E+05 | 1.05E+04 | 1.36E+04 | 1.31E+04 | 1.41E+05 | 1.52E+05 | 1.61E+05 | -- | -- |
| pmp001264 | 2.74E+02 | 2.56E+02 | 2.73E+02 | C_16_H_35_NO_2_ | [M+H]+ | Hexadecylsphingosine | Lipids | Sphingolipids | - | 2 | 5.78E+06 | 6.60E+06 | 4.63E+06 | 6.85E+06 | 7.27E+06 | 7.30E+06 | 6.27E+06 | 5.99E+06 | 5.93E+06 | 7.20E+06 | 7.22E+06 | 6.34E+06 | 5.94E+06 | 5.93E+06 | 6.27E+06 | -- | -- |
| MWSmce063 | 2.73E+02 | 2.27E+02 | 2.74E+02 | C_17_H_22_O_3_ | [M-H]- | Podocarpic acid | Terpenoids | Ditepenoids | 5947-49-9 | 2 | 7.17E+03 | 6.59E+03 | 6.97E+03 | 1.34E+04 | 1.31E+04 | 1.28E+04 | 4.02E+03 | 3.83E+03 | 4.38E+03 | 5.01E+03 | 6.32E+03 | 7.31E+03 | 7.67E+03 | 9.20E+03 | 8.20E+03 | C09171 | -- |
| pmp000292 | 2.77E+02 | 2.05E+02 | 2.76E+02 | C_15_H_16_O_5_ | [M+H]+ | Hamaudol | Others | Others | 735-46-6 | 2 | 5.63E+05 | 5.68E+05 | 5.93E+05 | 6.02E+05 | 5.90E+05 | 6.45E+05 | 3.39E+05 | 3.28E+05 | 3.52E+05 | 1.32E+05 | 1.36E+05 | 1.37E+05 | 4.31E+05 | 4.43E+05 | 4.90E+05 | C17483 | -- |
| Lmrj002793 | 2.77E+02 | 1.20E+02 | 2.76E+02 | C_14_H_16_N_2_O_4_ | [M+H]+ | Cyclo(Phe-Glu) | Amino acids and derivatives | Amino acids and derivatives | - | 2 | 3.34E+04 | 2.31E+04 | 1.96E+04 | 7.82E+03 | 6.50E+03 | 5.66E+03 | 1.43E+04 | 2.34E+04 | 2.83E+04 | 1.13E+04 | 1.36E+04 | 3.57E+03 | 1.94E+04 | 1.14E+04 | 1.80E+04 | -- | -- |
| pme1712 | 2.75E+02 | 2.57E+02 | 2.76E+02 | C_11_H_20_N_2_O_6_ | [M-H]- | L-Saccharopine | Amino acids and derivatives | Amino acids and derivatives | 997-68-2 | 3 | 2.40E+05 | 1.99E+05 | 2.31E+05 | 4.94E+04 | 4.88E+04 | 5.74E+04 | 1.04E+05 | 1.23E+05 | 1.12E+05 | 5.35E+04 | 7.01E+04 | 7.82E+04 | 1.10E+05 | 1.19E+05 | 1.26E+05 | C00449 | ko00300,ko00310,ko01100,ko01110,ko01230 |
| mws1038 | 2.77E+02 | 1.46E+02 | 2.78E+02 | C_11_H_22_N_2_O_4_S | [M-H]- | Pantetheine | Others | Others | 496-65-1 | 3 | 9.00E+00 | 9.00E+00 | 9.00E+00 | 1.86E+04 | 2.05E+04 | 2.85E+04 | 9.00E+00 | 9.00E+00 | 9.00E+00 | 9.00E+00 | 9.00E+00 | 9.00E+00 | 8.63E+03 | 6.19E+03 | 6.05E+03 | C00831 | ko00332,ko00770,ko01110 |
| MWSmce341 | 2.79E+02 | 1.49E+02 | 2.78E+02 | C_16_H_22_O_4_ | [M+H]+ | Butyl isobutyl phthalate | Phenolic acids | Phenolic acids | 17851-53-5 | 1 | 2.07E+07 | 1.69E+07 | 1.68E+07 | 2.03E+07 | 1.91E+07 | 1.94E+07 | 1.90E+07 | 1.88E+07 | 1.80E+07 | 1.95E+07 | 2.07E+07 | 2.08E+07 | 1.74E+07 | 1.79E+07 | 1.77E+07 | -- | -- |
| Lmlp012720 | 2.79E+02 | 1.49E+02 | 2.78E+02 | C_16_H_22_O_4_ | [M+H]+ | Dibutyl phthalate* | Phenolic acids | Phenolic acids | 84-74-2 | 1 | 1.24E+06 | 1.02E+06 | 1.04E+06 | 1.24E+06 | 1.16E+06 | 1.13E+06 | 1.15E+06 | 1.11E+06 | 1.10E+06 | 1.16E+06 | 1.19E+06 | 1.34E+06 | 1.16E+06 | 1.18E+06 | 1.11E+06 | C14214 | -- |
| pmp001230 | 2.79E+02 | 1.21E+02 | 2.78E+02 | C_16_H_22_O_4_ | [M+H]+ | Senkyunolide M | Others | Others | 146986-60-9 | 2 | 2.73E+06 | 2.19E+06 | 2.03E+06 | 2.57E+06 | 2.58E+06 | 2.27E+06 | 2.58E+06 | 2.63E+06 | 2.44E+06 | 2.53E+06 | 2.85E+06 | 2.93E+06 | 2.36E+06 | 2.27E+06 | 2.28E+06 | -- | -- |
| Lmxp011770 | 2.79E+02 | 1.49E+02 | 2.78E+02 | C_16_H_22_O_4_ | [M+H]+ | Diisobutyl phthalate* | Phenolic acids | Phenolic acids | 84-69-5 | 1 | 1.49E+06 | 1.14E+06 | 1.15E+06 | 1.36E+06 | 1.20E+06 | 1.18E+06 | 1.23E+06 | 1.21E+06 | 1.18E+06 | 1.31E+06 | 1.35E+06 | 1.49E+06 | 1.19E+06 | 1.22E+06 | 1.19E+06 | C15205 | -- |
| mws5035 | 2.79E+02 | 1.20E+02 | 2.78E+02 | C_15_H_22_N_2_O_3_ | [M+H]+ | L-Leucyl-L-phenylalanine | Amino acids and derivatives | Amino acids and derivatives | 56217-82-4 | 3 | 3.54E+05 | 3.26E+05 | 3.31E+05 | 3.00E+04 | 4.51E+04 | 3.75E+04 | 3.50E+04 | 5.38E+04 | 4.83E+04 | 4.74E+04 | 3.85E+04 | 5.19E+04 | 1.11E+05 | 1.16E+05 | 1.09E+05 | -- | -- |
| pmp001133 | 2.79E+02 | 1.73E+02 | 2.78E+02 | C_17_H_28_NO_2_+ | [M+H]+ | N-Methyldendrobine | Alkaloids | Sesquiterpene alkaloids | - | 2 | 7.92E+05 | 7.95E+05 | 8.76E+05 | 8.80E+05 | 7.45E+05 | 8.08E+05 | 8.32E+05 | 8.34E+05 | 9.32E+05 | 3.63E+05 | 3.97E+05 | 3.71E+05 | 8.00E+05 | 7.72E+05 | 8.92E+05 | -- | -- |
| mws0366 | 2.77E+02 | 2.77E+02 | 2.78E+02 | C_18_H_30_O_2_ | [M-H]- | γ-Linolenic Acid* | Lipids | Free fatty acids | 506-26-3 | 1 | 7.96E+06 | 7.97E+06 | 8.79E+06 | 1.37E+07 | 1.32E+07 | 1.33E+07 | 7.85E+06 | 7.46E+06 | 8.11E+06 | 9.35E+06 | 8.81E+06 | 9.07E+06 | 9.09E+06 | 1.03E+07 | 1.05E+07 | C06426 | ko00591,ko01040,ko01100 |
| pmb1650 | 2.79E+02 | 1.49E+02 | 2.78E+02 | C_18_H_30_O_2_ | [M+H]+ | Octadeca-11E,13E,15Z-trienoic acid | Lipids | Free fatty acids | 25575-00-2 | 3 | 7.75E+06 | 5.88E+06 | 6.00E+06 | 7.12E+06 | 7.81E+06 | 6.85E+06 | 7.41E+06 | 6.90E+06 | 6.96E+06 | 7.26E+06 | 7.51E+06 | 7.80E+06 | 6.55E+06 | 6.63E+06 | 6.26E+06 | -- | -- |
| Lmbn005923 | 2.77E+02 | 5.90E+01 | 2.78E+02 | C_18_H_30_O_2_ | [M-H]- | Crepenynic acid | Lipids | Free fatty acids | 2277-31-8 | 1 | 1.34E+05 | 1.40E+05 | 1.42E+05 | 2.31E+05 | 2.25E+05 | 2.27E+05 | 1.35E+05 | 1.34E+05 | 1.41E+05 | 1.58E+05 | 1.58E+05 | 1.58E+05 | 1.62E+05 | 1.84E+05 | 1.75E+05 | C07289 | ko00591 |
| pmb0889 | 2.79E+02 | 9.51E+01 | 2.78E+02 | C_18_H_30_O_2_ | [M+H]+ | Punicic acid (9Z,11E,13Z-octadecatrienoic acid) | Lipids | Free fatty acids | 544-72-9 | 3 | 1.09E+07 | 1.18E+07 | 1.27E+07 | 1.13E+07 | 1.09E+07 | 1.14E+07 | 1.28E+07 | 1.36E+07 | 1.31E+07 | 5.01E+06 | 5.60E+06 | 5.63E+06 | 1.09E+07 | 1.06E+07 | 1.21E+07 | C08364 | -- |
| mws0367 | 2.77E+02 | 2.77E+02 | 2.78E+02 | C_18_H_30_O_2_ | [M-H]- | α-Linolenic Acid* | Lipids | Free fatty acids | 463-40-1 | 1 | 7.28E+06 | 7.27E+06 | 7.36E+06 | 1.25E+07 | 1.21E+07 | 1.25E+07 | 7.48E+06 | 7.14E+06 | 7.20E+06 | 7.99E+06 | 8.45E+06 | 8.35E+06 | 8.93E+06 | 9.06E+06 | 1.03E+07 | C06427 | ko00592,ko01040,ko01100,ko01110 |
| mws0629 | 2.81E+02 | 1.66E+02 | 2.80E+02 | C_13_H_16_N_2_O_5_ | [M+H]+ | L-Aspartyl-L-Phenylalanine | Amino acids and derivatives | Amino acids and derivatives | 13433-09-5 | 3 | 2.30E+05 | 2.10E+05 | 2.03E+05 | 9.31E+04 | 8.54E+04 | 1.07E+05 | 1.31E+05 | 1.03E+05 | 1.04E+05 | 1.00E+05 | 8.00E+04 | 9.68E+04 | 1.20E+05 | 1.21E+05 | 1.50E+05 | -- | -- |
| mws1491 | 2.79E+02 | 2.79E+02 | 2.80E+02 | C_18_H_32_O_2_ | [M-H]- | Linoleic acid | Lipids | Free fatty acids | 60-33-3 | 1 | 2.83E+07 | 3.05E+07 | 3.15E+07 | 3.95E+07 | 3.58E+07 | 3.93E+07 | 3.25E+07 | 3.23E+07 | 3.43E+07 | 3.17E+07 | 3.43E+07 | 3.11E+07 | 3.57E+07 | 3.59E+07 | 3.66E+07 | C01595 | ko00591,ko01040,ko01100 |
| Lmbn006152 | 2.79E+02 | 5.90E+01 | 2.80E+02 | C_18_H_32_O_2_ | [M-H]- | (9Z,11E)-Octadecadienoic acid | Lipids | Free fatty acids | 2540-56-9 | 1 | 1.02E+05 | 9.49E+04 | 9.45E+04 | 1.13E+05 | 1.10E+05 | 1.21E+05 | 9.80E+04 | 8.99E+04 | 1.06E+05 | 1.01E+05 | 1.08E+05 | 1.00E+05 | 1.03E+05 | 1.19E+05 | 9.82E+04 | C04056 | ko00591 |
| Hmhp001812 | 2.82E+02 | 1.36E+02 | 2.81E+02 | C_11_H_15_N_5_O_4_ | [M+H]+ | 2'-O-Methyladenosine | Nucleotides and derivatives | Nucleotides and derivatives | 2140-79-6 | 1 | 1.19E+07 | 1.18E+07 | 1.15E+07 | 1.72E+06 | 2.23E+06 | 1.58E+06 | 2.55E+06 | 2.47E+06 | 2.42E+06 | 1.87E+06 | 1.88E+06 | 1.63E+06 | 5.41E+06 | 5.40E+06 | 6.08E+06 | -- | -- |
| YC512118 | 2.82E+02 | 2.47E+02 | 2.81E+02 | C_18_H_35_NO | [M+H]+ | Oleamide (9-Octadecenamide) | Lipids | Free fatty acids | 301-02-0 | 1 | 6.97E+04 | 8.31E+04 | 1.08E+05 | 6.45E+04 | 4.76E+04 | 6.73E+04 | 3.34E+04 | 6.36E+04 | 3.45E+04 | 8.98E+04 | 1.04E+05 | 6.69E+04 | 5.94E+04 | 6.82E+04 | 7.11E+04 | C19670 | -- |
| Zmhp004969 | 2.83E+02 | 1.21E+02 | 2.82E+02 | C_18_H_18_O_3_ | [M+H]+ | 4,4,5-Trihydroxy-l,l'-di-2-propenylbiphenyl | Lignans and Coumarins | Lignans | - | 3 | 1.51E+04 | 1.26E+04 | 7.56E+03 | 1.17E+04 | 7.11E+03 | 9.36E+03 | 9.00E+00 | 9.00E+00 | 9.00E+00 | 9.00E+00 | 9.00E+00 | 9.00E+00 | 8.98E+03 | 4.13E+03 | 7.13E+03 | -- | -- |
| Lmyn012331 | 2.81E+02 | 2.81E+02 | 2.82E+02 | C_18_H_34_O_2_ | [M-H]- | Petroselinic acid | Lipids | Free fatty acids | 593-39-5 | 1 | 1.45E+07 | 1.23E+07 | 1.46E+07 | 1.87E+07 | 1.96E+07 | 1.86E+07 | 1.58E+07 | 1.47E+07 | 1.44E+07 | 1.47E+07 | 1.62E+07 | 1.38E+07 | 1.65E+07 | 1.47E+07 | 1.45E+07 | C08363 | -- |
| mws2623 | 2.81E+02 | 2.81E+02 | 2.82E+02 | C_18_H_34_O_2_ | [M-H]- | 11-Octadecanoic acid(Vaccenic acid) | Lipids | Free fatty acids | 506-17-2 | 1 | 1.51E+07 | 1.46E+07 | 1.58E+07 | 2.22E+07 | 2.26E+07 | 2.11E+07 | 1.77E+07 | 1.74E+07 | 1.66E+07 | 1.62E+07 | 1.74E+07 | 1.70E+07 | 1.87E+07 | 1.70E+07 | 1.74E+07 | C21944 | -- |
| pme1178 | 2.84E+02 | 1.52E+02 | 2.83E+02 | C_10_H_13_N_5_O_5_ | [M+H]+ | Guanosine | Nucleotides and derivatives | Nucleotides and derivatives | 118-00-3 | 1 | 9.07E+07 | 9.06E+07 | 9.22E+07 | 2.35E+07 | 2.72E+07 | 2.45E+07 | 6.15E+07 | 6.25E+07 | 6.42E+07 | 2.43E+07 | 2.57E+07 | 2.30E+07 | 5.62E+07 | 5.97E+07 | 6.45E+07 | C00387 | ko00230,ko01100,ko02010 |
| Zmzp005934 | 2.84E+02 | 1.02E+02 | 2.83E+02 | C_18_H_37_NO | [M+H]+ | Stearamide | Alkaloids | Alkaloids | 124-26-5 | 1 | 4.74E+04 | 4.63E+04 | 5.70E+04 | 9.69E+04 | 7.99E+04 | 1.01E+05 | 3.50E+04 | 5.17E+04 | 4.01E+04 | 8.79E+04 | 9.66E+04 | 7.95E+04 | 5.35E+04 | 6.68E+04 | 6.82E+04 | C13846 | -- |
| Lmhn002051 | 2.83E+02 | 1.67E+02 | 2.84E+02 | C_12_H_12_O_8_ | [M-H]- | Vnilloylmalic acid | Phenolic acids | Phenolic acids | - | 3 | 3.08E+04 | 1.97E+04 | 1.52E+04 | 2.44E+04 | 2.32E+04 | 3.34E+04 | 8.80E+03 | 1.18E+04 | 1.60E+04 | 1.24E+04 | 1.54E+04 | 1.38E+04 | 1.57E+04 | 2.43E+04 | 2.45E+04 | -- | -- |
| mws0918 | 2.83E+02 | 2.68E+02 | 2.84E+02 | C_16_H_12_O_5_ | [M-H]- | Prunetin (5,4'-Dihydroxy-7-methoxyisoflavone) | Flavonoids | Isoflavones | 552-59-0 | 3 | 1.57E+05 | 1.31E+05 | 1.60E+05 | 6.13E+03 | 2.57E+04 | 7.05E+03 | 5.95E+03 | 2.64E+04 | 1.15E+04 | 3.88E+04 | 2.08E+04 | 2.62E+04 | 5.30E+04 | 6.09E+04 | 6.26E+04 | C10521 | ko00943 |
| mws4160 | 2.85E+02 | 2.70E+02 | 2.84E+02 | C_16_H_12_O_5_ | [M+H]+ | Wogonin (5,7-Dihydroxy-8-Methoxyflavone) | Flavonoids | Flavones | 632-85-9 | 3 | 1.84E+04 | 1.39E+04 | 1.67E+04 | 9.00E+00 | 9.00E+00 | 9.00E+00 | 9.00E+00 | 9.00E+00 | 9.00E+00 | 9.00E+00 | 9.00E+00 | 9.00E+00 | 5.12E+03 | 6.20E+03 | 5.84E+03 | C10197 | -- |
| mws0129 | 2.85E+02 | 2.70E+02 | 2.84E+02 | C_16_H_12_O_5_ | [M+H]+ | Genkwanin (Apigenin 7-methyl ether) | Flavonoids | Flavones | 437-64-9 | 2 | 1.08E+04 | 9.01E+03 | 8.81E+03 | 9.00E+00 | 9.00E+00 | 9.00E+00 | 9.00E+00 | 9.00E+00 | 9.00E+00 | 9.00E+00 | 9.00E+00 | 9.00E+00 | 3.94E+03 | 2.72E+03 | 3.21E+03 | C10046 | -- |
| mws0051 | 2.85E+02 | 2.70E+02 | 2.84E+02 | C_16_H_12_O_5_ | [M+H]+ | Acacetin | Flavonoids | Flavones | 480-44-4 | 3 | 1.21E+04 | 9.18E+03 | 1.20E+04 | 9.00E+00 | 9.00E+00 | 9.00E+00 | 9.00E+00 | 9.00E+00 | 9.00E+00 | 4.11E+03 | 1.51E+03 | 1.82E+03 | 4.44E+03 | 3.62E+03 | 3.58E+03 | C01470 | ko00944 |
| mws0668 | 2.83E+02 | 1.51E+02 | 2.84E+02 | C_10_H_12_N_4_O_6_ | [M-H]- | Xanthosine | Nucleotides and derivatives | Nucleotides and derivatives | 146-80-5 | 1 | 3.99E+06 | 2.74E+06 | 3.58E+06 | 3.75E+05 | 3.27E+05 | 3.63E+05 | 1.10E+06 | 1.22E+06 | 1.24E+06 | 1.36E+05 | 1.39E+05 | 1.50E+05 | 1.19E+06 | 1.37E+06 | 1.41E+06 | C01762 | ko00230,ko00232,ko01100,ko01110,ko02010 |
| pmn001491 | 2.83E+02 | 5.90E+01 | 2.84E+02 | C_15_H_24_O_5_ | [M-H]- | Dendrobiumane D | Others | Others | - | 3 | 1.35E+05 | 1.28E+05 | 1.56E+05 | 3.22E+04 | 3.87E+04 | 4.97E+04 | 1.34E+05 | 1.08E+05 | 1.29E+05 | 7.05E+04 | 7.61E+04 | 8.76E+04 | 1.16E+05 | 9.98E+04 | 1.10E+05 | -- | -- |
| mws1489 | 2.83E+02 | 2.83E+02 | 2.84E+02 | C_18_H_36_O_2_ | [M-H]- | Stearic Acid | Lipids | Free fatty acids | 57-11-4 | 3 | 3.10E+07 | 2.94E+07 | 2.65E+07 | 2.59E+07 | 3.98E+07 | 4.01E+07 | 3.44E+07 | 3.75E+07 | 3.04E+07 | 3.37E+07 | 3.54E+07 | 3.35E+07 | 3.62E+06 | 4.19E+06 | 3.98E+06 | C01530 | ko00061,ko01040 |
| pmp000019 | 2.87E+02 | 2.03E+02 | 2.86E+02 | C_16_H_14_O_5_ | [M+H]+ | Pabulenol | Lignans and Coumarins | Coumarins | 33889-70-2 | 2 | 1.83E+07 | 1.55E+07 | 1.65E+07 | 1.65E+07 | 1.71E+07 | 1.74E+07 | 1.08E+07 | 1.38E+07 | 1.26E+07 | 1.33E+07 | 1.29E+07 | 1.35E+07 | 1.44E+07 | 1.64E+07 | 1.69E+07 | -- | -- |
| MWSslk233 | 2.87E+02 | 2.03E+02 | 2.86E+02 | C_16_H_14_O_5_ | [M+H]+ | Oxypeucedanin | Lignans and Coumarins | Coumarins | 737-52-0 | 1 | 2.43E+07 | 2.38E+07 | 2.44E+07 | 2.60E+07 | 2.59E+07 | 2.49E+07 | 2.30E+07 | 2.24E+07 | 2.43E+07 | 2.23E+07 | 2.22E+07 | 2.16E+07 | 2.50E+07 | 2.35E+07 | 2.45E+07 | C09282 | -- |
| pmp000021 | 2.87E+02 | 2.03E+02 | 2.86E+02 | C_16_H_14_O_5_ | [M+H]+ | Isooxypeucedanine | Lignans and Coumarins | Coumarins | 5058-15-1 | 1 | 2.41E+07 | 2.40E+07 | 2.37E+07 | 2.50E+07 | 2.52E+07 | 2.59E+07 | 2.33E+07 | 2.30E+07 | 2.36E+07 | 2.22E+07 | 2.25E+07 | 2.30E+07 | 2.49E+07 | 2.54E+07 | 2.40E+07 | -- | -- |
| pme2289 | 2.87E+02 | 1.19E+02 | 2.86E+02 | C_20_H_30_O | [M+H]+ | Retinol (Vitamin A1) | Others | Vitamin | 68-26-8 | 3 | 4.19E+04 | 3.49E+04 | 3.55E+04 | 4.05E+04 | 4.09E+04 | 3.84E+04 | 1.86E+04 | 2.87E+04 | 2.39E+04 | 2.60E+04 | 2.74E+04 | 3.15E+04 | 2.91E+04 | 3.27E+04 | 3.80E+04 | C00473 | ko01100,ko01240 |
| Zmyn005252 | 2.85E+02 | 2.39E+02 | 2.86E+02 | C_17_H_34_O_3_ | [M-H]- | 3-Hydroxy-palmitic acid methyl ester | Lipids | Free fatty acids | 51883-36-4 | 1 | 8.15E+03 | 7.11E+03 | 5.09E+03 | 7.27E+03 | 5.57E+03 | 3.34E+03 | 1.12E+04 | 1.14E+04 | 9.03E+03 | 4.11E+03 | 3.77E+03 | 4.58E+03 | 8.03E+03 | 6.46E+03 | 7.85E+03 | C11849 | -- |
| mws1436 | 2.87E+02 | 9.72E+01 | 2.86E+02 | C_18_H_38_O_2_ | [M+H]+ | 1,18-Octadecanediol | Lipids | Free fatty acids | 3155-43-9 | 3 | 1.89E+04 | 2.10E+04 | 1.86E+04 | 2.93E+04 | 2.19E+04 | 2.34E+04 | 1.07E+04 | 8.18E+03 | 6.30E+03 | 1.55E+04 | 1.52E+04 | 1.72E+04 | 1.76E+04 | 1.77E+04 | 2.10E+04 | -- | -- |
| MWSmce421 | 2.87E+02 | 2.18E+02 | 2.88E+02 | C_16_H_16_O_5_ | [M-H]- | Alkannin | Quinones | Quinones | 517-88-4 | 2 | 1.05E+04 | 7.79E+03 | 8.02E+03 | 1.15E+04 | 9.92E+03 | 9.91E+03 | 6.51E+03 | 7.44E+03 | 6.97E+03 | 7.42E+03 | 8.04E+03 | 8.21E+03 | 6.96E+03 | 9.82E+03 | 8.52E+03 | C10292 | -- |
| Zmbp008068 | 2.89E+02 | 1.75E+02 | 2.88E+02 | C_16_H_16_O_5_ | [M+H]+ | Acetyllomatin | Lignans and Coumarins | Coumarins | - | 2 | 1.67E+05 | 1.75E+05 | 1.79E+05 | 2.04E+05 | 1.78E+05 | 1.84E+05 | 1.64E+05 | 1.67E+05 | 1.65E+05 | 1.59E+05 | 1.79E+05 | 1.70E+05 | 1.78E+05 | 1.91E+05 | 1.84E+05 | -- | -- |
| pmn001686 | 2.87E+02 | 2.41E+02 | 2.88E+02 | C_16_H_32_O_4_ | [M-H]- | 10,16-Dihydroxypalmitic acid | Lipids | Free fatty acids | 3233-90-7 | 3 | 1.96E+04 | 1.48E+04 | 2.06E+04 | 9.00E+00 | 9.00E+00 | 9.00E+00 | 9.00E+00 | 9.00E+00 | 9.00E+00 | 9.00E+00 | 9.00E+00 | 9.00E+00 | 5.86E+03 | 7.17E+03 | 5.50E+03 | C08285 | ko00073 |
| pmb3081 | 2.89E+02 | 9.70E+01 | 2.90E+02 | C_6_H_11_PO_11_ | [M-H]- | Glucarate O-Phosphoric acid | Others | Saccharides and Alcohols | - | 3 | 5.71E+06 | 5.69E+06 | 4.58E+06 | 7.94E+06 | 9.54E+06 | 9.12E+06 | 7.73E+06 | 7.51E+06 | 7.80E+06 | 1.29E+07 | 1.01E+07 | 1.18E+07 | 8.71E+06 | 8.92E+06 | 7.66E+06 | -- | -- |
| pme3163 | 2.89E+02 | 9.70E+01 | 2.90E+02 | C_7_H_15_O_10_P | [M-H]- | D-Sedoheptuiose 7-phosphate | Others | Saccharides and Alcohols | 2646-35-7 | 3 | 2.81E+06 | 2.84E+06 | 3.23E+06 | 5.37E+06 | 5.07E+06 | 5.28E+06 | 4.05E+06 | 4.08E+06 | 3.85E+06 | 5.73E+06 | 6.16E+06 | 6.68E+06 | 4.34E+06 | 4.94E+06 | 4.12E+06 | -- | -- |
| Zmdp001928 | 2.91E+02 | 1.30E+02 | 2.90E+02 | C_11_H_18_N_2_O_5_S | [M+H]+ | γ-L-Glutamyl-S-(trans-1-propenyl)-L-cysteine | Amino acids and derivatives | Amino acids and derivatives | - | 2 | 5.96E+04 | 5.07E+04 | 5.62E+04 | 1.78E+04 | 1.32E+04 | 1.14E+04 | 4.03E+04 | 4.75E+04 | 5.79E+04 | 1.38E+04 | 2.66E+04 | 1.48E+04 | 3.75E+04 | 3.64E+04 | 4.36E+04 | -- | -- |
| pmb2657 | 2.89E+02 | 9.71E+01 | 2.90E+02 | C_10_H_18_N_4_O_6_ | [M-H]- | Argininosuccinic acid | Organic acids | Organic acids | 2387-71-5 | 3 | 9.76E+04 | 7.92E+04 | 9.94E+04 | 4.36E+05 | 4.48E+05 | 4.55E+05 | 2.56E+05 | 2.49E+05 | 2.60E+05 | 3.14E+05 | 3.64E+05 | 3.41E+05 | 2.64E+05 | 2.84E+05 | 3.24E+05 | C03406 | ko00220,ko00250,ko01100,ko01110,ko01230 |
| pmp000295 | 2.93E+02 | 2.21E+02 | 2.92E+02 | C_15_H_16_O_6_ | [M+H]+ | Angelicain | Others | Others | 49624-66-0 | 1 | 7.58E+04 | 7.01E+04 | 5.11E+04 | 4.05E+04 | 4.52E+04 | 3.98E+04 | 3.51E+04 | 2.73E+04 | 2.76E+04 | 1.59E+04 | 1.84E+04 | 1.40E+04 | 3.94E+04 | 4.16E+04 | 4.06E+04 | -- | -- |
| Zmyn004548 | 2.91E+02 | 2.35E+02 | 2.92E+02 | C_18_H_28_O_3_ | [M-H]- | 12-Oxo-phytodienoic acid | Lipids | Free fatty acids | 85551-10-6 | 3 | 1.11E+04 | 1.24E+04 | 1.36E+04 | 3.38E+04 | 3.01E+04 | 3.42E+04 | 2.57E+04 | 2.45E+04 | 2.32E+04 | 2.76E+04 | 3.01E+04 | 2.87E+04 | 2.42E+04 | 2.48E+04 | 2.61E+04 | C01226 | ko00592,ko01100,ko01110 |
| MWSmce689 | 2.93E+02 | 9.51E+01 | 2.92E+02 | C_19_H_32_O_2_ | [M+H]+ | Methyl linolenate | Lipids | Free fatty acids | 301-00-8 | 2 | 5.48E+05 | 5.18E+05 | 5.84E+05 | 8.21E+05 | 6.99E+05 | 7.59E+05 | 5.93E+05 | 6.69E+05 | 7.06E+05 | 2.53E+05 | 2.70E+05 | 2.67E+05 | 5.52E+05 | 5.43E+05 | 5.61E+05 | -- | -- |
| Zmdn001564 | 2.93E+02 | 1.64E+02 | 2.94E+02 | C_14_H_18_N_2_O_5_ | [M-H]- | γ-Glutamylphenylalanine | Amino acids and derivatives | Amino acids and derivatives | 7432-24-8 | 1 | 1.84E+05 | 1.70E+05 | 1.93E+05 | 5.59E+04 | 5.21E+04 | 5.77E+04 | 4.06E+05 | 4.10E+05 | 4.31E+05 | 1.85E+05 | 1.79E+05 | 2.07E+05 | 1.83E+05 | 1.94E+05 | 2.47E+05 | -- | -- |
| Hmln007772 | 2.93E+02 | 2.21E+02 | 2.94E+02 | C_17_H_26_O_4_ | [M-H]- | Nordihydrocapsiate | Others | Others | 220012-53-3 | 2 | 3.47E+06 | 3.28E+06 | 2.89E+06 | 4.22E+06 | 4.35E+06 | 4.54E+06 | 5.07E+06 | 3.07E+06 | 4.99E+06 | 3.93E+06 | 4.41E+06 | 4.91E+06 | 5.53E+06 | 5.67E+06 | 6.17E+06 | -- | -- |
| pmb2786 | 2.93E+02 | 2.35E+02 | 2.94E+02 | C_18_H_30_O_3_ | [M-H]- | 9-Hydroxy-10,12,15-octadecatrienoic acid | Lipids | Free fatty acids | 89886-42-0 | 2 | 5.65E+05 | 5.50E+05 | 6.36E+05 | 3.52E+05 | 3.74E+05 | 3.63E+05 | 4.01E+05 | 3.86E+05 | 4.13E+05 | 1.48E+05 | 1.56E+05 | 1.76E+05 | 3.93E+05 | 4.06E+05 | 4.20E+05 | C16326 | ko00592 |
| Lmmn006306 | 2.93E+02 | 2.93E+02 | 2.94E+02 | C_18_H_30_O_3_ | [M-H]- | Machilusolide D | Others | Others | - | 2 | 4.56E+06 | 4.76E+06 | 4.75E+06 | 3.89E+06 | 3.70E+06 | 3.72E+06 | 3.56E+06 | 3.45E+06 | 3.72E+06 | 1.62E+06 | 1.77E+06 | 1.67E+06 | 3.50E+06 | 3.57E+06 | 3.94E+06 | -- | -- |
| pmb2792 | 2.93E+02 | 1.93E+02 | 2.94E+02 | C_18_H_30_O_3_ | [M-H]- | 13-Hydroxy-6,9,11-octadecatrienoic acid | Lipids | Free fatty acids | 74784-20-6 | 3 | 3.30E+05 | 1.28E+05 | 9.59E+04 | 2.16E+05 | 3.61E+05 | 1.62E+05 | 1.10E+05 | 1.03E+05 | 1.18E+05 | 2.54E+05 | 3.84E+05 | 1.69E+05 | 1.86E+05 | 2.01E+05 | 2.05E+05 | -- | -- |
| Zmpn003368 | 2.93E+02 | 1.95E+02 | 2.94E+02 | C_18_H_30_O_3_ | [M-H]- | 13S-Hydroxy-9Z,11E,15Z-octadecatrienoic acid | Lipids | Free fatty acids | 87984-82-5 | 3 | 6.09E+04 | 5.87E+04 | 6.38E+04 | 1.53E+05 | 1.39E+05 | 1.47E+05 | 8.30E+04 | 7.68E+04 | 7.57E+04 | 4.99E+04 | 5.95E+04 | 4.86E+04 | 8.75E+04 | 9.32E+04 | 9.48E+04 | C16316 | ko00592 |
| pmb2787 | 2.93E+02 | 2.75E+02 | 2.94E+02 | C_18_H_30_O_3_ | [M-H]- | 9-Oxo-10E,12Z-octadecadienoic acid | Lipids | Free fatty acids | 54232-59-6 | 3 | 3.77E+05 | 3.53E+05 | 3.96E+05 | 2.31E+05 | 2.26E+05 | 2.48E+05 | 2.54E+05 | 2.44E+05 | 2.59E+05 | 9.19E+04 | 9.88E+04 | 9.55E+04 | 2.40E+05 | 2.56E+05 | 2.69E+05 | C14766 | ko00591 |
| Zmyn004732 | 2.93E+02 | 1.85E+02 | 2.94E+02 | C_18_H_30_O_3_ | [M-H]- | 2R-hydroxy-9Z,12Z,15Z-octadecatrienoic acid | Lipids | Free fatty acids | - | 1 | 2.24E+06 | 2.20E+06 | 2.33E+06 | 1.87E+06 | 1.69E+06 | 1.89E+06 | 1.78E+06 | 1.83E+06 | 1.86E+06 | 8.08E+05 | 8.88E+05 | 7.90E+05 | 1.66E+06 | 1.79E+06 | 1.87E+06 | C16342 | ko00592 |
| Lmbn005443 | 2.93E+02 | 2.35E+02 | 2.94E+02 | C_18_H_30_O_3_ | [M-H]- | 13-KODE; (9Z,11E)-13-Oxooctadeca-9,11-dienoic acid | Lipids | Free fatty acids | 54739-30-9 | 2 | 6.24E+05 | 6.32E+05 | 6.74E+05 | 3.79E+05 | 3.67E+05 | 3.93E+05 | 4.40E+05 | 4.24E+05 | 4.20E+05 | 1.63E+05 | 1.72E+05 | 1.70E+05 | 4.03E+05 | 4.20E+05 | 4.48E+05 | C14765 | ko00591 |
| Lmcn009539 | 2.93E+02 | 2.93E+02 | 2.94E+02 | C_19_H_34_O_2_ | [M-H]- | E,E,Z-1,3,12-Nonadecatriene-5,14-diol | Lipids | Free fatty acids | - | 1 | 3.99E+04 | 3.45E+04 | 4.40E+04 | 1.03E+05 | 8.66E+04 | 9.73E+04 | 7.33E+04 | 7.41E+04 | 7.37E+04 | 7.16E+04 | 7.97E+04 | 6.65E+04 | 7.12E+04 | 7.44E+04 | 7.05E+04 | -- | -- |
| Lmqp000329 | 2.96E+02 | 1.04E+02 | 2.95E+02 | C_8_H_14_N_3_O_7_P | [M+H]+ | 5-Aminoimidazole ribonucleotide | Nucleotides and derivatives | Nucleotides and derivatives | 25635-88-5 | 1 | 5.08E+04 | 6.01E+04 | 4.94E+04 | 7.30E+05 | 6.65E+05 | 6.58E+05 | 9.64E+05 | 1.08E+06 | 1.11E+06 | 4.55E+05 | 4.01E+05 | 4.63E+05 | 5.82E+05 | 6.04E+05 | 5.99E+05 | -- | -- |
| Hmhp011280 | 2.96E+02 | 2.07E+02 | 2.95E+02 | C_18_H_33_NO_2_ | [M+H]+ | Tetrahydrobungeanool | Alkaloids | Alkaloids | - | 3 | 4.45E+03 | 4.14E+03 | 3.47E+03 | 2.64E+03 | 3.98E+03 | 3.41E+03 | 2.90E+03 | 2.75E+03 | 4.19E+03 | 1.64E+03 | 2.02E+03 | 1.25E+03 | 2.67E+03 | 4.57E+03 | 5.24E+03 | -- | -- |
| Hmqp005455 | 2.97E+02 | 8.11E+01 | 2.96E+02 | C_18_H_32_O_3_ | [M+H]+ | 15(R)-Hydroxylinoleic Acid | Lipids | Free fatty acids | 177931-23-6 | 2 | 6.57E+05 | 5.79E+05 | 6.87E+05 | 4.43E+05 | 4.42E+05 | 4.91E+05 | 7.33E+05 | 7.32E+05 | 7.50E+05 | 3.10E+05 | 3.03E+05 | 2.83E+05 | 5.27E+05 | 4.95E+05 | 5.97E+05 | -- | -- |
| Hmqp005411 | 2.97E+02 | 8.11E+01 | 2.96E+02 | C_18_H_32_O_3_ | [M+H]+ | 9-Oxo-12Z-Octadecenoic acid | Lipids | Free fatty acids | 112543-32-5 | 2 | 6.35E+05 | 6.31E+05 | 7.38E+05 | 4.98E+05 | 4.50E+05 | 4.22E+05 | 7.68E+05 | 7.65E+05 | 7.87E+05 | 2.97E+05 | 2.86E+05 | 2.77E+05 | 5.37E+05 | 4.86E+05 | 6.30E+05 | -- | -- |
| pmb2799 | 2.95E+02 | 1.95E+02 | 2.96E+02 | C_18_H_32_O_3_ | [M-H]- | 12,13-Epoxy-9-Octadecenoic Acid | Lipids | Free fatty acids | 6799-85-5 | 2 | 8.39E+05 | 8.38E+05 | 9.48E+05 | 5.96E+05 | 5.63E+05 | 5.48E+05 | 7.65E+05 | 7.78E+05 | 8.36E+05 | 3.03E+05 | 3.00E+05 | 2.99E+05 | 6.33E+05 | 6.49E+05 | 6.91E+05 | C14826 | ko00591,ko01100 |
| Lmbn005369 | 2.95E+02 | 1.95E+02 | 2.96E+02 | C_18_H_32_O_3_ | [M-H]- | 13(S)-HODE;13(S)-Hydroxyoctadeca-9Z,11E-dienoic acid* | Lipids | Free fatty acids | 10219-69-9 | 1 | 1.90E+06 | 1.72E+06 | 1.89E+06 | 2.73E+06 | 2.60E+06 | 2.61E+06 | 3.44E+06 | 3.30E+06 | 3.25E+06 | 1.24E+06 | 1.30E+06 | 1.34E+06 | 2.29E+06 | 2.44E+06 | 2.59E+06 | C14762 | ko00591 |
| Rfmb091 | 2.95E+02 | 1.95E+02 | 2.96E+02 | C_18_H_32_O_3_ | [M-H]- | 9S-Hydroxy-10E,12Z-octadecadienoic acid* | Lipids | Free fatty acids | 15514-85-9 | 1 | 1.94E+06 | 1.82E+06 | 1.92E+06 | 2.69E+06 | 2.66E+06 | 2.68E+06 | 3.38E+06 | 3.44E+06 | 3.42E+06 | 1.32E+06 | 1.42E+06 | 1.35E+06 | 2.36E+06 | 2.49E+06 | 2.58E+06 | C14767 | ko00591 |
| Lmbn005662 | 2.95E+02 | 1.71E+02 | 2.96E+02 | C_18_H_32_O_3_ | [M-H]- | 9(10)-EpOME;(9R,10S)-(12Z)-9,10-Epoxyoctadecenoic acid | Lipids | Free fatty acids | 16833-56-0 | 1 | 5.61E+05 | 5.63E+05 | 5.73E+05 | 7.02E+05 | 6.98E+05 | 7.17E+05 | 8.72E+05 | 9.01E+05 | 9.19E+05 | 4.08E+05 | 4.35E+05 | 3.98E+05 | 6.50E+05 | 6.58E+05 | 6.99E+05 | C14825 | ko00591,ko01100 |
| pme1474 | 2.98E+02 | 1.36E+02 | 2.97E+02 | C_11_H_15_N_5_O_3_S | [M+H]+ | 5'-Deoxy-5'-(methylthio)adenosine | Nucleotides and derivatives | Nucleotides and derivatives | 2457-80-9 | 1 | 1.49E+06 | 1.64E+06 | 1.40E+06 | 1.57E+06 | 1.51E+06 | 1.43E+06 | 2.46E+06 | 2.46E+06 | 2.49E+06 | 1.80E+06 | 1.92E+06 | 2.02E+06 | 1.93E+06 | 2.02E+06 | 2.04E+06 | C00170 | ko00270,ko00908,ko01100 |
| Zmwp002855 | 2.98E+02 | 1.36E+02 | 2.97E+02 | C_17_H_15_NO_4_ | [M+H]+ | Oxoassoanine N-oxide | Alkaloids | Plumerane | - | 2 | 1.75E+06 | 1.78E+06 | 1.72E+06 | 1.48E+06 | 1.59E+06 | 1.60E+06 | 2.52E+06 | 2.42E+06 | 2.71E+06 | 1.96E+06 | 2.20E+06 | 2.28E+06 | 1.90E+06 | 2.15E+06 | 2.12E+06 | -- | -- |
| pmb0197 | 2.98E+02 | 1.66E+02 | 2.97E+02 | C_11_H_15_N_5_O_5_ | [M+H]+ | N7-Methylguanosine | Nucleotides and derivatives | Nucleotides and derivatives | 20244-86-4 | 3 | 3.54E+05 | 3.33E+05 | 3.83E+05 | 2.16E+05 | 2.57E+05 | 2.10E+05 | 3.93E+05 | 3.48E+05 | 3.63E+05 | 2.04E+05 | 2.13E+05 | 1.70E+05 | 2.90E+05 | 2.82E+05 | 3.40E+05 | C20674 | -- |
| Lmhp007461 | 2.99E+02 | 2.31E+02 | 2.98E+02 | C_16_H_10_O_6_ | [M+H]+ | Aflatoxin P1 | Others | Others | 32215-02-4 | 2 | 1.05E+07 | 9.35E+06 | 9.49E+06 | 1.53E+07 | 1.34E+07 | 1.43E+07 | 5.92E+06 | 5.66E+06 | 6.43E+06 | 6.28E+06 | 6.13E+06 | 6.23E+06 | 8.94E+06 | 9.81E+06 | 1.03E+07 | C19587 | -- |
| MWSmce224 | 2.99E+02 | 1.63E+02 | 2.98E+02 | C_19_H_22_O_3_ | [M+H]+ | Aurapten | Lignans and Coumarins | Coumarins | 495-02-3 | 3 | 1.46E+05 | 1.18E+05 | 1.31E+05 | 1.59E+05 | 1.56E+05 | 1.53E+05 | 1.32E+05 | 1.34E+05 | 1.56E+05 | 1.83E+05 | 1.63E+05 | 2.02E+05 | 1.49E+05 | 1.48E+05 | 1.57E+05 | -- | -- |
| Zmgn005057 | 2.97E+02 | 1.83E+02 | 2.98E+02 | C_18_H_34_O_3_ | [M-H]- | 9,10-Epoxyoctadecanoic Acid | Lipids | Free fatty acids | 2443-39-2 | 2 | 7.73E+03 | 6.71E+03 | 6.59E+03 | 8.56E+03 | 6.57E+03 | 5.29E+03 | 9.36E+03 | 9.82E+03 | 9.45E+03 | 4.05E+03 | 3.75E+03 | 5.13E+03 | 4.70E+03 | 6.64E+03 | 8.12E+03 | C19418 | ko00073 |
| Zmyn004714 | 2.97E+02 | 1.83E+02 | 2.98E+02 | C_18_H_34_O_3_ | [M-H]- | Ricinoleic acid | Lipids | Free fatty acids | 141-22-0 | 3 | 5.73E+04 | 5.56E+04 | 5.81E+04 | 1.58E+05 | 1.56E+05 | 1.70E+05 | 8.46E+04 | 8.17E+04 | 8.58E+04 | 1.06E+05 | 1.15E+05 | 1.13E+05 | 1.08E+05 | 1.13E+05 | 1.15E+05 | C08365 | -- |
| pmf0297 | 2.97E+02 | 1.83E+02 | 2.98E+02 | C_20_H_42_O | [M-H]- | 1-Eicosanol | Lipids | Free fatty acids | 629-96-9 | 3 | 6.78E+04 | 6.21E+04 | 6.01E+04 | 1.63E+05 | 1.71E+05 | 1.84E+05 | 9.08E+04 | 9.22E+04 | 9.25E+04 | 1.09E+05 | 1.16E+05 | 1.22E+05 | 1.06E+05 | 1.16E+05 | 1.25E+05 | -- | -- |
| ML10195036 | 3.00E+02 | 6.01E+01 | 2.99E+02 | C_18_H_37_NO_2_ | [M+H]+ | 3-Dehydrosphinganine | Lipids | Sphingolipids | 16105-69-4 | 1 | 9.84E+04 | 9.67E+04 | 9.67E+04 | 1.06E+04 | 6.89E+03 | 9.40E+03 | 4.13E+04 | 4.85E+04 | 4.18E+04 | 7.27E+03 | 1.22E+04 | 1.03E+04 | 3.45E+04 | 3.87E+04 | 4.60E+04 | C02934 | ko00600,ko01100 |
| pmp000001 | 3.01E+02 | 2.86E+02 | 3.00E+02 | C_16_H_12_O_6_ | [M+H]+ | Hispidulin (5,7,4'-Trihydroxy-6-methoxyflavone) | Flavonoids | Flavones | 1447-88-7 | 3 | 6.88E+04 | 5.63E+04 | 8.67E+04 | 1.80E+04 | 1.66E+04 | 1.89E+04 | 1.90E+04 | 2.79E+04 | 1.95E+04 | 2.26E+04 | 2.55E+04 | 2.72E+04 | 3.76E+04 | 4.10E+04 | 4.12E+04 | C10058 | -- |
| mws0058 | 2.99E+02 | 2.84E+02 | 3.00E+02 | C_16_H_12_O_6_ | [M-H]- | Diosmetin (5,7,3'-Trihydroxy-4'-methoxyflavone) | Flavonoids | Flavanones | 520-34-3 | 2 | 5.07E+04 | 3.82E+04 | 5.84E+04 | 2.11E+04 | 1.95E+04 | 1.97E+04 | 2.08E+04 | 2.78E+04 | 2.31E+04 | 2.33E+04 | 2.90E+04 | 3.18E+04 | 3.26E+04 | 3.07E+04 | 4.36E+04 | C10038 | -- |
| Lmhn004976 | 2.99E+02 | 2.84E+02 | 3.00E+02 | C_16_H_12_O_6_ | [M-H]- | Aracarpene 2 | Flavonoids | Isoflavones | - | 1 | 4.70E+04 | 3.59E+04 | 5.88E+04 | 1.67E+04 | 1.99E+04 | 1.81E+04 | 2.16E+04 | 2.81E+04 | 2.55E+04 | 3.21E+04 | 2.50E+04 | 3.43E+04 | 3.74E+04 | 4.39E+04 | 3.95E+04 | -- | -- |
| Zmhp003514 | 3.01E+02 | 2.86E+02 | 3.00E+02 | C_16_H_12_O_6_ | [M+H]+ | 6,7,8-Tetrahydroxy-5-methoxyflavone | Flavonoids | Flavones | - | 3 | 7.43E+04 | 5.20E+04 | 9.48E+04 | 1.78E+04 | 1.27E+04 | 1.99E+04 | 1.76E+04 | 3.49E+04 | 2.81E+04 | 2.66E+04 | 1.96E+04 | 3.36E+04 | 4.17E+04 | 4.32E+04 | 5.01E+04 | -- | -- |
| Xmyn008071 | 2.99E+02 | 2.84E+02 | 3.00E+02 | C_16_H_12_O_6_ | [M-H]- | Gnetifolin B | Flavonoids | Flavones | 140671-06-3 | 2 | 3.75E+04 | 3.35E+04 | 5.38E+04 | 2.13E+04 | 2.03E+04 | 1.77E+04 | 1.93E+04 | 2.60E+04 | 2.09E+04 | 2.37E+04 | 1.87E+04 | 2.91E+04 | 3.08E+04 | 3.08E+04 | 3.13E+04 | -- | -- |
| Zmhn001926 | 2.99E+02 | 1.37E+02 | 3.00E+02 | C_13_H_16_O_8_ | [M-H]- | 1-O-Salicyl-D-glucose | Phenolic acids | Phenolic acids | 60517-74-0 | 1 | 4.37E+06 | 4.43E+06 | 6.15E+06 | 6.41E+06 | 6.96E+06 | 6.38E+06 | 1.10E+06 | 1.12E+06 | 1.27E+06 | 1.44E+06 | 1.42E+06 | 1.54E+06 | 3.66E+06 | 3.88E+06 | 4.04E+06 | -- | -- |
| Hmtn001302 | 2.99E+02 | 1.37E+02 | 3.00E+02 | C_13_H_16_O_8_ | [M-H]- | Glucosyloxybenzoic acid | Phenolic acids | Phenolic acids | - | 1 | 4.20E+06 | 4.25E+06 | 5.86E+06 | 6.17E+06 | 7.08E+06 | 6.39E+06 | 9.78E+05 | 1.06E+06 | 1.08E+06 | 1.47E+06 | 1.52E+06 | 1.32E+06 | 3.43E+06 | 3.83E+06 | 4.12E+06 | -- | -- |
| pmb3142 | 2.99E+02 | 1.37E+02 | 3.00E+02 | C_13_H_16_O_8_ | [M-H]- | Salicylic acid-2-O-glucoside | Phenolic acids | Phenolic acids | 10366-91-3 | 3 | 1.47E+06 | 1.48E+06 | 1.32E+06 | 2.05E+06 | 2.14E+06 | 2.31E+06 | 1.19E+06 | 1.23E+06 | 1.30E+06 | 1.38E+06 | 1.44E+06 | 1.48E+06 | 1.55E+06 | 1.66E+06 | 1.69E+06 | -- | -- |
| pmc1990 | 2.99E+02 | 2.23E+02 | 3.00E+02 | C_17_H_16_O_5_ | [M-H]- | 4'-Hydroxy-5,7-dimethoxyflavanone | Flavonoids | Flavanones | - | 3 | 5.90E+05 | 6.07E+05 | 5.60E+05 | 6.45E+05 | 6.66E+05 | 6.47E+05 | 7.29E+05 | 6.87E+05 | 7.58E+05 | 5.87E+05 | 7.18E+05 | 6.63E+05 | 1.67E+06 | 1.64E+06 | 2.09E+06 | -- | -- |
| pmp000296 | 3.01E+02 | 2.18E+02 | 3.00E+02 | C_17_H_16_O_5_ | [M+H]+ | Phellopterin | Lignans and Coumarins | Coumarins | 2543-94-4 | 2 | 3.72E+06 | 3.66E+06 | 3.24E+06 | 3.71E+06 | 3.27E+06 | 3.48E+06 | 2.72E+06 | 2.73E+06 | 2.50E+06 | 2.84E+06 | 2.99E+06 | 2.89E+06 | 3.23E+06 | 3.12E+06 | 3.35E+06 | C17047 | -- |
| mws2367 | 2.99E+02 | 1.19E+02 | 3.00E+02 | C_14_H_20_O_7_ | [M-H]- | Salidroside | Phenolic acids | Phenolic acids | 10338-51-9 | 3 | 1.22E+04 | 1.05E+04 | 9.98E+03 | 1.53E+04 | 1.44E+04 | 1.14E+04 | 3.84E+03 | 8.31E+03 | 5.97E+03 | 2.83E+03 | 5.84E+03 | 5.23E+03 | 6.65E+03 | 1.03E+04 | 1.38E+04 | C06046 | ko00350,ko01100 |
| Cmmn012461 | 2.99E+02 | 2.99E+02 | 3.00E+02 | C_20_H_28_O_2_ | [M-H]- | Dehydroabietic acid | Terpenoids | Ditepenoids | 1740-19-8 | 2 | 1.39E+05 | 1.36E+05 | 1.47E+05 | 1.86E+05 | 1.22E+05 | 1.21E+05 | 2.08E+05 | 1.75E+05 | 3.70E+05 | 1.48E+05 | 1.41E+05 | 1.34E+05 | 3.30E+05 | 3.43E+05 | 4.00E+05 | -- | -- |
| pme2816 | 3.01E+02 | 2.09E+02 | 3.00E+02 | C_20_H_28_O_2_ | [M+H]+ | 4-Oxoretinol | Others | Vitamin | 62702-55-0 | 3 | 5.85E+04 | 5.98E+04 | 5.17E+04 | 6.76E+04 | 6.13E+04 | 7.18E+04 | 4.89E+04 | 5.31E+04 | 5.63E+04 | 5.10E+04 | 5.74E+04 | 5.45E+04 | 5.25E+04 | 5.40E+04 | 5.43E+04 | C16683 | -- |
| MWS4295 | 2.99E+02 | 2.53E+02 | 3.00E+02 | C_18_H_36_O_3_ | [M-H]- | DL-2-hydroxystearic acid | Lipids | Free fatty acids | 629-22-1 | 1 | 2.49E+04 | 2.78E+04 | 2.64E+04 | 1.21E+04 | 1.27E+04 | 9.00E+03 | 2.57E+04 | 2.63E+04 | 2.81E+04 | 9.37E+03 | 7.77E+03 | 8.90E+03 | 1.89E+04 | 1.83E+04 | 1.69E+04 | -- | -- |
| Zmyn005384 | 2.99E+02 | 2.53E+02 | 3.00E+02 | C_18_H_36_O_3_ | [M-H]- | 2R-Hydroxyoctadecanoic Acid | Lipids | Free fatty acids | 26633-48-7 | 2 | 2.15E+04 | 2.64E+04 | 2.30E+04 | 1.23E+04 | 1.27E+04 | 9.12E+03 | 2.80E+04 | 2.58E+04 | 2.47E+04 | 7.72E+03 | 1.06E+04 | 7.89E+03 | 2.15E+04 | 1.97E+04 | 1.59E+04 | C03042 | -- |
| Lmqn008288 | 2.99E+02 | 2.99E+02 | 3.00E+02 | C_18_H_36_O_3_ | [M-H]- | 3-Hydroxyoctadecanoic Acid | Lipids | Free fatty acids | 45261-96-9 | 2 | 1.21E+05 | 1.36E+05 | 1.35E+05 | 1.74E+05 | 1.16E+05 | 1.17E+05 | 1.95E+05 | 1.61E+05 | 3.46E+05 | 1.39E+05 | 1.34E+05 | 1.24E+05 | 3.24E+05 | 3.39E+05 | 3.75E+05 | -- | -- |
| pmb3079 | 3.00E+02 | 9.69E+01 | 3.01E+02 | C_8_H_16_NO_9_P | [M-H]- | N-Acetyl-D-glucosamine-1-phosphate | Others | Saccharides and Alcohols | 6866-69-9 | 3 | 7.16E+04 | 7.74E+04 | 7.18E+04 | 4.83E+04 | 5.82E+04 | 6.89E+04 | 3.89E+04 | 3.23E+04 | 5.04E+04 | 8.73E+04 | 9.34E+04 | 9.27E+04 | 6.63E+04 | 7.00E+04 | 5.39E+04 | C04256 | -- |
| pme2954 | 3.03E+02 | 1.37E+02 | 3.02E+02 | C_15_H_10_O_7_ | [M+H]+ | Quercetin | Flavonoids | Flavonols | 117-39-5 | 3 | 2.04E+04 | 1.91E+04 | 1.59E+04 | 1.38E+04 | 1.63E+04 | 2.83E+04 | 2.50E+04 | 2.03E+04 | 2.57E+04 | 1.77E+04 | 2.76E+04 | 2.92E+04 | 1.54E+04 | 1.73E+04 | 2.17E+04 | C00389 | ko00941,ko00944,ko01100,ko01110 |
| Hmqn000843 | 3.01E+02 | 1.23E+02 | 3.02E+02 | C_13_H_18_O_8_ | [M-H]- | Tachioside | Phenolic acids | Phenolic acids | 109194-60-7 | 2 | 7.39E+04 | 5.77E+04 | 5.83E+04 | 1.66E+05 | 1.58E+05 | 1.39E+05 | 8.58E+04 | 8.68E+04 | 1.18E+05 | 6.86E+04 | 7.74E+04 | 5.07E+04 | 8.88E+04 | 9.45E+04 | 1.12E+05 | -- | -- |
| Zmdp007400 | 3.03E+02 | 2.71E+02 | 3.02E+02 | C_17_H_18_O_5_ | [M+H]+ | 4,5-dihydroxy-2,3,6-trimethoxy-9,10-dihydrophenanthrene | Others | Others | - | 2 | 4.49E+05 | 4.12E+05 | 4.16E+05 | 4.38E+05 | 4.51E+05 | 3.93E+05 | 3.88E+05 | 4.00E+05 | 4.22E+05 | 1.78E+05 | 1.32E+05 | 1.50E+05 | 3.75E+05 | 3.84E+05 | 3.90E+05 | -- | -- |
| Zmdp008064 | 3.03E+02 | 2.29E+02 | 3.02E+02 | C_19_H_26_O_3_ | [M+H]+ | cannabielsoin-C3 | Others | Others | - | 2 | 2.70E+04 | 2.44E+04 | 2.23E+04 | 2.50E+04 | 2.00E+04 | 2.43E+04 | 1.59E+04 | 1.56E+04 | 1.58E+04 | 1.18E+04 | 1.38E+04 | 1.33E+04 | 1.96E+04 | 1.87E+04 | 2.40E+04 | -- | -- |
| Lmbn014696 | 3.01E+02 | 3.01E+02 | 3.02E+02 | C_20_H_30_O_2_ | [M-H]- | Pimaric acid | Terpenoids | Ditepenoids | 127-27-5 | 1 | 2.91E+04 | 2.34E+04 | 2.91E+04 | 6.38E+04 | 2.07E+04 | 1.80E+04 | 5.81E+04 | 4.68E+04 | 1.77E+05 | 3.08E+04 | 2.79E+04 | 2.96E+04 | 9.13E+04 | 8.09E+04 | 8.76E+04 | C09159 | -- |
| MWSslk208 | 3.01E+02 | 3.01E+02 | 3.02E+02 | C_20_H_30_O_2_ | [M-H]- | Kaurenoic Acid | Terpenoids | Ditepenoids | 6730-83-2 | 1 | 3.15E+04 | 2.58E+04 | 3.35E+04 | 6.36E+04 | 2.05E+04 | 2.96E+04 | 5.38E+04 | 5.04E+04 | 1.77E+05 | 3.42E+04 | 2.75E+04 | 3.31E+04 | 8.84E+04 | 9.23E+04 | 8.77E+04 | C11874 | ko00904,ko01100,ko01110 |
| mws1375 | 3.04E+02 | 1.85E+02 | 3.03E+02 | C_12_H_21_N_3_O_6_ | [M+H]+ | Nicotianamine | Alkaloids | Alkaloids | 34441-14-0 | 2 | 3.06E+04 | 2.38E+04 | 2.60E+04 | 2.93E+04 | 2.65E+04 | 3.27E+04 | 1.76E+04 | 2.14E+04 | 2.07E+04 | 2.30E+04 | 2.21E+04 | 1.62E+04 | 2.52E+04 | 3.25E+04 | 2.82E+04 | C05324 | -- |
| pmp000022 | 3.05E+02 | 1.59E+02 | 3.04E+02 | C_16_H_16_O_6_ | [M+H]+ | Oxypeucedanin hydrate | Lignans and Coumarins | Coumarins | 2643-85-8 | 2 | 2.76E+07 | 2.92E+07 | 2.87E+07 | 1.36E+07 | 1.27E+07 | 1.26E+07 | 1.78E+07 | 1.82E+07 | 1.74E+07 | 1.15E+07 | 1.15E+07 | 1.15E+07 | 1.95E+07 | 1.86E+07 | 1.99E+07 | -- | -- |
| mws0369 | 3.03E+02 | 2.59E+02 | 3.04E+02 | C_20_H_32_O_2_ | [M-H]- | Arachidonic Acid | Lipids | Free fatty acids | 506-32-1 | 1 | 4.91E+03 | 4.01E+03 | 4.78E+03 | 5.40E+03 | 3.77E+03 | 6.12E+03 | 1.32E+04 | 1.04E+04 | 1.46E+04 | 1.09E+04 | 1.62E+04 | 1.18E+04 | 9.05E+03 | 8.62E+03 | 8.73E+03 | C00219 | ko00590,ko00591,ko01040,ko01100 |
| mws0049 | 3.07E+02 | 1.39E+02 | 3.06E+02 | C_15_H_14_O_7_ | [M+H]+ | Gallocatechin | Flavonoids | Flavanols | 970-73-0 | 3 | 5.93E+04 | 5.18E+04 | 7.17E+04 | 4.64E+04 | 5.78E+04 | 5.90E+04 | 5.94E+04 | 5.71E+04 | 5.10E+04 | 3.86E+04 | 5.33E+04 | 8.15E+04 | 4.91E+04 | 5.54E+04 | 5.21E+04 | C12127 | ko00941,ko01110 |
| pmb0772 | 3.07E+02 | 1.31E+02 | 3.06E+02 | C_19_H_18_N_2_O_2_ | [M+H]+ | N-Cinnamoylserotonin | Alkaloids | Plumerane | - | 3 | 4.81E+04 | 5.13E+04 | 4.79E+04 | 9.00E+00 | 9.00E+00 | 9.00E+00 | 9.00E+00 | 9.00E+00 | 9.00E+00 | 9.00E+00 | 9.00E+00 | 9.00E+00 | 1.03E+04 | 1.38E+04 | 1.70E+04 | -- | -- |
| pme1086 | 3.06E+02 | 1.43E+02 | 3.07E+02 | C_10_H_17_N_3_O_6_S | [M-H]- | Glutathione reduced form | Amino acids and derivatives | Amino acids and derivatives | 70-18-8 | 3 | 9.00E+00 | 9.00E+00 | 9.00E+00 | 2.18E+04 | 2.27E+04 | 2.66E+04 | 9.00E+00 | 9.00E+00 | 9.00E+00 | 1.63E+04 | 2.30E+04 | 1.06E+04 | 9.30E+03 | 1.03E+04 | 1.18E+04 | C00051 | ko00270,ko00480,ko01100,ko01240,ko02010 |
| Hmlp007622 | 3.08E+02 | 1.37E+02 | 3.07E+02 | C_18_H_29_NO_3_ | [M+H]+ | Decanoyl vanillylamide | Alkaloids | Phenolamine | - | 3 | 3.95E+04 | 3.38E+04 | 4.08E+04 | 3.29E+04 | 3.67E+04 | 4.30E+04 | 9.00E+00 | 9.00E+00 | 9.00E+00 | 9.00E+00 | 9.00E+00 | 9.00E+00 | 1.31E+04 | 1.76E+04 | 2.38E+04 | -- | -- |
| pmb2855 | 3.07E+02 | 1.45E+02 | 3.08E+02 | C_11_H_20_N_2_O_8_ | [M-H]- | L-Glutamine-O-glycoside | Amino acids and derivatives | Amino acids and derivatives | - | 3 | 5.98E+06 | 5.80E+06 | 5.66E+06 | 3.76E+05 | 4.10E+05 | 4.18E+05 | 4.43E+06 | 4.52E+06 | 5.16E+06 | 3.79E+05 | 4.83E+05 | 4.12E+05 | 2.69E+06 | 2.73E+06 | 2.92E+06 | -- | -- |
| pmn001610 | 3.07E+02 | 3.07E+02 | 3.08E+02 | C_20_H_36_O_2_ | [M-H]- | Eicosadienoic acid | Lipids | Free fatty acids | 5598-38-9 | 3 | 1.28E+06 | 1.18E+06 | 1.25E+06 | 3.14E+06 | 3.34E+06 | 3.31E+06 | 1.95E+06 | 2.25E+06 | 2.33E+06 | 2.53E+06 | 2.59E+06 | 2.65E+06 | 2.48E+06 | 2.32E+06 | 2.30E+06 | C16525 | ko01040 |
| pmb2857 | 3.08E+02 | 1.46E+02 | 3.09E+02 | C_11_H_19_NO_9_ | [M-H]- | L-Glutamic acid-O-glycoside | Amino acids and derivatives | Amino acids and derivatives | - | 3 | 1.40E+06 | 1.15E+06 | 1.08E+06 | 6.14E+04 | 3.32E+04 | 1.95E+04 | 7.95E+05 | 4.38E+05 | 5.44E+05 | 4.97E+04 | 2.80E+04 | 2.12E+04 | 3.98E+05 | 1.96E+05 | 3.63E+05 | -- | -- |
| Lmhn003074 | 3.09E+02 | 1.93E+02 | 3.10E+02 | C_14_H_14_O_8_ | [M-H]- | Feruloylmalic acid | Phenolic acids | Phenolic acids | - | 3 | 6.70E+04 | 7.19E+04 | 7.07E+04 | 1.13E+05 | 1.28E+05 | 1.23E+05 | 5.84E+04 | 5.03E+04 | 5.84E+04 | 8.65E+04 | 7.03E+04 | 6.37E+04 | 7.79E+04 | 8.93E+04 | 7.46E+04 | -- | -- |
| pmb2791 | 3.09E+02 | 2.09E+02 | 3.10E+02 | C_18_H_30_O_4_ | [M-H]- | 9-Hydroperoxy-10E,12,15Z-octadecatrienoic acid | Lipids | Free fatty acids | 111004-08-1 | 3 | 7.01E+04 | 2.69E+04 | 2.90E+04 | 3.90E+04 | 4.19E+04 | 6.48E+04 | 3.03E+04 | 2.90E+04 | 4.76E+04 | 5.23E+04 | 5.75E+04 | 6.28E+04 | 1.18E+05 | 1.20E+05 | 1.28E+05 | C16321 | ko00592,ko01110 |
| Zmdp001857 | 3.11E+02 | 1.65E+02 | 3.10E+02 | C_14_H_18_N_2_O_6_ | [M+H]+ | γ-Glutamyltyrosine | Amino acids and derivatives | Amino acids and derivatives | 7432-23-7 | 2 | 5.29E+05 | 4.31E+05 | 5.14E+05 | 1.66E+05 | 1.78E+05 | 1.68E+05 | 8.41E+05 | 8.57E+05 | 9.14E+05 | 4.05E+05 | 4.51E+05 | 4.41E+05 | 4.84E+05 | 4.85E+05 | 5.61E+05 | -- | -- |
| Zmzn003953 | 3.09E+02 | 2.27E+02 | 3.10E+02 | C_18_H_30_O_4_ | [M-H]- | 13(s)-hydroperoxy-(9z,11e,15z)-octadecatrienoic acid | Lipids | Free fatty acids | 67597-26-6 | 1 | 9.04E+04 | 1.10E+05 | 9.83E+04 | 1.36E+05 | 1.17E+05 | 1.07E+05 | 1.15E+05 | 1.05E+05 | 1.13E+05 | 1.14E+05 | 1.00E+05 | 1.12E+05 | 1.47E+05 | 1.42E+05 | 1.47E+05 | C04785 | ko00592,ko01100,ko01110 |
| Zmyn004449 | 3.09E+02 | 1.71E+02 | 3.10E+02 | C_18_H_30_O_4_ | [M-H]- | 9-Hydroxy-12-oxo-10(E),15(Z)-octadecadienoic acid | Lipids | Free fatty acids | - | 2 | 8.75E+04 | 9.30E+04 | 9.45E+04 | 9.26E+04 | 7.92E+04 | 9.62E+04 | 6.74E+04 | 5.69E+04 | 6.61E+04 | 4.07E+04 | 5.08E+04 | 5.03E+04 | 7.35E+04 | 7.49E+04 | 8.43E+04 | C21923 | ko00592,ko01110 |
| pme3967 | 3.12E+02 | 1.80E+02 | 3.11E+02 | C_12_H_17_N_5_O_5_ | [M+H]+ | 2-(Dimethylamino)guanosine | Nucleotides and derivatives | Nucleotides and derivatives | 2140-67-2 | 1 | 4.62E+06 | 4.86E+06 | 4.23E+06 | 7.81E+05 | 9.23E+05 | 6.76E+05 | 1.15E+06 | 1.36E+06 | 1.23E+06 | 5.68E+05 | 5.22E+05 | 5.55E+05 | 2.19E+06 | 2.04E+06 | 2.18E+06 | -- | -- |
| Lmcp002302 | 3.12E+02 | 1.80E+02 | 3.11E+02 | C_12_H_17_N_5_O_5_ | [M+H]+ | N6-(2-Hydroxyethyl)adenosine | Nucleotides and derivatives | Nucleotides and derivatives | 4338-48-1 | 1 | 4.57E+06 | 3.49E+06 | 4.43E+06 | 6.95E+05 | 8.84E+05 | 7.33E+05 | 1.18E+06 | 1.28E+06 | 1.13E+06 | 5.44E+05 | 4.78E+05 | 4.80E+05 | 2.14E+06 | 1.92E+06 | 2.22E+06 | -- | -- |
| Lmhn001477 | 3.11E+02 | 1.79E+02 | 3.12E+02 | C_13_H_12_O_9_ | [M-H]- | 2-Caffeoyl-L-tartaric acid (Caftaric acid) | Phenolic acids | Phenolic acids | 67879-58-7 | 3 | 3.75E+04 | 4.03E+04 | 4.16E+04 | 4.51E+03 | 5.70E+03 | 5.76E+03 | 7.09E+03 | 7.94E+03 | 9.37E+03 | 3.19E+03 | 4.10E+03 | 2.88E+03 | 1.35E+04 | 1.75E+04 | 1.19E+04 | -- | -- |
| Lmqp002761 | 3.13E+02 | 1.51E+02 | 3.12E+02 | C_19_H_20_O_4_ | [M+H]+ | Alnusonol | Phenolic acids | Phenolic acids | 52330-12-8 | 2 | 5.14E+04 | 7.13E+04 | 5.51E+04 | 1.56E+05 | 1.02E+05 | 5.18E+04 | 1.73E+05 | 2.13E+05 | 1.86E+05 | 2.05E+05 | 1.74E+05 | 2.72E+05 | 1.47E+05 | 1.71E+05 | 1.42E+05 | -- | -- |
| Lmbn005287 | 3.11E+02 | 2.49E+02 | 3.12E+02 | C_18_H_32_O_4_ | [M-H]- | 7S,8S-DiHODE; (9Z,12Z)-(7S,8S)-Dihydroxyoctadeca-9,12-dienoic acid | Lipids | Free fatty acids | 143288-65-7 | 2 | 7.12E+04 | 6.19E+04 | 6.44E+04 | 1.28E+04 | 1.21E+04 | 1.30E+04 | 9.02E+04 | 8.04E+04 | 1.04E+05 | 1.64E+04 | 1.72E+04 | 1.42E+04 | 5.13E+04 | 4.54E+04 | 5.54E+04 | C07354 | ko00591 |
| Rfmb087 | 3.11E+02 | 2.93E+02 | 3.12E+02 | C_18_H_32_O_4_ | [M-H]- | 9-Hydroxy-13-oxo-10-octadecenoic Acid | Lipids | Free fatty acids | - | 3 | 3.89E+05 | 4.20E+05 | 4.37E+05 | 1.33E+06 | 1.22E+06 | 1.20E+06 | 8.91E+05 | 9.25E+05 | 9.72E+05 | 5.72E+05 | 6.23E+05 | 5.78E+05 | 8.79E+05 | 9.24E+05 | 9.56E+05 | -- | -- |
| pmb2804 | 3.11E+02 | 1.71E+02 | 3.12E+02 | C_18_H_32_O_4_ | [M-H]- | 13S-Hydroperoxy-9Z,11E-octadecadienoic acid | Lipids | Free fatty acids | 33964-75-9 | 3 | 2.80E+06 | 2.90E+06 | 2.91E+06 | 2.58E+06 | 1.95E+06 | 2.09E+06 | 2.32E+06 | 2.34E+06 | 2.19E+06 | 1.08E+06 | 1.07E+06 | 1.01E+06 | 2.29E+06 | 2.45E+06 | 2.61E+06 | C04717 | ko00591,ko01100 |
| Zmjn004133 | 3.11E+02 | 2.93E+02 | 3.12E+02 | C_18_H_32_O_4_ | [M-H]- | 9S-Hydroperoxy-10E,12Z-octadecadienoic acid | Lipids | Free fatty acids | 5502-91-0 | 3 | 3.57E+05 | 4.15E+05 | 3.84E+05 | 3.34E+05 | 3.07E+05 | 3.31E+05 | 3.04E+05 | 3.20E+05 | 3.58E+05 | 1.66E+05 | 1.55E+05 | 1.64E+05 | 3.28E+05 | 3.33E+05 | 3.37E+05 | C14827 | ko00591,ko01100 |
| pmn001689 | 3.11E+02 | 2.23E+02 | 3.12E+02 | C_18_H_32_O_4_ | [M-H]- | 9-Hydroxy-12-oxo-15(Z)-octadecenoic acid | Lipids | Free fatty acids | - | 1 | 6.93E+05 | 6.49E+05 | 7.87E+05 | 1.67E+05 | 1.48E+05 | 1.41E+05 | 2.09E+05 | 2.06E+05 | 2.00E+05 | 4.60E+04 | 5.48E+04 | 4.66E+04 | 3.09E+05 | 3.33E+05 | 3.09E+05 | C21924 | ko00592,ko01110 |
| mws1433 | 3.14E+02 | 1.77E+02 | 3.13E+02 | C_18_H_19_NO_4_ | [M+H]+ | N-Feruloyltyramine | Alkaloids | Phenolamine | 66648-43-9 | 3 | 4.05E+04 | 4.62E+04 | 4.65E+04 | 4.04E+05 | 2.91E+05 | 3.78E+05 | 1.75E+05 | 1.49E+05 | 2.20E+05 | 1.95E+05 | 1.69E+05 | 1.78E+05 | 1.78E+05 | 1.88E+05 | 1.87E+05 | C02717 | -- |
| Lmmn002179 | 3.13E+02 | 1.07E+02 | 3.14E+02 | C_14_H_18_O_8_ | [M-H]- | Methyl salicylate-2-O-glucoside | Phenolic acids | Phenolic acids | 10019-60-0 | 1 | 1.33E+05 | 1.58E+05 | 1.41E+05 | 2.39E+05 | 2.14E+05 | 2.10E+05 | 2.20E+05 | 2.34E+05 | 2.23E+05 | 2.45E+05 | 2.71E+05 | 2.32E+05 | 1.89E+05 | 2.07E+05 | 2.31E+05 | -- | -- |
| Lmyn002403 | 3.13E+02 | 1.01E+02 | 3.14E+02 | C_14_H_18_O_8_ | [M-H]- | Mandelic acid-β-glucoside | Phenolic acids | Phenolic acids | - | 2 | 3.67E+04 | 3.71E+04 | 3.43E+04 | 5.12E+04 | 5.29E+04 | 5.83E+04 | 6.62E+04 | 6.56E+04 | 6.48E+04 | 7.37E+04 | 6.46E+04 | 7.34E+04 | 5.45E+04 | 5.09E+04 | 5.75E+04 | -- | -- |
| Cmzp002057 | 3.15E+02 | 2.55E+02 | 3.14E+02 | C_14_H_18_O_8_ | [M+H]+ | 6-O-Acetylarbutin | Phenolic acids | Phenolic acids | 10338-88-2 | 3 | 3.47E+04 | 4.40E+04 | 4.69E+04 | 5.59E+04 | 6.18E+04 | 5.50E+04 | 2.55E+04 | 2.76E+04 | 2.09E+04 | 3.89E+04 | 3.71E+04 | 4.79E+04 | 3.79E+04 | 3.40E+04 | 3.77E+04 | -- | -- |
| Lmbn005487 | 3.13E+02 | 2.51E+02 | 3.14E+02 | C_18_H_34_O_4_ | [M-H]- | 12,13-DHOME; (9Z)-12,13-Dihydroxyoctadec-9-enoic acid | Lipids | Free fatty acids | 263399-35-5 | 3 | 3.72E+04 | 3.17E+04 | 4.13E+04 | 6.04E+03 | 6.90E+03 | 5.43E+03 | 4.10E+04 | 4.30E+04 | 4.29E+04 | 8.82E+03 | 9.39E+03 | 9.28E+03 | 2.47E+04 | 2.73E+04 | 2.53E+04 | C14829 | ko00591 |
| Lmbn007891 | 3.13E+02 | 1.83E+02 | 3.14E+02 | C_18_H_34_O_4_ | [M-H]- | Hydroxy ricinoleic acid | Lipids | Free fatty acids | - | 1 | 5.73E+05 | 5.23E+05 | 6.87E+05 | 2.86E+05 | 2.38E+05 | 2.48E+05 | 2.13E+05 | 2.12E+05 | 2.08E+05 | 1.10E+05 | 1.17E+05 | 8.64E+04 | 2.85E+05 | 3.14E+05 | 3.50E+05 | -- | -- |
| pmp000492 | 3.16E+02 | 2.31E+02 | 3.15E+02 | C_19_H_25_NO_3_ | [M+H]+ | Piperoliene A | Alkaloids | Alkaloids | - | 2 | 6.63E+04 | 7.73E+04 | 7.49E+04 | 5.34E+04 | 7.22E+04 | 6.36E+04 | 5.95E+04 | 5.85E+04 | 5.46E+04 | 6.08E+04 | 3.92E+04 | 4.24E+04 | 5.39E+04 | 6.64E+04 | 8.38E+04 | -- | -- |
| pmb2871 | 3.15E+02 | 1.53E+02 | 3.16E+02 | C_13_H_16_O_9_ | [M-H]- | 1-O-Gentisoyl-D-glucoside | Phenolic acids | Phenolic acids | 23445-11-6 | 1 | 9.07E+06 | 8.42E+06 | 1.03E+07 | 1.12E+07 | 1.17E+07 | 1.09E+07 | 2.65E+06 | 2.61E+06 | 2.47E+06 | 2.07E+06 | 2.08E+06 | 2.21E+06 | 6.13E+06 | 6.75E+06 | 7.20E+06 | -- | -- |
| Lmfp001509 | 3.17E+02 | 1.53E+02 | 3.16E+02 | C_13_H_16_O_9_ | [M+H]+ | 1-O-Galloyl-rhamnose | Phenolic acids | Phenolic acids | - | 3 | 1.59E+04 | 1.17E+04 | 1.49E+04 | 4.03E+04 | 3.17E+04 | 4.20E+04 | 7.90E+03 | 1.21E+04 | 1.60E+04 | 2.39E+04 | 1.84E+04 | 1.98E+04 | 2.31E+04 | 2.47E+04 | 2.33E+04 | -- | -- |
| pmn001367 | 3.15E+02 | 1.53E+02 | 3.16E+02 | C_13_H_16_O_9_ | [M-H]- | Protocatechuic acid-4-O-glucoside | Phenolic acids | Phenolic acids | - | 1 | 1.18E+07 | 1.15E+07 | 1.38E+07 | 1.44E+07 | 1.50E+07 | 1.47E+07 | 4.31E+06 | 4.33E+06 | 4.12E+06 | 2.44E+06 | 2.52E+06 | 2.47E+06 | 8.19E+06 | 9.39E+06 | 9.57E+06 | -- | -- |
| MWSmce550 | 3.17E+02 | 2.31E+02 | 3.16E+02 | C_17_H_16_O_6_ | [M+H]+ | Byakangelicol | Others | Others | 26091-79-2 | 1 | 3.64E+07 | 3.58E+07 | 3.55E+07 | 3.65E+07 | 3.60E+07 | 3.52E+07 | 2.47E+07 | 2.54E+07 | 2.57E+07 | 2.64E+07 | 2.66E+07 | 2.74E+07 | 3.21E+07 | 3.20E+07 | 3.35E+07 | C16925 | -- |
| pmp000024 | 3.17E+02 | 2.33E+02 | 3.16E+02 | C_17_H_16_O_6_ | [M+H]+ | Isobyakangelicol | Lignans and Coumarins | Coumarins | 35214-81-4 | 1 | 5.69E+06 | 5.32E+06 | 4.95E+06 | 5.28E+06 | 5.52E+06 | 5.06E+06 | 3.94E+06 | 3.44E+06 | 4.31E+06 | 4.08E+06 | 3.86E+06 | 4.35E+06 | 4.23E+06 | 4.54E+06 | 4.77E+06 | C16914 | -- |
| pme3459 | 3.17E+02 | 2.99E+02 | 3.16E+02 | C_20_H_28_O_3_ | [M+H]+ | Cafestol | Terpenoids | Ditepenoids | 469-83-0 | 3 | 2.13E+05 | 2.10E+05 | 2.08E+05 | 1.93E+05 | 1.64E+05 | 1.93E+05 | 6.68E+04 | 8.47E+04 | 1.07E+05 | 9.16E+04 | 8.32E+04 | 1.01E+05 | 1.63E+05 | 1.62E+05 | 1.36E+05 | C09066 | -- |
| pmp000298 | 3.19E+02 | 1.89E+02 | 3.18E+02 | C_17_H_18_O_6_ | [M+H]+ | 30-O-Acetylhamaudol | Others | Others | - | 3 | 2.86E+05 | 1.41E+05 | 8.59E+04 | 1.89E+05 | 1.67E+05 | 1.48E+05 | 1.03E+05 | 1.32E+05 | 1.10E+05 | 1.41E+05 | 1.45E+05 | 1.12E+05 | 1.52E+05 | 1.81E+05 | 1.28E+05 | -- | -- |
| pmp000970 | 3.19E+02 | 3.01E+02 | 3.18E+02 | C_20_H_30_O_3_ | [M+H]+ | Hispanolone | Terpenoids | Ditepenoids | 18676-07-8 | 2 | 2.94E+04 | 3.16E+04 | 3.82E+04 | 4.60E+04 | 3.53E+04 | 4.47E+04 | 5.12E+04 | 6.78E+04 | 5.14E+04 | 2.25E+04 | 2.87E+04 | 2.76E+04 | 3.80E+04 | 5.85E+04 | 5.26E+04 | -- | -- |
| Hmqp005412 | 3.19E+02 | 3.19E+02 | 3.18E+02 | C_20_H_30_O_3_ | [M+H]+ | 12-Hydroxyabietic Acid | Terpenoids | Ditepenoids | 3484-61-5 | 2 | 1.40E+04 | 1.51E+04 | 1.44E+04 | 7.89E+03 | 1.14E+04 | 9.54E+03 | 1.34E+04 | 1.28E+04 | 1.11E+04 | 5.22E+03 | 9.92E+03 | 4.91E+03 | 9.58E+03 | 1.20E+04 | 1.57E+04 | -- | -- |
| pmp001267 | 3.20E+02 | 3.02E+02 | 3.19E+02 | C_21_H_37_NO | [M+H]+ | 2-(Dodecylamino)-3-phenyl-1-propanol | Others | Others | - | 2 | 3.53E+04 | 3.91E+04 | 4.26E+04 | 9.00E+00 | 9.00E+00 | 9.00E+00 | 2.46E+04 | 2.65E+04 | 2.24E+04 | 9.00E+00 | 9.00E+00 | 9.00E+00 | 2.25E+04 | 1.33E+04 | 1.46E+04 | -- | -- |
| pmb3075 | 3.19E+02 | 1.45E+02 | 3.20E+02 | C_16_H_16_O_7_ | [M-H]- | 3-O-p-Coumaroylshikimic acid | Phenolic acids | Phenolic acids | - | 3 | 9.00E+00 | 9.00E+00 | 9.00E+00 | 9.00E+00 | 9.00E+00 | 9.00E+00 | 5.07E+04 | 5.15E+04 | 4.75E+04 | 9.00E+00 | 9.00E+00 | 9.00E+00 | 1.04E+04 | 2.09E+04 | 2.11E+04 | -- | -- |
| pmb0751 | 3.21E+02 | 1.47E+02 | 3.20E+02 | C_16_H_16_O_7_ | [M+H]+ | Trans-5-O-(p-Coumaroyl)shikimate | Phenolic acids | Phenolic acids | - | 3 | 4.64E+04 | 4.46E+04 | 4.99E+04 | 3.79E+04 | 4.80E+04 | 3.71E+04 | 6.08E+05 | 6.39E+05 | 6.54E+05 | 2.98E+04 | 4.46E+04 | 5.30E+04 | 1.86E+05 | 1.72E+05 | 2.09E+05 | C02947 | ko00940,ko00941,ko00945,ko01100,ko01110 |
| MWS3083 | 3.19E+02 | 5.90E+01 | 3.20E+02 | C_20_H_32_O_3_ | [M-H]- | 15(S)-HETE; 15(S)-Hydroxy-5Z,8Z,11Z,13E-eicosatetraenoic acid | Lipids | Free fatty acids | 54845-95-3 | 2 | 4.59E+05 | 4.98E+05 | 4.95E+05 | 1.37E+06 | 1.22E+06 | 1.45E+06 | 9.26E+05 | 7.94E+05 | 8.64E+05 | 1.02E+06 | 1.17E+06 | 1.31E+06 | 7.89E+05 | 1.07E+06 | 9.93E+05 | C04742 | ko00590 |
| mws0582 | 3.22E+02 | 1.30E+02 | 3.21E+02 | C_11_H_19_N_3_O_6_S | [M+H]+ | S-(Methyl)glutathione | Amino acids and derivatives | Amino acids and derivatives | 2922-56-7 | 2 | 6.08E+05 | 4.26E+05 | 6.68E+05 | 1.22E+05 | 1.00E+05 | 1.09E+05 | 5.78E+05 | 9.21E+05 | 6.20E+05 | 4.00E+05 | 2.40E+05 | 2.73E+05 | 3.64E+05 | 3.82E+05 | 5.47E+05 | C11347 | -- |
| Hmlp007087 | 3.22E+02 | 1.53E+02 | 3.21E+02 | C_18_H_27_NO_4_ | [M+H]+ | 17-Hydroxycapsaicin | Alkaloids | Phenolamine | 69173-71-3 | 2 | 5.88E+04 | 8.51E+04 | 8.07E+04 | 1.90E+05 | 1.77E+05 | 1.40E+05 | 4.85E+04 | 4.85E+04 | 4.76E+04 | 8.42E+04 | 6.11E+04 | 1.14E+05 | 9.57E+04 | 9.99E+04 | 9.71E+04 | -- | -- |
| pme3174 | 3.24E+02 | 1.12E+02 | 3.23E+02 | C_9_H_14_N_3_O_8_P | [M+H]+ | Cytidine 5'-monophosphate(Cytidylic acid) | Nucleotides and derivatives | Nucleotides and derivatives | 63-37-6 | 3 | 1.61E+05 | 1.39E+05 | 1.74E+05 | 2.40E+04 | 1.58E+04 | 2.84E+04 | 1.35E+05 | 1.43E+05 | 1.24E+05 | 3.30E+04 | 2.32E+04 | 2.75E+04 | 9.41E+04 | 4.59E+04 | 9.22E+04 | C00055 | ko00240,ko01100 |
| pme2074 | 3.22E+02 | 1.30E+02 | 3.23E+02 | C_18_H_29_NO_4_ | [M-H]- | Jasmonoyl-L-Isoleucine | Amino acids and derivatives | Amino acids and derivatives | 120330-93-0 | 3 | 9.00E+00 | 9.00E+00 | 9.00E+00 | 4.54E+04 | 4.91E+04 | 4.91E+04 | 1.68E+04 | 1.41E+04 | 1.58E+04 | 1.57E+04 | 1.38E+04 | 1.43E+04 | 2.11E+04 | 1.92E+04 | 2.38E+04 | C18699 | ko04075 |
| pme3188 | 3.23E+02 | 2.11E+02 | 3.24E+02 | C_9_H_13_N_2_O_9_P | [M-H]- | Uridine 5'-monophosphate | Nucleotides and derivatives | Nucleotides and derivatives | 58-97-9 | 2 | 1.51E+06 | 1.36E+06 | 1.50E+06 | 3.10E+05 | 3.09E+05 | 2.95E+05 | 3.16E+06 | 3.12E+06 | 3.44E+06 | 4.86E+05 | 5.42E+05 | 5.10E+05 | 1.27E+06 | 1.49E+06 | 1.51E+06 | C00105 | ko00240,ko01100,ko01240 |
| YC512112 | 3.25E+02 | 1.63E+02 | 3.24E+02 | C_16_H_24_O_5_N_2_ | [M+H]+ | Anabasine-glucoside | Alkaloids | Pyridine alkaloids | - | 3 | 6.52E+03 | 7.22E+03 | 7.69E+03 | 5.48E+03 | 6.84E+03 | 1.27E+04 | 8.28E+03 | 7.22E+03 | 4.98E+03 | 1.90E+04 | 3.77E+03 | 1.34E+04 | 1.02E+04 | 3.77E+03 | 1.11E+04 | -- | -- |
| mws0393 | 3.25E+02 | 1.60E+02 | 3.24E+02 | C_20_H_24_O_2_N_2_ | [M+H]+ | Quinine | Alkaloids | Quinoline alkaloids | 130-95-0 | 3 | 6.73E+04 | 4.08E+04 | 7.26E+04 | 1.43E+05 | 1.09E+05 | 9.99E+04 | 7.37E+04 | 5.60E+04 | 9.87E+04 | 8.77E+04 | 7.63E+04 | 9.96E+04 | 9.75E+04 | 7.99E+04 | 8.23E+04 | C06526 | -- |
| Hmqp006023 | 3.26E+02 | 3.09E+02 | 3.24E+02 | C_20_H_36_O_3_ | [M+H]+ | Ethyl 9-Hydroxy-10,12-octadecadienoic acid | Lipids | Free fatty acids | - | 2 | 4.09E+04 | 4.10E+04 | 4.09E+04 | 1.29E+05 | 1.27E+05 | 1.27E+05 | 1.32E+05 | 1.23E+05 | 1.25E+05 | 2.06E+05 | 2.17E+05 | 2.10E+05 | 1.07E+05 | 1.09E+05 | 1.13E+05 | -- | -- |
| mws0983 | 3.26E+02 | 6.21E+01 | 3.25E+02 | C_20_H_39_NO_2_ | [M+H]+ | N-Oleoylethanolamine | Alkaloids | Alkaloids | 111-58-0 | 1 | 3.57E+05 | 3.39E+05 | 3.62E+05 | 1.06E+06 | 1.05E+06 | 1.05E+06 | 1.03E+06 | 1.04E+06 | 1.05E+06 | 1.69E+06 | 1.80E+06 | 1.74E+06 | 9.08E+05 | 8.82E+05 | 9.22E+05 | C20792 | -- |
| Zmhn002301 | 3.25E+02 | 1.63E+02 | 3.26E+02 | C_15_H_18_O_8_ | [M-H]- | p-Coumaric acid-4-O-glucoside* | Phenolic acids | Phenolic acids | 117405-48-8 | 3 | 2.03E+05 | 2.22E+05 | 2.05E+05 | 1.55E+05 | 1.66E+05 | 2.04E+05 | 1.94E+05 | 1.64E+05 | 1.83E+05 | 2.01E+05 | 2.40E+05 | 2.23E+05 | 1.69E+05 | 2.17E+05 | 2.38E+05 | C06739 | -- |
| pmn001419 | 3.25E+02 | 1.63E+02 | 3.26E+02 | C_15_H_18_O_8_ | [M-H]- | 1-O-[(E)-p-Cumaroyl]-D-glucose* | Phenolic acids | Phenolic acids | - | 3 | 3.87E+05 | 3.82E+05 | 4.14E+05 | 3.27E+05 | 3.76E+05 | 3.69E+05 | 3.51E+05 | 3.56E+05 | 4.23E+05 | 4.58E+05 | 4.09E+05 | 4.43E+05 | 3.88E+05 | 4.52E+05 | 4.11E+05 | -- | -- |
| Lmqn000351 | 3.25E+02 | 5.90E+01 | 3.26E+02 | C_12_H_22_O_10_ | [M-H]- | Rutinose | Others | Saccharides and Alcohols | 90-74-4 | 2 | 5.60E+04 | 4.79E+04 | 4.59E+04 | 1.10E+05 | 1.12E+05 | 1.07E+05 | 8.19E+04 | 7.70E+04 | 7.96E+04 | 6.35E+04 | 6.78E+04 | 7.21E+04 | 7.11E+04 | 7.96E+04 | 8.27E+04 | C08247 | -- |
| Lmsn000363 | 3.25E+02 | 1.45E+02 | 3.26E+02 | C_16_H_22_O_7_ | [M-H]- | Raspberryketone glucoside | Phenolic acids | Phenolic acids | 38963-94-9 | 2 | 8.58E+06 | 7.52E+06 | 8.86E+06 | 6.47E+06 | 6.47E+06 | 7.62E+06 | 1.10E+07 | 1.01E+07 | 8.88E+06 | 6.67E+06 | 6.49E+06 | 7.07E+06 | 8.95E+06 | 8.75E+06 | 8.31E+06 | -- | -- |
| Smcp000882 | 3.28E+02 | 3.10E+02 | 3.27E+02 | C_15_H_21_NO_7_ | [M+H]+ | N-benzoyl-2-aminoethyl-β-D-glucopyranoside | Others | Others | - | 1 | 3.08E+06 | 2.79E+06 | 3.19E+06 | 3.85E+05 | 3.59E+05 | 3.47E+05 | 2.31E+06 | 2.36E+06 | 2.33E+06 | 2.61E+05 | 2.99E+05 | 2.90E+05 | 1.69E+06 | 1.60E+06 | 1.80E+06 | -- | -- |
| Lmtn002233 | 3.27E+02 | 1.65E+02 | 3.28E+02 | C_15_H_20_O_8_ | [M-H]- | Androsin | Phenolic acids | Phenolic acids | 531-28-2 | 2 | 2.86E+04 | 3.60E+04 | 2.82E+04 | 1.31E+04 | 1.32E+04 | 1.76E+04 | 6.89E+04 | 6.50E+04 | 5.41E+04 | 9.00E+00 | 9.00E+00 | 9.00E+00 | 2.87E+04 | 2.63E+04 | 3.95E+04 | -- | -- |
| MWSmce267 | 3.29E+02 | 1.87E+02 | 3.28E+02 | C_19_H_20_O_5_ | [M+H]+ | Columbianadin | Lignans and Coumarins | Coumarins | 5058-13-9 | 3 | 6.90E+03 | 4.83E+03 | 6.56E+03 | 4.40E+03 | 6.62E+03 | 8.18E+03 | 1.44E+04 | 1.32E+04 | 1.35E+04 | 1.58E+04 | 1.40E+04 | 1.52E+04 | 1.28E+04 | 1.01E+04 | 9.25E+03 | -- | -- |
| MWSslk022 | 3.29E+02 | 2.29E+02 | 3.28E+02 | C_19_H_20_O_5_ | [M+H]+ | Decursinol angelate | Others | Others | 130848-06-5 | 2 | 1.95E+06 | 2.16E+06 | 1.85E+06 | 1.62E+06 | 1.71E+06 | 1.60E+06 | 6.98E+05 | 7.41E+05 | 7.68E+05 | 8.00E+05 | 7.66E+05 | 7.87E+05 | 1.37E+06 | 1.34E+06 | 1.42E+06 | -- | -- |
| MWSmce296 | 3.29E+02 | 2.29E+02 | 3.28E+02 | C_19_H_20_O_5_ | [M+H]+ | Decursin | Lignans and Coumarins | Coumarins | 5928-25-6 | 2 | 1.81E+06 | 2.04E+06 | 1.94E+06 | 1.68E+06 | 1.60E+06 | 1.58E+06 | 6.75E+05 | 6.93E+05 | 6.93E+05 | 7.44E+05 | 7.56E+05 | 7.97E+05 | 1.33E+06 | 1.36E+06 | 1.38E+06 | C09258 | -- |
| pmp000300 | 3.29E+02 | 2.29E+02 | 3.28E+02 | C_19_H_20_O_5_ | [M+H]+ | Deltoin | Lignans and Coumarins | Coumarins | 19662-71-6 | 2 | 2.13E+06 | 2.09E+06 | 1.90E+06 | 1.77E+06 | 1.66E+06 | 1.67E+06 | 7.10E+05 | 7.91E+05 | 7.07E+05 | 7.62E+05 | 8.46E+05 | 7.89E+05 | 1.34E+06 | 1.45E+06 | 1.46E+06 | C17482 | -- |
| pmn001691 | 3.27E+02 | 2.91E+02 | 3.28E+02 | C_18_H_32_O_5_ | [M-H]- | 9,12,13-Trihydroxy-10,15-octadecadienoic acid | Lipids | Free fatty acids | - | 2 | 4.14E+05 | 3.96E+05 | 3.93E+05 | 9.72E+05 | 9.92E+05 | 1.07E+06 | 5.95E+05 | 6.38E+05 | 6.36E+05 | 4.00E+05 | 5.32E+05 | 4.41E+05 | 6.16E+05 | 6.59E+05 | 6.94E+05 | -- | -- |
| mws0884 | 3.28E+02 | 1.34E+02 | 3.29E+02 | C_10_H_12_N_5_O_6_P | [M-H]- | Cyclic 3',5'-Adenylic acid | Nucleotides and derivatives | Nucleotides and derivatives | 60-92-4 | 1 | 7.08E+05 | 7.44E+05 | 7.32E+05 | 2.08E+05 | 2.36E+05 | 2.18E+05 | 2.75E+05 | 3.11E+05 | 2.53E+05 | 1.96E+05 | 1.92E+05 | 1.74E+05 | 3.38E+05 | 3.71E+05 | 3.97E+05 | C00575 | ko00230,ko01100 |
| pmp000004 | 3.31E+02 | 3.16E+02 | 3.30E+02 | C_17_H_14_O_7_ | [M+H]+ | 4',5,7-Trihydroxy-3',6-dimethoxyflavone (Jaceosidin) | Flavonoids | Flavones | 18085-97-7 | 3 | 2.08E+04 | 2.65E+04 | 2.60E+04 | 2.09E+04 | 2.01E+04 | 1.91E+04 | 2.99E+03 | 4.32E+03 | 4.65E+03 | 4.89E+03 | 2.89E+03 | 2.95E+03 | 1.71E+04 | 1.62E+04 | 1.40E+04 | -- | -- |
| Lmtn002565 | 3.29E+02 | 1.67E+02 | 3.30E+02 | C_14_H_18_O_9_ | [M-H]- | 1-O-Vanilloyl-D-Glucose | Phenolic acids | Phenolic acids | - | 3 | 9.00E+00 | 9.00E+00 | 9.00E+00 | 3.90E+04 | 3.55E+04 | 4.07E+04 | 9.00E+00 | 9.00E+00 | 9.00E+00 | 1.82E+04 | 2.25E+04 | 2.07E+04 | 1.94E+04 | 2.20E+04 | 2.98E+04 | C20470 | -- |
| Zmhn001883 | 3.29E+02 | 2.09E+02 | 3.30E+02 | C_14_H_18_O_9_ | [M-H]- | Vanillic acid-4-O-glucoside | Phenolic acids | Phenolic acids | 32142-31-7 | 2 | 6.71E+04 | 7.83E+04 | 7.37E+04 | 2.69E+05 | 2.62E+05 | 2.89E+05 | 9.30E+04 | 8.63E+04 | 9.93E+04 | 1.06E+05 | 9.89E+04 | 1.11E+05 | 1.30E+05 | 1.30E+05 | 1.51E+05 | -- | -- |
| pmn001517 | 3.29E+02 | 1.67E+02 | 3.30E+02 | C_15_H_22_O_8_ | [M-H]- | 3,4,5-Trimethoxyphenyl-1-O-Glucoside | Phenolic acids | Phenolic acids | - | 2 | 1.62E+06 | 1.69E+06 | 1.75E+06 | 2.43E+06 | 2.56E+06 | 2.63E+06 | 2.59E+06 | 2.64E+06 | 3.10E+06 | 3.31E+06 | 3.40E+06 | 3.23E+06 | 2.46E+06 | 2.23E+06 | 2.70E+06 | -- | -- |
| pmp000663 | 3.31E+02 | 9.71E+01 | 3.30E+02 | C_21_H_30_O_3_ | [M+H]+ | Tussilagonone | Terpenoids | Sesquiterpenoids | 110042-38-1 | 3 | 6.02E+03 | 5.19E+03 | 7.74E+03 | 3.13E+04 | 2.54E+04 | 3.04E+04 | 1.23E+04 | 1.25E+04 | 1.05E+04 | 2.06E+04 | 2.45E+04 | 2.22E+04 | 1.89E+04 | 1.89E+04 | 1.67E+04 | -- | -- |
| Hmqn003054 | 3.29E+02 | 3.11E+02 | 3.30E+02 | C_18_H_34_O_5_ | [M-H]- | 9,10,11-Trihydroxy-12-octadecenoic acid | Lipids | Free fatty acids | 61911-67-9 | 1 | 8.61E+05 | 7.86E+05 | 8.29E+05 | 1.56E+06 | 1.53E+06 | 1.55E+06 | 1.29E+06 | 1.33E+06 | 1.35E+06 | 1.03E+06 | 1.28E+06 | 1.03E+06 | 1.16E+06 | 1.27E+06 | 1.29E+06 | -- | -- |
| pmn001694 | 3.29E+02 | 2.29E+02 | 3.30E+02 | C_18_H_34_O_5_ | [M-H]- | 9,10,13-Trihydroxy-11-Octadecenoic Acid | Lipids | Free fatty acids | 29907-57-1 | 2 | 1.29E+07 | 1.14E+07 | 1.25E+07 | 2.63E+07 | 2.55E+07 | 2.49E+07 | 2.19E+07 | 2.17E+07 | 2.32E+07 | 1.73E+07 | 2.17E+07 | 1.82E+07 | 1.91E+07 | 2.11E+07 | 2.09E+07 | C14835 | ko00591 |
| Lmbn004240 | 3.29E+02 | 1.99E+02 | 3.30E+02 | C_18_H_34_O_5_ | [M-H]- | 9,10-Dihydroxy-12,13-epoxyoctadecanoic acid | Lipids | Free fatty acids | - | 1 | 1.42E+04 | 1.46E+04 | 1.53E+04 | 4.10E+04 | 4.21E+04 | 4.54E+04 | 2.07E+04 | 2.15E+04 | 2.35E+04 | 1.59E+04 | 2.50E+04 | 1.57E+04 | 2.50E+04 | 2.69E+04 | 2.81E+04 | C14837 | ko00591 |
| pmp001284 | 3.31E+02 | 3.13E+02 | 3.30E+02 | C_19_H_38_O_4_ | [M+H]+ | Monopalmitin | Lipids | Glycerol ester | 542-44-9 | 1 | 2.14E+04 | 2.67E+04 | 2.50E+04 | 3.54E+04 | 3.61E+04 | 3.84E+04 | 3.03E+04 | 3.09E+04 | 3.19E+04 | 3.64E+04 | 3.65E+04 | 3.20E+04 | 2.96E+04 | 3.48E+04 | 3.39E+04 | -- | -- |
| pme3184 | 3.32E+02 | 1.36E+02 | 3.31E+02 | C_10_H_14_N_5_O_6_P | [M+H]+ | 2'-Deoxyadenosine-5'-monophosphate | Nucleotides and derivatives | Nucleotides and derivatives | 653-63-4 | 2 | 4.12E+04 | 3.67E+04 | 3.39E+04 | 5.95E+04 | 8.68E+04 | 8.40E+04 | 2.34E+04 | 2.07E+04 | 2.63E+04 | 3.09E+04 | 2.26E+04 | 3.08E+04 | 3.82E+04 | 3.37E+04 | 5.32E+04 | C00360 | ko00230,ko01100 |
| pmb0789 | 3.32E+02 | 1.52E+02 | 3.31E+02 | C_14_H_21_NO_8_ | [M+H]+ | Pyridoxine-5'-O-glucoside | Others | Vitamin | - | 3 | 2.52E+05 | 2.01E+05 | 2.06E+05 | 3.74E+05 | 4.31E+05 | 3.76E+05 | 1.19E+05 | 1.05E+05 | 1.13E+05 | 1.28E+05 | 1.35E+05 | 1.49E+05 | 2.01E+05 | 1.86E+05 | 2.29E+05 | C03996 | -- |
| pmc0066 | 3.33E+02 | 1.37E+02 | 3.32E+02 | C_10_H_13_N_4_O_7_P | [M+H]+ | 2'-Deoxyinosine-5'-monophosphate | Nucleotides and derivatives | Nucleotides and derivatives | 3393-18-8 | 3 | 2.64E+06 | 3.60E+06 | 2.89E+06 | 2.14E+06 | 2.21E+06 | 2.16E+06 | 2.18E+06 | 2.29E+06 | 2.13E+06 | 1.43E+06 | 1.38E+06 | 1.28E+06 | 1.77E+06 | 2.05E+06 | 1.83E+06 | C06196 | ko00230 |
| pmn001518 | 3.31E+02 | 1.69E+02 | 3.32E+02 | C_13_H_16_O_10_ | [M-H]- | 1-O-Galloyl-D-glucose | Phenolic acids | Phenolic acids | 58511-73-2 | 3 | 1.20E+05 | 1.03E+05 | 1.12E+05 | 1.79E+05 | 2.02E+05 | 1.85E+05 | 4.47E+04 | 4.14E+04 | 5.11E+04 | 4.66E+04 | 5.29E+04 | 5.13E+04 | 9.72E+04 | 9.66E+04 | 9.88E+04 | C01158 | -- |
| Lmyn007622 | 3.31E+02 | 1.57E+02 | 3.32E+02 | C_18_H_36_O_5_ | [M-H]- | 9,10,18-Trihydroxystearic acid | Lipids | Free fatty acids | 496-86-6 | 1 | 1.45E+04 | 1.32E+04 | 1.32E+04 | 1.48E+04 | 1.91E+04 | 1.84E+04 | 1.88E+04 | 1.87E+04 | 1.70E+04 | 1.18E+04 | 1.34E+04 | 1.27E+04 | 1.51E+04 | 1.75E+04 | 1.42E+04 | C19621 | ko00073 |
| mws0675 | 3.35E+02 | 1.23E+02 | 3.34E+02 | C_11_H_15_N_2_O_8_P | [M+H]+ | β-Nicotinamide mononucleotide | Nucleotides and derivatives | Nucleotides and derivatives | 1094-61-7 | 3 | 3.56E+03 | 1.43E+04 | 1.34E+04 | 5.05E+04 | 4.92E+04 | 4.69E+04 | 2.28E+04 | 2.30E+04 | 2.15E+04 | 5.95E+04 | 4.63E+04 | 2.31E+04 | 1.69E+04 | 2.72E+04 | 3.33E+04 | C00455 | ko00760,ko01100 |
| pmp000302 | 3.35E+02 | 2.33E+02 | 3.34E+02 | C_17_H_18_O_7_ | [M+H]+ | Isodivaricatol | Others | Others | - | 2 | 3.29E+05 | 3.37E+05 | 3.12E+05 | 2.11E+05 | 2.25E+05 | 2.27E+05 | 2.09E+05 | 2.09E+05 | 2.02E+05 | 1.04E+05 | 1.14E+05 | 1.30E+05 | 2.44E+05 | 2.28E+05 | 2.42E+05 | -- | -- |
| pmp000301 | 3.35E+02 | 2.33E+02 | 3.34E+02 | C_17_H_18_O_7_ | [M+H]+ | Divaricatol | Others | Others | - | 2 | 4.19E+05 | 4.33E+05 | 4.64E+05 | 3.31E+05 | 3.03E+05 | 3.15E+05 | 3.11E+05 | 2.99E+05 | 2.79E+05 | 1.57E+05 | 1.62E+05 | 1.77E+05 | 3.07E+05 | 3.38E+05 | 3.09E+05 | -- | -- |
| pmp000025 | 3.35E+02 | 2.31E+02 | 3.34E+02 | C_17_H_18_O_7_ | [M+H]+ | Byakangelicin | Lignans and Coumarins | Coumarins | 19573-01-4 | 1 | 1.71E+07 | 1.68E+07 | 1.65E+07 | 8.52E+06 | 8.96E+06 | 8.32E+06 | 1.09E+07 | 9.14E+06 | 9.20E+06 | 7.58E+06 | 7.36E+06 | 6.63E+06 | 1.08E+07 | 1.09E+07 | 1.23E+07 | C09141 | -- |
| MWSmce268 | 3.33E+02 | 3.15E+02 | 3.34E+02 | C_20_H_30_O_4_ | [M-H]- | Lathyrol | Terpenoids | Ditepenoids | 34420-19-4 | 2 | 5.65E+03 | 5.02E+03 | 5.67E+03 | 1.23E+04 | 1.02E+04 | 8.03E+03 | 1.09E+04 | 9.74E+03 | 9.63E+03 | 6.54E+03 | 4.82E+03 | 6.43E+03 | 8.74E+03 | 9.58E+03 | 1.01E+04 | C09125 | -- |
| mws0932 | 3.37E+02 | 2.83E+02 | 3.36E+02 | C_20_H_32_O_4_ | [M+H]+ | 15-Hydroperoxyicosatetraenoic acid | Lipids | Free fatty acids | 70981-96-3 | 3 | 1.82E+04 | 1.63E+04 | 2.46E+04 | 9.00E+00 | 9.00E+00 | 9.00E+00 | 4.65E+03 | 5.81E+03 | 4.46E+03 | 9.00E+00 | 9.00E+00 | 9.00E+00 | 7.11E+03 | 7.41E+03 | 1.06E+04 | C05966 | ko00590,ko01100 |
| pma6460 | 3.37E+02 | 1.63E+02 | 3.38E+02 | C_16_H_18_O_8_ | [M-H]- | 4-O-p-Coumaroylquinic acid | Phenolic acids | Phenolic acids | 32451-86-8 | 3 | 1.47E+05 | 1.33E+05 | 1.44E+05 | 7.67E+04 | 8.31E+04 | 8.10E+04 | 2.91E+05 | 3.62E+05 | 3.22E+05 | 1.18E+05 | 1.29E+05 | 1.39E+05 | 1.49E+05 | 1.79E+05 | 2.03E+05 | C10441 | -- |
| pmb3074 | 3.37E+02 | 1.91E+02 | 3.38E+02 | C_16_H_18_O_8_ | [M-H]- | 5-O-p-Coumaroylquinic acid | Phenolic acids | Phenolic acids | 1899-30-5 | 2 | 1.29E+07 | 1.10E+07 | 1.28E+07 | 6.75E+06 | 5.81E+06 | 6.55E+06 | 3.65E+07 | 3.75E+07 | 3.20E+07 | 8.86E+06 | 8.32E+06 | 8.97E+06 | 1.49E+07 | 1.87E+07 | 1.63E+07 | C12208 | ko00940,ko00941,ko00945,ko01110 |
| pmb3068 | 3.37E+02 | 1.73E+02 | 3.38E+02 | C_16_H_18_O_8_ | [M-H]- | 1-O-p-Coumaroylquinic acid | Phenolic acids | Phenolic acids | - | 1 | 7.10E+04 | 6.83E+04 | 1.04E+05 | 3.36E+04 | 3.57E+04 | 2.73E+04 | 6.82E+04 | 5.95E+04 | 6.68E+04 | 9.38E+04 | 8.83E+04 | 8.33E+04 | 5.40E+04 | 7.09E+04 | 5.55E+04 | -- | -- |
| pmn001421 | 3.37E+02 | 1.91E+02 | 3.38E+02 | C_16_H_18_O_8_ | [M-H]- | 3-O-p-Coumaroylquinic acid | Phenolic acids | Phenolic acids | 87099-71-6 | 1 | 4.31E+06 | 3.81E+06 | 4.14E+06 | 2.32E+06 | 2.26E+06 | 2.35E+06 | 1.34E+07 | 1.36E+07 | 1.32E+07 | 2.84E+06 | 3.54E+06 | 2.84E+06 | 5.06E+06 | 5.51E+06 | 5.88E+06 | -- | -- |
| MWSmce310 | 3.39E+02 | 2.03E+02 | 3.38E+02 | C_21_H_22_O_4_ | [M+H]+ | Bergamottin | Lignans and Coumarins | Coumarins | 7380-40-7 | 1 | 1.14E+07 | 1.07E+07 | 1.15E+07 | 1.10E+07 | 1.06E+07 | 1.04E+07 | 6.10E+06 | 6.46E+06 | 6.67E+06 | 8.43E+06 | 8.76E+06 | 8.79E+06 | 9.45E+06 | 9.35E+06 | 9.92E+06 | C22152 | -- |
| pmp000026 | 3.39E+02 | 1.37E+02 | 3.38E+02 | C_21_H_22_O_4_ | [M+H]+ | 8-Geranoxypsoralen | Lignans and Coumarins | Coumarins | 71612-25-4 | 1 | 3.88E+07 | 3.67E+07 | 3.70E+07 | 3.87E+07 | 3.77E+07 | 3.47E+07 | 2.85E+07 | 2.97E+07 | 3.15E+07 | 3.14E+07 | 3.31E+07 | 3.41E+07 | 3.39E+07 | 3.48E+07 | 3.61E+07 | -- | -- |
| MWSmce544 | 3.39E+02 | 2.03E+02 | 3.38E+02 | C_21_H_22_O_4_ | [M+H]+ | 8-Geranyloxypsoralen | Lignans and Coumarins | Coumarins | 7437-55-0 | 1 | 3.02E+07 | 2.85E+07 | 2.82E+07 | 2.78E+07 | 2.89E+07 | 2.82E+07 | 2.05E+07 | 2.15E+07 | 2.15E+07 | 2.47E+07 | 2.44E+07 | 2.55E+07 | 2.60E+07 | 2.47E+07 | 2.69E+07 | -- | -- |
| Zmyn000083 | 3.39E+02 | 7.90E+01 | 3.40E+02 | C_6_H_14_O_12_P_2_ | [M-H]- | D-Glucose 1,6-bisphosphate | Others | Saccharides and Alcohols | 10139-18-1 | 3 | 2.61E+04 | 2.52E+04 | 2.92E+04 | 2.91E+04 | 2.89E+04 | 2.44E+04 | 4.82E+04 | 5.59E+04 | 5.94E+04 | 8.29E+04 | 8.97E+04 | 8.05E+04 | 4.51E+04 | 4.40E+04 | 5.32E+04 | C01231 | ko00500,ko01100 |
| pme3311 | 3.39E+02 | 9.70E+01 | 3.40E+02 | C_6_H_14_O_12_P_2_ | [M-H]- | D-Fructose-1,6-biphosphate | Others | Saccharides and Alcohols | 488-69-7 | 3 | 2.46E+04 | 2.10E+04 | 2.48E+04 | 3.64E+04 | 4.19E+04 | 3.58E+04 | 3.18E+04 | 3.03E+04 | 2.80E+04 | 7.74E+04 | 7.41E+04 | 7.60E+04 | 4.43E+04 | 3.93E+04 | 4.21E+04 | C00354 | ko00400,ko00710,ko01100,ko01110,ko01200 |
| Cmyn001328 | 3.39E+02 | 1.77E+02 | 3.40E+02 | C_15_H_16_O_9_ | [M-H]- | Daphnin | Lignans and Coumarins | Coumarins | 486-55-5 | 1 | 5.53E+05 | 6.35E+05 | 9.88E+05 | 1.90E+06 | 1.84E+06 | 2.02E+06 | 2.41E+06 | 1.97E+06 | 2.25E+06 | 1.90E+06 | 2.12E+06 | 2.03E+06 | 1.69E+06 | 1.83E+06 | 1.82E+06 | C01421 | -- |
| Zmpn002553 | 3.39E+02 | 1.77E+02 | 3.40E+02 | C_15_H_16_O_9_ | [M-H]- | Cichoriin | Phenolic acids | Phenolic acids | 531-58-8 | 2 | 5.63E+05 | 6.16E+05 | 1.17E+06 | 2.01E+06 | 1.98E+06 | 1.97E+06 | 2.43E+06 | 1.96E+06 | 2.51E+06 | 2.19E+06 | 1.98E+06 | 2.29E+06 | 1.65E+06 | 2.09E+06 | 1.97E+06 | C09206 | -- |
| mws1015 | 3.39E+02 | 1.77E+02 | 3.40E+02 | C_15_H_16_O_9_ | [M-H]- | Esculin (6,7-DihydroxyCoumarin-6-glucoside) | Lignans and Coumarins | Coumarins | 531-75-9 | 1 | 5.72E+05 | 6.53E+05 | 1.16E+06 | 2.16E+06 | 2.02E+06 | 1.93E+06 | 2.51E+06 | 2.03E+06 | 2.53E+06 | 1.97E+06 | 2.14E+06 | 2.07E+06 | 1.71E+06 | 2.01E+06 | 1.91E+06 | C09264 | -- |
| Lmbn001162 | 3.39E+02 | 1.77E+02 | 3.40E+02 | C_15_H_16_O_9_ | [M-H]- | Esculetin-7-O-glucoside | Lignans and Coumarins | Coumarins | - | 1 | 5.73E+05 | 6.21E+05 | 9.61E+05 | 1.87E+06 | 1.87E+06 | 1.78E+06 | 2.09E+06 | 2.08E+06 | 2.24E+06 | 1.91E+06 | 1.81E+06 | 2.18E+06 | 1.64E+06 | 1.83E+06 | 1.79E+06 | -- | -- |
| Hmcn004128 | 3.39E+02 | 1.59E+02 | 3.40E+02 | C_16_H_20_O_8_ | [M-H]- | P-Methoyxcinnamate glucoside | Phenolic acids | Phenolic acids | - | 1 | 3.82E+05 | 3.83E+05 | 3.66E+05 | 5.74E+05 | 6.11E+05 | 6.65E+05 | 4.65E+05 | 4.96E+05 | 4.24E+05 | 5.48E+05 | 5.46E+05 | 5.72E+05 | 4.94E+05 | 5.65E+05 | 5.53E+05 | -- | -- |
| Xmgp006913 | 3.41E+02 | 1.37E+02 | 3.40E+02 | C_20_H_20_O_5_ | [M+H]+ | 2,4,2',4'-tetrahydroxy-3'-prenylchalcone | Flavonoids | Chalcones | - | 3 | 6.08E+04 | 5.01E+04 | 5.66E+04 | 1.19E+05 | 9.63E+04 | 8.90E+04 | 2.11E+05 | 1.96E+05 | 2.60E+05 | 1.02E+05 | 1.24E+05 | 9.88E+04 | 1.18E+05 | 1.00E+05 | 1.17E+05 | -- | -- |
| Hmmp002150 | 3.42E+02 | 1.77E+02 | 3.41E+02 | C_19_H_19_NO_5_ | [M+H]+ | 3,5-Bis(3-methoxy-4-hydroxyphenyl)-2,3-dihydro-2(1H)-pyridinone | Alkaloids | Pyridine alkaloids | - | 2 | 6.48E+04 | 7.83E+04 | 6.53E+04 | 9.00E+00 | 9.00E+00 | 9.00E+00 | 9.00E+00 | 9.00E+00 | 9.00E+00 | 9.00E+00 | 9.00E+00 | 9.00E+00 | 2.68E+04 | 1.83E+04 | 2.22E+04 | -- | -- |
| pmn001420 | 3.41E+02 | 1.79E+02 | 3.42E+02 | C_15_H_18_O_9_ | [M-H]- | 1-O-[(E)-Caffeoyl]-D-glucose* | Phenolic acids | Phenolic acids | - | 1 | 3.51E+05 | 3.92E+05 | 3.57E+05 | 2.47E+05 | 3.11E+05 | 3.19E+05 | 4.54E+05 | 3.56E+05 | 4.90E+05 | 3.13E+05 | 2.98E+05 | 3.14E+05 | 3.08E+05 | 3.49E+05 | 3.66E+05 | -- | -- |
| Jmwn002620 | 3.41E+02 | 1.61E+02 | 3.42E+02 | C_15_H_18_O_9_ | [M-H]- | Vanillic Acid-4-O-Glucuronide | Phenolic acids | Phenolic acids | - | 2 | 4.66E+04 | 4.74E+04 | 4.41E+04 | 3.56E+05 | 4.11E+05 | 3.66E+05 | 8.47E+04 | 9.49E+04 | 9.14E+04 | 4.00E+04 | 3.76E+04 | 3.62E+04 | 1.28E+05 | 1.25E+05 | 1.47E+05 | -- | -- |
| Zmhn001793 | 3.41E+02 | 1.79E+02 | 3.42E+02 | C_15_H_18_O_9_ | [M-H]- | 6-O-Caffeoyl-D-glucose* | Phenolic acids | Phenolic acids | - | 1 | 3.64E+05 | 3.16E+05 | 3.80E+05 | 2.37E+05 | 2.79E+05 | 2.77E+05 | 4.70E+05 | 4.87E+05 | 5.05E+05 | 3.02E+05 | 3.27E+05 | 2.97E+05 | 3.46E+05 | 3.03E+05 | 3.33E+05 | -- | -- |
| mws5038 | 3.41E+02 | 8.90E+01 | 3.42E+02 | C_12_H_22_O_11_ | [M-H]- | Isomaltulose | Others | Saccharides and Alcohols | 13718-94-0 | 1 | 4.69E+06 | 3.95E+06 | 3.62E+06 | 6.44E+06 | 8.02E+06 | 5.52E+06 | 6.19E+06 | 4.19E+06 | 7.31E+06 | 5.27E+06 | 4.45E+06 | 4.85E+06 | 4.34E+06 | 8.26E+06 | 5.92E+06 | C01742 | -- |
| MA10039641 | 3.41E+02 | 8.90E+01 | 3.42E+02 | C_12_H_22_O_11_ | [M-H]- | Lactobiose | Others | Saccharides and Alcohols | 63-42-3 | 1 | 4.67E+06 | 3.76E+06 | 3.90E+06 | 5.74E+06 | 4.65E+06 | 5.60E+06 | 6.80E+06 | 5.86E+06 | 7.09E+06 | 6.06E+06 | 6.80E+06 | 4.11E+06 | 6.28E+06 | 6.78E+06 | 6.40E+06 | C00243 | ko00052,ko01100,ko02010 |
| mws1333 | 3.41E+02 | 1.13E+02 | 3.42E+02 | C_12_H_22_O_11_ | [M-H]- | Melibiose | Others | Saccharides and Alcohols | 585-99-9 | 1 | 2.00E+06 | 2.25E+06 | 2.11E+06 | 3.14E+06 | 2.91E+06 | 2.58E+06 | 3.37E+06 | 3.48E+06 | 2.86E+06 | 3.31E+06 | 4.17E+06 | 2.81E+06 | 2.83E+06 | 3.14E+06 | 2.92E+06 | C05402 | ko00052,ko01100,ko02010 |
| mws1080 | 3.41E+02 | 1.79E+02 | 3.42E+02 | C_12_H_22_O_11_ | [M-H]- | Galactinol | Others | Saccharides and Alcohols | 3687-64-7 | 1 | 1.64E+07 | 1.79E+07 | 1.55E+07 | 1.91E+07 | 2.06E+07 | 2.48E+07 | 2.17E+07 | 2.44E+07 | 2.35E+07 | 2.01E+07 | 2.20E+07 | 2.31E+07 | 2.06E+07 | 2.23E+07 | 1.91E+07 | C01235 | ko00052,ko01100 |
| Lmsn000381 | 3.41E+02 | 8.90E+01 | 3.42E+02 | C_12_H_22_O_11_ | [M-H]- | D-Maltose | Others | Saccharides and Alcohols | 69-79-4 | 1 | 2.99E+06 | 4.00E+06 | 3.88E+06 | 4.28E+06 | 5.71E+06 | 9.89E+06 | 5.09E+06 | 4.60E+06 | 5.66E+06 | 6.02E+06 | 5.25E+06 | 5.19E+06 | 5.02E+06 | 4.05E+06 | 6.96E+06 | C01971 | -- |
| mws0264 | 3.41E+02 | 1.19E+02 | 3.42E+02 | C_12_H_22_O_11_ | [M-H]- | D-Trehalose | Others | Saccharides and Alcohols | 99-20-7 | 1 | 3.35E+06 | 2.25E+06 | 2.25E+06 | 3.24E+06 | 3.01E+06 | 3.93E+06 | 4.07E+06 | 3.55E+06 | 3.21E+06 | 3.21E+06 | 4.14E+06 | 3.28E+06 | 3.37E+06 | 3.88E+06 | 3.49E+06 | C01083 | ko00500,ko01100,ko01110,ko02010 |
| pme0519 | 3.41E+02 | 1.19E+02 | 3.42E+02 | C_12_H_22_O_11_ | [M-H]- | D-Sucrose | Others | Saccharides and Alcohols | 57-50-1 | 1 | 5.03E+06 | 6.25E+06 | 5.55E+06 | 9.30E+06 | 6.74E+06 | 7.55E+06 | 9.09E+06 | 7.43E+06 | 8.29E+06 | 8.79E+06 | 9.58E+06 | 9.60E+06 | 6.38E+06 | 6.77E+06 | 9.12E+06 | C00089 | ko00052,ko00500,ko01100,ko01110,ko02010 |
| mws0906 | 3.41E+02 | 1.79E+02 | 3.42E+02 | C_16_H_22_O_8_ | [M-H]- | Coniferin | Phenolic acids | Phenolic acids | 531-29-3 | 2 | 4.35E+05 | 3.42E+05 | 4.27E+05 | 2.90E+05 | 2.99E+05 | 3.26E+05 | 4.39E+05 | 4.52E+05 | 5.17E+05 | 3.39E+05 | 1.81E+05 | 3.23E+05 | 3.79E+05 | 3.56E+05 | 4.15E+05 | C00761 | ko00940 |
| pmb2211 | 3.43E+02 | 2.41E+02 | 3.42E+02 | C_19_H_38_N_2_O_3_ | [M+H]+ | Cocamidopropyl betaine | Alkaloids | Alkaloids | 4292-10-8 | 2 | 2.80E+04 | 4.45E+04 | 3.83E+04 | 3.93E+04 | 3.24E+04 | 4.13E+04 | 4.70E+04 | 2.61E+04 | 3.87E+04 | 4.04E+04 | 3.34E+04 | 4.35E+04 | 3.90E+04 | 3.60E+04 | 3.86E+04 | -- | -- |
| Zmln000899 | 3.43E+02 | 1.81E+02 | 3.44E+02 | C_15_H_20_O_9_ | [M-H]- | Syringaldehyde-4-O-glucoside | Phenolic acids | Phenolic acids | - | 2 | 1.94E+05 | 2.16E+05 | 2.08E+05 | 3.24E+05 | 3.61E+05 | 3.07E+05 | 2.16E+05 | 2.30E+05 | 2.22E+05 | 2.61E+05 | 2.81E+05 | 2.65E+05 | 2.23E+05 | 2.42E+05 | 2.66E+05 | -- | -- |
| Lmmn000774 | 3.43E+02 | 1.81E+02 | 3.44E+02 | C_15_H_20_O_9_ | [M-H]- | Dihydrocaffeoylglucose | Phenolic acids | Phenolic acids | - | 3 | 1.37E+05 | 1.31E+05 | 1.31E+05 | 1.76E+05 | 2.42E+05 | 1.91E+05 | 1.17E+05 | 1.09E+05 | 1.24E+05 | 1.52E+05 | 1.85E+05 | 1.53E+05 | 1.53E+05 | 1.72E+05 | 2.02E+05 | -- | -- |
| MWSslk074 | 3.43E+02 | 5.90E+01 | 3.44E+02 | C_12_H_24_O_11_ | [M-H]- | Lactitol | Others | Saccharides and Alcohols | 585-86-4 | 2 | 1.47E+06 | 1.48E+06 | 1.59E+06 | 2.44E+06 | 2.46E+06 | 2.42E+06 | 2.52E+06 | 2.47E+06 | 2.47E+06 | 2.81E+06 | 3.60E+06 | 2.70E+06 | 2.14E+06 | 2.25E+06 | 2.71E+06 | -- | -- |
| mws0609 | 3.44E+02 | 1.50E+02 | 3.45E+02 | C_10_H_12_N_5_O_7_P | [M-H]- | Guanosine 3',5'-cyclic monophosphate | Nucleotides and derivatives | Nucleotides and derivatives | 7665-99-8 | 2 | 2.25E+05 | 2.41E+05 | 2.59E+05 | 7.83E+04 | 7.46E+04 | 6.96E+04 | 3.75E+06 | 3.65E+06 | 4.09E+06 | 2.09E+05 | 2.39E+05 | 2.25E+05 | 9.04E+05 | 1.07E+06 | 1.23E+06 | C00942 | ko00230,ko01100 |
| pmb0801 | 3.46E+02 | 1.84E+02 | 3.45E+02 | C_14_H_19_NO_9_ | [M+H]+ | 4-Pyridoxic acid-O-glucoside | Others | Vitamin | - | 3 | 7.21E+03 | 1.33E+04 | 9.73E+03 | 1.39E+04 | 1.93E+04 | 1.12E+04 | 8.58E+03 | 1.06E+04 | 1.63E+04 | 1.27E+04 | 8.57E+03 | 1.34E+04 | 1.18E+04 | 6.78E+03 | 1.28E+04 | -- | -- |
| pmp000303 | 3.47E+02 | 2.05E+02 | 3.46E+02 | C_19_H_22_O_6_ | [M+H]+ | 30-O-I-Butyrylhamaudol | Others | Others | - | 3 | 7.45E+03 | 7.87E+03 | 5.95E+03 | 9.00E+00 | 9.00E+00 | 9.00E+00 | 2.19E+03 | 2.76E+03 | 2.90E+03 | 9.00E+00 | 9.00E+00 | 9.00E+00 | 3.01E+03 | 3.77E+03 | 3.68E+03 | -- | -- |
| pmb0981 | 3.48E+02 | 1.36E+02 | 3.47E+02 | C_10_H_14_N_5_O_7_P | [M+H]+ | Adenosine 5'-monophosphate | Nucleotides and derivatives | Nucleotides and derivatives | 61-19-8 | 3 | 1.26E+06 | 1.12E+06 | 1.25E+06 | 2.45E+06 | 2.36E+06 | 2.39E+06 | 3.12E+06 | 3.61E+06 | 3.69E+06 | 1.91E+06 | 1.67E+06 | 1.98E+06 | 2.17E+06 | 1.89E+06 | 2.76E+06 | C00020 | ko00230,ko00908,ko01100,ko01110,ko01240 |
| pmf0557 | 3.27E+02 | 1.71E+02 | 3.50E+02 | C_16_H_11_N_2_NaO_4_S | [M-Na]- | Orange I | Alkaloids | Alkaloids | 523-44-4 | 2 | 8.44E+04 | 9.54E+04 | 9.14E+04 | 1.89E+05 | 1.66E+05 | 1.63E+05 | 7.23E+04 | 7.84E+04 | 7.75E+04 | 4.97E+04 | 5.75E+04 | 5.11E+04 | 9.86E+04 | 1.15E+05 | 1.02E+05 | C19371 | -- |
| pmb1562 | 3.51E+02 | 1.47E+02 | 3.50E+02 | C_21_H_34_O_4_ | [M+H]+ | 1-Stearidonoyl-Glycerol | Lipids | Glycerol ester | - | 3 | 1.59E+04 | 2.21E+04 | 1.88E+04 | 1.96E+04 | 2.12E+04 | 1.52E+04 | 3.11E+04 | 3.13E+04 | 3.50E+04 | 7.13E+03 | 7.30E+03 | 6.22E+03 | 1.32E+04 | 2.18E+04 | 2.18E+04 | -- | -- |
| pmb3093 | 3.51E+02 | 1.05E+02 | 3.52E+02 | C_16_H_16_O_9_ | [M-H]- | Esculetin-7-O-quinic acid | Lignans and Coumarins | Coumarins | - | 3 | 2.37E+05 | 2.87E+05 | 2.74E+05 | 8.85E+05 | 9.85E+05 | 6.80E+05 | 1.46E+06 | 1.17E+06 | 1.40E+06 | 1.72E+06 | 1.67E+06 | 1.97E+06 | 9.93E+05 | 1.20E+06 | 1.09E+06 | -- | -- |
| pmb0808 | 3.53E+02 | 1.79E+02 | 3.52E+02 | C_16_H_16_O_9_ | [M+H]+ | Esculetin-6-O-quinic acid | Lignans and Coumarins | Coumarins | - | 3 | 1.66E+05 | 2.24E+05 | 1.51E+05 | 3.44E+05 | 5.06E+05 | 4.40E+05 | 2.64E+05 | 2.76E+05 | 3.40E+05 | 3.42E+05 | 2.47E+05 | 3.52E+05 | 3.46E+05 | 2.78E+05 | 3.25E+05 | -- | -- |
| pmb0770 | 3.53E+02 | 1.77E+02 | 3.52E+02 | C_20_H_20_N_2_O_4_ | [M+H]+ | N-Feruloylserotonin | Alkaloids | Plumerane | 68573-23-9 | 3 | 9.00E+00 | 9.00E+00 | 9.00E+00 | 2.03E+04 | 1.63E+04 | 2.05E+04 | 9.00E+00 | 9.00E+00 | 9.00E+00 | 1.12E+04 | 8.54E+03 | 1.04E+04 | 9.64E+03 | 1.39E+04 | 1.02E+04 | -- | -- |
| Lmhp011562 | 3.53E+02 | 2.61E+02 | 3.52E+02 | C_21_H_36_O_4_ | [M+H]+ | 1-α-Linolenoyl-glycerol | Lipids | Glycerol ester | - | 1 | 2.51E+05 | 2.72E+05 | 2.61E+05 | 4.45E+05 | 3.84E+05 | 3.75E+05 | 2.75E+05 | 2.86E+05 | 2.95E+05 | 4.04E+05 | 4.30E+05 | 4.44E+05 | 3.45E+05 | 3.56E+05 | 4.24E+05 | -- | -- |
| Lmhp011388 | 3.53E+02 | 2.61E+02 | 3.52E+02 | C_21_H_36_O_4_ | [M+H]+ | 2-α-Linolenoyl-glycerol | Lipids | Glycerol ester | 55268-58-1 | 1 | 2.34E+04 | 2.61E+04 | 2.80E+04 | 5.94E+04 | 5.54E+04 | 6.27E+04 | 2.77E+04 | 3.02E+04 | 2.99E+04 | 5.72E+04 | 5.68E+04 | 5.00E+04 | 4.35E+04 | 4.63E+04 | 4.55E+04 | -- | -- |
| mws0178 | 3.53E+02 | 1.91E+02 | 3.54E+02 | C_16_H_18_O_9_ | [M-H]- | Chlorogenic acid (3-O-Caffeoylquinic acid)* | Phenolic acids | Phenolic acids | 327-97-9 | 1 | 1.41E+06 | 1.65E+06 | 1.51E+06 | 5.75E+06 | 5.14E+06 | 5.28E+06 | 3.19E+06 | 3.12E+06 | 3.39E+06 | 3.33E+06 | 2.92E+06 | 2.82E+06 | 2.95E+06 | 3.32E+06 | 3.93E+06 | C00852 | ko00940,ko00941,ko00945,ko01110 |
| mws1077 | 3.55E+02 | 1.93E+02 | 3.54E+02 | C_16_H_18_O_9_ | [M+H]+ | Scopoletin-7-O-glucoside (Scopolin) | Lignans and Coumarins | Coumarins | 531-44-2 | 2 | 4.52E+07 | 4.57E+07 | 4.46E+07 | 5.63E+07 | 5.75E+07 | 5.60E+07 | 9.70E+06 | 1.09E+07 | 1.17E+07 | 1.21E+07 | 1.22E+07 | 1.29E+07 | 3.17E+07 | 3.91E+07 | 3.63E+07 | C01527 | ko00940,ko01110 |
| mws2108 | 3.53E+02 | 1.91E+02 | 3.54E+02 | C_16_H_18_O_9_ | [M-H]- | Cryptochlorogenic acid (4-O-Caffeoylquinic acid)* | Phenolic acids | Phenolic acids | 905-99-7 | 1 | 1.86E+06 | 2.51E+06 | 1.80E+06 | 6.50E+06 | 5.89E+06 | 6.07E+06 | 4.57E+06 | 4.20E+06 | 4.66E+06 | 3.73E+06 | 4.27E+06 | 3.84E+06 | 4.45E+06 | 4.38E+06 | 4.50E+06 | -- | -- |
| pme1816 | 3.53E+02 | 1.91E+02 | 3.54E+02 | C_16_H_18_O_9_ | [M-H]- | Neochlorogenic acid (5-O-Caffeoylquinic acid) | Phenolic acids | Phenolic acids | 906-33-2 | 1 | 6.01E+06 | 6.89E+06 | 6.40E+06 | 1.88E+07 | 2.21E+07 | 1.86E+07 | 2.95E+07 | 2.65E+07 | 2.96E+07 | 3.24E+07 | 3.51E+07 | 4.14E+07 | 2.09E+07 | 2.42E+07 | 2.62E+07 | C17147 | -- |
| mws1727 | 3.55E+02 | 1.03E+02 | 3.54E+02 | C_21_H_22_O_5_ | [M+H]+ | Notopterol | Lignans and Coumarins | Coumarins | 88206-46-6 | 3 | 3.39E+03 | 4.38E+03 | 5.40E+03 | 6.46E+03 | 5.31E+03 | 2.64E+03 | 6.15E+03 | 5.79E+03 | 5.09E+03 | 6.39E+03 | 4.40E+03 | 9.41E+03 | 4.44E+03 | 7.84E+03 | 4.28E+03 | C17499 | -- |
| Lmhp112042 | 3.55E+02 | 2.63E+02 | 3.54E+02 | C_21_H_38_O_4_ | [M+H]+ | 1-Linoleoylglycerol* | Lipids | Glycerol ester | 2277-28-3 | 1 | 6.41E+05 | 6.23E+05 | 6.86E+05 | 9.99E+05 | 9.71E+05 | 8.61E+05 | 6.53E+05 | 6.97E+05 | 7.15E+05 | 9.49E+05 | 9.71E+05 | 1.04E+06 | 7.91E+05 | 7.89E+05 | 8.97E+05 | -- | -- |
| Lmhp012042 | 3.55E+02 | 2.63E+02 | 3.54E+02 | C_21_H_38_O_4_ | [M+H]+ | 2-Linoleoylglycerol* | Lipids | Glycerol ester | 3443-82-1 | 1 | 1.74E+05 | 1.63E+05 | 1.75E+05 | 3.13E+05 | 2.82E+05 | 2.71E+05 | 1.32E+05 | 1.44E+05 | 1.65E+05 | 2.37E+05 | 2.35E+05 | 2.25E+05 | 2.24E+05 | 2.32E+05 | 2.49E+05 | -- | -- |
| Lmhn001580 | 3.55E+02 | 2.23E+02 | 3.56E+02 | C_15_H_16_O_10_ | [M-H]- | Sinapoyltartaric acid | Phenolic acids | Phenolic acids | - | 3 | 7.62E+04 | 1.07E+05 | 1.02E+05 | 1.42E+05 | 1.24E+05 | 1.24E+05 | 4.40E+04 | 4.72E+04 | 3.90E+04 | 4.27E+04 | 4.38E+04 | 6.46E+04 | 8.39E+04 | 9.94E+04 | 1.05E+05 | -- | -- |
| Zmhn002422 | 3.55E+02 | 1.93E+02 | 3.56E+02 | C_16_H_20_O_9_ | [M-H]- | 1-O-Feruloyl-D-Glucose | Phenolic acids | Phenolic acids | - | 3 | 2.21E+05 | 2.23E+05 | 2.51E+05 | 2.47E+05 | 2.63E+05 | 3.29E+05 | 5.96E+05 | 5.64E+05 | 5.89E+05 | 6.28E+05 | 6.61E+05 | 6.24E+05 | 4.00E+05 | 4.39E+05 | 4.40E+05 | C17759 | -- |
| Hmmn002544 | 3.55E+02 | 1.93E+02 | 3.56E+02 | C_16_H_20_O_9_ | [M-H]- | Ferulic acid-4-O-glucoside | Phenolic acids | Phenolic acids | 117405-51-3 | 2 | 1.02E+05 | 1.13E+05 | 1.35E+05 | 3.95E+05 | 4.29E+05 | 4.01E+05 | 5.39E+05 | 5.88E+05 | 6.24E+05 | 6.76E+05 | 6.68E+05 | 8.67E+05 | 4.50E+05 | 4.65E+05 | 5.39E+05 | -- | -- |
| pmb0296 | 3.57E+02 | 2.65E+02 | 3.56E+02 | C_21_H_40_O_4_ | [M+H]+ | 1-Oleoyl-Sn-Glycerol | Lipids | Glycerol ester | 129784-87-8 | 1 | 6.69E+05 | 5.39E+05 | 6.54E+05 | 2.04E+06 | 1.86E+06 | 2.02E+06 | 8.91E+05 | 9.51E+05 | 8.86E+05 | 1.96E+06 | 2.06E+06 | 2.03E+06 | 1.58E+06 | 1.34E+06 | 1.60E+06 | -- | -- |
| Lmdn004267 | 3.57E+02 | 1.51E+02 | 3.58E+02 | C_20_H_22_O_6_ | [M-H]- | Epipinoresinol* | Lignans and Coumarins | Lignans | 24404-50-0 | 1 | 6.43E+03 | 9.11E+03 | 5.61E+03 | 3.19E+04 | 2.53E+04 | 3.20E+04 | 1.97E+05 | 1.84E+05 | 2.65E+05 | 5.32E+04 | 4.97E+04 | 4.48E+04 | 6.91E+04 | 7.80E+04 | 8.35E+04 | -- | -- |
| Lmsp004450 | 3.59E+02 | 1.31E+02 | 3.58E+02 | C_20_H_22_O_6_ | [M+H]+ | Dehydrodiconiferyl alcohol | Phenolic acids | Phenolic acids | 97465-82-2 | 1 | 3.35E+04 | 3.71E+04 | 2.67E+04 | 4.74E+04 | 3.10E+04 | 4.41E+04 | 7.25E+04 | 7.28E+04 | 9.86E+04 | 5.26E+04 | 5.60E+04 | 6.19E+04 | 5.78E+04 | 5.97E+04 | 6.49E+04 | -- | -- |
| mws0097 | 3.57E+02 | 1.51E+02 | 3.58E+02 | C_20_H_22_O_6_ | [M-H]- | Pinoresinol* | Lignans and Coumarins | Lignans | 487-36-5 | 1 | 7.98E+03 | 7.24E+03 | 9.49E+03 | 3.73E+04 | 2.81E+04 | 3.05E+04 | 2.33E+05 | 2.17E+05 | 3.08E+05 | 6.05E+04 | 5.01E+04 | 4.90E+04 | 8.05E+04 | 8.02E+04 | 9.95E+04 | C05366 | ko00998,ko01100,ko01110 |
| Zmdp008582 | 3.59E+02 | 2.19E+02 | 3.58E+02 | C_22_H_30_O_4_ | [M+H]+ | Cannabichromenic acid(CBCA) | Others | Others | 20408-52-0 | 2 | 6.74E+06 | 4.87E+06 | 4.77E+06 | 5.08E+06 | 4.99E+06 | 4.52E+06 | 1.09E+06 | 1.23E+06 | 1.13E+06 | 1.78E+06 | 2.00E+06 | 1.94E+06 | 3.31E+06 | 3.55E+06 | 3.89E+06 | -- | -- |
| pmb3107 | 3.59E+02 | 1.82E+02 | 3.60E+02 | C_15_H_20_O_10_ | [M-H]- | Glucosyringic Acid | Phenolic acids | Phenolic acids | - | 3 | 1.99E+04 | 1.98E+04 | 1.69E+04 | 2.83E+04 | 2.98E+04 | 2.73E+04 | 2.67E+04 | 2.61E+04 | 2.84E+04 | 2.77E+04 | 3.05E+04 | 2.60E+04 | 2.22E+04 | 2.40E+04 | 2.86E+04 | -- | -- |
| pmb0998 | 3.64E+02 | 1.52E+02 | 3.63E+02 | C_10_H_14_N_5_O_8_P | [M+H]+ | Guanosine 5'-monophosphate | Nucleotides and derivatives | Nucleotides and derivatives | 85-32-5 | 2 | 5.64E+04 | 5.53E+04 | 5.94E+04 | 5.59E+04 | 5.43E+04 | 4.03E+04 | 1.06E+05 | 1.11E+05 | 1.21E+05 | 6.14E+04 | 5.40E+04 | 5.85E+04 | 7.61E+04 | 6.37E+04 | 8.28E+04 | C00144 | ko00230,ko01100 |
| pmb0964 | 3.66E+02 | 2.04E+02 | 3.65E+02 | C_16_H_23_N_5_O_5_ | [M+H]+ | Isopentenyladenine-7-N-glucoside | Nucleotides and derivatives | Nucleotides and derivatives | - | 3 | 2.93E+04 | 3.90E+04 | 3.76E+04 | 2.75E+04 | 3.94E+04 | 5.31E+04 | 3.62E+04 | 3.31E+04 | 3.77E+04 | 3.26E+04 | 2.89E+04 | 2.27E+04 | 1.46E+04 | 3.11E+04 | 2.65E+04 | -- | -- |
| Lmgp003270 | 3.69E+02 | 1.77E+02 | 3.68E+02 | C_16_H_16_O_10_ | [M+H]+ | Scopoletin-7-O-glucuronide | Lignans and Coumarins | Coumarins | 132752-65-9 | 2 | 5.67E+06 | 5.96E+06 | 6.38E+06 | 1.06E+07 | 1.32E+07 | 1.12E+07 | 1.45E+07 | 1.47E+07 | 1.40E+07 | 1.54E+07 | 1.76E+07 | 1.54E+07 | 9.60E+06 | 1.20E+07 | 1.15E+07 | -- | -- |
| pmb0752 | 3.69E+02 | 1.77E+02 | 3.68E+02 | C_17_H_20_O_9_ | [M+H]+ | 3-O-Feruloylquinic acid | Phenolic acids | Phenolic acids | 1899-29-2 | 1 | 1.12E+07 | 9.09E+06 | 1.08E+07 | 8.65E+06 | 8.81E+06 | 8.24E+06 | 2.52E+07 | 2.26E+07 | 2.39E+07 | 2.58E+07 | 2.94E+07 | 2.88E+07 | 1.69E+07 | 1.35E+07 | 1.70E+07 | C02572 | -- |
| pma3724 | 3.69E+02 | 1.77E+02 | 3.68E+02 | C_17_H_20_O_9_ | [M+H]+ | 1-O-Feruloylquinic acid | Phenolic acids | Phenolic acids | - | 3 | 4.73E+06 | 4.70E+06 | 5.98E+06 | 9.65E+06 | 9.75E+06 | 1.01E+07 | 1.23E+07 | 1.18E+07 | 1.18E+07 | 1.49E+07 | 1.39E+07 | 1.37E+07 | 1.00E+07 | 8.72E+06 | 9.27E+06 | -- | -- |
| mws0179 | 3.67E+02 | 1.91E+02 | 3.68E+02 | C_17_H_20_O_9_ | [M-H]- | Chlorogenic acid methyl ester | Phenolic acids | Phenolic acids | 29708-87-0 | 2 | 6.80E+05 | 6.63E+05 | 6.77E+05 | 1.22E+06 | 1.29E+06 | 1.38E+06 | 1.21E+06 | 1.34E+06 | 1.35E+06 | 1.47E+06 | 1.44E+06 | 1.55E+06 | 1.19E+06 | 1.13E+06 | 1.17E+06 | -- | -- |
| Lmgn003073 | 7.35E+02 | 3.67E+02 | 3.68E+02 | C_17_H_20_O_9_ | [2M-H]- | 5-O-Feruloylquinic acid | Phenolic acids | Phenolic acids | - | 1 | 1.77E+06 | 2.24E+06 | 2.57E+06 | 6.12E+06 | 8.09E+06 | 6.41E+06 | 7.06E+06 | 8.56E+06 | 8.46E+06 | 8.23E+06 | 1.14E+07 | 1.07E+07 | 5.26E+06 | 7.32E+06 | 7.27E+06 | -- | -- |
| MWSmce025 | 3.69E+02 | 2.07E+02 | 3.70E+02 | C_16_H_18_O_10_ | [M-H]- | Fraxin | Lignans and Coumarins | Coumarins | 524-30-1 | 2 | 4.03E+05 | 4.08E+05 | 4.47E+05 | 1.95E+06 | 1.97E+06 | 1.81E+06 | 4.31E+05 | 4.56E+05 | 6.38E+05 | 4.85E+05 | 4.90E+05 | 4.56E+05 | 6.96E+05 | 8.87E+05 | 8.16E+05 | C09266 | -- |
| Lmbp002309 | 3.71E+02 | 2.09E+02 | 3.70E+02 | C_17_H_22_O_9_ | [M+H]+ | Sinapaldehyde-4-O-Glucoside | Phenolic acids | Phenolic acids | 154461-65-1 | 2 | 1.21E+05 | 1.23E+05 | 1.23E+05 | 2.33E+05 | 2.43E+05 | 2.93E+05 | 1.74E+05 | 1.59E+05 | 1.98E+05 | 1.52E+05 | 1.82E+05 | 1.37E+05 | 1.51E+05 | 1.75E+05 | 2.07E+05 | -- | -- |
| Hmgn002833 | 3.71E+02 | 2.97E+02 | 3.72E+02 | C_20_H_20_O_7_ | [M-H]- | 4-Ketopinoresinol | Others | Others | - | 1 | 8.84E+04 | 6.38E+04 | 1.04E+05 | 5.47E+04 | 6.00E+04 | 6.18E+04 | 5.66E+04 | 5.65E+04 | 7.43E+04 | 2.45E+04 | 3.49E+04 | 3.23E+04 | 5.88E+04 | 7.32E+04 | 7.43E+04 | -- | -- |
| mws0055 | 3.73E+02 | 3.43E+02 | 3.72E+02 | C_20_H_20_O_7_ | [M+H]+ | Tangeretin (4',5,6,7,8-Pentamethoxyflavone) | Flavonoids | Flavones | 481-53-8 | 3 | 4.22E+04 | 4.39E+04 | 4.10E+04 | 3.97E+04 | 3.26E+04 | 3.63E+04 | 3.16E+04 | 3.53E+04 | 3.91E+04 | 3.53E+04 | 2.60E+04 | 4.13E+04 | 3.74E+04 | 3.42E+04 | 3.97E+04 | C10190 | -- |
| Hmhp008730 | 3.73E+02 | 2.03E+02 | 3.72E+02 | C_21_H_24_O_6_ | [M+H]+ | 6',7'-Dihydroxybergamottin | Lignans and Coumarins | Coumarins | 145414-76-2 | 3 | 1.13E+04 | 3.76E+03 | 5.86E+03 | 4.02E+03 | 1.30E+04 | 9.87E+03 | 6.16E+03 | 7.45E+03 | 4.72E+03 | 8.22E+03 | 6.10E+03 | 5.21E+03 | 8.34E+03 | 3.27E+03 | 5.92E+03 | C22156 | -- |
| Hmgn004139 | 3.73E+02 | 3.29E+02 | 3.74E+02 | C_20_H_22_O_7_ | [M-H]- | Isohydroxymatairesinol | Lignans and Coumarins | Lignans | - | 3 | 2.85E+04 | 2.07E+04 | 2.33E+04 | 5.40E+03 | 7.25E+03 | 6.44E+03 | 9.32E+03 | 9.43E+03 | 1.15E+04 | 8.81E+03 | 7.56E+03 | 8.71E+03 | 1.10E+04 | 1.29E+04 | 1.76E+04 | -- | -- |
| mws0232 | 3.77E+02 | 2.43E+02 | 3.76E+02 | C_17_H_20_N_4_O_6_ | [M+H]+ | Riboflavin (Vitamin B2) | Others | Vitamin | 83-88-5 | 1 | 6.21E+05 | 6.69E+05 | 5.99E+05 | 1.30E+05 | 1.31E+05 | 1.23E+05 | 2.58E+05 | 2.47E+05 | 2.47E+05 | 9.09E+04 | 8.94E+04 | 8.40E+04 | 2.80E+05 | 2.59E+05 | 3.11E+05 | C00255 | ko00740,ko01100,ko01110,ko01240,ko02010 |
| pmp001132 | 3.76E+02 | 3.58E+02 | 3.76E+02 | C_22_H_34_NO_4_+ | [M]+ | N-Isopentenyl-6-hydroxydendroxinium | Alkaloids | Alkaloids | - | 2 | 3.05E+04 | 1.94E+04 | 2.09E+04 | 9.00E+00 | 9.00E+00 | 9.00E+00 | 1.04E+04 | 1.09E+04 | 5.52E+03 | 9.00E+00 | 9.00E+00 | 9.00E+00 | 9.34E+03 | 7.52E+03 | 8.97E+03 | -- | -- |
| Hmap010126 | 3.81E+02 | 1.63E+02 | 3.80E+02 | C_24_H_28_O_4_ | [M+H]+ | Badrakemone | Lignans and Coumarins | Coumarins | - | 3 | 5.60E+03 | 6.42E+03 | 6.82E+03 | 4.85E+03 | 6.68E+03 | 5.86E+03 | 1.84E+04 | 1.95E+04 | 1.94E+04 | 5.65E+03 | 4.12E+03 | 4.65E+03 | 1.06E+04 | 7.51E+03 | 6.87E+03 | -- | -- |
| pmp000258 | 3.81E+02 | 1.45E+02 | 3.80E+02 | C_24_H_28_O_4_ | [M+H]+ | Z-Ligustilide dimer E-232 | Others | Others | - | 3 | 9.31E+03 | 1.14E+04 | 8.67E+03 | 2.38E+03 | 1.02E+03 | 2.66E+03 | 6.25E+03 | 4.91E+03 | 5.43E+03 | 2.40E+03 | 2.74E+03 | 1.20E+03 | 4.51E+03 | 4.38E+03 | 4.87E+03 | -- | -- |
| pmp000257 | 3.81E+02 | 1.45E+02 | 3.80E+02 | C_24_H_28_O_4_ | [M+H]+ | Levistolide A | Others | Others | 88182-33-6 | 3 | 3.45E+03 | 2.86E+03 | 2.34E+03 | 2.73E+03 | 3.21E+03 | 1.80E+03 | 6.07E+03 | 5.80E+03 | 8.26E+03 | 8.03E+03 | 6.93E+03 | 9.80E+03 | 4.14E+03 | 4.06E+03 | 6.26E+03 | -- | -- |
| pmp000255 | 3.81E+02 | 2.93E+02 | 3.80E+02 | C_24_H_28_O_4_ | [M+H]+ | Z,Z9-6.89,7.39-Diligustilide | Others | Others | - | 3 | 5.22E+04 | 5.33E+04 | 4.93E+04 | 9.00E+00 | 9.00E+00 | 9.00E+00 | 7.50E+03 | 7.47E+03 | 7.37E+03 | 9.00E+00 | 9.00E+00 | 9.00E+00 | 1.74E+04 | 1.57E+04 | 1.47E+04 | -- | -- |
| Hmap010529 | 3.83E+02 | 1.63E+02 | 3.82E+02 | C_24_H_30_O_4_ | [M+H]+ | farnesiferol B | Lignans and Coumarins | Coumarins | - | 2 | 5.69E+05 | 4.19E+05 | 5.70E+05 | 3.11E+05 | 3.98E+05 | 3.71E+05 | 1.39E+06 | 1.48E+06 | 1.17E+06 | 7.88E+05 | 8.05E+05 | 8.88E+05 | 7.63E+05 | 8.56E+05 | 7.08E+05 | -- | -- |
| Hmap008482 | 3.83E+02 | 1.63E+02 | 3.82E+02 | C_24_H_30_O_4_ | [M+H]+ | Assafoetidin | Lignans and Coumarins | Coumarins | - | 3 | 4.94E+04 | 3.02E+04 | 4.17E+04 | 2.81E+04 | 2.82E+04 | 2.40E+04 | 7.74E+04 | 7.83E+04 | 7.61E+04 | 3.93E+04 | 3.32E+04 | 4.37E+04 | 5.64E+04 | 4.48E+04 | 5.47E+04 | -- | -- |
| pme3337 | 3.84E+02 | 2.52E+02 | 3.83E+02 | C_14_H_17_N_5_O_8_ | [M+H]+ | Succinyladenosine | Nucleotides and derivatives | Nucleotides and derivatives | 4542-23-8 | 1 | 3.87E+07 | 3.81E+07 | 4.03E+07 | 8.86E+06 | 8.37E+06 | 7.54E+06 | 2.01E+07 | 1.91E+07 | 2.00E+07 | 7.06E+06 | 7.60E+06 | 6.36E+06 | 2.27E+07 | 2.07E+07 | 2.34E+07 | -- | -- |
| Lmbp003208 | 3.85E+02 | 2.23E+02 | 3.84E+02 | C_17_H_20_O_10_ | [M+H]+ | Fraxidin-8-O-glucoside | Lignans and Coumarins | Coumarins | - | 2 | 8.38E+04 | 7.81E+04 | 8.89E+04 | 9.21E+04 | 1.12E+05 | 9.67E+04 | 1.05E+05 | 1.05E+05 | 6.79E+04 | 1.32E+05 | 1.59E+05 | 1.46E+05 | 1.16E+05 | 9.96E+04 | 1.01E+05 | -- | -- |
| Lmjp003090 | 3.85E+02 | 2.23E+02 | 3.84E+02 | C_17_H_20_O_10_ | [M+H]+ | Isofraxidin-7-O-glucoside | Lignans and Coumarins | Coumarins | 483-91-0 | 2 | 3.00E+04 | 3.55E+04 | 3.87E+04 | 4.89E+04 | 6.60E+04 | 5.69E+04 | 2.44E+04 | 1.21E+04 | 1.48E+04 | 3.90E+04 | 3.63E+04 | 4.81E+04 | 2.52E+04 | 3.84E+04 | 3.60E+04 | -- | -- |
| pme1286 | 3.85E+02 | 2.50E+02 | 3.84E+02 | C_14_H_20_N_6_O_5_S | [M+H]+ | S-(5'-Adenosy)-L-homocysteine | Amino acids and derivatives | Amino acids and derivatives | 979-92-0 | 3 | 1.95E+05 | 2.16E+05 | 1.80E+05 | 1.30E+05 | 1.29E+05 | 1.30E+05 | 2.99E+05 | 3.06E+05 | 2.90E+05 | 1.15E+05 | 1.18E+05 | 1.34E+05 | 1.76E+05 | 1.89E+05 | 1.84E+05 | C00021 | ko00270,ko01100,ko01230,ko01240 |
| Hmap010207 | 3.85E+02 | 1.63E+02 | 3.84E+02 | C_24_H_32_O_4_ | [M+H]+ | fekrynol | Lignans and Coumarins | Coumarins | - | 3 | 9.00E+00 | 9.00E+00 | 9.00E+00 | 4.87E+03 | 3.27E+03 | 2.46E+03 | 3.68E+03 | 6.18E+03 | 3.66E+03 | 4.73E+03 | 5.08E+03 | 5.95E+03 | 4.76E+03 | 4.11E+03 | 3.61E+03 | -- | -- |
| pmb3062 | 3.85E+02 | 1.51E+02 | 3.86E+02 | C_17_H_22_O_10_ | [M-H]- | 1-O-Eudesmoylquinic acid | Phenolic acids | Phenolic acids | - | 1 | 6.11E+04 | 5.41E+04 | 7.16E+04 | 4.75E+04 | 5.81E+04 | 6.74E+04 | 1.99E+05 | 1.57E+05 | 1.80E+05 | 2.19E+05 | 2.15E+05 | 2.33E+05 | 1.17E+05 | 1.41E+05 | 1.54E+05 | -- | -- |
| Zmhn002227 | 3.85E+02 | 2.23E+02 | 3.86E+02 | C_17_H_22_O_10_ | [M-H]- | 4-O-Glucosyl-sinapate | Phenolic acids | Phenolic acids | - | 2 | 4.38E+05 | 4.18E+05 | 4.02E+05 | 3.20E+05 | 3.23E+05 | 3.67E+05 | 5.10E+04 | 4.48E+04 | 3.68E+04 | 5.13E+04 | 6.43E+04 | 6.73E+04 | 1.94E+05 | 2.57E+05 | 2.53E+05 | C02919 | -- |
| mws4168 | 3.87E+02 | 2.27E+02 | 3.86E+02 | C_21_H_22_O_7_ | [M+H]+ | Praeruptorin A | Lignans and Coumarins | Coumarins | 73069-25-7 | 3 | 1.78E+04 | 2.31E+04 | 2.19E+04 | 4.81E+04 | 5.37E+04 | 5.25E+04 | 3.55E+04 | 3.65E+04 | 4.37E+04 | 4.77E+04 | 5.18E+04 | 4.84E+04 | 4.04E+04 | 3.91E+04 | 4.06E+04 | -- | -- |
| Zmbp010005 | 3.87E+02 | 2.45E+02 | 3.86E+02 | C_21_H_22_O_7_ | [M+H]+ | Pteryxin | Lignans and Coumarins | Coumarins | 13161-75-6 | 3 | 5.85E+04 | 6.79E+04 | 6.08E+04 | 1.10E+05 | 1.11E+05 | 1.16E+05 | 1.12E+05 | 9.99E+04 | 1.06E+05 | 1.36E+05 | 1.21E+05 | 1.28E+05 | 1.02E+05 | 1.19E+05 | 1.16E+05 | -- | -- |
| Cmyp003257 | 3.87E+02 | 2.25E+02 | 3.86E+02 | C_19_H_30_O_8_ | [M+H]+ | Icariside B1 | Others | Others | 109062-00-2 | 1 | 3.98E+05 | 3.97E+05 | 3.17E+05 | 5.31E+05 | 4.39E+05 | 4.96E+05 | 3.48E+05 | 3.22E+05 | 2.71E+05 | 2.84E+05 | 2.40E+05 | 2.31E+05 | 2.97E+05 | 4.53E+05 | 4.48E+05 | -- | -- |
| Lmqp003013 | 3.87E+02 | 1.49E+02 | 3.86E+02 | C_19_H_30_O_8_ | [M+H]+ | Citroside A | Terpenoids | Terpene | 120330-44-1 | 2 | 5.77E+04 | 3.53E+04 | 2.83E+04 | 4.04E+04 | 8.24E+04 | 6.12E+04 | 5.52E+04 | 6.99E+04 | 7.30E+04 | 8.75E+04 | 9.27E+04 | 5.66E+04 | 7.97E+04 | 6.55E+04 | 4.18E+04 | -- | -- |
| Hmcn001415 | 3.87E+02 | 2.25E+02 | 3.88E+02 | C_17_H_24_O_10_ | [M-H]- | Majoroside | Others | Others | 134140-02-6 | 2 | 7.98E+03 | 1.21E+04 | 1.30E+04 | 1.62E+04 | 1.86E+04 | 2.09E+04 | 2.15E+04 | 2.13E+04 | 1.71E+04 | 2.46E+04 | 3.71E+04 | 2.07E+04 | 1.84E+04 | 2.34E+04 | 2.50E+04 | -- | -- |
| mws1565 | 3.89E+02 | 2.09E+02 | 3.88E+02 | C_17_H_24_O_10_ | [M+H]+ | Geniposide | Terpenoids | Monoterpenoids | 24512-63-8 | 2 | 8.39E+03 | 1.89E+03 | 1.81E+04 | 6.91E+04 | 8.01E+04 | 5.80E+04 | 5.25E+03 | 1.97E+04 | 2.54E+04 | 1.95E+04 | 1.03E+04 | 5.97E+03 | 3.31E+04 | 3.08E+04 | 2.82E+04 | C09781 | -- |
| Lmzn001582 | 3.87E+02 | 2.07E+02 | 3.88E+02 | C_18_H_28_O_9_ | [M-H]- | 5'-Glucosyloxyjasmanic acid | Phenolic acids | Phenolic acids | - | 3 | 3.27E+04 | 3.72E+04 | 4.33E+04 | 8.91E+04 | 8.49E+04 | 9.50E+04 | 1.13E+05 | 1.02E+05 | 8.55E+04 | 9.43E+04 | 1.11E+05 | 1.18E+05 | 7.49E+04 | 6.44E+04 | 1.09E+05 | -- | -- |
| Lmwp011196 | 3.91E+02 | 1.49E+02 | 3.90E+02 | C_24_H_38_O_4_ | [M+H]+ | Bis(2-ethylhexyl)phthalate | Phenolic acids | Phenolic acids | 117-81-7 | 2 | 1.64E+06 | 1.52E+06 | 1.56E+06 | 1.61E+06 | 2.05E+06 | 2.10E+06 | 1.92E+06 | 1.56E+06 | 1.59E+06 | 1.89E+06 | 1.78E+06 | 1.83E+06 | 5.14E+05 | 5.47E+05 | 6.52E+05 | C03690 | -- |
| Smhp004654 | 3.93E+02 | 2.31E+02 | 3.92E+02 | C_19_H_20_O_9_ | [M+H]+ | 2,4,6-Trihydroxy-styrene-6-O-β-D-glucopyranose | Phenolic acids | Phenolic acids | - | 2 | 2.70E+04 | 1.79E+04 | 5.73E+04 | 4.42E+04 | 7.22E+04 | 4.75E+04 | 1.48E+04 | 1.48E+04 | 1.52E+04 | 1.58E+04 | 1.12E+04 | 1.81E+04 | 4.48E+04 | 3.09E+04 | 2.50E+04 | -- | -- |
| pmp000602 | 3.95E+02 | 2.33E+02 | 3.94E+02 | C_19_H_22_O_9_ | [M+H]+ | 6-Hydroxymusizin-8-O-β-D-glucoside | Others | Others | - | 2 | 5.58E+04 | 7.29E+04 | 5.91E+04 | 5.97E+04 | 7.13E+04 | 5.89E+04 | 6.47E+04 | 4.41E+04 | 6.39E+04 | 3.24E+04 | 6.01E+04 | 4.88E+04 | 5.79E+04 | 6.04E+04 | 7.26E+04 | -- | -- |
| Hmap008483 | 4.01E+02 | 1.63E+02 | 4.00E+02 | C_24_H_32_O_5_ | [M+H]+ | Ferukrin | Lignans and Coumarins | Coumarins | 62742-67-0 | 3 | 1.28E+04 | 1.57E+04 | 1.25E+04 | 8.73E+03 | 1.09E+04 | 9.96E+03 | 2.67E+04 | 2.72E+04 | 3.94E+04 | 1.85E+04 | 1.20E+04 | 2.40E+04 | 2.39E+04 | 2.55E+04 | 1.88E+04 | -- | -- |
| mws0043 | 4.03E+02 | 3.73E+02 | 4.02E+02 | C_21_H_22_O_8_ | [M+H]+ | Nobiletin (5,6,7,8,3',4'-Hexamethoxyflavone) | Flavonoids | Flavones | 478-01-3 | 3 | 2.71E+04 | 2.83E+04 | 3.03E+04 | 2.32E+04 | 2.91E+04 | 2.59E+04 | 3.02E+04 | 1.58E+04 | 1.52E+04 | 7.43E+03 | 1.67E+04 | 1.80E+04 | 1.92E+04 | 2.43E+04 | 1.71E+04 | C10112 | -- |
| Hmln003529 | 4.01E+02 | 2.69E+02 | 4.02E+02 | C_18_H_26_O_10_ | [M-H]- | Benzyl β-primeveroside | Phenolic acids | Phenolic acids | 130622-31-0 | 1 | 1.57E+06 | 9.40E+05 | 1.57E+06 | 1.64E+06 | 1.41E+06 | 1.83E+06 | 9.52E+05 | 7.80E+05 | 8.80E+05 | 8.04E+05 | 1.17E+06 | 9.68E+05 | 1.24E+06 | 1.29E+06 | 1.33E+06 | -- | -- |
| Cmjn004337 | 4.01E+02 | 2.69E+02 | 4.02E+02 | C_18_H_26_O_10_ | [M-H]- | Benzyl-(2''-O-xylosyl)glucoside | Phenolic acids | Phenolic acids | - | 1 | 1.10E+06 | 1.16E+06 | 8.02E+05 | 1.38E+06 | 1.86E+06 | 1.83E+06 | 7.56E+05 | 6.01E+05 | 9.52E+05 | 8.34E+05 | 8.42E+05 | 8.74E+05 | 9.61E+05 | 1.37E+06 | 1.29E+06 | -- | -- |
| pme3007 | 4.03E+02 | 1.59E+02 | 4.04E+02 | C_9_H_14_N_2_O_12_P_2_ | [M-H]- | Uridine 5'-diphosphate | Nucleotides and derivatives | Nucleotides and derivatives | 27821-45-0 | 3 | 1.09E+04 | 1.04E+04 | 1.11E+04 | 1.10E+05 | 1.15E+05 | 1.14E+05 | 4.62E+04 | 4.05E+04 | 4.63E+04 | 7.79E+04 | 7.53E+04 | 8.06E+04 | 5.16E+04 | 5.82E+04 | 6.90E+04 | C00015 | ko00240,ko00908,ko01100,ko01240 |
| pmp000679 | 4.22E+02 | 2.43E+02 | 4.04E+02 | C_17_H_24_O_11_ | [M+NH4]+ | 11-methyl-forsythide | Terpenoids | Monoterpenoids | 159598-00-2 | 2 | 2.60E+04 | 3.35E+04 | 3.95E+04 | 5.08E+04 | 3.31E+04 | 4.55E+04 | 9.66E+04 | 7.62E+04 | 9.47E+04 | 1.02E+05 | 1.08E+05 | 1.13E+05 | 5.14E+04 | 6.22E+04 | 4.46E+04 | -- | -- |
| pmp001054 | 4.22E+02 | 2.25E+02 | 4.04E+02 | C_17_H_24_O_11_ | [M+NH4]+ | Gardenoside | Terpenoids | Monoterpenoids | 24512-62-7 | 2 | 9.26E+03 | 3.64E+03 | 1.96E+04 | 3.25E+04 | 4.13E+04 | 2.11E+04 | 2.57E+04 | 1.62E+04 | 2.65E+04 | 1.83E+04 | 1.47E+04 | 2.52E+04 | 2.15E+04 | 3.09E+04 | 1.78E+04 | C09779 | -- |
| Lmjp003402 | 4.05E+02 | 3.87E+02 | 4.04E+02 | C_21_H_24_O_8_ | [M+H]+ | 2'-Hydroxy-3,4,5,3',4',6'-hexameth-oxychalcone | Flavonoids | Chalcones | - | 2 | 2.39E+03 | 3.86E+03 | 2.55E+03 | 2.25E+04 | 1.52E+04 | 2.14E+04 | 8.13E+03 | 6.12E+03 | 1.05E+04 | 1.33E+04 | 1.09E+04 | 1.42E+04 | 9.39E+03 | 7.35E+03 | 1.07E+04 | -- | -- |
| MWSmce489 | 4.07E+02 | 2.45E+02 | 4.06E+02 | C_20_H_22_O_9_ | [M+H]+ | 2,3,5,4'-Tetrahydroxystilbene-2-O-glucoside | Others | Stilbene | 82373-94-2 | 2 | 5.63E+04 | 4.88E+04 | 4.78E+04 | 3.63E+04 | 1.92E+04 | 1.78E+04 | 3.10E+04 | 1.27E+04 | 3.04E+04 | 7.69E+03 | 1.36E+04 | 1.64E+04 | 2.99E+04 | 2.72E+04 | 3.31E+04 | -- | -- |
| MWSslk186 | 4.05E+02 | 1.65E+02 | 4.06E+02 | C_17_H_26_O_11_ | [M-H]- | 8-O-Acetylharpagide | Terpenoids | Monoterpenoids | 6926-14-3 | 2 | 9.74E+03 | 9.17E+03 | 9.73E+03 | 8.70E+03 | 1.33E+04 | 1.20E+04 | 9.00E+00 | 9.00E+00 | 9.00E+00 | 9.00E+00 | 9.00E+00 | 9.00E+00 | 6.21E+03 | 8.90E+03 | 1.12E+04 | -- | -- |
| Zmdn005516 | 4.07E+02 | 2.45E+02 | 4.08E+02 | C_20_H_24_O_9_ | [M-H]- | Torachrysone-8-O-glucoside | Phenolic acids | Phenolic acids | 64032-49-1 | 2 | 6.09E+05 | 6.39E+05 | 5.70E+05 | 3.55E+05 | 3.69E+05 | 3.66E+05 | 2.86E+05 | 2.55E+05 | 2.76E+05 | 2.42E+05 | 2.73E+05 | 2.87E+05 | 3.79E+05 | 4.18E+05 | 4.68E+05 | -- | -- |
| Zmbp004146 | 4.09E+02 | 2.47E+02 | 4.08E+02 | C_20_H_24_O_9_ | [M+H]+ | Praeroside IV | Lignans and Coumarins | Coumarins | - | 2 | 2.27E+06 | 2.17E+06 | 2.07E+06 | 3.28E+06 | 2.91E+06 | 2.90E+06 | 3.36E+06 | 3.12E+06 | 3.30E+06 | 2.98E+06 | 3.02E+06 | 3.48E+06 | 2.71E+06 | 2.92E+06 | 3.00E+06 | -- | -- |
| MWSslk171 | 4.07E+02 | 2.27E+02 | 4.08E+02 | C_20_H_24_O_9_ | [M-H]- | Nodakenin | Lignans and Coumarins | Coumarins | 495-31-8 | 1 | 3.55E+06 | 2.59E+06 | 3.12E+06 | 4.79E+06 | 4.25E+06 | 4.68E+06 | 4.33E+06 | 3.94E+06 | 4.20E+06 | 3.93E+06 | 4.10E+06 | 3.95E+06 | 3.87E+06 | 4.19E+06 | 4.33E+06 | C09279 | -- |
| Zmsn001980 | 4.07E+02 | 2.03E+02 | 4.08E+02 | C_21_H_28_O_8_ | [M-H]- | Ixerisoside D | Terpenoids | Sesquiterpenoids | - | 2 | 2.97E+06 | 4.40E+06 | 4.15E+06 | 8.31E+06 | 9.03E+06 | 9.86E+06 | 2.03E+07 | 2.41E+07 | 2.37E+07 | 3.08E+07 | 2.83E+07 | 2.84E+07 | 1.37E+07 | 1.37E+07 | 1.42E+07 | -- | -- |
| pmb2165 | 4.12E+02 | 1.84E+02 | 4.11E+02 | C_18_H_38_NO_7_P | [M+H]+ | LysoPC 10:0 | Lipids | LPC | 22248-63-1 | 3 | 4.04E+04 | 4.25E+04 | 4.77E+04 | 9.00E+00 | 9.00E+00 | 9.00E+00 | 2.49E+03 | 4.94E+03 | 5.07E+03 | 9.00E+00 | 9.00E+00 | 9.00E+00 | 1.17E+04 | 9.65E+03 | 1.24E+04 | -- | -- |
| Hmhp002580 | 4.13E+02 | 1.36E+02 | 4.12E+02 | C_19_H_24_O_10_ | [M+H]+ | Methylpicraquassioside A | Lignans and Coumarins | Coumarins | - | 2 | 7.91E+04 | 6.34E+04 | 7.00E+04 | 1.07E+05 | 1.04E+05 | 8.66E+04 | 7.17E+04 | 6.00E+04 | 7.25E+04 | 7.67E+04 | 9.84E+04 | 9.82E+04 | 8.24E+04 | 8.49E+04 | 9.42E+04 | -- | -- |
| pme1587 | 4.17E+02 | 2.55E+02 | 4.16E+02 | C_21_H_20_O_9_ | [M+H]+ | Daidzein-7-O-glucoside(Daidzin) | Flavonoids | Isoflavones | 552-66-9 | 2 | 4.87E+04 | 4.14E+04 | 5.45E+04 | 4.84E+04 | 5.45E+04 | 4.98E+04 | 3.49E+04 | 3.99E+04 | 3.64E+04 | 3.61E+04 | 5.42E+04 | 2.83E+04 | 4.24E+04 | 4.71E+04 | 4.50E+04 | C10216 | ko00943 |
| pmp000417 | 4.17E+02 | 2.55E+02 | 4.16E+02 | C_21_H_20_O_9_ | [M+H]+ | Daidzein-4'-O-glucoside | Flavonoids | Isoflavones | - | 2 | 1.25E+05 | 1.08E+05 | 1.28E+05 | 8.09E+04 | 1.01E+05 | 7.41E+04 | 6.95E+04 | 4.50E+04 | 5.17E+04 | 4.60E+04 | 3.70E+04 | 3.98E+04 | 6.94E+04 | 6.01E+04 | 9.04E+04 | -- | -- |
| mws0071 | 4.15E+02 | 2.69E+02 | 4.16E+02 | C_21_H_20_O_9_ | [M-H]- | Apigenin-4'-O-rhamnoside | Flavonoids | Flavones | 133538-77-9 | 3 | 4.11E+04 | 3.74E+04 | 5.12E+04 | 9.00E+00 | 9.00E+00 | 9.00E+00 | 9.00E+00 | 9.00E+00 | 9.00E+00 | 9.00E+00 | 9.00E+00 | 9.00E+00 | 1.25E+04 | 1.28E+04 | 1.56E+04 | -- | -- |
| mws2523 | 4.21E+02 | 2.41E+02 | 4.22E+02 | C_12_H_23_O_14_P | [M-H]- | Trehalose 6-phosphate | Others | Saccharides and Alcohols | 4484-88-2 | 2 | 1.27E+05 | 1.12E+05 | 1.55E+05 | 7.24E+04 | 7.34E+04 | 6.77E+04 | 2.15E+05 | 2.24E+05 | 2.12E+05 | 9.64E+04 | 7.24E+04 | 9.82E+04 | 1.14E+05 | 1.38E+05 | 1.37E+05 | C00689 | ko00500,ko01100,ko01110 |
| Zmbp003590 | 4.25E+02 | 2.63E+02 | 4.24E+02 | C_20_H_24_O_10_ | [M+H]+ | Rutarin | Lignans and Coumarins | Coumarins | 20320-81-4 | 2 | 2.86E+07 | 3.30E+07 | 2.97E+07 | 4.46E+07 | 4.31E+07 | 4.12E+07 | 2.53E+07 | 2.78E+07 | 2.42E+07 | 3.00E+07 | 2.94E+07 | 2.85E+07 | 3.24E+07 | 3.28E+07 | 3.61E+07 | -- | -- |
| Hmap008864 | 4.25E+02 | 1.63E+02 | 4.24E+02 | C_26_H_32_O_5_ | [M+H]+ | badrakemin acetate | Lignans and Coumarins | Coumarins | - | 3 | 8.76E+04 | 8.19E+04 | 1.07E+05 | 9.89E+04 | 9.02E+04 | 9.03E+04 | 1.43E+05 | 1.52E+05 | 1.76E+05 | 1.61E+05 | 2.08E+05 | 1.76E+05 | 1.29E+05 | 1.67E+05 | 1.39E+05 | -- | -- |
| Hmcp005535 | 4.25E+02 | 1.91E+02 | 4.24E+02 | C_30_H_48_O | [M+H]+ | α-Amyrenone | Terpenoids | Triterpene | 638-96-0 | 3 | 9.00E+00 | 9.00E+00 | 9.00E+00 | 1.83E+04 | 2.26E+04 | 1.95E+04 | 9.00E+00 | 9.00E+00 | 9.00E+00 | 1.18E+04 | 1.13E+04 | 8.95E+03 | 1.05E+04 | 1.16E+04 | 1.11E+04 | -- | -- |
| pmb0864 | 4.26E+02 | 2.85E+02 | 4.25E+02 | C_19_H_40_NO_7_P | [M+H]+ | LysoPE 14:0 | Lipids | LPE | - | 1 | 4.68E+04 | 5.12E+04 | 4.62E+04 | 4.77E+04 | 4.85E+04 | 4.44E+04 | 6.12E+04 | 5.66E+04 | 7.12E+04 | 2.21E+04 | 2.64E+04 | 3.09E+04 | 4.13E+04 | 4.38E+04 | 5.20E+04 | -- | -- |
| Lmhp008337 | 4.26E+02 | 2.85E+02 | 4.25E+02 | C_19_H_40_NO_7_P | [M+H]+ | LysoPE 14:0(2n isomer) | Lipids | LPE | - | 1 | 1.34E+04 | 1.19E+04 | 1.15E+04 | 8.54E+03 | 1.04E+04 | 8.71E+03 | 7.53E+04 | 7.71E+04 | 9.04E+04 | 6.28E+03 | 4.53E+03 | 2.85E+03 | 2.51E+04 | 2.93E+04 | 2.46E+04 | -- | -- |
| mad1424 | 4.27E+02 | 2.95E+02 | 4.26E+02 | C_17_H_14_O_13_ | [M+H]+ | Ditartaroyl-hydroxycoumarin | Lignans and Coumarins | Coumarins | - | 3 | 1.19E+05 | 1.14E+05 | 1.09E+05 | 1.74E+05 | 1.88E+05 | 1.76E+05 | 1.10E+05 | 9.68E+04 | 1.09E+05 | 1.40E+05 | 1.33E+05 | 1.28E+05 | 1.49E+05 | 1.32E+05 | 1.51E+05 | -- | -- |
| Zmbp003916 | 4.27E+02 | 2.47E+02 | 4.26E+02 | C_20_H_26_O_10_ | [M+H]+ | Praeroside VI | Lignans and Coumarins | Coumarins | - | 2 | 4.20E+04 | 6.88E+04 | 4.80E+04 | 6.06E+04 | 7.94E+04 | 6.59E+04 | 5.29E+04 | 5.51E+04 | 3.02E+04 | 5.23E+04 | 5.48E+04 | 5.23E+04 | 4.63E+04 | 6.78E+04 | 5.58E+04 | -- | -- |
| Cmjp002035 | 4.27E+02 | 2.47E+02 | 4.26E+02 | C_21_H_30_O_9_ | [M+H]+ | Sonchuside E | Terpenoids | Sesquiterpenoids | - | 2 | 8.89E+05 | 8.68E+05 | 8.89E+05 | 9.54E+05 | 8.42E+05 | 1.03E+06 | 7.55E+05 | 8.36E+05 | 7.79E+05 | 1.08E+06 | 1.79E+06 | 4.83E+05 | 4.79E+05 | 8.31E+05 | 1.24E+06 | -- | -- |
| pme2117 | 4.26E+02 | 1.59E+02 | 4.27E+02 | C_10_H_15_N_5_O_10_P_2_ | [M-H]- | Adenosine 5'-diphosphate | Nucleotides and derivatives | Nucleotides and derivatives | 58-64-0 | 3 | 2.98E+04 | 3.30E+04 | 3.67E+04 | 2.57E+05 | 2.25E+05 | 2.03E+05 | 6.45E+04 | 7.00E+04 | 5.91E+04 | 1.71E+05 | 1.49E+05 | 1.84E+05 | 1.04E+05 | 1.37E+05 | 1.17E+05 | C00008 | ko00190,ko00195,ko00230,ko00908,ko01100,ko01110,ko01240 |
| Zmbp012004 | 4.29E+02 | 2.45E+02 | 4.28E+02 | C_24_H_28_O_7_ | [M+H]+ | Qianhucoumarin H | Lignans and Coumarins | Coumarins | - | 3 | 4.95E+03 | 5.92E+03 | 5.38E+03 | 3.96E+03 | 6.76E+03 | 6.62E+03 | 7.09E+03 | 1.14E+04 | 8.40E+03 | 5.58E+03 | 3.98E+03 | 4.41E+03 | 6.78E+03 | 7.63E+03 | 6.21E+03 | -- | -- |
| Zmbp011794 | 4.29E+02 | 2.45E+02 | 4.28E+02 | C_24_H_28_O_7_ | [M+H]+ | Praeruptorin E | Lignans and Coumarins | Coumarins | - | 3 | 8.36E+03 | 5.59E+03 | 6.87E+03 | 5.46E+03 | 6.87E+03 | 5.31E+03 | 1.02E+04 | 8.23E+03 | 8.47E+03 | 4.75E+03 | 6.00E+03 | 3.73E+03 | 6.39E+03 | 9.38E+03 | 6.30E+03 | -- | -- |
| Zmsn003589 | 4.27E+02 | 1.53E+02 | 4.28E+02 | C_21_H_32_O_9_ | [M-H]- | Lactuside B | Others | Others | 106009-42-1 | 2 | 5.74E+05 | 4.18E+05 | 6.56E+05 | 9.11E+05 | 9.46E+05 | 8.31E+05 | 6.91E+05 | 7.69E+05 | 6.15E+05 | 7.63E+05 | 8.05E+05 | 8.06E+05 | 7.16E+05 | 7.46E+05 | 8.29E+05 | -- | -- |
| Lmhn002574 | 4.31E+02 | 1.37E+02 | 4.32E+02 | C_20_H_16_O_11_ | [M-H]- | Caffeoyl(p-Hydroxybenzoyl)tartaric acid | Phenolic acids | Phenolic acids | - | 3 | 9.00E+00 | 9.00E+00 | 9.00E+00 | 5.07E+04 | 5.66E+04 | 5.00E+04 | 9.00E+00 | 9.00E+00 | 9.00E+00 | 9.00E+00 | 9.00E+00 | 9.00E+00 | 1.55E+04 | 1.83E+04 | 1.39E+04 | -- | -- |
| Lmgp004731 | 4.33E+02 | 2.71E+02 | 4.32E+02 | C_21_H_20_O_10_ | [M+H]+ | Genistein-7-O-galactoside | Flavonoids | Isoflavones | - | 3 | 1.22E+04 | 2.07E+03 | 3.11E+03 | 1.89E+04 | 1.22E+04 | 1.34E+04 | 1.25E+03 | 7.06E+03 | 7.73E+03 | 9.75E+03 | 6.08E+03 | 8.94E+03 | 1.10E+04 | 1.05E+04 | 8.93E+03 | -- | -- |
| mws0072 | 4.33E+02 | 2.71E+02 | 4.32E+02 | C_21_H_20_O_10_ | [M+H]+ | Apigenin-5-O-glucoside | Flavonoids | Flavones | 28757-27-9 | 3 | 6.89E+04 | 6.33E+04 | 6.93E+04 | 8.46E+04 | 9.06E+04 | 5.71E+04 | 3.13E+04 | 3.00E+04 | 4.21E+04 | 3.44E+04 | 4.17E+04 | 5.05E+04 | 4.51E+04 | 6.12E+04 | 4.89E+04 | -- | -- |
| HJN088 | 4.31E+02 | 1.25E+02 | 4.32E+02 | C_18_H_24_O_12_ | [M-H]- | Licoagroside B | Others | Others | - | 2 | 2.30E+04 | 1.90E+04 | 2.33E+04 | 3.00E+04 | 2.66E+04 | 2.94E+04 | 2.99E+04 | 2.63E+04 | 3.37E+04 | 3.36E+04 | 2.66E+04 | 3.55E+04 | 2.65E+04 | 3.11E+04 | 3.37E+04 | -- | -- |
| mws2118 | 4.35E+02 | 1.67E+02 | 4.36E+02 | C_21_H_24_O_10_ | [M-H]- | Phloretin-2'-O-glucoside (Phlorizin) | Flavonoids | Chalcones | 60-81-1 | 3 | 1.10E+04 | 8.29E+03 | 1.06E+04 | 2.11E+04 | 1.97E+04 | 2.17E+04 | 1.89E+04 | 2.21E+04 | 2.11E+04 | 1.99E+04 | 2.24E+04 | 2.22E+04 | 2.16E+04 | 2.55E+04 | 2.04E+04 | C01604 | ko00941,ko01110 |
| Lmhp008440 | 4.38E+02 | 2.97E+02 | 4.37E+02 | C_20_H_40_NO_7_P | [M+H]+ | LysoPE 15:1 | Lipids | LPE | - | 3 | 5.26E+04 | 4.68E+04 | 4.68E+04 | 3.48E+04 | 3.52E+04 | 3.46E+04 | 4.99E+04 | 4.22E+04 | 4.60E+04 | 2.03E+04 | 2.62E+04 | 2.66E+04 | 3.96E+04 | 4.01E+04 | 4.05E+04 | -- | -- |
| Lmhp008885 | 4.40E+02 | 2.99E+02 | 4.39E+02 | C_20_H_42_NO_7_P | [M+H]+ | LysoPE 15:0(2n isomer) | Lipids | LPE | - | 1 | 1.81E+05 | 1.83E+05 | 1.21E+05 | 5.31E+04 | 5.97E+04 | 5.09E+04 | 7.43E+04 | 7.35E+04 | 7.15E+04 | 1.82E+04 | 1.27E+04 | 1.81E+04 | 7.49E+04 | 5.39E+04 | 7.95E+04 | -- | -- |
| pmb0862 | 4.40E+02 | 1.84E+02 | 4.39E+02 | C_20_H_42_NO_7_P | [M+H]+ | LysoPC 12:0 | Lipids | LPC | 20559-18-6 | 3 | 2.87E+05 | 2.89E+05 | 3.05E+05 | 5.70E+03 | 8.28E+03 | 8.27E+03 | 4.23E+04 | 3.59E+04 | 3.94E+04 | 8.03E+03 | 8.28E+03 | 6.72E+03 | 9.20E+04 | 9.22E+04 | 9.07E+04 | -- | -- |
| Lmhp009187 | 4.40E+02 | 2.99E+02 | 4.39E+02 | C_20_H_42_NO_7_P | [M+H]+ | LysoPE 15:0 | Lipids | LPE | - | 1 | 1.44E+05 | 1.78E+05 | 1.71E+05 | 3.56E+05 | 3.24E+05 | 3.40E+05 | 2.46E+05 | 2.58E+05 | 2.89E+05 | 1.36E+05 | 1.65E+05 | 1.32E+05 | 2.60E+05 | 2.25E+05 | 2.63E+05 | -- | -- |
| pme1014 | 4.45E+02 | 3.41E+02 | 4.44E+02 | C_31_H_40_O_2_ | [M+H]+ | Menatetrenone (Vitamin K2) | Others | Vitamin | 11032-49-8 | 3 | 6.36E+04 | 4.54E+04 | 6.55E+04 | 2.90E+04 | 4.60E+04 | 3.74E+04 | 4.76E+04 | 6.48E+04 | 6.12E+04 | 5.44E+04 | 6.51E+04 | 6.17E+04 | 5.55E+06 | 5.13E+06 | 6.45E+06 | -- | -- |
| mws0052 | 4.47E+02 | 2.71E+02 | 4.46E+02 | C_21_H_18_O_11_ | [M+H]+ | Baicalin | Flavonoids | Flavones | 21967-41-9 | 3 | 1.07E+04 | 6.50E+03 | 1.13E+04 | 1.46E+04 | 7.09E+03 | 7.05E+03 | 1.51E+04 | 2.12E+04 | 2.45E+04 | 1.68E+04 | 1.57E+04 | 1.14E+04 | 1.19E+04 | 1.19E+04 | 1.30E+04 | C10025 | -- |
| mws0894 | 4.47E+02 | 2.85E+02 | 4.46E+02 | C_22_H_22_O_10_ | [M+H]+ | Glycitin | Flavonoids | Isoflavones | 40246-10-4 | 2 | 6.54E+04 | 3.73E+04 | 4.78E+04 | 9.43E+04 | 1.13E+05 | 9.87E+04 | 3.36E+04 | 5.97E+04 | 1.61E+04 | 5.71E+04 | 6.64E+04 | 6.51E+04 | 4.28E+04 | 4.64E+04 | 6.82E+04 | C16195 | ko00943 |
| Lmsp004915 | 4.49E+02 | 2.87E+02 | 4.48E+02 | C_21_H_20_O_11_ | [M+H]+ | Aureusidin-4-O-glucoside | Flavonoids | Aurones | - | 2 | 2.95E+05 | 2.62E+05 | 3.07E+05 | 7.29E+04 | 7.44E+04 | 7.04E+04 | 2.15E+05 | 2.99E+05 | 2.51E+05 | 1.07E+05 | 6.41E+04 | 7.37E+04 | 1.14E+05 | 1.65E+05 | 1.87E+05 | -- | -- |
| pme2459 | 4.49E+02 | 2.87E+02 | 4.48E+02 | C_21_H_20_O_11_ | [M+H]+ | Luteolin-7-O-glucoside (Cynaroside) | Flavonoids | Flavones | 5373-11-5 | 3 | 2.03E+05 | 1.78E+05 | 2.14E+05 | 9.20E+04 | 1.05E+05 | 1.14E+05 | 1.42E+05 | 9.69E+04 | 7.95E+04 | 6.17E+04 | 5.01E+04 | 4.90E+04 | 1.17E+05 | 5.47E+04 | 9.98E+04 | C03951 | ko00944 |
| MWS20147 | 4.49E+02 | 2.87E+02 | 4.48E+02 | C_21_H_20_O_11_ | [M+H]+ | Luteolin-3'-O-glucoside | Flavonoids | Flavones | 5154-41-6 | 2 | 9.34E+05 | 1.06E+06 | 1.00E+06 | 2.30E+05 | 2.36E+05 | 2.08E+05 | 6.93E+05 | 7.17E+05 | 8.15E+05 | 2.74E+05 | 2.23E+05 | 2.71E+05 | 5.47E+05 | 6.05E+05 | 5.20E+05 | -- | -- |
| MWSHY0136 | 4.49E+02 | 2.87E+02 | 4.48E+02 | C_21_H_20_O_11_ | [M+H]+ | Kaempferol-3-O-glucoside (Astragalin) | Flavonoids | Flavonols | 480-10-4 | 2 | 2.29E+05 | 3.02E+05 | 3.04E+05 | 3.59E+04 | 5.50E+04 | 6.78E+04 | 2.09E+05 | 1.31E+05 | 2.80E+05 | 6.35E+04 | 6.33E+04 | 9.05E+04 | 1.66E+05 | 1.37E+05 | 1.85E+05 | C12249 | ko00944,ko01110 |
| Lmmn004625 | 4.49E+02 | 2.87E+02 | 4.50E+02 | C_21_H_22_O_11_ | [M-H]- | Dihydrokaempferol-7-O-glucoside | Flavonoids | Flavanonols | - | 3 | 2.11E+04 | 1.10E+04 | 1.22E+04 | 2.70E+04 | 2.20E+04 | 2.66E+04 | 1.11E+04 | 1.01E+04 | 1.63E+04 | 1.18E+04 | 1.31E+04 | 7.98E+03 | 1.13E+04 | 1.50E+04 | 1.89E+04 | -- | -- |
| Cmxp003975 | 4.51E+02 | 2.89E+02 | 4.50E+02 | C_21_H_22_O_11_ | [M+H]+ | Okanin-4'-O-glucoside(Marein) | Flavonoids | Chalcones | 535-96-6 | 2 | 6.51E+04 | 5.86E+04 | 6.72E+04 | 8.11E+04 | 7.15E+04 | 9.81E+04 | 8.70E+04 | 8.84E+04 | 8.93E+04 | 1.23E+05 | 1.27E+05 | 2.04E+05 | 7.56E+04 | 1.07E+05 | 1.04E+05 | -- | -- |
| MWS20145 | 4.51E+02 | 2.89E+02 | 4.50E+02 | C_21_H_22_O_11_ | [M+H]+ | eriodictyol 7-O-β-D-glucopyranoside | Flavonoids | Flavanones | - | 2 | 5.05E+04 | 7.17E+04 | 9.80E+04 | 7.45E+04 | 1.02E+05 | 6.55E+04 | 8.11E+04 | 9.44E+04 | 8.10E+04 | 1.19E+05 | 9.70E+04 | 1.86E+05 | 7.91E+04 | 7.68E+04 | 7.54E+04 | -- | -- |
| Lmlp005236 | 4.51E+02 | 2.89E+02 | 4.50E+02 | C_21_H_22_O_11_ | [M+H]+ | Dihydrokaempferol-3-O-glucoside | Flavonoids | Flavanonols | 1049-08-8 | 2 | 1.67E+04 | 8.58E+03 | 1.60E+04 | 2.85E+04 | 4.43E+04 | 4.00E+04 | 1.42E+04 | 2.04E+04 | 3.03E+04 | 6.19E+04 | 5.79E+04 | 5.37E+04 | 3.03E+04 | 3.55E+04 | 3.10E+04 | -- | -- |
| mws0057 | 4.51E+02 | 2.89E+02 | 4.50E+02 | C_21_H_22_O_11_ | [M+H]+ | Eriodictyol-7-O-glucoside | Flavonoids | Flavanones | 38965-51-4 | 3 | 1.55E+04 | 1.47E+04 | 2.02E+04 | 3.60E+04 | 2.96E+04 | 2.81E+04 | 3.00E+04 | 4.04E+04 | 1.98E+04 | 5.42E+04 | 4.60E+04 | 4.88E+04 | 2.54E+04 | 3.38E+04 | 3.10E+04 | -- | -- |
| pmn001351 | 4.49E+02 | 2.45E+02 | 4.50E+02 | C_22_H_26_O_10_ | [M-H]- | Torachrysone-8-O-(6''-acetyl)glucoside | Phenolic acids | Phenolic acids | - | 2 | 3.68E+05 | 2.43E+05 | 3.09E+05 | 5.61E+05 | 5.44E+05 | 6.84E+05 | 2.77E+05 | 2.32E+05 | 3.32E+05 | 4.36E+05 | 4.23E+05 | 5.75E+05 | 6.46E+05 | 5.20E+05 | 5.53E+05 | -- | -- |
| Lmhp008763 | 4.52E+02 | 3.11E+02 | 4.51E+02 | C_21_H_42_NO_7_P | [M+H]+ | LysoPE 16:1(2n isomer) | Lipids | LPE | - | 2 | 1.08E+05 | 1.10E+05 | 9.35E+04 | 1.34E+05 | 1.38E+05 | 1.17E+05 | 1.61E+05 | 1.43E+05 | 1.67E+05 | 8.84E+04 | 8.18E+04 | 1.04E+05 | 1.14E+05 | 1.42E+05 | 1.45E+05 | -- | -- |
| Lmhp009034 | 4.52E+02 | 3.11E+02 | 4.51E+02 | C_21_H_42_NO_7_P | [M+H]+ | LysoPE 16:1 | Lipids | LPE | - | 2 | 8.68E+04 | 7.20E+04 | 8.78E+04 | 1.16E+05 | 7.47E+04 | 7.78E+04 | 8.08E+04 | 1.05E+05 | 9.78E+04 | 5.99E+04 | 5.39E+04 | 6.73E+04 | 8.03E+04 | 8.75E+04 | 1.05E+05 | -- | -- |
| HJN041 | 4.51E+02 | 2.89E+02 | 4.52E+02 | C_21_H_24_O_11_ | [M-H]- | Epicatechin glucoside | Flavonoids | Flavanols | - | 3 | 1.58E+04 | 1.71E+04 | 1.64E+04 | 2.00E+04 | 2.38E+04 | 2.85E+04 | 2.76E+04 | 2.15E+04 | 2.55E+04 | 2.48E+04 | 3.67E+04 | 2.28E+04 | 2.05E+04 | 1.93E+04 | 2.66E+04 | -- | -- |
| Zmhn001257 | 4.51E+02 | 2.89E+02 | 4.52E+02 | C_21_H_24_O_11_ | [M-H]- | Catechin-5-O-glucoside | Flavonoids | Flavanols | - | 2 | 1.61E+06 | 1.39E+06 | 1.72E+06 | 1.92E+04 | 2.05E+04 | 1.77E+04 | 1.37E+05 | 1.50E+05 | 1.67E+05 | 3.43E+04 | 3.30E+04 | 3.33E+04 | 4.63E+05 | 5.28E+05 | 5.76E+05 | -- | -- |
| pmb0876 | 4.54E+02 | 3.13E+02 | 4.53E+02 | C_21_H_44_NO_7_P | [M+H]+ | LysoPE 16:0 | Lipids | LPE | 53862-35-4 | 1 | 7.02E+06 | 7.12E+06 | 6.58E+06 | 1.66E+07 | 1.74E+07 | 1.70E+07 | 1.07E+07 | 1.03E+07 | 1.05E+07 | 7.28E+06 | 8.37E+06 | 7.80E+06 | 9.75E+06 | 1.09E+07 | 1.14E+07 | -- | -- |
| pmd0160 | 4.54E+02 | 3.13E+02 | 4.53E+02 | C_21_H_44_NO_7_P | [M+H]+ | LysoPE 16:0(2n isomer) | Lipids | LPE | - | 1 | 2.39E+06 | 2.31E+06 | 2.45E+06 | 3.16E+06 | 3.06E+06 | 3.21E+06 | 3.05E+06 | 3.18E+06 | 3.16E+06 | 1.37E+06 | 1.56E+06 | 1.58E+06 | 2.69E+06 | 2.75E+06 | 3.13E+06 | -- | -- |
| MWS5083 | 4.55E+02 | 9.70E+01 | 4.56E+02 | C_17_H_21_N_4_O_9_P | [M-H]- | Flavin Single Nucleotide(FMN) | Nucleotides and derivatives | Nucleotides and derivatives | 6184-17-4 | 2 | 2.75E+04 | 2.39E+04 | 2.68E+04 | 6.91E+04 | 4.75E+04 | 6.31E+04 | 2.74E+04 | 3.86E+04 | 3.41E+04 | 1.57E+04 | 3.44E+04 | 3.36E+04 | 3.40E+04 | 3.64E+04 | 4.86E+04 | C00061 | ko00190,ko00740,ko01100,ko01110,ko01240 |
| HJN110 | 4.55E+02 | 4.55E+02 | 4.56E+02 | C_30_H_48_O_3_ | [M-H]- | Betulinic acid | Terpenoids | Triterpene | 472-15-1 | 1 | 5.80E+03 | 5.72E+03 | 7.82E+03 | 1.10E+04 | 9.93E+03 | 1.05E+04 | 6.71E+03 | 5.40E+03 | 6.82E+03 | 8.17E+03 | 9.64E+03 | 8.88E+03 | 1.12E+04 | 1.01E+04 | 8.94E+03 | C08619 | -- |
| pmn001700 | 4.55E+02 | 4.55E+02 | 4.56E+02 | C_30_H_48_O_3_ | [M-H]- | 24,30-Dihydroxy-12(13)-enolupinol | Terpenoids | Triterpene | - | 1 | 1.51E+04 | 1.53E+04 | 2.81E+04 | 3.52E+04 | 3.96E+04 | 3.96E+04 | 8.85E+03 | 1.25E+04 | 1.44E+04 | 2.74E+04 | 3.02E+04 | 2.51E+04 | 2.35E+04 | 1.99E+04 | 1.98E+04 | -- | -- |
| Lmmn009170 | 4.55E+02 | 4.55E+02 | 4.56E+02 | C_30_H_48_O_3_ | [M-H]- | Mangiferolic acid | Terpenoids | Triterpene | 4184-34-3 | 1 | 5.29E+03 | 7.10E+03 | 6.63E+03 | 3.59E+03 | 1.01E+04 | 5.85E+03 | 4.60E+03 | 2.05E+03 | 2.15E+03 | 8.57E+02 | 3.22E+03 | 1.93E+03 | 6.44E+03 | 6.56E+03 | 5.18E+03 | -- | -- |
| Lmdn006811 | 4.55E+02 | 4.55E+02 | 4.56E+02 | C_30_H_48_O_3_ | [M-H]- | Soyasapogenol E | Terpenoids | Triterpene | 6750-59-0 | 2 | 3.83E+03 | 5.41E+03 | 4.55E+03 | 9.66E+03 | 1.14E+04 | 8.17E+03 | 5.75E+03 | 5.62E+03 | 6.08E+03 | 6.99E+03 | 6.79E+03 | 9.01E+03 | 8.64E+03 | 5.57E+03 | 6.30E+03 | C17420 | -- |
| mws4053 | 4.55E+02 | 4.55E+02 | 4.56E+02 | C_30_H_48_O_3_ | [M-H]- | Ursolic acid | Terpenoids | Triterpene | 77-52-1 | 3 | 5.01E+03 | 7.70E+03 | 5.52E+03 | 3.84E+03 | 1.14E+04 | 3.63E+03 | 4.15E+03 | 2.55E+03 | 5.13E+03 | 1.85E+03 | 3.74E+02 | 3.55E+02 | 4.94E+03 | 5.58E+03 | 4.65E+03 | C08988 | -- |
| pmb0464 | 4.58E+02 | 1.16E+02 | 4.57E+02 | C_16_H_27_NO_14_ | [M+H]+ | L-Aspartic acid-O-diglucoside | Amino acids and derivatives | Amino acids and derivatives | - | 3 | 7.55E+06 | 6.33E+06 | 7.78E+06 | 4.79E+05 | 5.18E+05 | 5.59E+05 | 3.48E+06 | 3.07E+06 | 3.92E+06 | 1.91E+06 | 1.70E+06 | 1.34E+06 | 2.52E+06 | 3.39E+06 | 3.20E+06 | -- | -- |
| pmb2654 | 4.60E+02 | 1.18E+02 | 4.61E+02 | C_19_H_27_NO_12_ | [M-H]- | Anthranilate-1-O-Sophoroside | Phenolic acids | Phenolic acids | - | 3 | 1.57E+06 | 1.67E+06 | 1.79E+06 | 3.16E+06 | 3.25E+06 | 3.33E+06 | 6.27E+06 | 6.62E+06 | 6.38E+06 | 8.00E+06 | 8.32E+06 | 6.75E+06 | 4.33E+06 | 3.82E+06 | 5.00E+06 | -- | -- |
| pmp000579 | 4.63E+02 | 3.01E+02 | 4.62E+02 | C_22_H_22_O_11_ | [M+H]+ | Diosmetin-7-O-galactoside | Flavonoids | Flavanones | - | 3 | 1.86E+04 | 2.34E+04 | 2.02E+04 | 2.39E+04 | 2.87E+04 | 3.18E+04 | 3.21E+04 | 3.57E+04 | 4.80E+04 | 4.38E+04 | 5.84E+04 | 3.26E+04 | 4.34E+04 | 3.67E+04 | 3.36E+04 | -- | -- |
| Lmdp003171 | 4.63E+02 | 3.17E+02 | 4.62E+02 | C_22_H_22_O_11_ | [M+H]+ | Azalein (Azaleatin-3-O-rhamnoside) | Flavonoids | Flavonols | 29028-02-2 | 2 | 3.42E+04 | 2.93E+04 | 2.02E+04 | 2.71E+03 | 2.70E+03 | 4.75E+03 | 1.96E+04 | 1.81E+04 | 2.38E+04 | 6.16E+03 | 3.05E+03 | 8.44E+03 | 8.85E+03 | 1.56E+04 | 1.11E+04 | -- | -- |
| Lmjp003655 | 4.63E+02 | 3.01E+02 | 4.62E+02 | C_22_H_22_O_11_ | [M+H]+ | 6-C-MethylKaempferol-3-glucoside | Flavonoids | Flavones | - | 3 | 1.30E+04 | 2.03E+04 | 1.90E+04 | 3.04E+04 | 1.86E+04 | 2.55E+04 | 3.86E+04 | 3.20E+04 | 3.54E+04 | 3.04E+04 | 3.95E+04 | 3.63E+04 | 3.18E+04 | 2.89E+04 | 3.52E+04 | -- | -- |
| Hmgp002189 | 4.63E+02 | 3.01E+02 | 4.62E+02 | C_22_H_22_O_11_ | [M+H]+ | Hispidulin-7-O-Glucoside | Flavonoids | Flavones | 17680-84-1 | 3 | 2.35E+04 | 1.96E+04 | 1.47E+04 | 2.03E+04 | 2.55E+04 | 2.97E+04 | 2.93E+04 | 3.04E+04 | 3.47E+04 | 4.55E+04 | 6.77E+04 | 5.39E+04 | 4.11E+04 | 3.16E+04 | 3.88E+04 | C17762 | -- |
| mws0061 | 4.63E+02 | 3.00E+02 | 4.64E+02 | C_21_H_20_O_12_ | [M-H]- | Quercetin-3-O-galactoside (Hyperin)* | Flavonoids | Flavonols | 482-36-0 | 1 | 1.36E+04 | 4.46E+03 | 8.65E+03 | 5.48E+04 | 5.10E+04 | 6.08E+04 | 5.99E+04 | 4.40E+04 | 6.36E+04 | 3.81E+04 | 4.31E+04 | 3.84E+04 | 6.02E+04 | 5.21E+04 | 4.03E+04 | C10073 | -- |
| pmp001309 | 4.65E+02 | 3.03E+02 | 4.64E+02 | C_21_H_20_O_12_ | [M+H]+ | 6-Hydroxykaempferol-7-O-glucoside | Flavonoids | Flavonols | - | 3 | 2.63E+04 | 1.69E+04 | 9.11E+03 | 1.88E+04 | 1.18E+04 | 1.65E+04 | 1.07E+04 | 4.25E+03 | 7.71E+03 | 7.03E+03 | 5.59E+03 | 1.04E+04 | 1.04E+04 | 1.81E+04 | 8.58E+03 | -- | -- |
| mws0856 | 4.63E+02 | 3.01E+02 | 4.64E+02 | C_21_H_20_O_12_ | [M-H]- | Quercetin-4'-O-glucoside (Spiraeoside)* | Flavonoids | Flavonols | 20229-56-5 | 1 | 9.00E+00 | 9.00E+00 | 9.00E+00 | 4.52E+04 | 4.76E+04 | 4.09E+04 | 3.68E+04 | 2.73E+04 | 5.47E+04 | 3.07E+04 | 2.38E+04 | 4.12E+04 | 2.70E+04 | 3.82E+04 | 3.95E+04 | -- | -- |
| mws0091 | 4.63E+02 | 3.00E+02 | 4.64E+02 | C_21_H_20_O_12_ | [M-H]- | Quercetin-3-O-glucoside (Isoquercitrin)* | Flavonoids | Flavonols | 482-35-9 | 1 | 5.06E+03 | 6.78E+03 | 1.17E+04 | 4.93E+04 | 3.49E+04 | 3.03E+04 | 4.01E+04 | 2.95E+04 | 4.94E+04 | 1.53E+04 | 3.45E+04 | 2.22E+04 | 2.66E+04 | 3.50E+04 | 3.07E+04 | C05623 | ko00944,ko01100,ko01110 |
| Lmzp002365 | 4.65E+02 | 3.03E+02 | 4.64E+02 | C_22_H_24_O_11_ | [M+H]+ | Hesperetin-7-O-glucoside | Flavonoids | Flavanones | 31712-49-9 | 2 | 7.47E+03 | 1.53E+04 | 9.49E+03 | 2.93E+04 | 1.90E+04 | 1.53E+04 | 3.21E+04 | 1.40E+04 | 1.01E+04 | 1.87E+04 | 1.88E+04 | 2.79E+04 | 1.64E+04 | 1.04E+04 | 2.71E+04 | C16422 | ko00941 |
| pme1598 | 4.63E+02 | 3.01E+02 | 4.64E+02 | C_22_H_24_O_11_ | [M-H]- | Hesperetin-5-O-glucoside | Flavonoids | Flavanones | 69651-80-5 | 3 | 6.30E+03 | 1.27E+04 | 1.45E+04 | 3.72E+04 | 3.66E+04 | 5.54E+04 | 5.31E+04 | 3.39E+04 | 4.81E+04 | 2.35E+04 | 4.36E+04 | 3.09E+04 | 2.49E+04 | 4.06E+04 | 3.51E+04 | -- | -- |
| Lmhp009769 | 4.66E+02 | 3.25E+02 | 4.65E+02 | C_22_H_44_NO_7_P | [M+H]+ | LysoPE 17:1 | Lipids | LPE | - | 3 | 1.03E+05 | 4.69E+04 | 8.43E+04 | 3.62E+04 | 4.22E+04 | 3.37E+04 | 4.62E+04 | 7.96E+04 | 1.01E+05 | 3.77E+04 | 2.69E+04 | 3.33E+04 | 4.55E+04 | 2.73E+04 | 4.69E+04 | -- | -- |
| Lmhp009464 | 4.66E+02 | 3.25E+02 | 4.65E+02 | C_22_H_44_NO_7_P | [M+H]+ | LysoPE 17:1(2n isomer) | Lipids | LPE | - | 3 | 7.88E+04 | 4.63E+04 | 4.91E+04 | 4.00E+04 | 5.07E+04 | 5.77E+04 | 4.32E+05 | 4.35E+05 | 4.62E+05 | 3.63E+04 | 3.90E+04 | 2.47E+04 | 1.32E+05 | 1.25E+05 | 1.40E+05 | -- | -- |
| pmd0130 | 4.68E+02 | 1.84E+02 | 4.67E+02 | C_22_H_46_NO_7_P | [M+H]+ | LysoPC 14:0 | Lipids | LPC | 20559-16-4 | 1 | 2.80E+05 | 2.67E+05 | 2.59E+05 | 1.42E+05 | 1.66E+05 | 1.70E+05 | 1.78E+05 | 1.61E+05 | 1.84E+05 | 1.02E+05 | 1.36E+05 | 1.24E+05 | 1.69E+05 | 1.65E+05 | 1.70E+05 | -- | -- |
| Lmhp010162 | 4.68E+02 | 3.27E+02 | 4.67E+02 | C_22_H_46_NO_7_P | [M+H]+ | LysoPE 17:0 | Lipids | LPE | - | 2 | 3.97E+04 | 4.50E+04 | 4.62E+04 | 4.09E+04 | 4.67E+04 | 3.47E+04 | 3.31E+04 | 3.58E+04 | 3.97E+04 | 1.97E+04 | 2.46E+04 | 2.61E+04 | 2.88E+04 | 4.15E+04 | 3.76E+04 | -- | -- |
| pmp000309 | 4.69E+02 | 3.07E+02 | 4.68E+02 | C_22_H_28_O_11_ | [M+H]+ | Cimifugin-7-O-glucoside | Others | Others | 80681-45-4 | 3 | 3.09E+04 | 2.38E+04 | 8.88E+04 | 6.34E+04 | 3.73E+04 | 6.74E+04 | 6.21E+04 | 4.33E+04 | 6.32E+04 | 8.05E+04 | 8.07E+04 | 9.48E+04 | 7.12E+04 | 5.12E+04 | 7.18E+04 | -- | -- |
| Cmbp003767 | 4.70E+02 | 1.31E+02 | 4.69E+02 | C_20_H_27_N_3_O_8_S | [M+H]+ | (E)-N5-(1-((Carboxymethyl)amino)-3-((3-(4-hydroxy-3-methoxyphenyl)allyl)thio)-1-oxopropan-2-yl)glutamine | Alkaloids | Alkaloids | - | 2 | 3.14E+04 | 2.77E+04 | 2.53E+04 | 1.37E+05 | 1.10E+05 | 1.20E+05 | 9.69E+05 | 7.62E+05 | 1.00E+06 | 1.95E+05 | 2.11E+05 | 1.82E+05 | 3.31E+05 | 2.70E+05 | 3.14E+05 | -- | -- |
| Lmzn006582 | 4.69E+02 | 4.69E+02 | 4.70E+02 | C_30_H_46_O_4_ | [M-H]- | Colubrinic acid | Terpenoids | Triterpene | - | 2 | 1.55E+04 | 1.38E+04 | 1.63E+04 | 1.67E+04 | 1.60E+04 | 1.55E+04 | 2.04E+04 | 2.12E+04 | 2.02E+04 | 1.55E+04 | 1.72E+04 | 1.73E+04 | 1.20E+04 | 1.22E+04 | 1.33E+04 | -- | -- |
| Hmgp002950 | 4.72E+02 | 2.20E+02 | 4.71E+02 | C_25_H_33_O_6_N_3_ | [M+H]+ | N1-Caffeoyl-N3-dihydrocaffeoylspermidine | Alkaloids | Phenolamine | - | 2 | 3.30E+04 | 2.60E+04 | 1.96E+04 | 3.67E+04 | 3.69E+04 | 3.29E+04 | 3.90E+04 | 3.51E+04 | 4.74E+04 | 6.38E+04 | 5.98E+04 | 4.65E+04 | 4.70E+04 | 2.87E+04 | 4.25E+04 | -- | -- |
| Zmpn008194 | 4.71E+02 | 4.71E+02 | 4.72E+02 | C_30_H_48_O_4_ | [M-H]- | Corosolic acid | Terpenoids | Triterpene | 4547-24-4 | 2 | 2.77E+05 | 2.73E+05 | 2.37E+05 | 4.34E+05 | 4.57E+05 | 4.29E+05 | 4.86E+04 | 5.71E+04 | 5.14E+04 | 5.43E+04 | 7.23E+04 | 6.51E+04 | 2.29E+05 | 2.38E+05 | 2.34E+05 | -- | -- |
| pmn001705 | 4.71E+02 | 4.71E+02 | 4.72E+02 | C_30_H_48_O_4_ | [M-H]- | 3,24-Dihydroxy-17,21-semiacetal-12(13)oleanolic fruit | Terpenoids | Triterpene | - | 2 | 4.67E+05 | 3.83E+05 | 4.84E+05 | 5.46E+05 | 6.21E+05 | 6.71E+05 | 4.31E+04 | 5.68E+04 | 4.24E+04 | 8.16E+04 | 9.05E+04 | 8.61E+04 | 3.12E+05 | 3.91E+05 | 3.26E+05 | -- | -- |
| pmn001706 | 4.71E+02 | 4.71E+02 | 4.72E+02 | C_30_H_48_O_4_ | [M-H]- | 2-Hydroxyoleanolic acid | Terpenoids | Triterpene | 26707-60-8 | 2 | 3.76E+05 | 5.30E+05 | 3.88E+05 | 5.86E+05 | 5.70E+05 | 5.81E+05 | 1.18E+05 | 8.46E+04 | 9.07E+04 | 7.27E+04 | 1.09E+05 | 1.18E+05 | 2.81E+05 | 2.99E+05 | 3.58E+05 | -- | -- |
| mws1610 | 4.71E+02 | 4.71E+02 | 4.72E+02 | C_30_H_48_O_4_ | [M-H]- | Maslinic acid | Terpenoids | Triterpene | 4373-41-5 | 3 | 4.33E+05 | 4.36E+05 | 3.91E+05 | 6.37E+05 | 6.63E+05 | 6.65E+05 | 8.25E+04 | 8.73E+04 | 7.89E+04 | 1.04E+05 | 1.21E+05 | 9.00E+04 | 3.36E+05 | 3.56E+05 | 3.78E+05 | C16939 | -- |
| Lmsn012425 | 4.71E+02 | 2.77E+02 | 4.72E+02 | C_30_H_48_O_4_ | [M-H]- | Rubusic acid (3β,7α-Dihydroxyolean-12-en-28-oic acid) | Terpenoids | Triterpene | 23984-26-1 | 3 | 1.37E+05 | 1.47E+05 | 1.35E+05 | 3.00E+05 | 2.86E+05 | 2.87E+05 | 4.95E+04 | 5.31E+04 | 6.14E+04 | 1.00E+05 | 1.01E+05 | 1.00E+05 | 1.56E+05 | 1.55E+05 | 1.69E+05 | -- | -- |
| Lmzn006284 | 4.71E+02 | 4.71E+02 | 4.72E+02 | C_30_H_48_O_4_ | [M-H]- | 2α-Hydroxyursolic acid | Terpenoids | Triterpene | - | 3 | 4.49E+05 | 3.72E+05 | 3.61E+05 | 5.87E+05 | 5.44E+05 | 5.95E+05 | 8.48E+04 | 6.57E+04 | 9.33E+04 | 7.22E+04 | 1.03E+05 | 6.35E+04 | 3.13E+05 | 3.58E+05 | 3.71E+05 | -- | -- |
| pmb1912 | 4.74E+02 | 3.27E+02 | 4.73E+02 | C_20_H_23_N_7_O_7_ | [M+H]+ | 10-Formyltetrahydrofolic Acid | Alkaloids | Alkaloids | 2800-34-2 | 2 | 5.16E+05 | 5.30E+05 | 5.00E+05 | 7.00E+05 | 5.63E+05 | 6.85E+05 | 4.40E+05 | 3.87E+05 | 3.87E+05 | 5.24E+05 | 4.08E+05 | 5.62E+05 | 4.75E+05 | 5.93E+05 | 5.86E+05 | C00234 | ko00670,ko00970,ko01100,ko01200,ko01240 |
| Lmhp008233 | 4.74E+02 | 3.33E+02 | 4.73E+02 | C_23_H_40_NO_7_P | [M+H]+ | LysoPE 18:4 | Lipids | LPE | - | 3 | 5.82E+03 | 5.93E+03 | 4.42E+03 | 5.39E+03 | 5.63E+03 | 3.80E+03 | 6.90E+03 | 4.15E+03 | 3.93E+03 | 3.36E+03 | 5.20E+03 | 4.26E+03 | 4.32E+03 | 5.92E+03 | 6.31E+03 | -- | -- |
| MWSmce621 | 4.73E+02 | 1.49E+02 | 4.74E+02 | C_22_H_18_O_12_ | [M-H]- | Cichoric Acid | Phenolic acids | Phenolic acids | 6537-80-0 | 2 | 1.44E+05 | 1.52E+05 | 1.28E+05 | 2.35E+05 | 1.82E+05 | 2.57E+05 | 1.27E+05 | 1.26E+05 | 1.38E+05 | 1.50E+05 | 1.23E+05 | 1.28E+05 | 1.11E+05 | 1.33E+05 | 1.87E+05 | C10437 | -- |
| Zmdn006148 | 4.73E+02 | 2.69E+02 | 4.74E+02 | C_23_H_22_O_11_ | [M-H]- | Emodin-8-O-(6'-acetyl)-glucoside | Quinones | Anthraquinone | - | 2 | 4.21E+04 | 5.96E+04 | 4.77E+04 | 1.03E+04 | 1.15E+04 | 7.97E+03 | 1.58E+04 | 1.51E+04 | 1.87E+04 | 2.61E+04 | 1.31E+04 | 2.00E+04 | 2.07E+04 | 2.41E+04 | 3.31E+04 | -- | -- |
| Lmtn001569 | 4.74E+02 | 3.12E+02 | 4.75E+02 | C_20_H_29_NO_12_ | [M-H]- | 2-Glucosyl-glucosyloxy-2-phenylacetic acid amide | Alkaloids | Alkaloids | - | 2 | 6.06E+05 | 6.49E+05 | 5.89E+05 | 9.68E+05 | 9.48E+05 | 9.65E+05 | 6.94E+05 | 6.69E+05 | 7.79E+05 | 7.04E+05 | 8.64E+05 | 8.50E+05 | 7.48E+05 | 8.29E+05 | 8.41E+05 | -- | -- |
| Lmhp008801 | 4.76E+02 | 3.35E+02 | 4.75E+02 | C_23_H_42_NO_7_P | [M+H]+ | LysoPE 18:3 | Lipids | LPE | - | 1 | 1.12E+05 | 7.87E+04 | 8.91E+04 | 1.37E+05 | 1.17E+05 | 1.21E+05 | 1.41E+05 | 1.31E+05 | 1.48E+05 | 1.08E+05 | 1.23E+05 | 8.74E+04 | 1.27E+05 | 1.28E+05 | 1.37E+05 | -- | -- |
| Lmhp008589 | 4.76E+02 | 3.35E+02 | 4.75E+02 | C_23_H_42_NO_7_P | [M+H]+ | LysoPE 18:3(2n isomer) | Lipids | LPE | - | 1 | 7.39E+04 | 6.81E+04 | 6.70E+04 | 7.02E+04 | 6.73E+04 | 1.01E+05 | 8.27E+04 | 8.86E+04 | 1.02E+05 | 5.47E+04 | 6.05E+04 | 6.56E+04 | 7.14E+04 | 7.15E+04 | 8.79E+04 | -- | -- |
| pmb0881 | 4.78E+02 | 3.37E+02 | 4.77E+02 | C_23_H_44_NO_7_P | [M+H]+ | LysoPE 18:2 | Lipids | LPE | - | 2 | 4.04E+06 | 3.85E+06 | 3.92E+06 | 4.07E+06 | 4.66E+06 | 4.02E+06 | 4.35E+06 | 5.03E+06 | 5.48E+06 | 3.38E+06 | 3.66E+06 | 4.99E+06 | 4.36E+06 | 5.07E+06 | 4.82E+06 | -- | -- |
| pmb0874 | 4.78E+02 | 3.37E+02 | 4.77E+02 | C_23_H_44_NO_7_P | [M+H]+ | LysoPE 18:2(2n isomer) | Lipids | LPE | - | 2 | 4.13E+06 | 3.75E+06 | 4.09E+06 | 5.82E+06 | 5.93E+06 | 5.92E+06 | 4.86E+06 | 5.24E+06 | 5.49E+06 | 4.23E+06 | 4.34E+06 | 4.93E+06 | 4.93E+06 | 5.13E+06 | 5.47E+06 | -- | -- |
| pme3391 | 4.79E+02 | 3.17E+02 | 4.79E+02 | C_22_H_23_O_12_+ | [M]+ | Petunidin-3-O-glucoside | Flavonoids | Anthocyanidins | 6988-81-4 | 3 | 4.64E+04 | 3.93E+04 | 4.49E+04 | 5.35E+04 | 6.04E+04 | 5.80E+04 | 5.49E+04 | 8.18E+04 | 2.89E+04 | 6.56E+04 | 2.76E+04 | 2.11E+04 | 6.30E+04 | 5.04E+04 | 4.44E+04 | C12139 | ko00942 |
| pmb0856 | 4.80E+02 | 3.39E+02 | 4.79E+02 | C_23_H_46_NO_7_P | [M+H]+ | LysoPE 18:1(2n isomer) | Lipids | LPE | - | 3 | 4.78E+05 | 4.59E+05 | 4.94E+05 | 8.44E+05 | 6.58E+05 | 7.75E+05 | 5.53E+05 | 6.46E+05 | 7.30E+05 | 4.84E+05 | 4.68E+05 | 4.99E+05 | 6.10E+05 | 6.28E+05 | 6.75E+05 | -- | -- |
| mws0289 | 4.80E+02 | 3.39E+02 | 4.79E+02 | C_23_H_46_NO_7_P | [M+H]+ | LysoPE 18:1 | Lipids | LPE | 89576-29-4 | 3 | 7.61E+05 | 7.62E+05 | 7.73E+05 | 1.30E+06 | 1.17E+06 | 1.25E+06 | 1.04E+06 | 1.01E+06 | 1.11E+06 | 7.69E+05 | 8.52E+05 | 7.99E+05 | 9.49E+05 | 1.14E+06 | 1.11E+06 | -- | -- |
| pmb2260 | 4.80E+02 | 1.84E+02 | 4.79E+02 | C_23_H_46_NO_7_P | [M+H]+ | LysoPC 15:1 | Lipids | LPC | - | 2 | 9.11E+04 | 8.61E+04 | 8.33E+04 | 2.40E+04 | 2.95E+04 | 3.91E+04 | 6.29E+04 | 6.53E+04 | 6.55E+04 | 4.58E+04 | 4.99E+04 | 5.48E+04 | 5.06E+04 | 5.27E+04 | 5.12E+04 | -- | -- |
| Lmhp009129 | 4.82E+02 | 1.84E+02 | 4.81E+02 | C_23_H_48_NO_7_P | [M+H]+ | LysoPC 15:0(2n isomer) | Lipids | LPC | - | 2 | 3.75E+05 | 2.79E+05 | 2.99E+05 | 1.09E+05 | 1.39E+05 | 1.47E+05 | 2.63E+05 | 2.76E+05 | 2.52E+05 | 8.79E+04 | 1.24E+05 | 1.02E+05 | 1.80E+05 | 2.14E+05 | 1.94E+05 | -- | -- |
| pmb2319 | 4.82E+02 | 1.84E+02 | 4.81E+02 | C_23_H_48_NO_7_P | [M+H]+ | LysoPC 15:0 | Lipids | LPC | 108273-89-8 | 2 | 1.08E+06 | 9.97E+05 | 9.47E+05 | 7.09E+05 | 7.24E+05 | 7.88E+05 | 8.81E+05 | 8.35E+05 | 8.56E+05 | 4.66E+05 | 5.68E+05 | 5.08E+05 | 7.80E+05 | 7.34E+05 | 8.59E+05 | -- | -- |
| pmb0883 | 4.82E+02 | 3.41E+02 | 4.81E+02 | C_23_H_48_NO_7_P | [M+H]+ | LysoPE 18:0 | Lipids | LPE | 69747-55-3 | 3 | 4.66E+04 | 4.36E+04 | 4.73E+04 | 1.77E+05 | 1.88E+05 | 1.64E+05 | 8.72E+04 | 7.73E+04 | 8.77E+04 | 7.04E+04 | 9.38E+04 | 8.45E+04 | 9.77E+04 | 8.40E+04 | 9.77E+04 | -- | -- |
| Hmbp002498 | 4.87E+02 | 1.93E+02 | 4.86E+02 | C_21_H_26_O_13_ | [M+H]+ | Scopoletin-7-O-xylosyl(1→6)glucoside | Lignans and Coumarins | Coumarins | - | 1 | 9.64E+06 | 1.99E+07 | 1.33E+07 | 1.58E+07 | 1.06E+07 | 1.45E+07 | 3.12E+06 | 2.19E+06 | 1.98E+06 | 2.96E+06 | 2.80E+06 | 4.25E+06 | 9.01E+06 | 5.69E+06 | 1.29E+07 | -- | -- |
| pmb2723 | 4.85E+02 | 3.23E+02 | 4.86E+02 | C_21_H_26_O_13_ | [M-H]- | 4-Hydroxycoumarin di-glucoside | Lignans and Coumarins | Coumarins | - | 3 | 9.61E+05 | 9.30E+05 | 8.79E+05 | 1.28E+06 | 1.15E+06 | 1.05E+06 | 7.37E+05 | 8.31E+05 | 6.99E+05 | 1.47E+06 | 1.45E+06 | 1.47E+06 | 1.05E+06 | 1.06E+06 | 1.30E+06 | -- | -- |
| Lmmn000214 | 4.87E+02 | 3.41E+02 | 4.88E+02 | C_18_H_32_O_15_ | [M-H]- | Solatriose | Others | Saccharides and Alcohols | 528-40-5 | 3 | 6.41E+05 | 5.68E+05 | 6.38E+05 | 7.31E+05 | 7.93E+05 | 6.49E+05 | 1.42E+06 | 1.82E+06 | 1.59E+06 | 1.21E+06 | 1.18E+06 | 9.22E+05 | 9.31E+05 | 8.95E+05 | 1.02E+06 | -- | -- |
| Hmjn003948 | 4.87E+02 | 4.87E+02 | 4.88E+02 | C_30_H_48_O_5_ | [M-H]- | Madasiatic acid | Terpenoids | Triterpene | 26532-66-1 | 3 | 6.22E+03 | 1.33E+04 | 8.26E+03 | 2.00E+04 | 2.34E+04 | 2.28E+04 | 9.00E+00 | 9.00E+00 | 9.00E+00 | 9.00E+00 | 9.00E+00 | 9.00E+00 | 8.90E+03 | 1.04E+04 | 1.06E+04 | -- | -- |
| Lmmp003903 | 4.91E+02 | 2.87E+02 | 4.90E+02 | C_23_H_22_O_12_ | [M+H]+ | Kaempferol-3-O-(2''-acetyl)glucoside | Flavonoids | Flavonols | - | 3 | 4.21E+05 | 1.16E+06 | 8.06E+05 | 1.22E+06 | 6.66E+05 | 1.10E+06 | 7.63E+05 | 4.48E+05 | 8.05E+05 | 7.25E+05 | 9.85E+05 | 8.33E+05 | 1.07E+06 | 5.80E+05 | 7.26E+05 | -- | -- |
| pmp001142 | 4.92E+02 | 1.45E+02 | 4.91E+02 | C_24_H_29_NO_10_ | [M+H]+ | Cimicifugamide A | Alkaloids | Phenolamine | - | 3 | 1.51E+04 | 1.45E+04 | 2.73E+04 | 5.02E+04 | 4.60E+04 | 6.79E+04 | 3.82E+04 | 2.31E+04 | 2.66E+04 | 4.80E+04 | 2.84E+04 | 5.86E+04 | 4.28E+04 | 3.79E+04 | 4.31E+04 | -- | -- |
| pmb0863 | 4.92E+02 | 1.84E+02 | 4.91E+02 | C_24_H_46_NO_7_P | [M+H]+ | LysoPC 16:2(2n isomer) | Lipids | LPC | - | 3 | 4.69E+04 | 3.85E+04 | 4.24E+04 | 3.00E+04 | 2.68E+04 | 2.08E+04 | 3.97E+04 | 4.02E+04 | 5.02E+04 | 3.79E+04 | 3.66E+04 | 4.98E+04 | 3.63E+04 | 3.23E+04 | 3.77E+04 | -- | -- |
| pma1303 | 4.92E+02 | 1.84E+02 | 4.91E+02 | C_24_H_46_NO_7_P | [M+H]+ | LysoPC 16:2 | Lipids | LPC | - | 3 | 4.77E+04 | 4.71E+04 | 3.88E+04 | 2.93E+04 | 2.80E+04 | 2.57E+04 | 4.28E+04 | 4.23E+04 | 4.72E+04 | 3.35E+04 | 4.77E+04 | 4.08E+04 | 3.57E+04 | 2.89E+04 | 3.80E+04 | -- | -- |
| pme0444 | 4.93E+02 | 3.31E+02 | 4.93E+02 | C_23_H_25_O_12_+ | [M]+ | Malvidin-3-O-glucoside (Oenin) | Flavonoids | Anthocyanidins | 18470-06-9 | 3 | 2.06E+04 | 1.62E+04 | 2.17E+04 | 3.28E+04 | 2.87E+04 | 3.72E+04 | 1.34E+04 | 1.79E+04 | 2.26E+04 | 2.17E+04 | 2.68E+04 | 2.87E+04 | 2.29E+04 | 3.77E+04 | 2.05E+04 | C12140 | ko00942 |
| pme0443 | 4.93E+02 | 3.31E+02 | 4.93E+02 | C_23_H_25_O_12_+ | [M]+ | Malvidin-3-O-galactoside (Primulin) | Flavonoids | Anthocyanidins | 30113-37-2 | 3 | 1.59E+04 | 1.81E+04 | 8.99E+03 | 3.49E+04 | 2.62E+04 | 2.41E+04 | 1.18E+04 | 1.11E+04 | 7.62E+03 | 1.97E+04 | 1.38E+04 | 1.53E+04 | 2.38E+04 | 1.51E+04 | 1.78E+04 | -- | -- |
| Lmhp008833 | 4.94E+02 | 1.84E+02 | 4.93E+02 | C_24_H_48_NO_7_P | [M+H]+ | LysoPC 16:1(2n isomer) | Lipids | LPC | - | 1 | 6.05E+05 | 4.82E+05 | 5.13E+05 | 4.10E+05 | 4.50E+05 | 4.71E+05 | 4.40E+05 | 4.65E+05 | 4.64E+05 | 4.06E+05 | 5.11E+05 | 4.52E+05 | 4.37E+05 | 4.02E+05 | 4.77E+05 | -- | -- |
| pmp001270 | 4.94E+02 | 1.84E+02 | 4.93E+02 | C_24_H_48_NO_7_P | [M+H]+ | LysoPC 16:1 | Lipids | LPC | 76790-27-7 | 1 | 5.78E+05 | 5.24E+05 | 4.40E+05 | 3.76E+05 | 4.47E+05 | 4.22E+05 | 4.53E+05 | 4.50E+05 | 4.78E+05 | 4.01E+05 | 4.43E+05 | 4.43E+05 | 4.13E+05 | 4.19E+05 | 4.59E+05 | -- | -- |
| pmb0855 | 4.96E+02 | 1.84E+02 | 4.95E+02 | C_24_H_50_NO_7_P | [M+H]+ | LysoPC 16:0 | Lipids | LPC | 17364-16-8 | 1 | 3.48E+07 | 3.25E+07 | 3.23E+07 | 2.95E+07 | 3.28E+07 | 3.21E+07 | 3.72E+07 | 3.66E+07 | 4.11E+07 | 2.17E+07 | 2.47E+07 | 2.36E+07 | 3.10E+07 | 3.14E+07 | 3.15E+07 | -- | -- |
| pmd0132 | 4.96E+02 | 1.84E+02 | 4.95E+02 | C_24_H_50_NO_7_P | [M+H]+ | LysoPC 16:0(2n isomer) | Lipids | LPC | - | 1 | 6.87E+06 | 6.02E+06 | 6.11E+06 | 2.84E+06 | 3.18E+06 | 2.85E+06 | 6.09E+06 | 6.72E+06 | 6.96E+06 | 2.20E+06 | 2.27E+06 | 2.44E+06 | 4.11E+06 | 4.19E+06 | 4.53E+06 | -- | -- |
| Lmgp003989 | 4.99E+02 | 1.63E+02 | 4.98E+02 | C_25_H_22_O_11_ | [M+H]+ | Dicaffeoylshikimic acid | Phenolic acids | Phenolic acids | - | 1 | 6.83E+05 | 5.94E+05 | 3.94E+05 | 5.98E+06 | 3.63E+06 | 6.35E+06 | 4.88E+06 | 4.50E+06 | 4.17E+06 | 4.07E+06 | 2.99E+06 | 6.22E+06 | 4.11E+06 | 3.53E+06 | 3.02E+06 | -- | -- |
| pmb3064 | 4.99E+02 | 1.63E+02 | 5.00E+02 | C_22_H_28_O_13_ | [M-H]- | 3-O-p-Coumaroylquinic acid-O-glucoside | Phenolic acids | Phenolic acids | - | 3 | 5.88E+03 | 5.59E+03 | 8.77E+03 | 6.08E+03 | 8.64E+03 | 6.64E+03 | 4.98E+03 | 5.50E+03 | 8.17E+03 | 7.34E+03 | 8.53E+03 | 6.08E+03 | 1.12E+04 | 9.02E+03 | 1.03E+04 | -- | -- |
| pmp000193 | 5.03E+02 | 2.55E+02 | 5.02E+02 | C_24_H_22_O_12_ | [M+H]+ | 6''-O-Malonyldaidzin | Flavonoids | Isoflavones | 124590-31-4 | 2 | 1.63E+05 | 1.86E+05 | 1.61E+05 | 3.90E+05 | 4.20E+05 | 2.92E+05 | 1.24E+05 | 1.38E+05 | 1.17E+05 | 2.59E+05 | 2.33E+05 | 1.60E+05 | 2.23E+05 | 2.18E+05 | 1.98E+05 | C16191 | ko00943 |
| pmb3000 | 5.03E+02 | 3.41E+02 | 5.04E+02 | C_24_H_24_O_12_ | [M-H]- | Chrysoeriol-7-O-(6''-acetyl)glucoside | Flavonoids | Flavones | - | 2 | 8.81E+03 | 1.27E+04 | 2.06E+04 | 3.07E+04 | 3.44E+04 | 4.28E+04 | 1.18E+04 | 7.20E+03 | 8.55E+03 | 9.89E+03 | 8.54E+03 | 1.94E+04 | 2.14E+04 | 1.68E+04 | 2.66E+04 | -- | -- |
| mws1589 | 5.03E+02 | 1.79E+02 | 5.04E+02 | C_18_H_32_O_16_ | [M-H]- | D-Panose | Others | Saccharides and Alcohols | 33401-87-5 | 2 | 1.74E+06 | 1.40E+06 | 1.61E+06 | 2.12E+06 | 2.31E+06 | 1.99E+06 | 1.25E+06 | 1.30E+06 | 7.46E+05 | 1.54E+06 | 2.32E+06 | 1.32E+06 | 9.79E+05 | 1.90E+06 | 1.23E+06 | C00713 | -- |
| pme0500 | 5.03E+02 | 3.23E+02 | 5.04E+02 | C_18_H_32_O_16_ | [M-H]- | D-Melezitose | Others | Saccharides and Alcohols | 597-12-6 | 1 | 2.41E+06 | 3.06E+06 | 2.19E+06 | 3.05E+06 | 3.81E+06 | 4.39E+06 | 1.88E+06 | 2.79E+06 | 3.27E+06 | 3.71E+06 | 4.23E+06 | 3.09E+06 | 2.58E+06 | 2.84E+06 | 1.78E+06 | C08243 | -- |
| MWS0442 | 5.27E+02 | 3.65E+02 | 5.04E+02 | C_18_H_32_O_16_ | [M+Na]+ | Maltotriose | Others | Saccharides and Alcohols | 1109-28-0 | 1 | 3.38E+05 | 4.23E+05 | 3.65E+05 | 5.24E+05 | 4.81E+05 | 5.91E+05 | 4.60E+05 | 3.60E+05 | 2.92E+05 | 2.97E+05 | 3.17E+05 | 2.86E+05 | 4.01E+05 | 3.37E+05 | 3.65E+05 | C01835 | ko02010 |
| pme2125 | 5.03E+02 | 1.79E+02 | 5.04E+02 | C_18_H_32_O_16_ | [M-H]- | Raffinose | Others | Saccharides and Alcohols | 512-69-6 | 3 | 4.78E+06 | 4.32E+06 | 4.73E+06 | 6.85E+06 | 5.48E+06 | 6.10E+06 | 4.49E+06 | 4.23E+06 | 4.71E+06 | 6.34E+06 | 5.23E+06 | 5.30E+06 | 5.74E+06 | 6.15E+06 | 6.66E+06 | C00492 | ko00052,ko01100,ko02010 |
| pmp001141 | 5.06E+02 | 1.45E+02 | 5.05E+02 | C_25_H_31_NO_10_ | [M+H]+ | Cimicifugamide | Alkaloids | Phenolamine | - | 3 | 9.00E+00 | 9.00E+00 | 9.00E+00 | 4.45E+04 | 5.73E+04 | 7.80E+04 | 9.00E+00 | 9.00E+00 | 9.00E+00 | 9.00E+00 | 9.00E+00 | 9.00E+00 | 3.53E+04 | 2.36E+04 | 2.53E+04 | -- | -- |
| Lmhp008718 | 5.06E+02 | 1.84E+02 | 5.05E+02 | C_25_H_48_NO_7_P | [M+H]+ | LysoPC 17:2 | Lipids | LPC | - | 1 | 1.02E+05 | 1.10E+05 | 1.01E+05 | 7.64E+04 | 7.42E+04 | 5.70E+04 | 8.90E+04 | 8.39E+04 | 8.73E+04 | 6.79E+04 | 8.20E+04 | 8.48E+04 | 9.31E+04 | 7.89E+04 | 8.70E+04 | -- | -- |
| Lmhp010757 | 5.06E+02 | 3.65E+02 | 5.05E+02 | C_25_H_48_NO_7_P | [M+H]+ | LysoPE 20:2 | Lipids | LPE | - | 1 | 2.68E+04 | 2.48E+04 | 2.32E+04 | 4.86E+04 | 5.38E+04 | 5.68E+04 | 4.52E+04 | 4.47E+04 | 5.13E+04 | 3.65E+04 | 4.25E+04 | 4.56E+04 | 3.79E+04 | 3.98E+04 | 4.67E+04 | -- | -- |
| Lmhp010514 | 5.06E+02 | 3.65E+02 | 5.05E+02 | C_25_H_48_NO_7_P | [M+H]+ | LysoPE 20:2(2n isomer) | Lipids | LPE | - | 1 | 2.39E+04 | 2.94E+04 | 3.26E+04 | 5.18E+04 | 5.27E+04 | 4.96E+04 | 4.14E+04 | 4.52E+04 | 4.73E+04 | 3.89E+04 | 4.06E+04 | 3.99E+04 | 4.46E+04 | 4.29E+04 | 4.34E+04 | -- | -- |
| Smnp010958 | 5.07E+02 | 4.89E+02 | 5.06E+02 | C_30_H_18_O_8_ | [M+H]+ | Asphodelin | Quinones | Anthraquinone | 51419-55-7 | 2 | 5.01E+04 | 4.57E+04 | 4.73E+04 | 2.79E+04 | 2.57E+04 | 2.35E+04 | 1.48E+04 | 2.01E+04 | 1.66E+04 | 1.56E+04 | 1.04E+04 | 7.53E+03 | 2.46E+04 | 2.68E+04 | 2.72E+04 | -- | -- |
| Lmhp009590 | 5.08E+02 | 1.84E+02 | 5.07E+02 | C_25_H_50_NO_7_P | [M+H]+ | LysoPC 17:1 | Lipids | LPC | - | 1 | 1.62E+05 | 1.43E+05 | 1.38E+05 | 7.00E+04 | 8.94E+04 | 8.93E+04 | 1.54E+05 | 1.29E+05 | 1.29E+05 | 8.45E+04 | 1.10E+05 | 1.31E+05 | 1.07E+05 | 1.19E+05 | 1.28E+05 | -- | -- |
| Hmcp002187 | 5.09E+02 | 3.47E+02 | 5.08E+02 | C_23_H_24_O_13_ | [M+H]+ | Limocitrin-3-O-galactoside | Flavonoids | Flavonols | - | 3 | 2.42E+04 | 1.52E+04 | 2.83E+04 | 1.27E+04 | 7.33E+03 | 1.01E+04 | 1.33E+04 | 1.81E+04 | 2.89E+04 | 2.41E+04 | 2.44E+04 | 1.91E+04 | 1.40E+04 | 2.09E+04 | 1.58E+04 | -- | -- |
| Hmcp001636 | 5.09E+02 | 3.47E+02 | 5.08E+02 | C_23_H_24_O_13_ | [M+H]+ | Limocitrin-7-O-glucoside | Flavonoids | Flavonols | - | 3 | 4.11E+04 | 2.95E+04 | 3.05E+04 | 5.09E+04 | 5.99E+04 | 5.61E+04 | 6.45E+04 | 4.65E+04 | 4.36E+04 | 8.88E+04 | 1.19E+05 | 7.89E+04 | 5.59E+04 | 6.21E+04 | 3.94E+04 | -- | -- |
| pmb0565 | 5.09E+02 | 3.47E+02 | 5.08E+02 | C_23_H_24_O_13_ | [M+H]+ | Syringetin-3-O-glucoside | Flavonoids | Flavonols | 40039-49-4 | 3 | 3.04E+04 | 3.16E+04 | 2.34E+04 | 4.61E+04 | 7.36E+04 | 6.09E+04 | 5.33E+04 | 5.32E+04 | 4.87E+04 | 1.44E+05 | 1.24E+05 | 7.45E+04 | 8.36E+04 | 5.24E+04 | 6.27E+04 | -- | -- |
| Hmcn000773 | 5.07E+02 | 1.79E+02 | 5.08E+02 | C_21_H_32_O_14_ | [M-H]- | 6'-O-Glucosylaucubin | Others | Others | - | 3 | 2.11E+04 | 1.86E+04 | 1.52E+04 | 2.77E+04 | 3.21E+04 | 3.66E+04 | 3.82E+04 | 4.26E+04 | 4.90E+04 | 4.28E+04 | 4.82E+04 | 4.75E+04 | 3.04E+04 | 3.22E+04 | 3.33E+04 | -- | -- |
| pmb2406 | 5.10E+02 | 1.84E+02 | 5.09E+02 | C_25_H_52_NO_7_P | [M+H]+ | LysoPC 17:0 | Lipids | LPC | 50930-23-9 | 2 | 1.07E+05 | 8.99E+04 | 1.02E+05 | 5.63E+04 | 5.20E+04 | 5.08E+04 | 1.11E+05 | 1.04E+05 | 1.05E+05 | 2.86E+04 | 3.56E+04 | 3.63E+04 | 6.47E+04 | 5.94E+04 | 7.25E+04 | -- | -- |
| Lmhp010515 | 5.10E+02 | 1.84E+02 | 5.09E+02 | C_25_H_52_NO_7_P | [M+H]+ | LysoPC 17:0(2n isomer) | Lipids | LPC | - | 2 | 1.40E+05 | 1.28E+05 | 1.32E+05 | 6.00E+04 | 6.35E+04 | 7.09E+04 | 1.38E+05 | 1.41E+05 | 1.62E+05 | 4.48E+04 | 5.66E+04 | 5.05E+04 | 9.92E+04 | 9.83E+04 | 1.01E+05 | -- | -- |
| Lmhp009526 | 5.15E+02 | 2.61E+02 | 5.14E+02 | C_27_H_46_O_9_ | [M+H]+ | 2-α-Linolenoyl-glycerol-1-O-glucoside* | Lipids | Glycerol ester | - | 1 | 9.00E+00 | 9.00E+00 | 9.00E+00 | 3.14E+04 | 2.24E+04 | 2.33E+04 | 9.00E+00 | 9.00E+00 | 9.00E+00 | 8.33E+03 | 5.80E+03 | 9.50E+03 | 1.30E+04 | 1.17E+04 | 1.32E+04 | -- | -- |
| Lmhp009773 | 5.15E+02 | 2.61E+02 | 5.14E+02 | C_27_H_46_O_9_ | [M+H]+ | 1-α-Linolenoyl-glycerol-3-O-glucoside* | Lipids | Glycerol ester | - | 1 | 9.00E+00 | 9.00E+00 | 9.00E+00 | 2.75E+04 | 2.11E+04 | 1.90E+04 | 9.00E+00 | 9.00E+00 | 9.00E+00 | 7.38E+03 | 7.18E+03 | 6.89E+03 | 6.43E+03 | 7.86E+03 | 1.01E+04 | -- | -- |
| Hmqp006235 | 5.16E+02 | 1.84E+02 | 5.15E+02 | C_26_H_46_NO_7_P | [M+H]+ | LysoPC 18:4 | Lipids | LPC | - | 2 | 2.24E+05 | 2.12E+05 | 2.45E+05 | 1.10E+05 | 1.31E+05 | 1.15E+05 | 7.69E+04 | 7.00E+04 | 8.90E+04 | 6.97E+04 | 7.52E+04 | 8.60E+04 | 1.18E+05 | 1.18E+05 | 1.23E+05 | -- | -- |
| MWSmce346 | 5.17E+02 | 1.63E+02 | 5.16E+02 | C_25_H_24_O_12_ | [M+H]+ | 1,3-Dicaffeoylquinic acid | Phenolic acids | Phenolic acids | 19870-46-3 | 1 | 4.42E+05 | 5.01E+05 | 5.05E+05 | 6.27E+06 | 8.15E+06 | 8.67E+06 | 5.09E+06 | 4.50E+06 | 6.74E+06 | 5.27E+06 | 5.26E+06 | 4.98E+06 | 5.81E+06 | 4.84E+06 | 5.57E+06 | C10445 | -- |
| Li512115 | 5.15E+02 | 3.53E+02 | 5.16E+02 | C_25_H_24_O_12_ | [M-H]- | Isochlorogenic acid B | Phenolic acids | Phenolic acids | 14534-61-3 | 1 | 3.03E+06 | 2.75E+06 | 2.55E+06 | 3.01E+07 | 3.30E+07 | 2.70E+07 | 2.71E+07 | 2.44E+07 | 2.49E+07 | 2.34E+07 | 2.61E+07 | 2.50E+07 | 2.52E+07 | 2.54E+07 | 2.22E+07 | C10468 | -- |
| pmn001382 | 5.15E+02 | 3.53E+02 | 5.16E+02 | C_25_H_24_O_12_ | [M-H]- | Isochlorogenic acid A | Phenolic acids | Phenolic acids | 2450-53-5 | 2 | 7.72E+05 | 5.30E+05 | 7.40E+05 | 7.47E+06 | 7.52E+06 | 1.03E+07 | 4.44E+06 | 2.98E+06 | 5.35E+06 | 3.29E+06 | 3.65E+06 | 4.13E+06 | 4.12E+06 | 4.54E+06 | 5.09E+06 | -- | -- |
| MWSmce328 | 5.17E+02 | 1.63E+02 | 5.16E+02 | C_25_H_24_O_12_ | [M+H]+ | Isochlorogenic acid C | Phenolic acids | Phenolic acids | 57378-72-0 | 1 | 3.20E+06 | 3.25E+06 | 2.68E+06 | 1.97E+07 | 2.77E+07 | 2.77E+07 | 1.85E+07 | 2.12E+07 | 2.02E+07 | 2.34E+07 | 2.25E+07 | 1.90E+07 | 1.81E+07 | 1.84E+07 | 1.87E+07 | -- | -- |
| Lmhp010334 | 5.17E+02 | 2.63E+02 | 5.16E+02 | C_27_H_48_O_9_ | [M+H]+ | 2-Linoleoylglycerol-1-O-glucoside* | Lipids | Glycerol ester | - | 1 | 5.04E+03 | 5.66E+03 | 4.65E+03 | 2.63E+04 | 3.52E+04 | 2.92E+04 | 5.20E+03 | 5.07E+03 | 5.60E+03 | 2.49E+04 | 2.32E+04 | 2.06E+04 | 1.87E+04 | 1.78E+04 | 1.43E+04 | -- | -- |
| Lmhp010573 | 5.17E+02 | 2.63E+02 | 5.16E+02 | C_27_H_48_O_9_ | [M+H]+ | 1-Linoleoylglycerol-3-O-glucoside* | Lipids | Glycerol ester | - | 1 | 5.21E+03 | 4.94E+03 | 5.57E+03 | 3.51E+04 | 3.33E+04 | 2.72E+04 | 4.03E+03 | 5.91E+03 | 4.79E+03 | 2.48E+04 | 2.53E+04 | 2.33E+04 | 1.59E+04 | 2.03E+04 | 1.86E+04 | -- | -- |
| pmn001497 | 5.75E+02 | 2.79E+02 | 5.16E+02 | C_33_H_44_N_2_O_3_ | [M+CH3COOH-H]- | Dendrocrepine | Alkaloids | Alkaloids | 51020-39-4 | 2 | 1.33E+05 | 1.24E+05 | 1.35E+05 | 9.77E+05 | 9.52E+05 | 9.76E+05 | 1.69E+05 | 1.71E+05 | 1.67E+05 | 7.04E+05 | 7.15E+05 | 7.55E+05 | 4.69E+05 | 5.07E+05 | 5.50E+05 | -- | -- |
| pmb0854 | 5.18E+02 | 1.84E+02 | 5.17E+02 | C_26_H_48_NO_7_P | [M+H]+ | LysoPC 18:3 | Lipids | LPC | - | 1 | 1.23E+06 | 1.11E+06 | 1.18E+06 | 6.03E+05 | 6.33E+05 | 6.30E+05 | 1.14E+06 | 1.09E+06 | 1.22E+06 | 7.54E+05 | 7.60E+05 | 8.25E+05 | 7.81E+05 | 9.03E+05 | 8.37E+05 | -- | -- |
| pmb0865 | 5.18E+02 | 1.84E+02 | 5.17E+02 | C_26_H_48_NO_7_P | [M+H]+ | LysoPC 18:3(2n isomer) | Lipids | LPC | - | 1 | 1.39E+06 | 1.16E+06 | 1.31E+06 | 6.00E+05 | 7.51E+05 | 8.29E+05 | 1.28E+06 | 1.18E+06 | 1.27E+06 | 7.47E+05 | 9.07E+05 | 8.81E+05 | 8.58E+05 | 9.40E+05 | 8.68E+05 | -- | -- |
| pmp000530 | 5.19E+02 | 3.57E+02 | 5.18E+02 | C_26_H_30_O_11_ | [M+H]+ | Phellodendroside | Flavonoids | Flavanonols | 40451-69-2 | 2 | 2.66E+05 | 3.01E+05 | 2.74E+05 | 7.89E+05 | 6.96E+05 | 6.47E+05 | 4.66E+05 | 4.79E+05 | 5.00E+05 | 4.24E+05 | 5.72E+05 | 5.83E+05 | 4.25E+05 | 4.70E+05 | 5.07E+05 | -- | -- |
| pmp000531 | 5.19E+02 | 3.57E+02 | 5.18E+02 | C_26_H_30_O_11_ | [M+H]+ | Phellamurin | Flavonoids | Flavanonols | 52589-11-4 | 3 | 1.85E+05 | 1.54E+05 | 1.79E+05 | 2.23E+05 | 2.42E+05 | 2.35E+05 | 3.40E+05 | 2.99E+05 | 3.50E+05 | 2.79E+05 | 3.42E+05 | 3.29E+05 | 2.51E+05 | 2.61E+05 | 2.81E+05 | C09808 | -- |
| pmp001251 | 5.20E+02 | 1.84E+02 | 5.19E+02 | C_26_H_50_NO_7_P | [M+H]+ | LysoPC 18:2(2n isomer) | Lipids | LPC | - | 1 | 4.39E+07 | 4.13E+07 | 3.96E+07 | 2.82E+07 | 2.95E+07 | 2.95E+07 | 2.73E+07 | 2.74E+07 | 2.89E+07 | 2.28E+07 | 2.57E+07 | 2.29E+07 | 2.65E+07 | 2.79E+07 | 3.09E+07 | -- | -- |
| pmp001273 | 5.20E+02 | 1.84E+02 | 5.19E+02 | C_26_H_50_NO_7_P | [M+H]+ | LysoPC 18:2 | Lipids | LPC | - | 1 | 3.81E+07 | 3.46E+07 | 3.69E+07 | 2.13E+07 | 2.44E+07 | 2.33E+07 | 2.32E+07 | 2.27E+07 | 2.59E+07 | 1.80E+07 | 2.00E+07 | 2.04E+07 | 2.43E+07 | 2.56E+07 | 2.65E+07 | -- | -- |
| pma0791 | 5.21E+02 | 2.73E+02 | 5.20E+02 | C_24_H_24_O_13_ | [M+H]+ | Naringenin-7-O-(6''-malonyl)glucoside | Flavonoids | Flavanones | - | 3 | 3.59E+04 | 3.55E+04 | 3.00E+04 | 1.08E+04 | 1.15E+04 | 6.44E+03 | 1.67E+04 | 2.00E+04 | 1.22E+04 | 7.83E+03 | 6.34E+03 | 5.68E+03 | 2.13E+04 | 1.50E+04 | 1.44E+04 | -- | -- |
| Cmsp003083 | 5.21E+02 | 1.31E+02 | 5.20E+02 | C_26_H_32_O_11_ | [M+H]+ | Dehydrodiconiferyl alcohol-4-O-glucoside | Others | Others | 107870-88-2 | 1 | 1.70E+06 | 1.11E+06 | 1.42E+06 | 2.42E+06 | 2.60E+06 | 2.58E+06 | 1.40E+06 | 1.30E+06 | 1.49E+06 | 1.69E+06 | 1.70E+06 | 1.64E+06 | 1.69E+06 | 1.77E+06 | 1.81E+06 | -- | -- |
| Lmsp003655 | 5.21E+02 | 1.31E+02 | 5.20E+02 | C_26_H_32_O_11_ | [M+H]+ | Dehydrodiconiferyl alcohol-gamma'-O-glucoside | Phenolic acids | Phenolic acids | - | 1 | 1.43E+06 | 1.21E+06 | 1.47E+06 | 2.30E+06 | 2.26E+06 | 2.05E+06 | 1.27E+06 | 1.10E+06 | 1.34E+06 | 1.44E+06 | 1.34E+06 | 1.37E+06 | 1.48E+06 | 1.49E+06 | 1.80E+06 | -- | -- |
| Lmhp010190 | 5.22E+02 | 1.84E+02 | 5.21E+02 | C_26_H_52_NO_7_P | [M+H]+ | LysoPC 18:1(2n isomer) | Lipids | LPC | - | 1 | 3.55E+06 | 3.04E+06 | 3.27E+06 | 4.53E+06 | 4.95E+06 | 4.76E+06 | 2.96E+06 | 2.93E+06 | 3.16E+06 | 3.44E+06 | 3.64E+06 | 3.29E+06 | 3.68E+06 | 3.40E+06 | 3.73E+06 | -- | -- |
| pmp001281 | 5.22E+02 | 1.84E+02 | 5.21E+02 | C_26_H_52_NO_7_P | [M+H]+ | LysoPC 18:1 | Lipids | LPC | - | 1 | 8.13E+06 | 7.41E+06 | 7.45E+06 | 3.62E+06 | 3.82E+06 | 3.99E+06 | 5.22E+06 | 5.13E+06 | 5.80E+06 | 3.09E+06 | 3.24E+06 | 3.38E+06 | 4.72E+06 | 4.75E+06 | 5.20E+06 | -- | -- |
| HJAP018 | 5.23E+02 | 3.19E+02 | 5.22E+02 | C_23_H_22_O_14_ | [M+H]+ | Myricetin-3-O-(6''-acetyl)glucoside | Flavonoids | Flavonols | - | 3 | 2.55E+04 | 1.08E+04 | 8.88E+03 | 2.79E+04 | 2.23E+04 | 1.96E+04 | 3.36E+04 | 2.20E+04 | 2.98E+04 | 1.40E+04 | 2.57E+04 | 1.78E+04 | 2.92E+04 | 2.41E+04 | 1.33E+04 | -- | -- |
| Lmmn002274 | 5.21E+02 | 3.59E+02 | 5.22E+02 | C_26_H_34_O_11_ | [M-H]- | Isolariciresinol-9'-O-glucoside | Lignans and Coumarins | Lignans | 63358-12-3 | 3 | 8.59E+03 | 1.15E+04 | 1.13E+04 | 2.26E+04 | 1.57E+04 | 2.20E+04 | 2.35E+04 | 2.17E+04 | 1.48E+04 | 1.34E+04 | 2.05E+04 | 1.65E+04 | 1.09E+04 | 1.43E+04 | 1.13E+04 | -- | -- |
| Lmtn002596 | 5.21E+02 | 3.59E+02 | 5.22E+02 | C_26_H_34_O_11_ | [M-H]- | Dihydrodehydrodiconiferyl alcohol-4-O-glucoside | Lignans and Coumarins | Lignans | - | 3 | 9.00E+00 | 9.00E+00 | 9.00E+00 | 1.40E+04 | 1.55E+04 | 8.47E+03 | 2.34E+04 | 1.53E+04 | 2.12E+04 | 1.32E+04 | 3.08E+04 | 2.60E+04 | 2.32E+04 | 1.64E+04 | 1.77E+04 | -- | -- |
| mws0126 | 5.24E+02 | 1.84E+02 | 5.23E+02 | C_26_H_54_NO_7_P | [M+H]+ | LysoPC 18:0 | Lipids | LPC | 19420-57-6 | 1 | 4.86E+05 | 4.37E+05 | 4.45E+05 | 4.03E+05 | 4.46E+05 | 4.67E+05 | 5.40E+05 | 5.17E+05 | 5.46E+05 | 2.64E+05 | 3.17E+05 | 3.13E+05 | 4.29E+05 | 4.54E+05 | 4.36E+05 | -- | -- |
| pmd0136 | 5.24E+02 | 1.84E+02 | 5.23E+02 | C_26_H_54_NO_7_P | [M+H]+ | LysoPC 18:0(2n isomer) | Lipids | LPC | - | 1 | 1.64E+05 | 1.56E+05 | 1.70E+05 | 6.69E+04 | 7.86E+04 | 8.98E+04 | 1.51E+05 | 1.30E+05 | 1.68E+05 | 4.30E+04 | 5.74E+04 | 5.82E+04 | 1.10E+05 | 1.10E+05 | 1.13E+05 | -- | -- |
| Lmtn003096 | 5.23E+02 | 3.61E+02 | 5.24E+02 | C_26_H_36_O_11_ | [M-H]- | Secoisolariciresinol 4-O-glucoside | Lignans and Coumarins | Lignans | - | 2 | 7.92E+05 | 7.61E+05 | 8.96E+05 | 1.59E+06 | 1.73E+06 | 1.57E+06 | 1.55E+06 | 1.50E+06 | 1.61E+06 | 1.84E+06 | 1.70E+06 | 1.85E+06 | 1.40E+06 | 1.48E+06 | 1.55E+06 | -- | -- |
| Zmcn005156 | 5.29E+02 | 3.53E+02 | 5.30E+02 | C_26_H_26_O_12_ | [M-H]- | 4,5-O-Dicaffeoylquinic Acid Methyl Ester | Phenolic acids | Phenolic acids | - | 2 | 6.17E+04 | 5.95E+04 | 7.94E+04 | 2.00E+06 | 1.96E+06 | 1.96E+06 | 3.42E+05 | 2.98E+05 | 2.91E+05 | 2.80E+05 | 3.22E+05 | 3.18E+05 | 5.77E+05 | 7.58E+05 | 7.78E+05 | -- | -- |
| Lmjp003731 | 5.31E+02 | 1.77E+02 | 5.30E+02 | C_26_H_26_O_12_ | [M+H]+ | 3,4-O-Dicaffeoylquinic Acid Methyl Ester* | Phenolic acids | Phenolic acids | - | 2 | 2.05E+05 | 2.11E+05 | 2.22E+05 | 3.79E+06 | 4.22E+06 | 3.68E+06 | 1.09E+06 | 1.13E+06 | 1.64E+06 | 1.06E+06 | 1.38E+06 | 1.04E+06 | 1.72E+06 | 1.76E+06 | 1.81E+06 | -- | -- |
| Lmjp003822 | 5.31E+02 | 1.77E+02 | 5.30E+02 | C_26_H_26_O_12_ | [M+H]+ | 3,5-O-Dicaffeoylquinic Acid Methyl Ester* | Phenolic acids | Phenolic acids | 159934-13-1 | 2 | 1.88E+05 | 1.91E+05 | 2.38E+05 | 4.10E+06 | 4.19E+06 | 3.58E+06 | 1.19E+06 | 1.27E+06 | 1.37E+06 | 1.32E+06 | 1.27E+06 | 1.09E+06 | 1.89E+06 | 1.71E+06 | 1.90E+06 | -- | -- |
| pmb2554 | 5.29E+02 | 1.93E+02 | 5.30E+02 | C_23_H_30_O_14_ | [M-H]- | 5-O-Feruloyl quinic acid glucoside* | Phenolic acids | Phenolic acids | - | 1 | 9.69E+04 | 1.02E+05 | 9.32E+04 | 8.33E+04 | 9.38E+04 | 8.60E+04 | 1.30E+05 | 1.20E+05 | 1.34E+05 | 1.47E+05 | 1.52E+05 | 1.74E+05 | 1.18E+05 | 1.19E+05 | 1.16E+05 | -- | -- |
| pmb2833 | 5.29E+02 | 1.93E+02 | 5.30E+02 | C_23_H_30_O_14_ | [M-H]- | 3-O-Feruloylquinic acid-O-glucoside* | Phenolic acids | Phenolic acids | - | 1 | 8.57E+04 | 9.72E+04 | 9.65E+04 | 7.66E+04 | 6.55E+04 | 7.25E+04 | 1.10E+05 | 1.08E+05 | 1.11E+05 | 1.28E+05 | 1.39E+05 | 1.54E+05 | 9.65E+04 | 1.02E+05 | 1.23E+05 | -- | -- |
| Lmhp007840 | 5.34E+02 | 1.84E+02 | 5.33E+02 | C_27_H_52_NO_7_P | [M+H]+ | LysoPC 19:2 | Lipids | LPC | - | 3 | 8.72E+03 | 8.65E+03 | 9.03E+03 | 3.40E+03 | 6.42E+03 | 3.72E+03 | 5.75E+03 | 5.67E+03 | 5.02E+03 | 2.85E+03 | 5.21E+03 | 3.87E+03 | 6.43E+03 | 7.55E+03 | 3.33E+03 | -- | -- |
| Lmmp003817 | 5.35E+02 | 2.87E+02 | 5.34E+02 | C_24_H_22_O_14_ | [M+H]+ | Kaempferol-3-O-(6''-malonyl)glucoside* | Flavonoids | Flavonols | - | 3 | 2.55E+05 | 2.41E+05 | 1.80E+05 | 3.12E+05 | 2.74E+05 | 3.31E+05 | 1.09E+05 | 1.18E+05 | 1.39E+05 | 1.83E+05 | 1.49E+05 | 1.61E+05 | 1.93E+05 | 2.34E+05 | 2.91E+05 | -- | -- |
| Lmdp004892 | 5.35E+02 | 2.87E+02 | 5.34E+02 | C_24_H_22_O_14_ | [M+H]+ | Kaempferol-3-O-(6''-malonyl)galactoside* | Flavonoids | Flavonols | - | 3 | 2.69E+05 | 1.60E+05 | 1.91E+05 | 2.86E+05 | 2.29E+05 | 2.65E+05 | 1.06E+05 | 1.04E+05 | 1.42E+05 | 1.39E+05 | 1.55E+05 | 2.07E+05 | 2.25E+05 | 1.93E+05 | 2.47E+05 | -- | -- |
| mws0092 | 5.33E+02 | 3.71E+02 | 5.34E+02 | C_27_H_34_O_11_ | [M-H]- | Arctiin | Lignans and Coumarins | Lignans | 20362-31-6 | 3 | 9.13E+03 | 7.88E+03 | 7.22E+03 | 6.15E+04 | 8.15E+04 | 7.55E+04 | 2.47E+04 | 2.59E+04 | 2.54E+04 | 2.95E+04 | 2.83E+04 | 4.58E+04 | 2.84E+04 | 2.78E+04 | 3.46E+04 | C16915 | -- |
| Lmhp010908 | 5.36E+02 | 1.84E+02 | 5.35E+02 | C_27_H_54_NO_7_P | [M+H]+ | LysoPC 19:1 | Lipids | LPC | - | 3 | 1.16E+05 | 1.05E+05 | 1.11E+05 | 1.31E+05 | 1.06E+05 | 1.32E+05 | 8.46E+04 | 7.68E+04 | 7.03E+04 | 7.27E+04 | 5.49E+04 | 6.46E+04 | 8.54E+04 | 9.86E+04 | 9.57E+04 | -- | -- |
| Zmfn000481 | 5.35E+02 | 3.23E+02 | 5.36E+02 | C_14_H_22_N_2_O_16_P_2_ | [M-H]- | Uridine-5'-Diphosphate-D-Xylose | Nucleotides and derivatives | Nucleotides and derivatives | 3616-06-6 | 3 | 1.02E+04 | 8.92E+03 | 7.07E+03 | 1.39E+05 | 1.20E+05 | 1.31E+05 | 3.31E+04 | 2.98E+04 | 3.10E+04 | 5.06E+04 | 5.55E+04 | 5.59E+04 | 5.42E+04 | 6.62E+04 | 6.56E+04 | C00190 | ko00520,ko00908,ko01100 |
| pmn001375 | 5.35E+02 | 3.73E+02 | 5.36E+02 | C_26_H_32_O_12_ | [M-H]- | 1-Hydroxypinoresinol-1-O-Glucoside | Lignans and Coumarins | Lignans | 81495-71-8 | 2 | 5.45E+04 | 5.21E+04 | 3.88E+04 | 9.25E+04 | 9.68E+04 | 1.01E+05 | 7.23E+04 | 6.23E+04 | 8.81E+04 | 7.70E+04 | 9.09E+04 | 9.83E+04 | 6.94E+04 | 1.09E+05 | 8.17E+04 | -- | -- |
| Zmbp004679 | 5.41E+02 | 2.47E+02 | 5.40E+02 | C_25_H_32_O_13_ | [M+H]+ | Decuroside IV | Lignans and Coumarins | Coumarins | 96627-80-4 | 2 | 4.31E+05 | 3.84E+05 | 2.65E+05 | 2.18E+05 | 4.55E+05 | 4.70E+05 | 2.11E+05 | 4.67E+05 | 4.14E+05 | 2.85E+05 | 3.07E+05 | 4.06E+05 | 3.11E+05 | 3.72E+05 | 5.23E+05 | -- | -- |
| Lmhp008742 | 5.42E+02 | 1.84E+02 | 5.41E+02 | C_28_H_48_NO_7_P | [M+H]+ | LysoPC 20:5 | Lipids | LPC | 162440-04-2 | 2 | 5.04E+03 | 6.35E+03 | 6.57E+03 | 1.40E+04 | 2.22E+04 | 1.42E+04 | 7.09E+03 | 8.53E+03 | 9.84E+03 | 2.32E+03 | 4.87E+03 | 6.68E+03 | 1.02E+04 | 1.24E+04 | 7.14E+03 | -- | -- |
| pmc0960 | 5.44E+02 | 1.84E+02 | 5.43E+02 | C_28_H_50_NO_7_P | [M+H]+ | LysoPC 20:4 | Lipids | LPC | - | 3 | 7.40E+03 | 5.50E+03 | 7.49E+03 | 2.04E+04 | 1.24E+04 | 1.96E+04 | 9.00E+00 | 9.00E+00 | 9.00E+00 | 8.50E+03 | 5.64E+03 | 5.44E+03 | 1.02E+04 | 8.67E+03 | 5.70E+03 | -- | -- |
| pmp001282 | 5.44E+02 | 4.85E+02 | 5.43E+02 | C_28_H_50_NO_7_P | [M+H]+ | Propyl 2-(trimethylammonio)ethyl phosphate | Others | Others | - | 1 | 2.24E+05 | 2.71E+05 | 2.75E+05 | 1.73E+05 | 1.34E+05 | 1.19E+05 | 2.17E+05 | 1.99E+05 | 2.31E+05 | 1.55E+05 | 1.47E+05 | 1.67E+05 | 1.81E+05 | 2.43E+05 | 2.37E+05 | -- | -- |
| Lmhp009890 | 5.46E+02 | 1.84E+02 | 5.45E+02 | C_28_H_52_NO_7_P | [M+H]+ | LysoPC 20:3 | Lipids | LPC | 1199257-41-4 | 3 | 6.38E+03 | 2.82E+03 | 5.71E+03 | 3.87E+04 | 2.24E+04 | 2.98E+04 | 6.27E+03 | 5.13E+03 | 3.17E+03 | 4.09E+03 | 3.49E+03 | 5.72E+03 | 1.07E+04 | 8.42E+03 | 7.59E+03 | -- | -- |
| pmd0147 | 5.48E+02 | 1.84E+02 | 5.47E+02 | C_28_H_54_NO_7_P | [M+H]+ | LysoPC 20:2 | Lipids | LPC | - | 3 | 1.04E+05 | 9.56E+04 | 1.08E+05 | 4.46E+04 | 4.39E+04 | 4.02E+04 | 1.10E+05 | 9.46E+04 | 1.12E+05 | 4.71E+04 | 6.11E+04 | 5.07E+04 | 6.73E+04 | 6.89E+04 | 6.94E+04 | -- | -- |
| pmd0146 | 5.48E+02 | 1.84E+02 | 5.47E+02 | C_28_H_54_NO_7_P | [M+H]+ | LysoPC 20:2(2n isomer) | Lipids | LPC | - | 3 | 3.74E+04 | 3.44E+04 | 3.38E+04 | 1.81E+04 | 1.50E+04 | 1.79E+04 | 3.56E+04 | 3.30E+04 | 3.67E+04 | 1.42E+04 | 1.97E+04 | 2.28E+04 | 2.42E+04 | 2.57E+04 | 3.15E+04 | -- | -- |
| Zmdp003677 | 5.49E+02 | 2.55E+02 | 5.48E+02 | C_26_H_28_O_13_ | [M+H]+ | Daidzein-7-O-Glucoside-4'-O-Apioside | Flavonoids | Isoflavones | 108069-01-8 | 2 | 4.94E+03 | 1.16E+04 | 9.78E+03 | 9.20E+03 | 1.94E+04 | 2.04E+04 | 1.06E+04 | 9.17E+03 | 8.46E+03 | 1.56E+04 | 1.70E+04 | 1.52E+04 | 1.62E+04 | 1.27E+04 | 1.69E+04 | -- | -- |
| Lmhp011549 | 5.50E+02 | 1.84E+02 | 5.49E+02 | C_28_H_56_NO_7_P | [M+H]+ | LysoPC 20:1 | Lipids | LPC | - | 3 | 1.63E+04 | 1.87E+04 | 1.72E+04 | 8.34E+03 | 9.95E+03 | 1.29E+04 | 1.83E+04 | 1.41E+04 | 1.80E+04 | 1.13E+04 | 9.42E+03 | 1.27E+04 | 1.73E+04 | 1.55E+04 | 1.38E+04 | -- | -- |
| HJAP007 | 5.51E+02 | 3.47E+02 | 5.50E+02 | C_25_H_26_O_14_ | [M+H]+ | Syringetin-3-O-(6''-Acetyl)glucoside | Flavonoids | Flavonols | - | 3 | 6.84E+03 | 5.71E+03 | 4.88E+03 | 2.77E+03 | 5.29E+03 | 6.89E+03 | 4.74E+03 | 3.64E+03 | 1.58E+03 | 2.60E+03 | 2.85E+03 | 2.95E+03 | 8.90E+03 | 4.50E+03 | 5.92E+03 | -- | -- |
| Lmmn002260 | 5.51E+02 | 3.89E+02 | 5.52E+02 | C_27_H_36_O_12_ | [M-H]- | 5'-Methoxyisolariciresinol-9'-O-glucoside | Lignans and Coumarins | Lignans | - | 2 | 4.86E+05 | 4.61E+05 | 5.17E+05 | 6.62E+05 | 6.91E+05 | 6.15E+05 | 1.08E+06 | 1.16E+06 | 1.21E+06 | 1.10E+06 | 1.09E+06 | 1.13E+06 | 8.03E+05 | 9.00E+05 | 8.73E+05 | -- | -- |
| Rfmb25702 | 5.61E+02 | 3.57E+02 | 5.62E+02 | C_28_H_34_O_12_ | [M-H]- | Pinoresinol-4-O-(6''-acetyl)glucoside | Lignans and Coumarins | Lignans | - | 3 | 6.08E+03 | 4.97E+03 | 4.32E+03 | 9.59E+03 | 8.98E+03 | 5.10E+03 | 1.28E+04 | 7.90E+03 | 7.77E+03 | 1.13E+04 | 1.08E+04 | 1.30E+04 | 7.58E+03 | 6.06E+03 | 6.11E+03 | -- | -- |
| pmb2922 | 5.65E+02 | 3.23E+02 | 5.66E+02 | C_15_H_24_N_2_O_17_P_2_ | [M-H]- | Uridine 5'-diphospho-D-glucose | Nucleotides and derivatives | Nucleotides and derivatives | 133-89-1 | 1 | 2.89E+04 | 4.20E+04 | 3.75E+04 | 1.35E+06 | 1.40E+06 | 1.51E+06 | 2.11E+05 | 2.18E+05 | 2.14E+05 | 9.88E+05 | 9.75E+05 | 9.66E+05 | 5.71E+05 | 7.65E+05 | 6.68E+05 | C00029 | ko00040,ko00052,ko00053,ko00240,ko00500,ko00520,ko00524,ko00561,ko00908,ko01100,ko01110,ko01240 |
| Zmbp004654 | 5.71E+02 | 2.47E+02 | 5.70E+02 | C_26_H_34_O_14_ | [M+H]+ | Decuroside III | Lignans and Coumarins | Coumarins | 96638-81-2 | 2 | 5.58E+05 | 4.03E+05 | 4.40E+05 | 1.17E+06 | 6.35E+05 | 1.19E+06 | 7.70E+05 | 7.55E+05 | 8.51E+05 | 4.29E+05 | 7.64E+05 | 5.26E+05 | 6.18E+05 | 7.77E+05 | 8.34E+05 | C09257 | -- |
| Lmpp003930 | 5.79E+02 | 2.71E+02 | 5.78E+02 | C_30_H_26_O_12_ | [M+H]+ | Apigenin-7-O-(6''-p-Coumaryl)glucoside | Flavonoids | Flavones | - | 3 | 2.03E+04 | 1.52E+04 | 1.08E+04 | 2.92E+04 | 2.04E+04 | 2.40E+04 | 3.81E+04 | 3.30E+04 | 4.19E+04 | 3.11E+04 | 4.50E+04 | 5.03E+04 | 3.29E+04 | 3.20E+04 | 3.93E+04 | -- | -- |
| Lmgp004474 | 5.79E+02 | 2.71E+02 | 5.78E+02 | C_27_H_30_O_14_ | [M+H]+ | Genistein-7-O-galactoside-rhamnose | Flavonoids | Isoflavones | - | 3 | 1.27E+04 | 2.28E+04 | 1.17E+04 | 3.33E+04 | 2.48E+04 | 2.21E+04 | 4.73E+04 | 3.57E+04 | 3.23E+04 | 4.38E+04 | 4.19E+04 | 2.84E+04 | 2.41E+04 | 2.35E+04 | 3.55E+04 | -- | -- |
| pmb0492 | 5.84E+02 | 3.25E+02 | 5.83E+02 | C_34_H_37_N_3_O_6_ | [M+H]+ | N',N'',N'''-p-Coumaroyl-cinnamoyl-caffeoyl spermidine | Alkaloids | Phenolamine | - | 3 | 3.01E+05 | 2.55E+05 | 3.10E+05 | 4.30E+05 | 3.48E+05 | 3.33E+05 | 1.93E+05 | 1.87E+05 | 2.05E+05 | 1.64E+05 | 1.75E+05 | 1.37E+05 | 2.33E+05 | 2.24E+05 | 2.84E+05 | -- | -- |
| Lmyp004407 | 5.95E+02 | 2.87E+02 | 5.94E+02 | C_30_H_26_O_13_ | [M+H]+ | Kaempferol-3-O-(2''-p-Coumaroyl)galactoside | Flavonoids | Flavonols | - | 3 | 2.44E+03 | 2.29E+03 | 3.07E+03 | 4.04E+03 | 4.10E+03 | 7.69E+03 | 2.15E+04 | 1.74E+04 | 2.52E+04 | 2.86E+04 | 2.30E+04 | 2.52E+04 | 1.52E+04 | 1.37E+04 | 1.58E+04 | -- | -- |
| Smlp011494 | 5.95E+02 | 5.95E+02 | 5.94E+02 | C_34_H_26_O_10_ | [M+H]+ | Amentoflavone-4',4''',7,7''-tetramethyl ether | Flavonoids | Flavones | - | 2 | 1.44E+06 | 1.68E+06 | 1.78E+06 | 1.86E+06 | 1.94E+06 | 1.75E+06 | 1.93E+06 | 2.35E+06 | 2.65E+06 | 2.42E+06 | 2.40E+06 | 2.27E+06 | 1.63E+06 | 1.91E+06 | 2.05E+06 | -- | -- |
| MWSslk254 | 5.95E+02 | 2.87E+02 | 5.94E+02 | C_27_H_30_O_15_ | [M+H]+ | Kaempferol-3-O-glucorhamnoside* | Flavonoids | Flavonols | 40437-72-7 | 1 | 3.35E+05 | 1.16E+05 | 2.13E+05 | 9.00E+00 | 9.00E+00 | 9.00E+00 | 9.00E+00 | 9.00E+00 | 9.00E+00 | 9.00E+00 | 9.00E+00 | 9.00E+00 | 5.82E+04 | 6.28E+04 | 7.52E+04 | -- | -- |
| MWSHY0080 | 5.95E+02 | 2.87E+02 | 5.94E+02 | C_27_H_30_O_15_ | [M+H]+ | Luteolin-7-O-neohesperidoside (Lonicerin) | Flavonoids | Flavones | 25694-72-8 | 1 | 3.23E+05 | 9.27E+04 | 1.90E+05 | 9.00E+00 | 9.00E+00 | 9.00E+00 | 9.00E+00 | 9.00E+00 | 9.00E+00 | 9.00E+00 | 9.00E+00 | 9.00E+00 | 4.97E+04 | 5.24E+04 | 7.57E+04 | C12630 | ko00944 |
| mws1073 | 5.95E+02 | 4.57E+02 | 5.94E+02 | C_27_H_30_O_15_ | [M+H]+ | Apigenin-6,8-di-C-glucoside (Vicenin-2) | Flavonoids | Flavonoid carbonoside | 23666-13-9 | 2 | 3.16E+04 | 1.08E+04 | 2.87E+04 | 3.47E+04 | 5.46E+04 | 4.37E+04 | 8.26E+03 | 1.99E+04 | 1.90E+04 | 2.81E+04 | 1.49E+04 | 3.26E+04 | 3.26E+04 | 2.61E+04 | 2.80E+04 | C10195 | -- |
| MWSHY0061 | 5.95E+02 | 2.87E+02 | 5.94E+02 | C_27_H_30_O_15_ | [M+H]+ | Kaempferol-3-O-neohesperidoside* | Flavonoids | Flavonols | 32602-81-6 | 1 | 3.26E+05 | 8.67E+04 | 2.13E+05 | 9.00E+00 | 9.00E+00 | 9.00E+00 | 9.00E+00 | 9.00E+00 | 9.00E+00 | 9.00E+00 | 9.00E+00 | 9.00E+00 | 5.32E+04 | 4.76E+04 | 5.71E+04 | -- | -- |
| Lmnp102682 | 5.95E+02 | 4.63E+02 | 5.94E+02 | C_27_H_30_O_15_ | [M+H]+ | Hispidulin-8-C-(2''-O-xylosyl)glucoside | Flavonoids | Flavonoid carbonoside | - | 3 | 3.08E+04 | 2.06E+04 | 2.74E+04 | 2.10E+04 | 1.35E+04 | 2.52E+04 | 1.65E+04 | 1.66E+04 | 1.60E+04 | 2.94E+04 | 2.33E+04 | 1.47E+04 | 1.87E+04 | 3.26E+04 | 2.19E+04 | -- | -- |
| Lmsp004670 | 5.95E+02 | 2.87E+02 | 5.94E+02 | C_27_H_30_O_15_ | [M+H]+ | Kaempferol-3-O-glucoside-7-O-rhamnoside* | Flavonoids | Flavonols | - | 1 | 2.93E+05 | 6.30E+04 | 1.88E+05 | 9.00E+00 | 9.00E+00 | 9.00E+00 | 9.00E+00 | 9.00E+00 | 9.00E+00 | 9.00E+00 | 9.00E+00 | 9.00E+00 | 5.82E+04 | 5.82E+04 | 6.85E+04 | -- | -- |
| pme1793 | 5.95E+02 | 2.71E+02 | 5.95E+02 | C_27_H_31_O_15_+ | [M]+ | Pelargonidin-3,5-O-diglucoside | Flavonoids | Anthocyanidins | 17334-58-6 | 3 | 5.19E+03 | 1.30E+04 | 8.87E+03 | 5.69E+03 | 8.32E+03 | 7.76E+03 | 8.29E+03 | 9.90E+03 | 1.14E+04 | 1.40E+04 | 7.80E+03 | 1.14E+04 | 1.29E+04 | 9.33E+03 | 1.09E+04 | C08725 | ko00942 |
| Cmyp005122 | 6.03E+02 | 2.69E+02 | 6.02E+02 | C_34_H_34_O_10_ | [M+H]+ | Genkwadaphnin | Terpenoids | Ditepenoids | 55073-32-0 | 2 | 6.02E+05 | 5.33E+05 | 6.48E+05 | 5.33E+05 | 5.56E+05 | 5.05E+05 | 3.54E+05 | 4.13E+05 | 3.70E+05 | 3.14E+05 | 2.88E+05 | 3.42E+05 | 4.69E+05 | 4.81E+05 | 4.15E+05 | -- | -- |
| Lmqn000780 | 6.06E+02 | 3.85E+02 | 6.07E+02 | C_17_H_27_N_3_O_17_P_2_ | [M-H]- | Uridine 5'-diphospho-N-acetylglucosamine | Nucleotides and derivatives | Nucleotides and derivatives | 528-04-1 | 2 | 5.27E+03 | 5.25E+03 | 3.86E+03 | 4.32E+04 | 3.91E+04 | 4.75E+04 | 3.33E+04 | 3.08E+04 | 3.83E+04 | 6.43E+04 | 6.40E+04 | 6.95E+04 | 3.26E+04 | 3.96E+04 | 4.43E+04 | C00043 | ko00520,ko00524,ko01100 |
| pmp001080 | 6.09E+02 | 3.01E+02 | 6.08E+02 | C_28_H_32_O_15_ | [M+H]+ | Diosmetin-7-O-Neohesperidoside (Neodiosmin) | Flavonoids | Flavanones | 38665-01-9 | 1 | 1.37E+04 | 1.52E+04 | 2.26E+04 | 4.68E+04 | 3.91E+04 | 6.04E+04 | 7.19E+04 | 8.88E+04 | 9.65E+04 | 1.37E+05 | 1.47E+05 | 2.47E+05 | 6.72E+04 | 6.84E+04 | 9.06E+04 | -- | -- |
| mws1661 | 6.09E+02 | 3.01E+02 | 6.08E+02 | C_28_H_32_O_15_ | [M+H]+ | Diosmetin-7-O-rutinoside (Diosmin) | Flavonoids | Flavanones | 520-27-4 | 1 | 2.20E+05 | 2.20E+05 | 2.16E+05 | 6.02E+05 | 6.70E+05 | 6.76E+05 | 1.44E+06 | 1.26E+06 | 1.25E+06 | 1.77E+06 | 1.81E+06 | 2.21E+06 | 1.01E+06 | 9.53E+05 | 1.20E+06 | C10039 | -- |
| pmb3002 | 6.07E+02 | 2.99E+02 | 6.08E+02 | C_28_H_32_O_15_ | [M-H]- | Chrysoeriol-7-O-rutinoside | Flavonoids | Flavones | - | 1 | 6.45E+04 | 6.58E+04 | 7.32E+04 | 1.24E+05 | 1.42E+05 | 1.75E+05 | 3.58E+05 | 3.77E+05 | 3.24E+05 | 4.92E+05 | 4.94E+05 | 5.34E+05 | 2.46E+05 | 2.26E+05 | 2.70E+05 | -- | -- |
| Hmgp002121 | 6.09E+02 | 4.63E+02 | 6.08E+02 | C_31_H_28_O_13_ | [M+H]+ | Hispidulin-7-O-(6''-O-p-Coumaroyl)Glucoside | Flavonoids | Flavones | - | 2 | 1.15E+05 | 9.81E+04 | 1.35E+05 | 3.07E+05 | 3.65E+05 | 3.23E+05 | 6.20E+05 | 5.55E+05 | 5.27E+05 | 9.17E+05 | 9.23E+05 | 1.16E+06 | 4.68E+05 | 5.11E+05 | 5.55E+05 | -- | -- |
| Lmpp003929 | 6.09E+02 | 3.01E+02 | 6.09E+02 | C_31_H_29_O_13_+ | [M]+ | Peonidin-3-O-(6''-O-p-coumaroyl)glucoside | Flavonoids | Anthocyanidins | - | 1 | 1.15E+05 | 1.19E+05 | 1.34E+05 | 3.35E+05 | 4.04E+05 | 3.68E+05 | 6.40E+05 | 6.02E+05 | 5.98E+05 | 9.84E+05 | 9.73E+05 | 1.23E+06 | 4.96E+05 | 5.23E+05 | 5.28E+05 | -- | -- |
| Lmbp002592 | 6.11E+02 | 2.87E+02 | 6.10E+02 | C_27_H_30_O_16_ | [M+H]+ | Kaempferol-3,7-di-O-glucoside | Flavonoids | Flavonols | - | 3 | 9.50E+04 | 9.24E+04 | 8.57E+04 | 9.00E+00 | 9.00E+00 | 9.00E+00 | 9.00E+00 | 9.00E+00 | 9.00E+00 | 9.00E+00 | 9.00E+00 | 9.00E+00 | 2.46E+04 | 2.57E+04 | 2.81E+04 | -- | -- |
| pmb0711 | 6.11E+02 | 3.03E+02 | 6.10E+02 | C_27_H_30_O_16_ | [M+H]+ | Quercetin-7-O-rutinoside* | Flavonoids | Flavonols | 147714-62-3 | 2 | 9.48E+04 | 2.51E+04 | 6.14E+04 | 6.96E+03 | 6.27E+03 | 1.53E+04 | 2.08E+03 | 6.30E+03 | 6.10E+03 | 7.67E+03 | 4.19E+03 | 4.20E+03 | 2.06E+04 | 3.26E+04 | 1.89E+04 | -- | -- |
| Zmxp003107 | 6.11E+02 | 2.87E+02 | 6.10E+02 | C_27_H_30_O_16_ | [M+H]+ | Luteolin-7,3'-di-O-glucoside | Flavonoids | Flavones | 52187-80-1 | 3 | 6.39E+04 | 1.05E+05 | 9.16E+04 | 1.26E+03 | 9.95E+03 | 1.07E+04 | 3.45E+03 | 8.53E+03 | 9.99E+03 | 9.28E+03 | 3.02E+03 | 4.98E+03 | 3.13E+04 | 1.92E+04 | 3.71E+04 | -- | -- |
| MWSHY0067 | 6.11E+02 | 3.03E+02 | 6.10E+02 | C_27_H_30_O_16_ | [M+H]+ | Quercetin-3-O-rutinoside (Rutin)* | Flavonoids | Flavonols | 153-18-4 | 2 | 7.69E+04 | 2.09E+04 | 3.28E+04 | 1.12E+04 | 6.96E+03 | 9.61E+03 | 4.83E+03 | 3.33E+03 | 9.80E+03 | 4.91E+03 | 5.57E+03 | 7.68E+03 | 2.23E+04 | 2.43E+04 | 1.47E+04 | C05625 | ko00944,ko01100,ko01110 |
| Lmpp003662 | 6.11E+02 | 3.03E+02 | 6.11E+02 | C_30_H_27_O_14_+ | [M]+ | Delphinidin-3-O-(6''-O-p-coumaroyl)glucoside | Flavonoids | Anthocyanidins | - | 3 | 9.40E+04 | 2.08E+04 | 3.76E+04 | 9.00E+00 | 9.00E+00 | 9.00E+00 | 9.00E+00 | 9.00E+00 | 9.00E+00 | 9.00E+00 | 9.00E+00 | 9.00E+00 | 2.73E+04 | 1.75E+04 | 1.61E+04 | C16370 | ko00942 |
| MWS0132 | 6.11E+02 | 3.06E+02 | 6.12E+02 | C_20_H_32_N_6_O_12_S_2_ | [M-H]- | Oxiglutatione | Amino acids and derivatives | Amino acids and derivatives | 27025-41-8 | 1 | 7.14E+04 | 7.97E+04 | 7.01E+04 | 8.32E+05 | 7.37E+05 | 7.87E+05 | 1.36E+05 | 1.18E+05 | 9.25E+04 | 3.11E+05 | 4.05E+05 | 3.71E+05 | 3.06E+05 | 3.95E+05 | 3.29E+05 | C00127 | ko00480,ko01100,ko01240 |
| pmb0628 | 6.13E+02 | 4.51E+02 | 6.12E+02 | C_27_H_32_O_16_ | [M+H]+ | Eriodictyol-8-C-glucoside-4'-O-glucoside | Flavonoids | Flavanones | - | 3 | 5.72E+04 | 3.54E+04 | 4.17E+04 | 9.00E+00 | 9.00E+00 | 9.00E+00 | 9.00E+00 | 9.00E+00 | 9.00E+00 | 9.00E+00 | 9.00E+00 | 9.00E+00 | 1.01E+04 | 1.54E+04 | 6.88E+03 | -- | -- |
| Smcn001947 | 6.23E+02 | 1.61E+02 | 6.24E+02 | C_29_H_36_O_15_ | [M-H]- | Verbascoside | Phenolic acids | Phenolic acids | 61276-17-3 | 2 | 2.28E+04 | 1.56E+04 | 2.02E+04 | 2.56E+04 | 3.72E+04 | 4.38E+04 | 9.00E+00 | 9.00E+00 | 9.00E+00 | 9.76E+03 | 7.90E+03 | 1.57E+04 | 2.21E+04 | 1.80E+04 | 2.14E+04 | C10501 | -- |
| pmp001310 | 6.27E+02 | 3.03E+02 | 6.26E+02 | C_27_H_30_O_17_ | [M+H]+ | 6-Hydroxykaempferol-3,6-O-Diglucoside | Flavonoids | Flavonols | - | 3 | 2.17E+04 | 3.56E+04 | 4.72E+04 | 9.00E+00 | 9.00E+00 | 9.00E+00 | 9.00E+00 | 9.00E+00 | 9.00E+00 | 9.00E+00 | 9.00E+00 | 9.00E+00 | 1.52E+04 | 1.38E+04 | 1.45E+04 | -- | -- |
| Lmtp003677 | 6.27E+02 | 3.03E+02 | 6.26E+02 | C_27_H_30_O_17_ | [M+H]+ | Quercetin-3-O-sophoroside (Baimaside) | Flavonoids | Flavonols | 18609-17-1 | 3 | 2.04E+04 | 2.79E+04 | 6.00E+04 | 9.00E+00 | 9.00E+00 | 9.00E+00 | 9.00E+00 | 9.00E+00 | 9.00E+00 | 9.00E+00 | 9.00E+00 | 9.00E+00 | 1.13E+04 | 1.89E+04 | 1.52E+04 | C12667 | ko00944 |
| Zmhp002730 | 6.27E+02 | 3.03E+02 | 6.26E+02 | C_27_H_30_O_17_ | [M+H]+ | 6-Hydroxykaempferol 6,7-Diglucoside | Flavonoids | Flavonols | 142674-16-6 | 2 | 2.02E+04 | 3.11E+04 | 4.62E+04 | 9.00E+00 | 9.00E+00 | 9.00E+00 | 9.00E+00 | 9.00E+00 | 9.00E+00 | 9.00E+00 | 9.00E+00 | 9.00E+00 | 1.32E+04 | 1.07E+04 | 7.91E+03 | -- | -- |
| pmb2653 | 6.49E+02 | 1.45E+02 | 6.50E+02 | C_24_H_42_O_20_ | [M-H]- | DMelezitose O-rhamnoside | Others | Saccharides and Alcohols | - | 3 | 2.15E+06 | 2.04E+06 | 2.11E+06 | 1.17E+06 | 1.05E+06 | 1.24E+06 | 2.17E+06 | 2.52E+06 | 2.66E+06 | 1.83E+06 | 1.78E+06 | 1.80E+06 | 1.71E+06 | 2.00E+06 | 1.88E+06 | -- | -- |
| pmb0530 | 6.64E+02 | 1.36E+02 | 6.63E+02 | C_21_H_27_N_7_O_14_P_2_ | [M+H]+ | Nicotinic acid adenine dinucleotide | Nucleotides and derivatives | Nucleotides and derivatives | 53-84-9 | 1 | 1.45E+04 | 1.75E+04 | 1.44E+04 | 3.36E+05 | 3.22E+05 | 3.33E+05 | 4.67E+04 | 7.00E+04 | 5.45E+04 | 1.85E+05 | 2.25E+05 | 2.16E+05 | 9.71E+04 | 1.51E+05 | 1.79E+05 | C00003 | ko00190,ko00730,ko00760,ko01100,ko01240 |
| mws4163 | 6.65E+02 | 4.85E+02 | 6.66E+02 | C_24_H_42_O_21_ | [M-H]- | Nystose | Others | Saccharides and Alcohols | 13133-07-8 | 3 | 8.17E+04 | 1.11E+05 | 6.89E+04 | 1.74E+04 | 2.98E+04 | 1.93E+04 | 4.76E+04 | 7.68E+04 | 6.06E+04 | 2.37E+04 | 1.98E+04 | 1.14E+04 | 3.10E+04 | 5.02E+04 | 3.56E+04 | -- | -- |
| mws1593 | 6.65E+02 | 1.61E+02 | 6.66E+02 | C_24_H_42_O_21_ | [M-H]- | D-Maltotetraose | Others | Saccharides and Alcohols | 34612-38-9 | 3 | 1.32E+05 | 1.44E+05 | 1.28E+05 | 4.18E+04 | 3.36E+04 | 4.69E+04 | 3.94E+04 | 3.81E+04 | 4.40E+04 | 2.80E+04 | 2.57E+04 | 4.13E+04 | 5.55E+04 | 8.16E+04 | 7.48E+04 | C02052 | -- |
| Lmyn005812 | 6.75E+02 | 3.97E+02 | 6.76E+02 | C_33_H_56_O_14_ | [M-H]- | Gingerglycolipid A | Lipids | Glycerol ester | 145937-22-0 | 1 | 2.64E+05 | 2.56E+05 | 2.91E+05 | 1.10E+06 | 8.54E+05 | 7.96E+05 | 1.62E+05 | 1.63E+05 | 1.86E+05 | 4.38E+05 | 4.36E+05 | 4.04E+05 | 5.09E+05 | 5.47E+05 | 4.53E+05 | -- | -- |
| pmp001276 | 6.77E+02 | 6.77E+02 | 6.76E+02 | C_33_H_56_O_14_ | [M+H]+ | 1-Linolenoyl-rac-glycerol-diglucoside | Lipids | Free fatty acids | - | 1 | 6.57E+05 | 7.35E+05 | 8.21E+05 | 2.51E+06 | 2.45E+06 | 2.66E+06 | 1.92E+06 | 2.13E+06 | 1.93E+06 | 3.05E+06 | 3.45E+06 | 3.34E+06 | 1.72E+06 | 2.25E+06 | 2.45E+06 | -- | -- |
| Lmhp008513 | 6.77E+02 | 2.61E+02 | 6.76E+02 | C_33_H_56_O_14_ | [M+H]+ | 2-α-Linolenoyl-glycerol-1,3-di-O-glucoside* | Lipids | Glycerol ester | - | 1 | 7.89E+03 | 8.19E+03 | 7.16E+03 | 3.36E+04 | 3.25E+04 | 2.43E+04 | 4.06E+03 | 6.73E+03 | 5.33E+03 | 1.28E+04 | 1.81E+04 | 1.98E+04 | 1.58E+04 | 1.31E+04 | 1.83E+04 | -- | -- |
| Lmhp008744 | 6.77E+02 | 2.61E+02 | 6.76E+02 | C_33_H_56_O_14_ | [M+H]+ | 1-α-Linolenoyl-glycerol-2,3-di-O-glucoside* | Lipids | Glycerol ester | - | 1 | 6.13E+03 | 8.20E+03 | 8.11E+03 | 2.19E+04 | 2.52E+04 | 2.42E+04 | 4.83E+03 | 6.57E+03 | 4.64E+03 | 1.24E+04 | 1.78E+04 | 1.87E+04 | 1.48E+04 | 2.08E+04 | 2.42E+04 | -- | -- |
| HJN102 | 6.77E+02 | 5.15E+02 | 6.78E+02 | C_34_H_30_O_15_ | [M-H]- | 3,4,5-Tricaffeoylquinic acid | Phenolic acids | Phenolic acids | 86632-03-3 | 2 | 5.64E+03 | 9.73E+03 | 9.78E+03 | 1.30E+05 | 1.31E+05 | 1.54E+05 | 2.42E+04 | 3.59E+04 | 3.07E+04 | 2.94E+04 | 3.56E+04 | 3.04E+04 | 5.07E+04 | 4.78E+04 | 5.06E+04 | -- | -- |
| Lmgn002250 | 6.77E+02 | 1.91E+02 | 6.78E+02 | C_31_H_34_O_17_ | [M-H]- | Dicaffeoylquinic acid-O-glucoside | Phenolic acids | Phenolic acids | - | 1 | 8.35E+04 | 4.98E+04 | 1.68E+05 | 1.06E+05 | 9.58E+04 | 1.11E+05 | 5.34E+04 | 5.61E+04 | 7.02E+04 | 5.83E+04 | 6.57E+04 | 5.78E+04 | 9.01E+04 | 1.17E+05 | 1.12E+05 | -- | -- |
| Lmhp009384 | 6.79E+02 | 2.63E+02 | 6.78E+02 | C_33_H_58_O_14_ | [M+H]+ | 1-Linoleoylglycerol-2,3-di-O-glucoside* | Lipids | Glycerol ester | - | 1 | 4.00E+04 | 3.08E+04 | 2.95E+04 | 8.65E+04 | 4.56E+04 | 9.38E+04 | 3.51E+04 | 3.03E+04 | 3.64E+04 | 7.44E+04 | 8.78E+04 | 7.66E+04 | 5.85E+04 | 6.14E+04 | 7.35E+04 | -- | -- |
| pmp001271 | 6.79E+02 | 2.63E+02 | 6.78E+02 | C_33_H_58_O_14_ | [M+H]+ | 1-Linoleoyl-sn-glycerol-diglucoside* | Lipids | Free fatty acids | - | 1 | 4.55E+04 | 3.63E+04 | 3.63E+04 | 8.88E+04 | 7.76E+04 | 6.74E+04 | 3.28E+04 | 3.17E+04 | 4.15E+04 | 7.98E+04 | 9.84E+04 | 8.19E+04 | 7.14E+04 | 7.79E+04 | 7.25E+04 | -- | -- |
| Lmyn006011 | 6.77E+02 | 3.97E+02 | 6.78E+02 | C_33_H_58_O_14_ | [M-H]- | Gingerglycolipid B | Lipids | Glycerol ester | 88168-90-5 | 1 | 1.71E+06 | 1.52E+06 | 1.62E+06 | 4.47E+06 | 4.13E+06 | 4.14E+06 | 1.94E+06 | 1.69E+06 | 1.83E+06 | 3.74E+06 | 4.70E+06 | 3.92E+06 | 3.56E+06 | 3.25E+06 | 4.17E+06 | -- | -- |
| Lmhp009190 | 6.79E+02 | 2.63E+02 | 6.78E+02 | C_33_H_58_O_14_ | [M+H]+ | 2-Linoleoylglycerol-1,3-di-O-glucoside* | Lipids | Glycerol ester | - | 1 | 2.80E+04 | 3.21E+04 | 4.01E+04 | 6.93E+04 | 7.02E+04 | 8.24E+04 | 3.40E+04 | 3.38E+04 | 3.86E+04 | 6.72E+04 | 1.68E+05 | 1.04E+05 | 6.94E+04 | 6.15E+04 | 6.70E+04 | -- | -- |
| Lmyn006221 | 6.79E+02 | 3.97E+02 | 6.80E+02 | C_33_H_60_O_14_ | [M-H]- | Gingerglycolipid C | Lipids | Glycerol ester | - | 2 | 5.95E+04 | 5.70E+04 | 5.99E+04 | 2.18E+05 | 2.05E+05 | 1.78E+05 | 1.03E+05 | 9.49E+04 | 8.45E+04 | 1.75E+05 | 2.16E+05 | 2.23E+05 | 1.43E+05 | 1.69E+05 | 1.35E+05 | -- | -- |
| Hmln001931 | 6.81E+02 | 3.57E+02 | 6.82E+02 | C_32_H_42_O_16_ | [M-H]- | Matairesinol-4,4'-di-O-glucoside | Lignans and Coumarins | Lignans | - | 3 | 4.13E+03 | 9.23E+03 | 4.08E+03 | 4.08E+03 | 4.58E+03 | 4.62E+03 | 1.26E+04 | 8.21E+03 | 1.44E+04 | 1.07E+04 | 9.25E+03 | 8.22E+03 | 4.09E+03 | 8.69E+03 | 1.03E+04 | -- | -- |
| pmn001370 | 6.81E+02 | 5.19E+02 | 6.82E+02 | C_32_H_42_O_16_ | [M-H]- | Pinoresinol-4,4'-O-di-O-glucoside | Lignans and Coumarins | Lignans | 63902-38-5 | 2 | 2.28E+05 | 2.15E+05 | 2.24E+05 | 4.77E+05 | 3.83E+05 | 4.36E+05 | 2.48E+05 | 2.16E+05 | 3.10E+05 | 2.97E+05 | 3.06E+05 | 2.70E+05 | 2.97E+05 | 3.33E+05 | 3.70E+05 | -- | -- |
| Lmgn002253 | 6.95E+02 | 3.53E+02 | 6.96E+02 | C_31_H_36_O_18_ | [M-H]- | Syringoylcaffeoylquinic acid-D-glucose | Phenolic acids | Phenolic acids | - | 2 | 2.20E+05 | 3.68E+05 | 2.35E+05 | 4.54E+05 | 3.96E+05 | 5.01E+05 | 3.95E+05 | 4.45E+05 | 4.52E+05 | 3.30E+05 | 3.56E+05 | 3.82E+05 | 3.66E+05 | 4.01E+05 | 3.07E+05 | -- | -- |
| pmn001368 | 6.99E+02 | 5.37E+02 | 7.00E+02 | C_32_H_44_O_17_ | [M-H]- | Olivil-4,4'-Di-O-glucoside | Lignans and Coumarins | Lignans | - | 3 | 5.27E+04 | 4.05E+04 | 4.22E+04 | 4.22E+04 | 3.70E+04 | 3.86E+04 | 2.99E+04 | 2.88E+04 | 3.65E+04 | 2.66E+04 | 2.28E+04 | 2.33E+04 | 3.53E+04 | 3.40E+04 | 4.09E+04 | -- | -- |
| pme2651 | 7.42E+02 | 6.20E+02 | 7.43E+02 | C_21_H_28_N_7_O_17_P_3_ | [M-H]- | NADP (Nicotinamide adenine dinucleotide phosphate) | Nucleotides and derivatives | Nucleotides and derivatives | 53-59-8 | 3 | 5.42E+03 | 1.97E+03 | 4.13E+03 | 6.79E+04 | 6.25E+04 | 6.79E+04 | 7.77E+03 | 5.36E+03 | 6.00E+03 | 2.89E+04 | 3.70E+04 | 4.51E+04 | 3.22E+04 | 3.21E+04 | 2.68E+04 | C00006 | ko00195,ko00480,ko00760,ko01100,ko01240 |
| pmn001505 | 7.63E+02 | 7.63E+02 | 7.64E+02 | C_41_H_64_O_13_ | [M-H]- | Oleanolic acid-3-O-xylosyl(1→3)glucuronide | Terpenoids | Triterpene Saponin | - | 2 | 9.59E+04 | 1.04E+05 | 7.88E+04 | 1.75E+05 | 1.56E+05 | 1.58E+05 | 6.81E+04 | 7.08E+04 | 6.84E+04 | 1.02E+05 | 1.10E+05 | 1.18E+05 | 1.30E+05 | 1.16E+05 | 1.40E+05 | -- | -- |
| Lmmn005092 | 9.55E+02 | 9.55E+02 | 9.56E+02 | C_47_H_72_O_20_ | [M-H]- | Medicagenic acid-3-O-glucuronide-28-O-rhamnosyl(1,2)-arabinoside | Terpenoids | Triterpene Saponin | - | 2 | 3.36E+04 | 4.28E+04 | 3.74E+04 | 1.07E+05 | 1.06E+05 | 1.01E+05 | 1.65E+04 | 1.37E+04 | 1.75E+04 | 2.06E+04 | 2.78E+04 | 2.85E+04 | 5.47E+04 | 5.13E+04 | 4.84E+04 | -- | -- |
| Lmmn005003 | 1.09E+03 | 1.09E+03 | 1.09E+03 | C_52_H_80_O_24_ | [M-H]- | Medicagenic acid-3-O-glucuronide-28-O-xylosyl(1,4)-rhamnosyl(1,2)-arabinoside | Terpenoids | Triterpene Saponin | 128192-15-4 | 3 | 7.73E+04 | 6.97E+04 | 7.73E+04 | 2.44E+05 | 2.31E+05 | 2.52E+05 | 7.53E+04 | 6.72E+04 | 6.82E+04 | 7.62E+04 | 9.46E+04 | 1.83E+05 | 1.17E+05 | 1.07E+05 | 1.17E+05 | -- | -- |

**Supplementary table S6** Differential metabolites in TJXD vs TJXH

| Index | Formula | Compounds | Class I | Class II | CAS | Level | TJXD-1 | TJXD-2 | TJXD-3 | TJXH-1 | TJXH-2 | TJXH-3 | VIP | p_value | FDR | Fold_Change | Log_2_FC | Type |
| --- | --- | --- | --- | --- | --- | --- | --- | --- | --- | --- | --- | --- | --- | --- | --- | --- | --- | --- |
| pme3200 | C_2_H_7_N_3_ | 1-Methylguanidine | Nucleotides and derivatives | Nucleotides and derivatives | 471-29-4 | 3 | 3.80E+03 | 2.89E+03 | 6.19E+03 | 3.65E+04 | 3.36E+04 | 3.26E+04 | 1.21E+00 | 4.86E-05 | 9.88E-04 | 7.96E+00 | 2.99E+00 | up |
| pme1841 | C_5_H_14_N_2_ | Cadaverine | Alkaloids | Alkaloids | 462-94-2 | 3 | 9.03E+04 | 1.07E+05 | 9.88E+04 | 7.17E+05 | 6.80E+05 | 7.03E+05 | 1.23E+00 | 4.07E-05 | 9.22E-04 | 7.10E+00 | 2.83E+00 | up |
| mws0601 | C_5_H_5_NO_2_ | Pyrrole-2-carboxylic acid | Organic acids | Organic acids | 634-97-9 | 3 | 1.37E+04 | 1.56E+04 | 1.25E+04 | 1.09E+05 | 1.05E+05 | 1.11E+05 | 1.23E+00 | 1.65E-05 | 7.62E-04 | 7.79E+00 | 2.96E+00 | up |
| mws0376 | C_4_H_4_O_4_ | Fumaric acid | Organic acids | Organic acids | 110-17-8 | 1 | 3.53E+05 | 3.63E+05 | 4.22E+05 | 1.42E+06 | 1.48E+06 | 1.24E+06 | 1.23E+00 | 2.89E-03 | 1.22E-02 | 3.64E+00 | 1.86E+00 | up |
| pme0120 | C_5_H_11_NO_2_ | 5-Aminovaleric acid | Organic acids | Organic acids | 660-88-8 | 3 | 3.40E+04 | 4.83E+04 | 5.07E+04 | 1.27E+05 | 1.62E+05 | 1.70E+05 | 1.20E+00 | 7.75E-03 | 2.33E-02 | 3.45E+00 | 1.79E+00 | up |
| MWSmce548 | C_5_H_11_NO_2_ | Betaine | Alkaloids | Alkaloids | 107-43-7 | 2 | 3.56E+05 | 3.62E+05 | 3.65E+05 | 4.03E+06 | 4.07E+06 | 4.11E+06 | 1.24E+00 | 3.55E-05 | 8.96E-04 | 1.13E+01 | 3.50E+00 | up |
| mws0192 | C_4_H_6_O_4_ | Succinic acid | Organic acids | Organic acids | 110-15-6 | 1 | 7.63E+05 | 6.34E+05 | 7.74E+05 | 1.74E+06 | 1.60E+06 | 1.74E+06 | 1.22E+00 | 1.06E-04 | 1.62E-03 | 2.34E+00 | 1.23E+00 | up |
| mws0470 | C_4_H_6_O_4_ | Methylmalonic acid | Organic acids | Organic acids | 516-05-2 | 1 | 6.69E+05 | 7.09E+05 | 7.18E+05 | 1.64E+06 | 1.73E+06 | 1.73E+06 | 1.23E+00 | 1.31E-04 | 1.92E-03 | 2.43E+00 | 1.28E+00 | up |
| pme2923 | C_4_H_6_O_4_ | Acetoxyacetic acid | Organic acids | Organic acids | 13831-30-6 | 3 | 9.00E+00 | 9.00E+00 | 9.00E+00 | 5.75E+05 | 5.65E+05 | 5.18E+05 | 1.24E+00 | 1.00E-03 | 7.05E-03 | 6.14E+04 | 1.59E+01 | up |
| pme3096 | C_3_H_5_NO_4_ | Aminomalonic acid | Organic acids | Organic acids | 1068-84-4 | 3 | 1.12E+06 | 1.10E+06 | 1.18E+06 | 2.70E+06 | 2.59E+06 | 2.69E+06 | 1.23E+00 | 9.83E-06 | 6.24E-04 | 2.35E+00 | 1.23E+00 | up |
| mws0628 | C_7_H_6_O_2_ | 4-Hydroxybenzaldehyde | Phenolic acids | Phenolic acids | 123-08-0 | 1 | 2.93E+05 | 3.31E+05 | 3.02E+05 | 1.18E+06 | 1.28E+06 | 1.32E+06 | 1.23E+00 | 9.69E-04 | 6.99E-03 | 4.09E+00 | 2.03E+00 | up |
| mws0133 | C_6_H_6_N_2_O | Nicotinamide | Others | Vitamin | 98-92-0 | 1 | 3.50E+06 | 3.48E+06 | 3.48E+06 | 9.42E+06 | 8.74E+06 | 9.59E+06 | 1.23E+00 | 2.05E-03 | 9.91E-03 | 2.65E+00 | 1.41E+00 | up |
| Lmbp000668 | C_6_H_5_NO_2_ | Isonicotinic acid | Others | Vitamin | 55-22-1 | 2 | 6.89E+05 | 7.14E+05 | 6.76E+05 | 2.70E+06 | 2.64E+06 | 2.95E+06 | 1.23E+00 | 1.94E-03 | 9.58E-03 | 3.99E+00 | 2.00E+00 | up |
| pme1216 | C_6_H_5_NO_2_ | 2-Picolinic acid | Organic acids | Organic acids | 98-98-6 | 3 | 3.55E+05 | 4.00E+05 | 3.63E+05 | 2.11E+06 | 2.02E+06 | 1.98E+06 | 1.23E+00 | 1.57E-04 | 2.02E-03 | 5.46E+00 | 2.45E+00 | up |
| pme0490 | C_6_H_5_NO_2_ | Nicotinic acid (Vitamin B3) | Others | Vitamin | 59-67-6 | 1 | 6.88E+05 | 7.01E+05 | 6.41E+05 | 2.70E+06 | 2.80E+06 | 2.78E+06 | 1.23E+00 | 4.07E-06 | 4.04E-04 | 4.08E+00 | 2.03E+00 | up |
| mws0572 | C_5_H_7_N_3_O | 5-Methylcytosine | Nucleotides and derivatives | Nucleotides and derivatives | 554-01-8 | 3 | 9.00E+00 | 9.00E+00 | 9.00E+00 | 3.10E+03 | 4.40E+03 | 4.42E+03 | 1.23E+00 | 1.19E-02 | 3.15E-02 | 4.42E+02 | 8.79E+00 | up |
| mws0251 | C_5_H_6_N_2_O_2_ | Thymine | Nucleotides and derivatives | Nucleotides and derivatives | 65-71-4 | 3 | 9.00E+00 | 9.00E+00 | 9.00E+00 | 3.15E+04 | 2.92E+04 | 3.21E+04 | 1.24E+00 | 8.15E-04 | 6.11E-03 | 3.44E+03 | 1.17E+01 | up |
| mws0263 | C_5_H_7_NO_3_ | 5-Oxo-L-Proline | Amino acids and derivatives | Amino acids and derivatives | 98-79-3 | 3 | 1.34E+04 | 2.13E+04 | 2.59E+04 | 1.04E+05 | 1.39E+05 | 8.44E+04 | 1.19E+00 | 2.58E-02 | 5.50E-02 | 5.41E+00 | 2.43E+00 | up |
| pme2693 | C_6_H_14_N_2_O | N-Acetylputrescine | Alkaloids | Alkaloids | 18233-70-0 | 3 | 2.53E+04 | 1.54E+04 | 2.63E+04 | 5.44E+04 | 6.66E+04 | 5.99E+04 | 1.16E+00 | 1.58E-03 | 8.79E-03 | 2.70E+00 | 1.43E+00 | up |
| pmb0501 | C_5_H_14_N_4_ | Agmatine | Alkaloids | Alkaloids | 306-60-5 | 3 | 1.60E+04 | 1.74E+04 | 2.29E+04 | 6.52E+04 | 6.14E+04 | 7.39E+04 | 1.21E+00 | 1.11E-03 | 7.18E-03 | 3.56E+00 | 1.83E+00 | up |
| Zmpn000638 | C_4_H_9_N_3_O_2_ | 3-Guanidinopropionic acid | Organic acids | Organic acids | 353-09-3 | 2 | 5.84E+04 | 6.27E+04 | 5.97E+04 | 1.71E+05 | 1.90E+05 | 1.91E+05 | 1.23E+00 | 1.90E-03 | 9.58E-03 | 3.06E+00 | 1.61E+00 | up |
| pme0243 | C_5_H_8_O_4_ | Glutaric acid | Organic acids | Organic acids | 110-94-1 | 3 | 6.43E+04 | 3.25E+04 | 7.77E+04 | 2.25E+05 | 2.56E+05 | 3.37E+05 | 1.16E+00 | 1.35E-02 | 3.47E-02 | 4.69E+00 | 2.23E+00 | up |
| MWS1882 | C_4_H_7_NO_4_ | Iminodiacetic acid | Organic acids | Organic acids | 142-73-4 | 2 | 4.94E+05 | 5.28E+05 | 4.43E+05 | 1.31E+05 | 1.36E+05 | 1.55E+05 | 1.23E+00 | 2.83E-03 | 1.21E-02 | 2.88E-01 | -1.79E+00 | down |
| MWSmce609 | C_4_H_7_NO_4_ | L-Aspartic acid | Amino acids and derivatives | Amino acids and derivatives | 56-84-8 | 2 | 6.92E+05 | 6.82E+05 | 5.39E+05 | 1.96E+05 | 2.15E+05 | 2.16E+05 | 1.22E+00 | 1.19E-02 | 3.15E-02 | 3.28E-01 | -1.61E+00 | down |
| mws5028 | C_7_H_5_NO_2_ | 2-Benzoxazolinone | Others | Others | 59-49-4 | 2 | 9.00E+00 | 9.00E+00 | 9.00E+00 | 5.48E+04 | 4.44E+04 | 5.03E+04 | 1.24E+00 | 3.67E-03 | 1.44E-02 | 5.54E+03 | 1.24E+01 | up |
| pmb0374 | C_5_H_5_N_5_ | 2-Aminopurine | Nucleotides and derivatives | Nucleotides and derivatives | 452-06-2 | 1 | 1.76E+05 | 1.82E+05 | 1.53E+05 | 3.62E+05 | 3.82E+05 | 3.47E+05 | 1.22E+00 | 1.46E-04 | 1.99E-03 | 2.13E+00 | 1.09E+00 | up |
| pme0033 | C_5_H_4_N_4_O | Hypoxanthine | Nucleotides and derivatives | Nucleotides and derivatives | 68-94-0 | 3 | 1.20E+04 | 1.97E+04 | 1.16E+04 | 1.49E+05 | 1.44E+05 | 1.65E+05 | 1.22E+00 | 4.86E-04 | 4.53E-03 | 1.06E+01 | 3.40E+00 | up |
| MWSmce466 | C_8_H_8_O_2_ | 4'-Hydroxyacetophenone | Phenolic acids | Phenolic acids | 99-93-4 | 1 | 2.98E+04 | 4.64E+04 | 4.74E+04 | 8.62E+04 | 1.06E+05 | 7.68E+04 | 1.13E+00 | 1.33E-02 | 3.43E-02 | 2.18E+00 | 1.12E+00 | up |
| MWS1848 | C_8_H_8_O_2_ | Phenyl acetate | Phenolic acids | Phenolic acids | 122-79-2 | 1 | 5.00E+04 | 4.96E+04 | 4.40E+04 | 1.19E+05 | 1.05E+05 | 1.21E+05 | 1.22E+00 | 2.52E-03 | 1.12E-02 | 2.40E+00 | 1.26E+00 | up |
| Lmbn001981 | C_7_H_6_O_3_ | 2,5-Dihydroxybenzaldehyde | Phenolic acids | Phenolic acids | 1194-98-5 | 2 | 2.52E+05 | 2.80E+05 | 2.19E+05 | 6.70E+05 | 6.73E+05 | 6.13E+05 | 1.22E+00 | 1.17E-04 | 1.75E-03 | 2.61E+00 | 1.38E+00 | up |
| mws0749 | C_7_H_6_O_3_ | 4-Hydroxybenzoic acid | Phenolic acids | Phenolic acids | 99-96-7 | 2 | 3.23E+05 | 3.77E+05 | 3.38E+05 | 9.54E+05 | 8.94E+05 | 9.52E+05 | 1.23E+00 | 2.77E-05 | 8.03E-04 | 2.70E+00 | 1.43E+00 | up |
| Hmgn001653 | C_7_H_6_O_3_ | Protocatechualdehyde | Phenolic acids | Phenolic acids | 139-85-5 | 2 | 1.99E+05 | 2.00E+05 | 1.90E+05 | 5.02E+05 | 4.88E+05 | 4.83E+05 | 1.23E+00 | 2.04E-05 | 7.62E-04 | 2.50E+00 | 1.32E+00 | up |
| mws2368 | C_8_H_10_O_2_ | Tyrosol | Phenolic acids | Phenolic acids | 501-94-0 | 2 | 5.42E+03 | 5.47E+03 | 6.02E+03 | 1.94E+04 | 1.50E+04 | 1.99E+04 | 1.22E+00 | 1.39E-02 | 3.54E-02 | 3.21E+00 | 1.68E+00 | up |
| mws0704 | C_2_H_8_NO_4_P | O-Phosphorylethanolamine | Alkaloids | Alkaloids | 1071-23-4 | 3 | 1.73E+04 | 2.32E+04 | 2.20E+04 | 4.06E+04 | 4.16E+04 | 4.46E+04 | 1.19E+00 | 1.19E-03 | 7.31E-03 | 2.03E+00 | 1.02E+00 | up |
| Rfmb320 | C_7_H_13_NO_2_ | 1-Methylpiperidine-2-carboxylic acid | Organic acids | Organic acids | 7730-87-2 | 3 | 4.81E+04 | 2.43E+04 | 4.37E+04 | 9.86E+04 | 1.04E+05 | 1.29E+05 | 1.14E+00 | 4.34E-03 | 1.60E-02 | 2.85E+00 | 1.51E+00 | up |
| MWS3020 | C_7_H_16_N_2_O | N-Acetylcadaverine | Alkaloids | Alkaloids | 32343-73-0 | 2 | 2.69E+04 | 3.24E+04 | 3.64E+04 | 1.36E+05 | 1.35E+05 | 1.45E+05 | 1.23E+00 | 2.32E-05 | 7.62E-04 | 4.34E+00 | 2.12E+00 | up |
| pme0295 | C_6_H_11_NO_3_ | 4-Acetamidobutyric acid | Organic acids | Organic acids | 3025-96-5 | 3 | 2.83E+05 | 2.98E+05 | 3.01E+05 | 6.20E+06 | 6.24E+06 | 6.04E+06 | 1.24E+00 | 9.15E-05 | 1.45E-03 | 2.10E+01 | 4.39E+00 | up |
| mws0567 | C_5_H_11_N_3_O_2_ | 4-Guanidinobutyric acid | Organic acids | Organic acids | 463-00-3 | 1 | 1.87E+06 | 2.08E+06 | 1.92E+06 | 1.25E+07 | 1.24E+07 | 1.26E+07 | 1.23E+00 | 3.49E-08 | 3.32E-05 | 6.39E+00 | 2.68E+00 | up |
| pme3146 | C_5_H_10_N_2_O_3_ | β-Ureidoisobutyric acid | Organic acids | Organic acids | 2905-86-4 | 3 | 9.00E+00 | 9.00E+00 | 9.00E+00 | 3.13E+04 | 2.45E+04 | 2.63E+04 | 1.24E+00 | 5.51E-03 | 1.85E-02 | 3.04E+03 | 1.16E+01 | up |
| Zmzn000113 | C_5_H_9_NO_4_ | L-threo-3-Methylaspartate | Amino acids and derivatives | Amino acids and derivatives | 6061-13-8 | 3 | 4.91E+05 | 5.70E+05 | 5.51E+05 | 1.06E+05 | 1.18E+05 | 8.78E+04 | 1.23E+00 | 1.10E-03 | 7.18E-03 | 1.93E-01 | -2.37E+00 | down |
| mws1024 | C_9_H_8_O_2_ | p-Coumaraldehyde | Phenolic acids | Phenolic acids | 2538-87-6 | 3 | 9.00E+00 | 9.00E+00 | 9.00E+00 | 2.50E+04 | 2.40E+04 | 2.26E+04 | 1.24E+00 | 8.00E-04 | 6.04E-03 | 2.65E+03 | 1.14E+01 | up |
| MWS20194 | C_9_H_8_O_2_ | Cinnamic acid | Phenolic acids | Phenolic acids | 140-10-3 | 1 | 1.24E+05 | 1.41E+05 | 1.30E+05 | 1.93E+06 | 1.94E+06 | 2.11E+06 | 1.23E+00 | 8.80E-04 | 6.44E-03 | 1.52E+01 | 3.92E+00 | up |
| pme2987 | C_9_H_8_O_2_ | 3,4-Dihydrocoumarin | Lignans and Coumarins | Coumarins | 119-84-6 | 2 | 9.00E+00 | 9.00E+00 | 9.00E+00 | 8.99E+03 | 7.34E+03 | 1.03E+04 | 1.24E+00 | 9.40E-03 | 2.65E-02 | 9.88E+02 | 9.95E+00 | up |
| pme3154 | C_6_H_12_O_4_ | Mevalonic acid | Organic acids | Organic acids | 150-97-0 | 2 | 9.00E+00 | 9.00E+00 | 9.00E+00 | 1.96E+05 | 1.46E+05 | 2.12E+05 | 1.24E+00 | 1.14E-02 | 3.04E-02 | 2.05E+04 | 1.43E+01 | up |
| mws0489 | C_8_H_6_O_3_ | Benzoylformic acid | Organic acids | Organic acids | 611-73-4 | 3 | 9.00E+00 | 9.00E+00 | 9.00E+00 | 4.23E+04 | 6.86E+04 | 6.73E+04 | 1.23E+00 | 2.01E-02 | 4.64E-02 | 6.60E+03 | 1.27E+01 | up |
| mws0921 | C_9_H_10_O_2_ | p-Coumaryl alcohol | Phenolic acids | Phenolic acids | 3690-05-9 | 3 | 5.85E+03 | 4.31E+03 | 5.29E+03 | 4.82E+04 | 4.87E+04 | 6.93E+04 | 1.23E+00 | 1.83E-02 | 4.30E-02 | 1.08E+01 | 3.43E+00 | up |
| mws0008 | C_9_H_10_O_2_ | Hydrocinnamic acid | Phenolic acids | Phenolic acids | 501-52-0 | 3 | 9.00E+00 | 9.00E+00 | 9.00E+00 | 2.67E+04 | 1.57E+04 | 2.57E+04 | 1.23E+00 | 2.33E-02 | 5.08E-02 | 2.52E+03 | 1.13E+01 | up |
| pme1109 | C_5_H_5_N_5_O | Guanine | Nucleotides and derivatives | Nucleotides and derivatives | 73-40-5 | 2 | 1.19E+05 | 1.28E+05 | 1.22E+05 | 9.24E+05 | 8.29E+05 | 8.38E+05 | 1.23E+00 | 1.56E-03 | 8.73E-03 | 7.01E+00 | 2.81E+00 | up |
| pme0256 | C_5_H_4_N_4_O_2_ | Xanthine | Nucleotides and derivatives | Nucleotides and derivatives | 69-89-6 | 1 | 9.00E+00 | 9.00E+00 | 9.00E+00 | 1.43E+05 | 1.31E+05 | 1.42E+05 | 1.24E+00 | 8.00E-04 | 6.04E-03 | 1.54E+04 | 1.39E+01 | up |
| MWS2984 | C_4_H_4_N_6_O | 8-Azaguanine | Nucleotides and derivatives | Nucleotides and derivatives | 134-58-7 | 2 | 1.84E+04 | 2.13E+04 | 1.82E+04 | 1.42E+05 | 1.29E+05 | 1.46E+05 | 1.23E+00 | 1.18E-03 | 7.31E-03 | 7.20E+00 | 2.85E+00 | up |
| mws0182 | C_8_H_8_O_3_ | p-Hydroxyphenyl acetic acid | Phenolic acids | Phenolic acids | 156-38-7 | 3 | 9.00E+00 | 9.00E+00 | 9.00E+00 | 2.04E+05 | 1.77E+05 | 1.72E+05 | 1.24E+00 | 2.91E-03 | 1.22E-02 | 2.05E+04 | 1.43E+01 | up |
| pme2362 | C_8_H_8_O_3_ | Mandelic acid | Phenolic acids | Phenolic acids | 90-64-2 | 3 | 2.42E+04 | 2.58E+04 | 2.51E+04 | 1.54E+05 | 1.27E+05 | 1.11E+05 | 1.23E+00 | 1.35E-02 | 3.47E-02 | 5.22E+00 | 2.38E+00 | up |
| mws0444 | C_7_H_7_NO_3_ | 3-Aminosalicylic acid | Phenolic acids | Phenolic acids | 570-23-0 | 3 | 9.00E+00 | 9.00E+00 | 9.00E+00 | 6.01E+04 | 4.94E+04 | 4.02E+04 | 1.24E+00 | 1.30E-02 | 3.39E-02 | 5.54E+03 | 1.24E+01 | up |
| Hmlp000935 | C_8_H_11_NO_2_ | Vanillylamine | Alkaloids | Phenolamine | 1196-92-5 | 2 | 9.00E+00 | 9.00E+00 | 9.00E+00 | 1.18E+04 | 1.31E+04 | 1.41E+04 | 1.24E+00 | 2.77E-03 | 1.21E-02 | 1.44E+03 | 1.05E+01 | up |
| mws0180 | C_7_H_6_O_4_ | 2,5-Dihydroxybenzoic acid; Gentisic Acid* | Phenolic acids | Phenolic acids | 490-79-9 | 2 | 4.95E+04 | 7.41E+04 | 7.31E+04 | 2.21E+06 | 1.94E+06 | 2.20E+06 | 1.23E+00 | 1.70E-03 | 9.02E-03 | 3.23E+01 | 5.01E+00 | up |
| mws0183 | C_7_H_6_O_4_ | 3,4-Dihydroxybenzoic acid (Protocatechuic acid)* | Phenolic acids | Phenolic acids | 99-50-3 | 2 | 9.37E+04 | 1.26E+05 | 1.13E+05 | 3.48E+06 | 3.27E+06 | 3.97E+06 | 1.23E+00 | 3.55E-03 | 1.41E-02 | 3.23E+01 | 5.01E+00 | up |
| mws0639 | C_7_H_6_O_4_ | 2,3-Dihydroxybenzoic Acid* | Phenolic acids | Phenolic acids | 303-38-8 | 2 | 1.35E+05 | 1.41E+05 | 7.91E+04 | 9.00E+00 | 9.00E+00 | 9.00E+00 | 1.23E+00 | 2.64E-02 | 5.58E-02 | 7.62E-05 | -1.37E+01 | down |
| mws0005 | C_10_H_12_N_2_ | Tryptamine | Alkaloids | Plumerane | 61-54-1 | 2 | 7.83E+04 | 8.89E+04 | 6.61E+04 | 2.11E+05 | 1.78E+05 | 2.27E+05 | 1.20E+00 | 4.94E-03 | 1.73E-02 | 2.64E+00 | 1.40E+00 | up |
| mws1417 | C_9_H_7_NO_2_ | Indole-3-carboxylic acid* | Alkaloids | Plumerane | 771-50-6 | 2 | 9.40E+04 | 1.29E+05 | 1.23E+05 | 3.18E+05 | 3.27E+05 | 3.36E+05 | 1.21E+00 | 4.71E-04 | 4.44E-03 | 2.84E+00 | 1.50E+00 | up |
| mws0102 | C_9_H_7_NO_2_ | Indole-5-carboxylic acid* | Alkaloids | Plumerane | 1670-81-1 | 2 | 9.46E+04 | 1.22E+05 | 1.02E+05 | 3.94E+05 | 2.98E+05 | 2.95E+05 | 1.21E+00 | 1.59E-02 | 3.88E-02 | 3.10E+00 | 1.63E+00 | up |
| mws1346 | C_6_H_11_NO_4_ | DL-2-Aminoadipic acid | Alkaloids | Alkaloids | 542-32-5 | 3 | 1.79E+05 | 1.25E+05 | 1.67E+05 | 3.33E+05 | 3.89E+05 | 3.84E+05 | 1.19E+00 | 9.87E-04 | 7.01E-03 | 2.35E+00 | 1.23E+00 | up |
| pme3382 | C_6_H_11_NO_4_ | N-Acetyl-L-threonine | Amino acids and derivatives | Amino acids and derivatives | 17093-74-2 | 3 | 3.75E+04 | 4.36E+04 | 3.00E+04 | 8.64E+04 | 8.33E+04 | 8.22E+04 | 1.19E+00 | 3.91E-03 | 1.49E-02 | 2.27E+00 | 1.18E+00 | up |
| mws1320 | C_10_H_11_NO | Tryptophol | Alkaloids | Plumerane | 526-55-6 | 1 | 9.00E+00 | 9.00E+00 | 9.00E+00 | 1.95E+04 | 1.93E+04 | 1.93E+04 | 1.24E+00 | 1.73E-05 | 7.62E-04 | 2.15E+03 | 1.11E+01 | up |
| Lmqp008175 | C_10_H_10_O_2_ | Methyl Cinnamate | Phenolic acids | Phenolic acids | 103-26-4 | 2 | 1.26E+06 | 1.19E+06 | 1.08E+06 | 4.93E+05 | 4.51E+05 | 6.51E+05 | 1.18E+00 | 1.44E-03 | 8.19E-03 | 4.52E-01 | -1.14E+00 | down |
| Lmbp000123 | C_6_H_13_NO_2_S | L-Homomethionine | Amino acids and derivatives | Amino acids and derivatives | 25148-30-5 | 3 | 5.12E+05 | 4.68E+05 | 5.36E+05 | 7.75E+04 | 7.65E+04 | 8.78E+04 | 1.23E+00 | 1.64E-03 | 8.89E-03 | 1.59E-01 | -2.65E+00 | down |
| pme1439 | C_9_H_8_O_3_ | p-Coumaric acid | Phenolic acids | Phenolic acids | 501-98-4 | 3 | 9.96E+04 | 1.55E+05 | 7.57E+04 | 4.03E+05 | 3.50E+05 | 4.18E+05 | 1.17E+00 | 9.11E-04 | 6.62E-03 | 3.55E+00 | 1.83E+00 | up |
| Lmmn001643 | C_9_H_8_O_3_ | 2-Hydroxycinnamic acid | Phenolic acids | Phenolic acids | 583-17-5 | 1 | 9.25E+05 | 1.01E+06 | 9.71E+05 | 5.71E+06 | 7.42E+06 | 7.07E+06 | 1.23E+00 | 8.05E-03 | 2.38E-02 | 6.95E+00 | 2.80E+00 | up |
| Lmmn004032 | C_11_H_16_O | Cis-Jasmone | Others | Others | 488-10-8 | 2 | 5.25E+03 | 5.82E+03 | 3.41E+03 | 1.60E+04 | 1.36E+04 | 2.01E+04 | 1.18E+00 | 1.56E-02 | 3.82E-02 | 3.43E+00 | 1.78E+00 | up |
| pme3083 | C_8_H_7_NO_3_ | 2-(Formylamino)benzoic acid | Phenolic acids | Phenolic acids | 3342-77-6 | 3 | 1.46E+05 | 1.68E+05 | 1.77E+05 | 9.48E+05 | 9.55E+05 | 9.85E+05 | 1.23E+00 | 9.13E-07 | 2.59E-04 | 5.88E+00 | 2.56E+00 | up |
| mws0467 | C_9_H_10_O_3_ | 3-(4-Hydroxyphenyl)-propionic acid | Phenolic acids | Phenolic acids | 501-97-3 | 2 | 4.17E+04 | 5.71E+04 | 4.91E+04 | 1.05E+05 | 1.09E+05 | 1.07E+05 | 1.20E+00 | 4.02E-03 | 1.52E-02 | 2.17E+00 | 1.12E+00 | up |
| mws2125 | C_3_H_5_O_6_P | Phosphoenolpyruvate | Organic acids | Organic acids | 138-08-9 | 3 | 1.89E+05 | 1.81E+05 | 2.11E+05 | 7.19E+04 | 8.87E+04 | 7.99E+04 | 1.22E+00 | 1.43E-03 | 8.19E-03 | 4.14E-01 | -1.27E+00 | down |
| MWStz091 | C_8_H_12_N_2_O_2_ | Cyclo(L-Ala-L-Pro) | Amino acids and derivatives | Amino acids and derivatives | 36357-32-1 | 2 | 9.00E+00 | 9.00E+00 | 9.00E+00 | 3.58E+04 | 3.28E+04 | 4.05E+04 | 1.24E+00 | 3.76E-03 | 1.46E-02 | 4.04E+03 | 1.20E+01 | up |
| pme1383 | C_8_H_11_NO_3_ | Pyridoxine | Others | Vitamin | 65-23-6 | 3 | 6.60E+04 | 5.80E+04 | 6.32E+04 | 3.19E+05 | 3.58E+05 | 3.41E+05 | 1.23E+00 | 1.15E-03 | 7.24E-03 | 5.44E+00 | 2.44E+00 | up |
| Zmzn000078 | C_3_H_7_O_6_P | Dihydroxyacetone phosphate | Others | Saccharides and Alcohols | 57-04-5 | 3 | 4.88E+04 | 3.95E+04 | 4.32E+04 | 1.46E+04 | 1.01E+04 | 2.18E+03 | 1.04E+00 | 2.07E-03 | 9.91E-03 | 2.04E-01 | -2.29E+00 | down |
| mws0024 | C_7_H_6_O_5_ | Gallic acid | Phenolic acids | Phenolic acids | 149-91-7 | 3 | 9.00E+00 | 9.00E+00 | 9.00E+00 | 3.29E+05 | 2.79E+05 | 2.95E+05 | 1.24E+00 | 2.33E-03 | 1.06E-02 | 3.35E+04 | 1.50E+01 | up |
| ML10176345 | C_7_H_8_O_5_ | 3-Dehydroshikimic acid | Organic acids | Organic acids | 2922-42-1 | 3 | 8.71E+03 | 5.80E+03 | 9.91E+03 | 9.00E+00 | 9.00E+00 | 9.00E+00 | 1.23E+00 | 2.18E-02 | 4.90E-02 | 1.11E-03 | -9.82E+00 | down |
| Lmbn003524 | C_9_H_16_O_3_ | 9-Oxononanoic acid | Organic acids | Organic acids | 2553-17-5 | 3 | 2.77E+04 | 2.02E+04 | 2.37E+04 | 9.66E+04 | 9.45E+04 | 9.68E+04 | 1.23E+00 | 3.02E-04 | 3.38E-03 | 4.01E+00 | 2.01E+00 | up |
| pme0253 | C_8_H_15_NO_3_ | N-Acetyl-L-leucine | Amino acids and derivatives | Amino acids and derivatives | 1188-21-2 | 3 | 1.71E+04 | 6.32E+03 | 1.49E+04 | 4.81E+05 | 3.53E+05 | 4.46E+05 | 1.22E+00 | 7.96E-03 | 2.37E-02 | 3.34E+01 | 5.06E+00 | up |
| MA10039492 | C_6_H_6_O_6_ | Dehydroascorbic acid | Others | Vitamin | 490-83-5 | 3 | 2.07E+04 | 9.18E+03 | 1.50E+04 | 9.00E+00 | 9.00E+00 | 9.00E+00 | 1.23E+00 | 4.62E-02 | 8.60E-02 | 6.01E-04 | -1.07E+01 | down |
| pme1651 | C_10_H_9_NO_2_ | Indole 3-acetic acid (IAA) | Alkaloids | Plumerane | 87-51-4 | 3 | 4.19E+04 | 5.56E+04 | 5.12E+04 | 4.07E+05 | 4.24E+05 | 3.93E+05 | 1.23E+00 | 8.35E-05 | 1.39E-03 | 8.23E+00 | 3.04E+00 | up |
| mws1075 | C_10_H_8_O_3_ | 7-Methoxycoumarin | Lignans and Coumarins | Coumarins | 531-59-9 | 3 | 1.21E+04 | 1.15E+04 | 1.00E+04 | 5.07E+04 | 3.09E+04 | 6.03E+04 | 1.19E+00 | 5.20E-02 | 9.38E-02 | 4.22E+00 | 2.08E+00 | up |
| Zmgn001448 | C_7_H_12_O_5_ | 2-Propylmalic Acid* | Organic acids | Organic acids | - | 1 | 6.00E+05 | 5.95E+05 | 6.16E+05 | 2.66E+06 | 2.47E+06 | 2.69E+06 | 1.23E+00 | 1.07E-03 | 7.18E-03 | 4.32E+00 | 2.11E+00 | up |
| pmb3101 | C_7_H_12_O_5_ | 2-Isopropylmalic Acid | Organic acids | Organic acids | 49601-06-1 | 1 | 7.83E+05 | 9.23E+05 | 8.50E+05 | 3.41E+06 | 3.58E+06 | 3.58E+06 | 1.23E+00 | 8.43E-06 | 6.18E-04 | 4.13E+00 | 2.05E+00 | up |
| Lmbn001754 | C_7_H_12_O_5_ | 3-Isopropylmalic Acid* | Organic acids | Organic acids | 921-28-8 | 1 | 7.30E+05 | 7.93E+05 | 8.12E+05 | 3.42E+06 | 3.16E+06 | 3.22E+06 | 1.23E+00 | 4.10E-04 | 4.19E-03 | 4.20E+00 | 2.07E+00 | up |
| mws1195 | C_10_H_10_O_3_ | p-Coumaric acid methyl ester | Phenolic acids | Phenolic acids | 3943-97-3 | 2 | 4.98E+04 | 6.78E+04 | 5.37E+04 | 1.76E+05 | 1.99E+05 | 2.10E+05 | 1.22E+00 | 1.11E-03 | 7.18E-03 | 3.41E+00 | 1.77E+00 | up |
| mws0009 | C_10_H_10_O_3_ | Coniferaldehyde | Phenolic acids | Phenolic acids | 20649-42-7 | 1 | 2.51E+04 | 2.32E+04 | 3.34E+04 | 8.62E+04 | 7.35E+04 | 9.60E+04 | 1.20E+00 | 4.62E-03 | 1.66E-02 | 3.13E+00 | 1.65E+00 | up |
| Jmzn006005 | C_10_H_10_O_3_ | 3,4-Methylenedioxy cinnamyl alcohol | Lignans and Coumarins | Lignans | 58095-76-4 | 3 | 1.01E+05 | 1.19E+05 | 9.87E+04 | 3.68E+05 | 4.00E+05 | 4.00E+05 | 1.23E+00 | 1.00E-04 | 1.56E-03 | 3.66E+00 | 1.87E+00 | up |
| pmb2795 | C_10_H_10_O_3_ | 4-Methoxycinnamic acid | Phenolic acids | Phenolic acids | 830-09-1 | 3 | 4.99E+04 | 5.73E+04 | 4.93E+04 | 1.71E+05 | 1.96E+05 | 2.00E+05 | 1.23E+00 | 2.23E-03 | 1.03E-02 | 3.62E+00 | 1.86E+00 | up |
| mws0093 | C_10_H_12_O_3_ | Coniferyl alcohol | Phenolic acids | Phenolic acids | 458-35-5 | 3 | 3.51E+04 | 3.68E+04 | 6.31E+04 | 1.73E+05 | 1.91E+05 | 1.95E+05 | 1.19E+00 | 3.65E-04 | 3.94E-03 | 4.15E+00 | 2.05E+00 | up |
| Hmtn001288 | C_9_H_10_O_4_ | Methyl 2,4-dihydroxyphenylacetate | Phenolic acids | Phenolic acids | 67828-42-6 | 3 | 9.00E+00 | 9.00E+00 | 9.00E+00 | 7.08E+04 | 6.81E+04 | 7.03E+04 | 1.24E+00 | 1.43E-04 | 1.99E-03 | 7.75E+03 | 1.29E+01 | up |
| Lmrn001951 | C_9_H_10_O_4_ | (S)-2-Hydroxy-3-(4-Hydroxyphenyl)Propanoic Acid | Phenolic acids | Phenolic acids | 23508-35-2 | 3 | 9.00E+00 | 9.00E+00 | 9.00E+00 | 5.43E+04 | 4.22E+04 | 4.40E+04 | 1.24E+00 | 6.35E-03 | 2.01E-02 | 5.20E+03 | 1.23E+01 | up |
| mws1350 | C_9_H_10_O_4_ | Syringaldehyde; 4-Hydroxy-3,5-Dimethoxybenzaldehyde | Phenolic acids | Phenolic acids | 134-96-3 | 3 | 9.00E+00 | 9.00E+00 | 9.00E+00 | 1.40E+04 | 1.79E+04 | 1.67E+04 | 1.24E+00 | 4.98E-03 | 1.74E-02 | 1.80E+03 | 1.08E+01 | up |
| pme0137 | C_7_H_12_N_2_O_4_ | N-Acetyl-L-Glutamine | Amino acids and derivatives | Amino acids and derivatives | 2490-97-3 | 3 | 9.00E+00 | 9.00E+00 | 9.00E+00 | 1.16E+05 | 1.07E+05 | 1.23E+05 | 1.24E+00 | 1.66E-03 | 8.92E-03 | 1.28E+04 | 1.36E+01 | up |
| Zmtn001624 | C_10_H_7_NO_3_ | N-Acetylisatin | Alkaloids | Plumerane | 574-17-4 | 3 | 2.94E+04 | 2.81E+04 | 1.57E+04 | 5.27E+04 | 3.74E+04 | 6.30E+04 | 1.03E+00 | 4.91E-02 | 8.91E-02 | 2.09E+00 | 1.07E+00 | up |
| pme2743 | C_10_H_11_NO_3_ | N-Phenylacetylglycine | Amino acids and derivatives | Amino acids and derivatives | 500-98-1 | 3 | 9.00E+00 | 9.00E+00 | 9.00E+00 | 3.31E+04 | 4.52E+04 | 4.26E+04 | 1.24E+00 | 8.28E-03 | 2.42E-02 | 4.48E+03 | 1.21E+01 | up |
| Lmdn003756 | C_10_H_10_O_4_ | Methyl caffeate | Phenolic acids | Phenolic acids | 3843-74-1 | 1 | 7.82E+03 | 1.23E+04 | 4.87E+03 | 9.00E+00 | 9.00E+00 | 9.00E+00 | 1.23E+00 | 6.11E-02 | 1.07E-01 | 1.08E-03 | -9.85E+00 | down |
| mws0014 | C_10_H_10_O_4_ | Ferulic acid | Phenolic acids | Phenolic acids | 537-98-4 | 1 | 1.39E+06 | 1.38E+06 | 1.40E+06 | 2.87E+06 | 2.79E+06 | 4.55E+06 | 1.16E+00 | 7.22E-02 | 1.22E-01 | 2.45E+00 | 1.29E+00 | up |
| Lmrj002244 | C_10_H_14_N_2_O_2_ | Cyclo(Pro-Pro) | Amino acids and derivatives | Amino acids and derivatives | 6708-06-1 | 3 | 4.17E+04 | 3.95E+04 | 5.17E+04 | 1.93E+05 | 1.65E+05 | 1.49E+05 | 1.22E+00 | 7.05E-03 | 2.19E-02 | 3.82E+00 | 1.93E+00 | up |
| MWSmce388 | C_11_H_6_O_4_ | Xanthotoxol | Lignans and Coumarins | Coumarins | 2009-24-7 | 2 | 4.28E+06 | 4.68E+06 | 4.40E+06 | 9.71E+06 | 9.25E+06 | 1.00E+07 | 1.23E+00 | 2.67E-04 | 3.03E-03 | 2.17E+00 | 1.12E+00 | up |
| MWSmce082 | C_11_H_10_O_4_ | Scoparone | Lignans and Coumarins | Coumarins | 120-08-1 | 1 | 2.85E+04 | 2.66E+04 | 3.74E+04 | 1.64E+05 | 1.75E+05 | 1.84E+05 | 1.23E+00 | 1.50E-04 | 1.99E-03 | 5.66E+00 | 2.50E+00 | up |
| Zmgn002106 | C_11_H_13_NO_3_ | N-Acetyl-L-phenylalanine | Amino acids and derivatives | Amino acids and derivatives | 2018-61-3 | 1 | 9.00E+00 | 9.00E+00 | 9.00E+00 | 7.22E+04 | 7.73E+04 | 8.04E+04 | 1.24E+00 | 9.81E-04 | 7.01E-03 | 8.51E+03 | 1.31E+01 | up |
| Hmcp003783 | C_13_H_10_N_2_O | 1-Acetyl-β-carboline | Alkaloids | Plumerane | - | 1 | 6.10E+04 | 6.30E+04 | 6.88E+04 | 1.51E+05 | 1.55E+05 | 1.50E+05 | 1.23E+00 | 4.45E-05 | 9.64E-04 | 2.37E+00 | 1.24E+00 | up |
| Lmhp002764 | C_11_H_18_N_2_O_2_ | Cyclo(Pro-Leu) | Amino acids and derivatives | Amino acids and derivatives | 5654-86-4 | 3 | 9.00E+00 | 9.00E+00 | 9.00E+00 | 3.86E+04 | 4.18E+04 | 1.13E+04 | 1.23E+00 | 8.76E-02 | 1.42E-01 | 3.39E+03 | 1.17E+01 | up |
| pmb2507 | C_5_H_11_O_7_P | 2-Deoxyribose-1-phosphate | Nucleotides and derivatives | Nucleotides and derivatives | 17210-42-3 | 3 | 4.44E+06 | 3.91E+06 | 3.07E+06 | 1.15E+07 | 1.09E+07 | 1.09E+07 | 1.21E+00 | 6.44E-04 | 5.43E-03 | 2.91E+00 | 1.54E+00 | up |
| mws0863 | C_5_H_11_O_7_P | 2-Deoxyribose-5'-phosphate | Nucleotides and derivatives | Nucleotides and derivatives | 102916-66-5 | 3 | 7.09E+04 | 7.35E+04 | 6.12E+04 | 1.47E+05 | 2.06E+05 | 2.01E+05 | 1.20E+00 | 2.21E-02 | 4.92E-02 | 2.69E+00 | 1.43E+00 | up |
| pme0170 | C_8_H_16_N_4_O_3_ | N-Acetyl-L-Arginine | Amino acids and derivatives | Amino acids and derivatives | 155-84-0 | 2 | 4.04E+05 | 4.61E+05 | 3.86E+05 | 7.94E+06 | 7.77E+06 | 7.03E+06 | 1.23E+00 | 1.42E-03 | 8.19E-03 | 1.82E+01 | 4.18E+00 | up |
| MWSmce184 | C_12_H_10_O_4_ | Ethyl 3-coumarincarboxylate | Lignans and Coumarins | Coumarins | 1846-76-0 | 3 | 2.08E+04 | 2.07E+04 | 2.52E+04 | 7.26E+04 | 7.46E+04 | 8.65E+04 | 1.22E+00 | 2.94E-03 | 1.22E-02 | 3.50E+00 | 1.81E+00 | up |
| Lmjp002764 | C_11_H_10_O_5_ | Umckalin (7-hydroxy-5,6-dimethoxycoumarin) | Lignans and Coumarins | Coumarins | 43053-62-9 | 2 | 2.28E+05 | 2.04E+05 | 2.07E+05 | 4.40E+05 | 3.37E+05 | 6.81E+05 | 1.09E+00 | 1.15E-01 | 1.78E-01 | 2.28E+00 | 1.19E+00 | up |
| MWSmce177 | C_12_H_14_O_4_ | Ethyl ferulate | Phenolic acids | Phenolic acids | 4046-02-0 | 1 | 1.00E+04 | 1.02E+04 | 1.13E+04 | 4.88E+04 | 5.02E+04 | 5.79E+04 | 1.23E+00 | 3.95E-03 | 1.50E-02 | 4.96E+00 | 2.31E+00 | up |
| mws0520 | C_11_H_13_NO_4_ | N-Acetyl-L-tyrosine | Amino acids and derivatives | Amino acids and derivatives | 537-55-3 | 3 | 5.41E+04 | 4.61E+04 | 3.89E+04 | 3.64E+05 | 3.86E+05 | 3.80E+05 | 1.23E+00 | 9.53E-06 | 6.24E-04 | 8.12E+00 | 3.02E+00 | up |
| Lmhp001430 | C_10_H_14_N_2_O_4_ | Cyclo(Pro-Glu) | Amino acids and derivatives | Amino acids and derivatives | - | 2 | 9.00E+00 | 9.00E+00 | 9.00E+00 | 6.86E+03 | 5.03E+03 | 7.95E+03 | 1.23E+00 | 1.62E-02 | 3.93E-02 | 7.35E+02 | 9.52E+00 | up |
| pmp000287 | C_12_H_8_O_5_ | 5-Methoxy-8-hydroxypsoralen | Lignans and Coumarins | Coumarins | 28437-68-5 | 2 | 1.85E+06 | 1.75E+06 | 1.01E+06 | 3.42E+06 | 2.86E+06 | 3.49E+06 | 1.10E+00 | 8.13E-03 | 2.39E-02 | 2.12E+00 | 1.08E+00 | up |
| pmb0962 | C_10_H_22_N_2_O_4_ | L-Lysine-Butanoic Acid | Amino acids and derivatives | Amino acids and derivatives | 80407-71-2 | 3 | 5.62E+04 | 5.68E+04 | 3.23E+04 | 1.01E+05 | 1.04E+05 | 9.28E+04 | 1.10E+00 | 1.36E-02 | 3.47E-02 | 2.05E+00 | 1.03E+00 | up |
| mws4176 | C_12_H_16_N_2_O_3_ | L-Alanyl-L-Phenylalanine | Amino acids and derivatives | Amino acids and derivatives | 3061-90-3 | 2 | 3.72E+04 | 4.14E+04 | 3.55E+04 | 6.86E+04 | 7.96E+04 | 9.22E+04 | 1.20E+00 | 2.01E-02 | 4.64E-02 | 2.11E+00 | 1.08E+00 | up |
| pme0264 | C_10_H_14_N_2_O_5_ | Thymidine | Nucleotides and derivatives | Nucleotides and derivatives | 50-89-5 | 1 | 6.82E+04 | 8.92E+04 | 6.85E+04 | 2.29E+05 | 2.39E+05 | 2.30E+05 | 1.22E+00 | 3.43E-04 | 3.75E-03 | 3.09E+00 | 1.63E+00 | up |
| ML10180524 | C_9_H_13_N_3_O_5_ | Cytarabine | Nucleotides and derivatives | Nucleotides and derivatives | 147-94-4 | 3 | 1.04E+06 | 1.12E+06 | 1.07E+06 | 2.01E+06 | 2.51E+06 | 2.55E+06 | 1.21E+00 | 1.62E-02 | 3.93E-02 | 2.19E+00 | 1.13E+00 | up |
| pme3732 | C_9_H_13_N_3_O_5_ | Cytidine | Nucleotides and derivatives | Nucleotides and derivatives | 65-46-3 | 3 | 5.65E+06 | 4.47E+06 | 4.99E+06 | 1.37E+07 | 1.38E+07 | 1.40E+07 | 1.22E+00 | 8.59E-04 | 6.35E-03 | 2.74E+00 | 1.45E+00 | up |
| mws0976 | C_9_H_12_N_2_O_6_ | β-Pseudouridine | Nucleotides and derivatives | Nucleotides and derivatives | 1445-07-4 | 2 | 7.64E+04 | 6.97E+04 | 7.58E+04 | 1.83E+05 | 1.79E+05 | 1.27E+05 | 1.18E+00 | 3.76E-02 | 7.30E-02 | 2.21E+00 | 1.14E+00 | up |
| pme3961 | C_10_H_13_N_5_O_3_ | 2'-Deoxyadenosine | Nucleotides and derivatives | Nucleotides and derivatives | 958-09-8 | 2 | 3.74E+05 | 3.76E+05 | 3.51E+05 | 1.07E+06 | 1.04E+06 | 1.03E+06 | 1.23E+00 | 3.35E-06 | 4.04E-04 | 2.85E+00 | 1.51E+00 | up |
| mws1715 | C_10_H_13_N_5_O_3_ | Cordycepin (3'-Deoxyadenosine) | Nucleotides and derivatives | Nucleotides and derivatives | 73-03-0 | 3 | 9.00E+00 | 9.00E+00 | 9.00E+00 | 1.89E+04 | 1.94E+04 | 1.79E+04 | 1.24E+00 | 5.48E-04 | 4.96E-03 | 2.08E+03 | 1.10E+01 | up |
| mws0040 | C_15_H_10_O_4_ | Chrysin | Flavonoids | Flavones | 480-40-0 | 3 | 9.50E+04 | 9.93E+04 | 9.96E+04 | 4.52E+04 | 3.23E+04 | 4.01E+04 | 1.21E+00 | 1.41E-03 | 8.19E-03 | 4.00E-01 | -1.32E+00 | down |
| mws0120 | C_8_H_20_NO_6_P | Choline Alfoscerate | Lipids | PC | 28319-77-9 | 3 | 4.79E+05 | 4.74E+05 | 5.16E+05 | 1.09E+06 | 1.15E+06 | 1.09E+06 | 1.23E+00 | 6.83E-05 | 1.18E-03 | 2.27E+00 | 1.18E+00 | up |
| pme1187 | C_10_H_14_N_2_O_6_ | 5-Methyluridine | Nucleotides and derivatives | Nucleotides and derivatives | 1463-10-1 | 3 | 1.50E+04 | 1.27E+04 | 1.08E+04 | 2.72E+04 | 3.10E+04 | 2.07E+04 | 1.14E+00 | 3.25E-02 | 6.59E-02 | 2.05E+00 | 1.03E+00 | up |
| Lmlp003161 | C_14_H_20_N_2_O_3_ | N-Feruloylputrescine | Alkaloids | Phenolamine | 501-13-3 | 3 | 1.80E+04 | 1.74E+04 | 3.00E+04 | 9.00E+00 | 9.00E+00 | 9.00E+00 | 1.23E+00 | 3.34E-02 | 6.70E-02 | 4.13E-04 | -1.12E+01 | down |
| pme1184 | C_10_H_13_N_5_O_4_ | 2'-Deoxyguanosine | Nucleotides and derivatives | Nucleotides and derivatives | 961-07-9 | 2 | 1.32E+05 | 1.45E+05 | 1.26E+05 | 4.08E+05 | 3.99E+05 | 4.08E+05 | 1.23E+00 | 3.76E-05 | 8.96E-04 | 3.01E+00 | 1.59E+00 | up |
| MWSHY0140 | C_16_H_12_O_4_ | 3-Hydroxy-3'-methoxyflavone | Flavonoids | Flavones | 76666-32-5 | 2 | 9.00E+00 | 9.00E+00 | 9.00E+00 | 6.50E+04 | 6.97E+04 | 7.06E+04 | 1.24E+00 | 6.33E-04 | 5.43E-03 | 7.61E+03 | 1.29E+01 | up |
| mws1060 | C_10_H_12_N_4_O_5_ | 9-(Arabinosyl)hypoxanthine | Nucleotides and derivatives | Nucleotides and derivatives | 7013-16-3 | 1 | 1.02E+05 | 1.03E+05 | 1.05E+05 | 2.71E+05 | 2.56E+05 | 2.67E+05 | 1.23E+00 | 4.39E-04 | 4.29E-03 | 2.56E+00 | 1.36E+00 | up |
| pmp000086 | C_13_H_16_O_6_ | 1-Feruloyl-sn-glycerol* | Phenolic acids | Phenolic acids | - | 3 | 8.16E+03 | 1.54E+04 | 7.22E+03 | 4.05E+04 | 2.95E+04 | 2.70E+04 | 1.13E+00 | 1.58E-02 | 3.86E-02 | 3.15E+00 | 1.65E+00 | up |
| pmp000087 | C_13_H_16_O_6_ | 2-Feruloyl-sn-glycerol* | Phenolic acids | Phenolic acids | - | 3 | 1.58E+04 | 1.02E+04 | 1.10E+04 | 4.22E+04 | 3.37E+04 | 2.93E+04 | 1.17E+00 | 1.44E-02 | 3.59E-02 | 2.85E+00 | 1.51E+00 | up |
| mws1355 | C_16_H_32_O_3_ | 16-Hydroxyhexadecanoic acid | Lipids | Free fatty acids | 506-13-8 | 3 | 9.00E+00 | 9.00E+00 | 9.00E+00 | 6.13E+03 | 6.08E+03 | 6.78E+03 | 1.24E+00 | 1.28E-03 | 7.75E-03 | 7.03E+02 | 9.46E+00 | up |
| Lmmn003323 | C_16_H_32_O_3_ | 2-Hydroxyhexadecanoic acid | Organic acids | Organic acids | 764-67-0 | 1 | 2.19E+06 | 2.41E+06 | 2.00E+06 | 9.73E+06 | 1.02E+07 | 9.76E+06 | 1.23E+00 | 4.25E-06 | 4.04E-04 | 4.49E+00 | 2.17E+00 | up |
| Zmsp001272 | C_16_H_19_NO_3_ | Norgalanthamine | Alkaloids | Alkaloids | - | 2 | 1.05E+04 | 1.36E+04 | 1.31E+04 | 2.49E+05 | 2.68E+05 | 2.89E+05 | 1.23E+00 | 1.94E-03 | 9.58E-03 | 2.17E+01 | 4.44E+00 | up |
| pmp000292 | C_15_H_16_O_5_ | Hamaudol | Others | Others | 735-46-6 | 2 | 1.32E+05 | 1.36E+05 | 1.37E+05 | 3.39E+05 | 3.28E+05 | 3.52E+05 | 1.23E+00 | 7.74E-04 | 6.04E-03 | 2.51E+00 | 1.33E+00 | up |
| pmp001133 | C_17_H_28_NO_2_+ | N-Methyldendrobine | Alkaloids | Sesquiterpene alkaloids | - | 2 | 3.63E+05 | 3.97E+05 | 3.71E+05 | 8.32E+05 | 8.34E+05 | 9.32E+05 | 1.23E+00 | 2.37E-03 | 1.07E-02 | 2.30E+00 | 1.20E+00 | up |
| pmb0889 | C_18_H_30_O_2_ | Punicic acid (9Z,11E,13Z-octadecatrienoic acid) | Lipids | Free fatty acids | 544-72-9 | 3 | 5.01E+06 | 5.60E+06 | 5.63E+06 | 1.28E+07 | 1.36E+07 | 1.31E+07 | 1.23E+00 | 2.23E-05 | 7.62E-04 | 2.43E+00 | 1.28E+00 | up |
| pme1178 | C_10_H_13_N_5_O_5_ | Guanosine | Nucleotides and derivatives | Nucleotides and derivatives | 118-00-3 | 1 | 2.43E+07 | 2.57E+07 | 2.30E+07 | 6.15E+07 | 6.25E+07 | 6.42E+07 | 1.23E+00 | 4.12E-06 | 4.04E-04 | 2.58E+00 | 1.37E+00 | up |
| Zmzp005934 | C_18_H_37_NO | Stearamide | Alkaloids | Alkaloids | 124-26-5 | 1 | 8.79E+04 | 9.66E+04 | 7.95E+04 | 3.50E+04 | 5.17E+04 | 4.01E+04 | 1.17E+00 | 2.82E-03 | 1.21E-02 | 4.80E-01 | -1.06E+00 | down |
| mws0051 | C_16_H_12_O_5_ | Acacetin | Flavonoids | Flavones | 480-44-4 | 3 | 4.11E+03 | 1.51E+03 | 1.82E+03 | 9.00E+00 | 9.00E+00 | 9.00E+00 | 1.23E+00 | 9.48E-02 | 1.52E-01 | 3.63E-03 | -8.11E+00 | down |
| mws0668 | C_10_H_12_N_4_O_6_ | Xanthosine | Nucleotides and derivatives | Nucleotides and derivatives | 146-80-5 | 1 | 1.36E+05 | 1.39E+05 | 1.50E+05 | 1.10E+06 | 1.22E+06 | 1.24E+06 | 1.23E+00 | 1.75E-03 | 9.18E-03 | 8.39E+00 | 3.07E+00 | up |
| Zmyn005252 | C_17_H_34_O_3_ | 3-Hydroxy-palmitic acid methyl ester | Lipids | Free fatty acids | 51883-36-4 | 1 | 4.11E+03 | 3.77E+03 | 4.58E+03 | 1.12E+04 | 1.14E+04 | 9.03E+03 | 1.21E+00 | 9.26E-03 | 2.63E-02 | 2.54E+00 | 1.35E+00 | up |
| Zmdp001928 | C_11_H_18_N_2_O_5_S | γ-L-Glutamyl-S-(trans-1-propenyl)-L-cysteine | Amino acids and derivatives | Amino acids and derivatives | - | 2 | 1.38E+04 | 2.66E+04 | 1.48E+04 | 4.03E+04 | 4.75E+04 | 5.79E+04 | 1.12E+00 | 1.11E-02 | 2.99E-02 | 2.64E+00 | 1.40E+00 | up |
| MWSmce689 | C_19_H_32_O_2_ | Methyl linolenate | Lipids | Free fatty acids | 301-00-8 | 2 | 2.53E+05 | 2.70E+05 | 2.67E+05 | 5.93E+05 | 6.69E+05 | 7.06E+05 | 1.23E+00 | 6.16E-03 | 1.98E-02 | 2.49E+00 | 1.32E+00 | up |
| Zmdn001564 | C_14_H_18_N_2_O_5_ | γ-Glutamylphenylalanine | Amino acids and derivatives | Amino acids and derivatives | 7432-24-8 | 1 | 1.85E+05 | 1.79E+05 | 2.07E+05 | 4.06E+05 | 4.10E+05 | 4.31E+05 | 1.23E+00 | 4.39E-05 | 9.64E-04 | 2.18E+00 | 1.13E+00 | up |
| pmb2786 | C_18_H_30_O_3_ | 9-Hydroxy-10,12,15-octadecatrienoic acid | Lipids | Free fatty acids | 89886-42-0 | 2 | 1.48E+05 | 1.56E+05 | 1.76E+05 | 4.01E+05 | 3.86E+05 | 4.13E+05 | 1.23E+00 | 3.36E-05 | 8.90E-04 | 2.50E+00 | 1.32E+00 | up |
| Lmmn006306 | C_18_H_30_O_3_ | Machilusolide D | Others | Others | - | 2 | 1.62E+06 | 1.77E+06 | 1.67E+06 | 3.56E+06 | 3.45E+06 | 3.72E+06 | 1.23E+00 | 1.42E-04 | 1.99E-03 | 2.12E+00 | 1.08E+00 | up |
| pmb2792 | C_18_H_30_O_3_ | 13-Hydroxy-6,9,11-octadecatrienoic acid | Lipids | Free fatty acids | 74784-20-6 | 3 | 2.54E+05 | 3.84E+05 | 1.69E+05 | 1.10E+05 | 1.03E+05 | 1.18E+05 | 1.07E+00 | 1.26E-01 | 1.89E-01 | 4.10E-01 | -1.28E+00 | down |
| pmb2787 | C_18_H_30_O_3_ | 9-Oxo-10E,12Z-octadecadienoic acid | Lipids | Free fatty acids | 54232-59-6 | 3 | 9.19E+04 | 9.88E+04 | 9.55E+04 | 2.54E+05 | 2.44E+05 | 2.59E+05 | 1.23E+00 | 1.15E-04 | 1.73E-03 | 2.65E+00 | 1.41E+00 | up |
| Zmyn004732 | C_18_H_30_O_3_ | 2R-hydroxy-9Z,12Z,15Z-octadecatrienoic acid | Lipids | Free fatty acids | - | 1 | 8.08E+05 | 8.88E+05 | 7.90E+05 | 1.78E+06 | 1.83E+06 | 1.86E+06 | 1.23E+00 | 2.07E-05 | 7.62E-04 | 2.20E+00 | 1.14E+00 | up |
| Lmbn005443 | C_18_H_30_O_3_ | 13-KODE; (9Z,11E)-13-Oxooctadeca-9,11-dienoic acid | Lipids | Free fatty acids | 54739-30-9 | 2 | 1.63E+05 | 1.72E+05 | 1.70E+05 | 4.40E+05 | 4.24E+05 | 4.20E+05 | 1.23E+00 | 6.35E-05 | 1.15E-03 | 2.54E+00 | 1.35E+00 | up |
| Lmqp000329 | C_8_H_14_N_3_O_7_P | 5-Aminoimidazole ribonucleotide | Nucleotides and derivatives | Nucleotides and derivatives | 25635-88-5 | 1 | 4.55E+05 | 4.01E+05 | 4.63E+05 | 9.64E+05 | 1.08E+06 | 1.11E+06 | 1.22E+00 | 1.46E-03 | 8.29E-03 | 2.39E+00 | 1.25E+00 | up |
| Hmhp011280 | C_18_H_33_NO_2_ | Tetrahydrobungeanool | Alkaloids | Alkaloids | - | 3 | 1.64E+03 | 2.02E+03 | 1.25E+03 | 2.90E+03 | 2.75E+03 | 4.19E+03 | 1.08E+00 | 5.07E-02 | 9.20E-02 | 2.00E+00 | 1.00E+00 | up |
| Hmqp005455 | C_18_H_32_O_3_ | 15(R)-Hydroxylinoleic Acid | Lipids | Free fatty acids | 177931-23-6 | 2 | 3.10E+05 | 3.03E+05 | 2.83E+05 | 7.33E+05 | 7.32E+05 | 7.50E+05 | 1.23E+00 | 3.89E-06 | 4.04E-04 | 2.47E+00 | 1.31E+00 | up |
| Hmqp005411 | C_18_H_32_O_3_ | 9-Oxo-12Z-Octadecenoic acid | Lipids | Free fatty acids | 112543-32-5 | 2 | 2.97E+05 | 2.86E+05 | 2.77E+05 | 7.68E+05 | 7.65E+05 | 7.87E+05 | 1.23E+00 | 1.09E-06 | 2.59E-04 | 2.70E+00 | 1.43E+00 | up |
| pmb2799 | C_18_H_32_O_3_ | 12,13-Epoxy-9-Octadecenoic Acid | Lipids | Free fatty acids | 6799-85-5 | 2 | 3.03E+05 | 3.00E+05 | 2.99E+05 | 7.65E+05 | 7.78E+05 | 8.36E+05 | 1.23E+00 | 1.89E-03 | 9.58E-03 | 2.64E+00 | 1.40E+00 | up |
| Lmbn005369 | C_18_H_32_O_3_ | 13(S)-HODE;13(S)-Hydroxyoctadeca-9Z,11E-dienoic acid* | Lipids | Free fatty acids | 10219-69-9 | 1 | 1.24E+06 | 1.30E+06 | 1.34E+06 | 3.44E+06 | 3.30E+06 | 3.25E+06 | 1.23E+00 | 6.13E-05 | 1.15E-03 | 2.58E+00 | 1.37E+00 | up |
| Rfmb091 | C_18_H_32_O_3_ | 9S-Hydroxy-10E,12Z-octadecadienoic acid* | Lipids | Free fatty acids | 15514-85-9 | 1 | 1.32E+06 | 1.42E+06 | 1.35E+06 | 3.38E+06 | 3.44E+06 | 3.42E+06 | 1.23E+00 | 5.76E-06 | 4.99E-04 | 2.51E+00 | 1.32E+00 | up |
| Lmbn005662 | C_18_H_32_O_3_ | 9(10)-EpOME;(9R,10S)-(12Z)-9,10-Epoxyoctadecenoic acid | Lipids | Free fatty acids | 16833-56-0 | 1 | 4.08E+05 | 4.35E+05 | 3.98E+05 | 8.72E+05 | 9.01E+05 | 9.19E+05 | 1.23E+00 | 1.59E-05 | 7.62E-04 | 2.17E+00 | 1.12E+00 | up |
| Zmgn005057 | C_18_H_34_O_3_ | 9,10-Epoxyoctadecanoic Acid | Lipids | Free fatty acids | 2443-39-2 | 2 | 4.05E+03 | 3.75E+03 | 5.13E+03 | 9.36E+03 | 9.82E+03 | 9.45E+03 | 1.20E+00 | 3.25E-03 | 1.32E-02 | 2.21E+00 | 1.15E+00 | up |
| ML10195036 | C_18_H_37_NO_2_ | 3-Dehydrosphinganine | Lipids | Sphingolipids | 16105-69-4 | 1 | 7.27E+03 | 1.22E+04 | 1.03E+04 | 4.13E+04 | 4.85E+04 | 4.18E+04 | 1.21E+00 | 6.45E-04 | 5.43E-03 | 4.42E+00 | 2.14E+00 | up |
| MWS4295 | C_18_H_36_O_3_ | DL-2-hydroxystearic acid | Lipids | Free fatty acids | 629-22-1 | 1 | 9.37E+03 | 7.77E+03 | 8.90E+03 | 2.57E+04 | 2.63E+04 | 2.81E+04 | 1.23E+00 | 8.79E-05 | 1.43E-03 | 3.08E+00 | 1.62E+00 | up |
| Zmyn005384 | C_18_H_36_O_3_ | 2R-Hydroxyoctadecanoic Acid | Lipids | Free fatty acids | 26633-48-7 | 2 | 7.72E+03 | 1.06E+04 | 7.89E+03 | 2.80E+04 | 2.58E+04 | 2.47E+04 | 1.21E+00 | 1.96E-04 | 2.45E-03 | 3.00E+00 | 1.58E+00 | up |
| pmb3079 | C_8_H_16_NO_9_P | N-Acetyl-D-glucosamine-1-phosphate | Others | Saccharides and Alcohols | 6866-69-9 | 3 | 8.73E+04 | 9.34E+04 | 9.27E+04 | 3.89E+04 | 3.23E+04 | 5.04E+04 | 1.18E+00 | 5.66E-03 | 1.89E-02 | 4.45E-01 | -1.17E+00 | down |
| Zmdp007400 | C_17_H_18_O_5_ | 4,5-dihydroxy-2,3,6-trimethoxy-9,10-dihydrophenanthrene | Others | Others | - | 2 | 1.78E+05 | 1.32E+05 | 1.50E+05 | 3.88E+05 | 4.00E+05 | 4.22E+05 | 1.21E+00 | 1.91E-04 | 2.42E-03 | 2.63E+00 | 1.40E+00 | up |
| pme1086 | C_10_H_17_N_3_O_6_S | Glutathione reduced form | Amino acids and derivatives | Amino acids and derivatives | 70-18-8 | 3 | 1.63E+04 | 2.30E+04 | 1.06E+04 | 9.00E+00 | 9.00E+00 | 9.00E+00 | 1.23E+00 | 4.38E-02 | 8.24E-02 | 5.41E-04 | -1.09E+01 | down |
| pmb2855 | C_11_H_20_N_2_O_8_ | L-Glutamine-O-glycoside | Amino acids and derivatives | Amino acids and derivatives | - | 3 | 3.79E+05 | 4.83E+05 | 4.12E+05 | 4.43E+06 | 4.52E+06 | 5.16E+06 | 1.23E+00 | 2.50E-03 | 1.12E-02 | 1.11E+01 | 3.47E+00 | up |
| pmb2857 | C_11_H_19_NO_9_ | L-Glutamic acid-O-glycoside | Amino acids and derivatives | Amino acids and derivatives | - | 3 | 4.97E+04 | 2.80E+04 | 2.12E+04 | 7.95E+05 | 4.38E+05 | 5.44E+05 | 1.21E+00 | 3.32E-02 | 6.69E-02 | 1.80E+01 | 4.17E+00 | up |
| Zmdp001857 | C_14_H_18_N_2_O_6_ | γ-Glutamyltyrosine | Amino acids and derivatives | Amino acids and derivatives | 7432-23-7 | 2 | 4.05E+05 | 4.51E+05 | 4.41E+05 | 8.41E+05 | 8.57E+05 | 9.14E+05 | 1.23E+00 | 2.16E-04 | 2.60E-03 | 2.02E+00 | 1.01E+00 | up |
| pme3967 | C_12_H_17_N_5_O_5_ | 2-(Dimethylamino)guanosine | Nucleotides and derivatives | Nucleotides and derivatives | 2140-67-2 | 1 | 5.68E+05 | 5.22E+05 | 5.55E+05 | 1.15E+06 | 1.36E+06 | 1.23E+06 | 1.22E+00 | 6.00E-03 | 1.97E-02 | 2.27E+00 | 1.18E+00 | up |
| Lmcp002302 | C_12_H_17_N_5_O_5_ | N6-(2-Hydroxyethyl)adenosine | Nucleotides and derivatives | Nucleotides and derivatives | 4338-48-1 | 1 | 5.44E+05 | 4.78E+05 | 4.80E+05 | 1.18E+06 | 1.28E+06 | 1.13E+06 | 1.23E+00 | 1.12E-03 | 7.18E-03 | 2.39E+00 | 1.26E+00 | up |
| Lmhn001477 | C_13_H_12_O_9_ | 2-Caffeoyl-L-tartaric acid (Caftaric acid) | Phenolic acids | Phenolic acids | 67879-58-7 | 3 | 3.19E+03 | 4.10E+03 | 2.88E+03 | 7.09E+03 | 7.94E+03 | 9.37E+03 | 1.18E+00 | 7.42E-03 | 2.26E-02 | 2.40E+00 | 1.26E+00 | up |
| Lmbn005287 | C_18_H_32_O_4_ | 7S,8S-DiHODE; (9Z,12Z)-(7S,8S)-Dihydroxyoctadeca-9,12-dienoic acid | Lipids | Free fatty acids | 143288-65-7 | 2 | 1.64E+04 | 1.72E+04 | 1.42E+04 | 9.02E+04 | 8.04E+04 | 1.04E+05 | 1.23E+00 | 7.12E-03 | 2.20E-02 | 5.74E+00 | 2.52E+00 | up |
| pmb2804 | C_18_H_32_O_4_ | 13S-Hydroperoxy-9Z,11E-octadecadienoic acid | Lipids | Free fatty acids | 33964-75-9 | 3 | 1.08E+06 | 1.07E+06 | 1.01E+06 | 2.32E+06 | 2.34E+06 | 2.19E+06 | 1.23E+00 | 3.08E-04 | 3.41E-03 | 2.17E+00 | 1.11E+00 | up |
| Zmjn004133 | C_18_H_32_O_4_ | 9S-Hydroperoxy-10E,12Z-octadecadienoic acid | Lipids | Free fatty acids | 5502-91-0 | 3 | 1.66E+05 | 1.55E+05 | 1.64E+05 | 3.04E+05 | 3.20E+05 | 3.58E+05 | 1.22E+00 | 7.24E-03 | 2.22E-02 | 2.02E+00 | 1.02E+00 | up |
| pmn001689 | C_18_H_32_O_4_ | 9-Hydroxy-12-oxo-15(Z)-octadecenoic acid | Lipids | Free fatty acids | - | 1 | 4.60E+04 | 5.48E+04 | 4.66E+04 | 2.09E+05 | 2.06E+05 | 2.00E+05 | 1.23E+00 | 2.44E-06 | 4.04E-04 | 4.17E+00 | 2.06E+00 | up |
| Lmbn005487 | C_18_H_34_O_4_ | 12,13-DHOME; (9Z)-12,13-Dihydroxyoctadec-9-enoic acid | Lipids | Free fatty acids | 263399-35-5 | 3 | 8.82E+03 | 9.39E+03 | 9.28E+03 | 4.10E+04 | 4.30E+04 | 4.29E+04 | 1.23E+00 | 1.48E-04 | 1.99E-03 | 4.61E+00 | 2.21E+00 | up |
| Lmbn007891 | C_18_H_34_O_4_ | Hydroxy ricinoleic acid | Lipids | Free fatty acids | - | 1 | 1.10E+05 | 1.17E+05 | 8.64E+04 | 2.13E+05 | 2.12E+05 | 2.08E+05 | 1.20E+00 | 6.24E-03 | 1.99E-02 | 2.02E+00 | 1.02E+00 | up |
| pmp000970 | C_20_H_30_O_3_ | Hispanolone | Terpenoids | Ditepenoids | 18676-07-8 | 2 | 2.25E+04 | 2.87E+04 | 2.76E+04 | 5.12E+04 | 6.78E+04 | 5.14E+04 | 1.18E+00 | 2.14E-02 | 4.84E-02 | 2.16E+00 | 1.11E+00 | up |
| pmp001267 | C_21_H_37_NO | 2-(Dodecylamino)-3-phenyl-1-propanol | Others | Others | - | 2 | 9.00E+00 | 9.00E+00 | 9.00E+00 | 2.46E+04 | 2.65E+04 | 2.24E+04 | 1.24E+00 | 2.26E-03 | 1.03E-02 | 2.72E+03 | 1.14E+01 | up |
| pmb3075 | C_16_H_16_O_7_ | 3-O-p-Coumaroylshikimic acid | Phenolic acids | Phenolic acids | - | 3 | 9.00E+00 | 9.00E+00 | 9.00E+00 | 5.07E+04 | 5.15E+04 | 4.75E+04 | 1.24E+00 | 6.02E-04 | 5.26E-03 | 5.54E+03 | 1.24E+01 | up |
| pmb0751 | C_16_H_16_O_7_ | Trans-5-O-(p-Coumaroyl)shikimate | Phenolic acids | Phenolic acids | - | 3 | 2.98E+04 | 4.46E+04 | 5.30E+04 | 6.08E+05 | 6.39E+05 | 6.54E+05 | 1.23E+00 | 4.86E-05 | 9.88E-04 | 1.49E+01 | 3.90E+00 | up |
| mws0582 | C_11_H_19_N_3_O_6_S | S-(Methyl)glutathione | Amino acids and derivatives | Amino acids and derivatives | 2922-56-7 | 2 | 4.00E+05 | 2.40E+05 | 2.73E+05 | 5.78E+05 | 9.21E+05 | 6.20E+05 | 1.10E+00 | 4.77E-02 | 8.77E-02 | 2.32E+00 | 1.21E+00 | up |
| pme3174 | C_9_H_14_N_3_O_8_P | Cytidine 5'-monophosphate(Cytidylic acid) | Nucleotides and derivatives | Nucleotides and derivatives | 63-37-6 | 3 | 3.30E+04 | 2.32E+04 | 2.75E+04 | 1.35E+05 | 1.43E+05 | 1.24E+05 | 1.22E+00 | 3.70E-04 | 3.96E-03 | 4.81E+00 | 2.26E+00 | up |
| pme3188 | C_9_H_13_N_2_O_9_P | Uridine 5'-monophosphate | Nucleotides and derivatives | Nucleotides and derivatives | 58-97-9 | 2 | 4.86E+05 | 5.42E+05 | 5.10E+05 | 3.16E+06 | 3.12E+06 | 3.44E+06 | 1.23E+00 | 1.08E-03 | 7.18E-03 | 6.32E+00 | 2.66E+00 | up |
| Smcp000882 | C_15_H_21_NO_7_ | N-benzoyl-2-aminoethyl-β-D-glucopyranoside | Others | Others | - | 1 | 2.61E+05 | 2.99E+05 | 2.90E+05 | 2.31E+06 | 2.36E+06 | 2.33E+06 | 1.23E+00 | 2.49E-07 | 1.18E-04 | 8.23E+00 | 3.04E+00 | up |
| Lmtn002233 | C_15_H_20_O_8_ | Androsin | Phenolic acids | Phenolic acids | 531-28-2 | 2 | 9.00E+00 | 9.00E+00 | 9.00E+00 | 6.89E+04 | 6.50E+04 | 5.41E+04 | 1.24E+00 | 5.02E-03 | 1.74E-02 | 6.96E+03 | 1.28E+01 | up |
| Lmtn002565 | C_14_H_18_O_9_ | 1-O-Vanilloyl-D-Glucose | Phenolic acids | Phenolic acids | - | 3 | 1.82E+04 | 2.25E+04 | 2.07E+04 | 9.00E+00 | 9.00E+00 | 9.00E+00 | 1.24E+00 | 3.71E-03 | 1.45E-02 | 4.40E-04 | -1.12E+01 | down |
| mws0932 | C_20_H_32_O_4_ | 15-Hydroperoxyicosatetraenoic acid | Lipids | Free fatty acids | 70981-96-3 | 3 | 9.00E+00 | 9.00E+00 | 9.00E+00 | 4.65E+03 | 5.81E+03 | 4.46E+03 | 1.24E+00 | 7.14E-03 | 2.20E-02 | 5.53E+02 | 9.11E+00 | up |
| pma6460 | C_16_H_18_O_8_ | 4-O-p-Coumaroylquinic acid | Phenolic acids | Phenolic acids | 32451-86-8 | 3 | 1.18E+05 | 1.29E+05 | 1.39E+05 | 2.91E+05 | 3.62E+05 | 3.22E+05 | 1.22E+00 | 7.06E-03 | 2.19E-02 | 2.52E+00 | 1.33E+00 | up |
| pmb3074 | C_16_H_18_O_8_ | 5-O-p-Coumaroylquinic acid | Phenolic acids | Phenolic acids | 1899-30-5 | 2 | 8.86E+06 | 8.32E+06 | 8.97E+06 | 3.65E+07 | 3.75E+07 | 3.20E+07 | 1.23E+00 | 3.63E-03 | 1.43E-02 | 4.06E+00 | 2.02E+00 | up |
| pmn001421 | C_16_H_18_O_8_ | 3-O-p-Coumaroylquinic acid | Phenolic acids | Phenolic acids | 87099-71-6 | 1 | 2.84E+06 | 3.54E+06 | 2.84E+06 | 1.34E+07 | 1.36E+07 | 1.32E+07 | 1.23E+00 | 2.25E-05 | 7.62E-04 | 4.35E+00 | 2.12E+00 | up |
| pme3311 | C_6_H_14_O_12_P_2_ | D-Fructose-1,6-biphosphate | Others | Saccharides and Alcohols | 488-69-7 | 3 | 7.74E+04 | 7.41E+04 | 7.60E+04 | 3.18E+04 | 3.03E+04 | 2.80E+04 | 1.23E+00 | 7.09E-06 | 5.63E-04 | 3.96E-01 | -1.34E+00 | down |
| Xmgp006913 | C_20_H_20_O_5_ | 2,4,2',4'-tetrahydroxy-3'-prenylchalcone | Flavonoids | Chalcones | - | 3 | 1.02E+05 | 1.24E+05 | 9.88E+04 | 2.11E+05 | 1.96E+05 | 2.60E+05 | 1.18E+00 | 1.64E-02 | 3.96E-02 | 2.06E+00 | 1.04E+00 | up |
| Jmwn002620 | C_15_H_18_O_9_ | Vanillic Acid-4-O-Glucuronide | Phenolic acids | Phenolic acids | - | 2 | 4.00E+04 | 3.76E+04 | 3.62E+04 | 8.47E+04 | 9.49E+04 | 9.14E+04 | 1.23E+00 | 1.15E-03 | 7.24E-03 | 2.38E+00 | 1.25E+00 | up |
| mws0609 | C_10_H_12_N_5_O_7_P | Guanosine 3',5'-cyclic monophosphate | Nucleotides and derivatives | Nucleotides and derivatives | 7665-99-8 | 2 | 2.09E+05 | 2.39E+05 | 2.25E+05 | 3.75E+06 | 3.65E+06 | 4.09E+06 | 1.23E+00 | 1.27E-03 | 7.74E-03 | 1.71E+01 | 4.09E+00 | up |
| pmp000303 | C_19_H_22_O_6_ | 30-O-I-Butyrylhamaudol | Others | Others | - | 3 | 9.00E+00 | 9.00E+00 | 9.00E+00 | 2.19E+03 | 2.76E+03 | 2.90E+03 | 1.23E+00 | 6.83E-03 | 2.13E-02 | 2.91E+02 | 8.18E+00 | up |
| pmb1562 | C_21_H_34_O_4_ | 1-Stearidonoyl-Glycerol | Lipids | Glycerol ester | - | 3 | 7.13E+03 | 7.30E+03 | 6.22E+03 | 3.11E+04 | 3.13E+04 | 3.50E+04 | 1.23E+00 | 1.36E-03 | 8.05E-03 | 4.72E+00 | 2.24E+00 | up |
| pmb0770 | C_20_H_20_N_2_O_4_ | N-Feruloylserotonin | Alkaloids | Plumerane | 68573-23-9 | 3 | 1.12E+04 | 8.54E+03 | 1.04E+04 | 9.00E+00 | 9.00E+00 | 9.00E+00 | 1.24E+00 | 6.05E-03 | 1.97E-02 | 8.96E-04 | -1.01E+01 | down |
| pmb0296 | C_21_H_40_O_4_ | 1-Oleoyl-Sn-Glycerol | Lipids | Glycerol ester | 129784-87-8 | 1 | 1.96E+06 | 2.06E+06 | 2.03E+06 | 8.91E+05 | 9.51E+05 | 8.86E+05 | 1.23E+00 | 2.05E-05 | 7.62E-04 | 4.52E-01 | -1.15E+00 | down |
| Lmdn004267 | C_20_H_22_O_6_ | Epipinoresinol* | Lignans and Coumarins | Lignans | 24404-50-0 | 1 | 5.32E+04 | 4.97E+04 | 4.48E+04 | 1.97E+05 | 1.84E+05 | 2.65E+05 | 1.22E+00 | 2.17E-02 | 4.87E-02 | 4.37E+00 | 2.13E+00 | up |
| mws0097 | C_20_H_22_O_6_ | Pinoresinol* | Lignans and Coumarins | Lignans | 487-36-5 | 1 | 6.05E+04 | 5.01E+04 | 4.90E+04 | 2.33E+05 | 2.17E+05 | 3.08E+05 | 1.22E+00 | 1.79E-02 | 4.23E-02 | 4.75E+00 | 2.25E+00 | up |
| Hmgn002833 | C_20_H_20_O_7_ | 4-Ketopinoresinol | Others | Others | - | 1 | 2.45E+04 | 3.49E+04 | 3.23E+04 | 5.66E+04 | 5.65E+04 | 7.43E+04 | 1.15E+00 | 1.69E-02 | 4.06E-02 | 2.04E+00 | 1.03E+00 | up |
| mws0232 | C_17_H_20_N_4_O_6_ | Riboflavin (Vitamin B2) | Others | Vitamin | 83-88-5 | 1 | 9.09E+04 | 8.94E+04 | 8.40E+04 | 2.58E+05 | 2.47E+05 | 2.47E+05 | 1.23E+00 | 1.45E-05 | 7.62E-04 | 2.85E+00 | 1.51E+00 | up |
| pmp001132 | C_22_H_34_NO_4_+ | N-Isopentenyl-6-hydroxydendroxinium | Alkaloids | Alkaloids | - | 2 | 9.00E+00 | 9.00E+00 | 9.00E+00 | 1.04E+04 | 1.09E+04 | 5.52E+03 | 1.23E+00 | 3.53E-02 | 6.91E-02 | 9.96E+02 | 9.96E+00 | up |
| Hmap010126 | C_24_H_28_O_4_ | Badrakemone | Lignans and Coumarins | Coumarins | - | 3 | 5.65E+03 | 4.12E+03 | 4.65E+03 | 1.84E+04 | 1.95E+04 | 1.94E+04 | 1.22E+00 | 2.45E-05 | 7.77E-04 | 3.98E+00 | 1.99E+00 | up |
| pmp000258 | C_24_H_28_O_4_ | Z-Ligustilide dimer E-232 | Others | Others | - | 3 | 2.40E+03 | 2.74E+03 | 1.20E+03 | 6.25E+03 | 4.91E+03 | 5.43E+03 | 1.10E+00 | 5.45E-03 | 1.85E-02 | 2.61E+00 | 1.39E+00 | up |
| pmp000255 | C_24_H_28_O_4_ | Z,Z9-6.89,7.39-Diligustilide | Others | Others | - | 3 | 9.00E+00 | 9.00E+00 | 9.00E+00 | 7.50E+03 | 7.47E+03 | 7.37E+03 | 1.24E+00 | 2.74E-05 | 8.03E-04 | 8.27E+02 | 9.69E+00 | up |
| pme3337 | C_14_H_17_N_5_O_8_ | Succinyladenosine | Nucleotides and derivatives | Nucleotides and derivatives | 4542-23-8 | 1 | 7.06E+06 | 7.60E+06 | 6.36E+06 | 2.01E+07 | 1.91E+07 | 2.00E+07 | 1.23E+00 | 1.34E-05 | 7.51E-04 | 2.82E+00 | 1.50E+00 | up |
| Lmjp003090 | C_17_H_20_O_10_ | Isofraxidin-7-O-glucoside | Lignans and Coumarins | Coumarins | 483-91-0 | 2 | 3.90E+04 | 3.63E+04 | 4.81E+04 | 2.44E+04 | 1.21E+04 | 1.48E+04 | 1.11E+00 | 9.69E-03 | 2.70E-02 | 4.16E-01 | -1.26E+00 | down |
| pme1286 | C_14_H_20_N_6_O_5_S | S-(5'-Adenosy)-L-homocysteine | Amino acids and derivatives | Amino acids and derivatives | 979-92-0 | 3 | 1.15E+05 | 1.18E+05 | 1.34E+05 | 2.99E+05 | 3.06E+05 | 2.90E+05 | 1.23E+00 | 2.91E-05 | 8.03E-04 | 2.44E+00 | 1.28E+00 | up |
| pmb2165 | C_18_H_38_NO_7_P | LysoPC 10:0 | Lipids | LPC | 22248-63-1 | 3 | 9.00E+00 | 9.00E+00 | 9.00E+00 | 2.49E+03 | 4.94E+03 | 5.07E+03 | 1.23E+00 | 3.85E-02 | 7.42E-02 | 4.63E+02 | 8.85E+00 | up |
| mws2523 | C_12_H_23_O_14_P | Trehalose 6-phosphate | Others | Saccharides and Alcohols | 4484-88-2 | 2 | 9.64E+04 | 7.24E+04 | 9.82E+04 | 2.15E+05 | 2.24E+05 | 2.12E+05 | 1.21E+00 | 1.29E-03 | 7.77E-03 | 2.44E+00 | 1.29E+00 | up |
| Hmcp005535 | C_30_H_48_O | α-Amyrenone | Terpenoids | Triterpene | 638-96-0 | 3 | 1.18E+04 | 1.13E+04 | 8.95E+03 | 9.00E+00 | 9.00E+00 | 9.00E+00 | 1.24E+00 | 6.72E-03 | 2.11E-02 | 8.43E-04 | -1.02E+01 | down |
| pmb0864 | C_19_H_40_NO_7_P | LysoPE 14:0 | Lipids | LPE | - | 1 | 2.21E+04 | 2.64E+04 | 3.09E+04 | 6.12E+04 | 5.66E+04 | 7.12E+04 | 1.19E+00 | 4.12E-03 | 1.54E-02 | 2.38E+00 | 1.25E+00 | up |
| Lmhp008337 | C_19_H_40_NO_7_P | LysoPE 14:0(2n isomer) | Lipids | LPE | - | 1 | 6.28E+03 | 4.53E+03 | 2.85E+03 | 7.53E+04 | 7.71E+04 | 9.04E+04 | 1.22E+00 | 2.80E-03 | 1.21E-02 | 1.78E+01 | 4.15E+00 | up |
| pme2117 | C_10_H_15_N_5_O_10_P_2_ | Adenosine 5'-diphosphate | Nucleotides and derivatives | Nucleotides and derivatives | 58-64-0 | 3 | 1.71E+05 | 1.49E+05 | 1.84E+05 | 6.45E+04 | 7.00E+04 | 5.91E+04 | 1.22E+00 | 5.75E-03 | 1.91E-02 | 3.85E-01 | -1.38E+00 | down |
| Lmhp008885 | C_20_H_42_NO_7_P | LysoPE 15:0(2n isomer) | Lipids | LPE | - | 1 | 1.82E+04 | 1.27E+04 | 1.81E+04 | 7.43E+04 | 7.35E+04 | 7.15E+04 | 1.22E+00 | 1.56E-04 | 2.02E-03 | 4.47E+00 | 2.16E+00 | up |
| pmb0862 | C_20_H_42_NO_7_P | LysoPC 12:0 | Lipids | LPC | 20559-18-6 | 3 | 8.03E+03 | 8.28E+03 | 6.72E+03 | 4.23E+04 | 3.59E+04 | 3.94E+04 | 1.23E+00 | 2.02E-03 | 9.90E-03 | 5.10E+00 | 2.35E+00 | up |
| Lmsp004915 | C_21_H_20_O_11_ | Aureusidin-4-O-glucoside | Flavonoids | Aurones | - | 2 | 1.07E+05 | 6.41E+04 | 7.37E+04 | 2.15E+05 | 2.99E+05 | 2.51E+05 | 1.18E+00 | 7.80E-03 | 2.33E-02 | 3.13E+00 | 1.65E+00 | up |
| MWS20147 | C_21_H_20_O_11_ | Luteolin-3'-O-glucoside | Flavonoids | Flavones | 5154-41-6 | 2 | 2.74E+05 | 2.23E+05 | 2.71E+05 | 6.93E+05 | 7.17E+05 | 8.15E+05 | 1.22E+00 | 1.93E-03 | 9.58E-03 | 2.89E+00 | 1.53E+00 | up |
| MWSHY0136 | C_21_H_20_O_11_ | Kaempferol-3-O-glucoside (Astragalin) | Flavonoids | Flavonols | 480-10-4 | 2 | 6.35E+04 | 6.33E+04 | 9.05E+04 | 2.09E+05 | 1.31E+05 | 2.80E+05 | 1.11E+00 | 8.27E-02 | 1.37E-01 | 2.85E+00 | 1.51E+00 | up |
| Lmlp005236 | C_21_H_22_O_11_ | Dihydrokaempferol-3-O-glucoside | Flavonoids | Flavanonols | 1049-08-8 | 2 | 6.19E+04 | 5.79E+04 | 5.37E+04 | 1.42E+04 | 2.04E+04 | 3.03E+04 | 1.13E+00 | 6.40E-03 | 2.02E-02 | 3.74E-01 | -1.42E+00 | down |
| Zmhn001257 | C_21_H_24_O_11_ | Catechin-5-O-glucoside | Flavonoids | Flavanols | - | 2 | 3.43E+04 | 3.30E+04 | 3.33E+04 | 1.37E+05 | 1.50E+05 | 1.67E+05 | 1.23E+00 | 5.48E-03 | 1.85E-02 | 4.51E+00 | 2.17E+00 | up |
| pmd0160 | C_21_H_44_NO_7_P | LysoPE 16:0(2n isomer) | Lipids | LPE | - | 1 | 1.37E+06 | 1.56E+06 | 1.58E+06 | 3.05E+06 | 3.18E+06 | 3.16E+06 | 1.23E+00 | 1.47E-04 | 1.99E-03 | 2.08E+00 | 1.06E+00 | up |
| pmn001700 | C_30_H_48_O_3_ | 24,30-Dihydroxy-12(13)-enolupinol | Terpenoids | Triterpene | - | 1 | 2.74E+04 | 3.02E+04 | 2.51E+04 | 8.85E+03 | 1.25E+04 | 1.44E+04 | 1.16E+00 | 2.16E-03 | 1.02E-02 | 4.33E-01 | -1.21E+00 | down |
| mws4053 | C_30_H_48_O_3_ | Ursolic acid | Terpenoids | Triterpene | 77-52-1 | 3 | 1.85E+03 | 3.74E+02 | 3.55E+02 | 4.15E+03 | 2.55E+03 | 5.13E+03 | 1.04E+00 | 3.34E-02 | 6.70E-02 | 4.59E+00 | 2.20E+00 | up |
| pmb0464 | C_16_H_27_NO_14_ | L-Aspartic acid-O-diglucoside | Amino acids and derivatives | Amino acids and derivatives | - | 3 | 1.91E+06 | 1.70E+06 | 1.34E+06 | 3.48E+06 | 3.07E+06 | 3.92E+06 | 1.17E+00 | 5.20E-03 | 1.78E-02 | 2.11E+00 | 1.08E+00 | up |
| Lmdp003171 | C_22_H_22_O_11_ | Azalein (Azaleatin-3-O-rhamnoside) | Flavonoids | Flavonols | 29028-02-2 | 2 | 6.16E+03 | 3.05E+03 | 8.44E+03 | 1.96E+04 | 1.81E+04 | 2.38E+04 | 1.12E+00 | 3.30E-03 | 1.33E-02 | 3.48E+00 | 1.80E+00 | up |
| Lmhp009769 | C_22_H_44_NO_7_P | LysoPE 17:1 | Lipids | LPE | - | 3 | 3.77E+04 | 2.69E+04 | 3.33E+04 | 4.62E+04 | 7.96E+04 | 1.01E+05 | 1.04E+00 | 1.09E-01 | 1.70E-01 | 2.31E+00 | 1.21E+00 | up |
| Lmhp009464 | C_22_H_44_NO_7_P | LysoPE 17:1(2n isomer) | Lipids | LPE | - | 3 | 3.63E+04 | 3.90E+04 | 2.47E+04 | 4.32E+05 | 4.35E+05 | 4.62E+05 | 1.23E+00 | 5.69E-05 | 1.08E-03 | 1.33E+01 | 3.73E+00 | up |
| Cmbp003767 | C_20_H_27_N_3_O_8_S | (E)-N5-(1-((Carboxymethyl)amino)-3-((3-(4-hydroxy-3-methoxyphenyl)allyl)thio)-1-oxopropan-2-yl)glutamine | Alkaloids | Alkaloids | - | 2 | 1.95E+05 | 2.11E+05 | 1.82E+05 | 9.69E+05 | 7.62E+05 | 1.00E+06 | 1.23E+00 | 9.99E-03 | 2.77E-02 | 4.64E+00 | 2.22E+00 | up |
| Lmhp009129 | C_23_H_48_NO_7_P | LysoPC 15:0(2n isomer) | Lipids | LPC | - | 2 | 8.79E+04 | 1.24E+05 | 1.02E+05 | 2.63E+05 | 2.76E+05 | 2.52E+05 | 1.21E+00 | 5.57E-04 | 4.96E-03 | 2.51E+00 | 1.33E+00 | up |
| pmd0132 | C_24_H_50_NO_7_P | LysoPC 16:0(2n isomer) | Lipids | LPC | - | 1 | 2.20E+06 | 2.27E+06 | 2.44E+06 | 6.09E+06 | 6.72E+06 | 6.96E+06 | 1.23E+00 | 2.12E-03 | 1.01E-02 | 2.86E+00 | 1.52E+00 | up |
| pmb0565 | C_23_H_24_O_13_ | Syringetin-3-O-glucoside | Flavonoids | Flavonols | 40039-49-4 | 3 | 1.44E+05 | 1.24E+05 | 7.45E+04 | 5.33E+04 | 5.32E+04 | 4.87E+04 | 1.09E+00 | 9.30E-02 | 1.49E-01 | 4.54E-01 | -1.14E+00 | down |
| pmb2406 | C_25_H_52_NO_7_P | LysoPC 17:0 | Lipids | LPC | 50930-23-9 | 2 | 2.86E+04 | 3.56E+04 | 3.63E+04 | 1.11E+05 | 1.04E+05 | 1.05E+05 | 1.22E+00 | 2.87E-05 | 8.03E-04 | 3.18E+00 | 1.67E+00 | up |
| Lmhp010515 | C_25_H_52_NO_7_P | LysoPC 17:0(2n isomer) | Lipids | LPC | - | 2 | 4.48E+04 | 5.66E+04 | 5.05E+04 | 1.38E+05 | 1.41E+05 | 1.62E+05 | 1.22E+00 | 1.86E-03 | 9.58E-03 | 2.90E+00 | 1.54E+00 | up |
| Lmhp009526 | C_27_H_46_O_9_ | 2-α-Linolenoyl-glycerol-1-O-glucoside* | Lipids | Glycerol ester | - | 1 | 8.33E+03 | 5.80E+03 | 9.50E+03 | 9.00E+00 | 9.00E+00 | 9.00E+00 | 1.23E+00 | 1.87E-02 | 4.38E-02 | 1.14E-03 | -9.77E+00 | down |
| Lmhp009773 | C_27_H_46_O_9_ | 1-α-Linolenoyl-glycerol-3-O-glucoside* | Lipids | Glycerol ester | - | 1 | 7.38E+03 | 7.18E+03 | 6.89E+03 | 9.00E+00 | 9.00E+00 | 9.00E+00 | 1.24E+00 | 4.02E-04 | 4.16E-03 | 1.26E-03 | -9.63E+00 | down |
| Lmhp010334 | C_27_H_48_O_9_ | 2-Linoleoylglycerol-1-O-glucoside* | Lipids | Glycerol ester | - | 1 | 2.49E+04 | 2.32E+04 | 2.06E+04 | 5.20E+03 | 5.07E+03 | 5.60E+03 | 1.23E+00 | 4.36E-03 | 1.60E-02 | 2.31E-01 | -2.11E+00 | down |
| Lmhp010573 | C_27_H_48_O_9_ | 1-Linoleoylglycerol-3-O-glucoside* | Lipids | Glycerol ester | - | 1 | 2.48E+04 | 2.53E+04 | 2.33E+04 | 4.03E+03 | 5.91E+03 | 4.79E+03 | 1.22E+00 | 1.99E-05 | 7.62E-04 | 2.01E-01 | -2.32E+00 | down |
| pmn001497 | C_33_H_44_N_2_O_3_ | Dendrocrepine | Alkaloids | Alkaloids | 51020-39-4 | 2 | 7.04E+05 | 7.15E+05 | 7.55E+05 | 1.69E+05 | 1.71E+05 | 1.67E+05 | 1.24E+00 | 7.23E-04 | 5.78E-03 | 2.34E-01 | -2.10E+00 | down |
| pma0791 | C_24_H_24_O_13_ | Naringenin-7-O-(6''-malonyl)glucoside | Flavonoids | Flavanones | - | 3 | 7.83E+03 | 6.34E+03 | 5.68E+03 | 1.67E+04 | 2.00E+04 | 1.22E+04 | 1.15E+00 | 4.16E-02 | 7.90E-02 | 2.46E+00 | 1.30E+00 | up |
| pmd0136 | C_26_H_54_NO_7_P | LysoPC 18:0(2n isomer) | Lipids | LPC | - | 1 | 4.30E+04 | 5.74E+04 | 5.82E+04 | 1.51E+05 | 1.30E+05 | 1.68E+05 | 1.20E+00 | 5.60E-03 | 1.88E-02 | 2.84E+00 | 1.50E+00 | up |
| pmc0960 | C_28_H_50_NO_7_P | LysoPC 20:4 | Lipids | LPC | - | 3 | 8.50E+03 | 5.64E+03 | 5.44E+03 | 9.00E+00 | 9.00E+00 | 9.00E+00 | 1.23E+00 | 2.23E-02 | 4.94E-02 | 1.38E-03 | -9.50E+00 | down |
| pmb2922 | C_15_H_24_N_2_O_17_P_2_ | Uridine 5'-diphospho-D-glucose | Nucleotides and derivatives | Nucleotides and derivatives | 133-89-1 | 1 | 9.88E+05 | 9.75E+05 | 9.66E+05 | 2.11E+05 | 2.18E+05 | 2.14E+05 | 1.24E+00 | 1.26E-05 | 7.51E-04 | 2.19E-01 | -2.19E+00 | down |
| pmp001080 | C_28_H_32_O_15_ | Diosmetin-7-O-Neohesperidoside (Neodiosmin) | Flavonoids | Flavanones | 38665-01-9 | 1 | 1.37E+05 | 1.47E+05 | 2.47E+05 | 7.19E+04 | 8.88E+04 | 9.65E+04 | 1.06E+00 | 1.18E-01 | 1.81E-01 | 4.85E-01 | -1.05E+00 | down |
| MWS0132 | C_20_H_32_N_6_O_12_S_2_ | Oxiglutatione | Amino acids and derivatives | Amino acids and derivatives | 27025-41-8 | 1 | 3.11E+05 | 4.05E+05 | 3.71E+05 | 1.36E+05 | 1.18E+05 | 9.25E+04 | 1.20E+00 | 4.90E-03 | 1.72E-02 | 3.19E-01 | -1.65E+00 | down |
| Smcn001947 | C_29_H_36_O_15_ | Verbascoside | Phenolic acids | Phenolic acids | 61276-17-3 | 2 | 9.76E+03 | 7.90E+03 | 1.57E+04 | 9.00E+00 | 9.00E+00 | 9.00E+00 | 1.23E+00 | 4.16E-02 | 7.90E-02 | 8.10E-04 | -1.03E+01 | down |
| pmb0530 | C_21_H_27_N_7_O_14_P_2_ | Nicotinic acid adenine dinucleotide | Nucleotides and derivatives | Nucleotides and derivatives | 53-84-9 | 1 | 1.85E+05 | 2.25E+05 | 2.16E+05 | 4.67E+04 | 7.00E+04 | 5.45E+04 | 1.21E+00 | 1.32E-03 | 7.87E-03 | 2.74E-01 | -1.87E+00 | down |
| mws4163 | C_24_H_42_O_21_ | Nystose | Others | Saccharides and Alcohols | 13133-07-8 | 3 | 2.37E+04 | 1.98E+04 | 1.14E+04 | 4.76E+04 | 7.68E+04 | 6.06E+04 | 1.14E+00 | 2.26E-02 | 4.95E-02 | 3.38E+00 | 1.75E+00 | up |
| Lmyn005812 | C_33_H_56_O_14_ | Gingerglycolipid A | Lipids | Glycerol ester | 145937-22-0 | 1 | 4.38E+05 | 4.36E+05 | 4.04E+05 | 1.62E+05 | 1.63E+05 | 1.86E+05 | 1.23E+00 | 8.89E-05 | 1.43E-03 | 4.00E-01 | -1.32E+00 | down |
| Lmhp008513 | C_33_H_56_O_14_ | 2-α-Linolenoyl-glycerol-1,3-di-O-glucoside* | Lipids | Glycerol ester | - | 1 | 1.28E+04 | 1.81E+04 | 1.98E+04 | 4.06E+03 | 6.73E+03 | 5.33E+03 | 1.17E+00 | 2.13E-02 | 4.81E-02 | 3.18E-01 | -1.65E+00 | down |
| Lmhp008744 | C_33_H_56_O_14_ | 1-α-Linolenoyl-glycerol-2,3-di-O-glucoside* | Lipids | Glycerol ester | - | 1 | 1.24E+04 | 1.78E+04 | 1.87E+04 | 4.83E+03 | 6.57E+03 | 4.64E+03 | 1.18E+00 | 2.28E-02 | 4.99E-02 | 3.28E-01 | -1.61E+00 | down |
| Lmhp009384 | C_33_H_58_O_14_ | 1-Linoleoylglycerol-2,3-di-O-glucoside* | Lipids | Glycerol ester | - | 1 | 7.44E+04 | 8.78E+04 | 7.66E+04 | 3.51E+04 | 3.03E+04 | 3.64E+04 | 1.22E+00 | 2.96E-03 | 1.22E-02 | 4.26E-01 | -1.23E+00 | down |
| pmp001271 | C_33_H_58_O_14_ | 1-Linoleoyl-sn-glycerol-diglucoside* | Lipids | Free fatty acids | - | 1 | 7.98E+04 | 9.84E+04 | 8.19E+04 | 3.28E+04 | 3.17E+04 | 4.15E+04 | 1.20E+00 | 4.36E-03 | 1.60E-02 | 4.07E-01 | -1.30E+00 | down |
| Lmyn006011 | C_33_H_58_O_14_ | Gingerglycolipid B | Lipids | Glycerol ester | 88168-90-5 | 1 | 3.74E+06 | 4.70E+06 | 3.92E+06 | 1.94E+06 | 1.69E+06 | 1.83E+06 | 1.21E+00 | 1.24E-02 | 3.25E-02 | 4.42E-01 | -1.18E+00 | down |
| Lmhp009190 | C_33_H_58_O_14_ | 2-Linoleoylglycerol-1,3-di-O-glucoside* | Lipids | Glycerol ester | - | 1 | 6.72E+04 | 1.68E+05 | 1.04E+05 | 3.40E+04 | 3.38E+04 | 3.86E+04 | 1.11E+00 | 1.20E-01 | 1.83E-01 | 3.14E-01 | -1.67E+00 | down |
| Lmyn006221 | C_33_H_60_O_14_ | Gingerglycolipid C | Lipids | Glycerol ester | - | 2 | 1.75E+05 | 2.16E+05 | 2.23E+05 | 1.03E+05 | 9.49E+04 | 8.45E+04 | 1.20E+00 | 1.04E-02 | 2.87E-02 | 4.60E-01 | -1.12E+00 | down |
| pme2651 | C_21_H_28_N_7_O_17_P_3_ | NADP (Nicotinamide adenine dinucleotide phosphate) | Nucleotides and derivatives | Nucleotides and derivatives | 53-59-8 | 3 | 2.89E+04 | 3.70E+04 | 4.51E+04 | 7.77E+03 | 5.36E+03 | 6.00E+03 | 1.21E+00 | 2.05E-02 | 4.68E-02 | 1.72E-01 | -2.54E+00 | down |

**Supplementary table S7** Differential metabolites in TYD vs TYH

| Index | Formula | Compounds | Class I | Class II | CAS | Level | TJXD-1 | TJXD-2 | TJXD-3 | TJXH-1 | TJXH-2 | TJXH-3 | VIP | p_value | FDR | Fold_Change | Log_2_FC | Type |
| --- | --- | --- | --- | --- | --- | --- | --- | --- | --- | --- | --- | --- | --- | --- | --- | --- | --- | --- |
| pme3200 | C_2_H_7_N_3_ | 1-Methylguanidine | Nucleotides and derivatives | Nucleotides and derivatives | 471-29-4 | 3 | 3.04E+03 | 2.35E+03 | 4.47E+03 | 3.23E+04 | 2.63E+04 | 2.04E+04 | 1.12E+00 | 1.87E-02 | 3.29E-02 | 8.01E+00 | 3.00E+00 | up |
| pme2292 | C_4_H_12_N_2_ | Putrescine | Alkaloids | Alkaloids | 110-60-1 | 3 | 4.85E+05 | 5.18E+05 | 4.68E+05 | 1.79E+05 | 2.08E+05 | 1.99E+05 | 1.13E+00 | 2.73E-04 | 2.55E-03 | 3.98E-01 | -1.33E+00 | down |
[truncated: 94,786 more chars]
